# Transcriptomic insight into the translational value of two murine models in human atopic dermatitis

**DOI:** 10.1038/s41598-021-86049-w

**Published:** 2021-03-23

**Authors:** Young-Won Kim, Eun-A Ko, Sung-Cherl Jung, Donghee Lee, Yelim Seo, Seongtae Kim, Jung-Ha Kim, Hyoweon Bang, Tong Zhou, Jae-Hong Ko

**Affiliations:** 1 Department of Physiology, College of Medicine, Chung-Ang University, Seoul, 06974 Korea; 2 Department of Physiology, School of Medicine, Jeju National University, Jeju, 63243 Korea; 3 Department of Family Medicine, College of Medicine, Chung-Ang University Hospital, Seoul, 06973 Korea; 4 Department of Physiology and Cell Biology, University of Nevada, Reno School of Medicine, Reno, NV 89557 USA

**Keywords:** Computational biology and bioinformatics, Biomarkers

## Abstract

This study sought to develop a novel diagnostic tool for atopic dermatitis (AD). Mouse transcriptome data were obtained via RNA-sequencing of dorsal skin tissues of CBA/J mice affected with contact hypersensitivity (induced by treatment with 1-chloro-2,4-dinitrobenzene) or brush stimulation-induced AD-like skin condition. Human transcriptome data were collected from German, Swedish, and American cohorts of AD patients from the Gene Expression Omnibus database. edgeR and SAM algorithms were used to analyze differentially expressed murine and human genes, respectively. The FAIME algorithm was then employed to assign pathway scores based on KEGG pathway database annotations. Numerous genes and pathways demonstrated similar dysregulation patterns in both the murine models and human AD. Upon integrating transcriptome information from both murine and human data, we identified 36 commonly dysregulated differentially expressed genes, which were designated as a 36-gene signature. A severity score (AD index) was applied to each human sample to assess the predictive power of the 36-gene AD signature. The diagnostic power and predictive accuracy of this signature were demonstrated for both AD severity and treatment outcomes in patients with AD. This genetic signature is expected to improve both AD diagnosis and targeted preclinical research.

## Introduction

Patients with atopic dermatitis (AD) often exhibit an itchy rash, xerosis, skin barrier defects, chronic relapses, and emotional distress, which reduces their quality of life^1^. The diagnosis of AD is generally based on visible clinical symptoms, with limited therapeutic options available for this condition. The most common diagnostic criteria and severity scoring tools are the Hanifin and Rajka criteria^2^ and the SCORing AD (SCORAD) index^3^, respectively.

Various murine models have been developed for studying AD; however, their ability to recapitulate the pathophysiological features and complex clinical manifestations of human AD is limited. In the contact hypersensitivity (CHS) model, hapten 1-chloro-2,4-dinitrobenzene is applied to the skin to stimulate keratinocytes, which produce various biochemical mediators, such as interleukin (IL)-1β and tumor necrosis factor (TNF)-α^4^. These responses promote the migration and maturation of dermal dendritic cells, which then migrate to draining lymph nodes, presenting contact allergens to naïve T cells. In the skin-scratching stimulation (SSS) model, mice exhibit a temporary self-scratching behavior within a few minutes of brush stimulation. This leads to the physiological stimulation of the skin via activation of the substance P signaling pathway, following the binding of tachykinin receptor 1^5,6^. Using these murine models, we previously suggested—from a molecular genetic perspective—that itching is caused by induction of damage to the chemical/physical skin barrier, which is related to the rate of wound healing, particularly in the case of inflammatory reactions, and pain signal intensity^7^. We also noted that pruritus and a skin barrier disorder were representative symptoms of AD. Therefore, we employed these two pruritus murine models, which demonstrate early stages of skin reactions, rather than the chronic AD NC/Nga mouse model^8^.

In this study, we developed objective AD criteria based on molecular signatures that can be applied as potential tools for improving the accuracy of AD diagnosis and evaluating AD treatment outcomes^9^.

## Results

### Gene dysregulation patterns in CHS and SSS murine models

**Figure 1:**
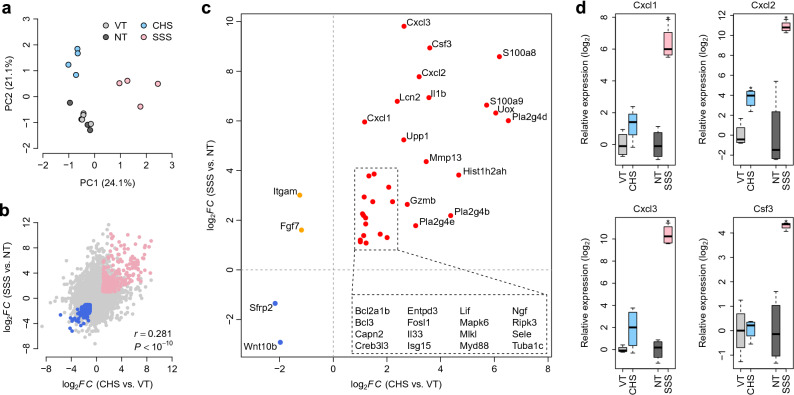
Differential gene expression in contact hypersensitivity (CHS) and skin-scratching stimulation (SSS) murine models. (**a**) Principal component analysis of whole-genome expression. (**b**) Correlation of log_2_-transformed gene expression fold-changes (log_2_FC) between the CHS and vehicle control (VT) groups (X-axis), and SSS and non-treated control (NT) groups (Y-axis). Pink/blue dots denote genes commonly upregulated/downregulated in CHS and SSS samples relative to their expression in the VT and NT samples, respectively. (**c**) Top dysregulated genes within prioritized Kyoto Encyclopedia of Genes and Genomes pathways. X-axis: log_2_FC values between the CHS and VT groups; Y-axis: log_2_FC values between the SSS and NT groups. Red/blue dots denote genes commonly upregulated/downregulated in both murine models, and the orange dots denote genes dysregulated in opposite directions. (**d**) qPCR validation of selected genes. *Indicates the statistical significance at *P* < 0.05.

We prepared 16 polyA-enriched RNA-seq libraries of mouse skin samples with four biological replicates per group (vehicle control, VT; non-treated control, NT; CHS model, and SSS model). In total, 13,259 genes were identified with average expression levels > 1 transcript per million. To assess transcriptome heterogeneity within the different murine models, we conducted principal component analysis of the whole-genome gene expression data, which revealed distinct transcriptome patterns between the VT and CHS and NT and SSS samples (Fig. 1a). However, the first and second principal components of the VT and NT samples did not differ significantly (Fig. 1a), suggesting similar transcriptomic landscapes for these two controls. To identify differentially expressed genes (DEGs) in both murine models, we compared gene expression patterns between the VT and CHS samples, and between the NT and SSS samples. Using the following cut-offs: a false discovery rate (FDR) < 5% and fold-change (FC) > 2, 993 upregulated and 1,214 downregulated DEGs were detected in the CHS samples, relative to the VT samples (Supplementary Tables S1 and S2). Comparatively, 1,608 and 999 DEGs were upregulated and downregulated, respectively, in the SSS samples, relative to the NT samples (Supplementary Tables S3 and S4). Interestingly, FC gene expression values in the VT and CHS groups were positively correlated with those observed between the NT and SSS groups (Pearson’s correlation (*r*) = 0.281, *P* < 10^−10^). In both models, 292 and 293 DEGs were commonly upregulated and downregulated, respectively (Fig. 1b), suggesting that a considerable number of DEGs shared similar dysregulation patterns in the two murine models.

### Dysregulated pathways in the murine models

To investigate transcriptomic alterations in the two murine models, we examined dysregulated pathways based on the Kyoto Encyclopedia of Genes and Genomes (KEGG) pathway database annotations^10–12^. For each pathway, we obtained a pathway score using the FAIME algorithm, with a higher pathway score indicating higher overall expression. In total, 39 upregulated and 49 downregulated KEGG pathways (*t*-test: corrected *P* < 0.05) were detected in CHS samples compared with VT samples (Supplementary Tables S5 and S6). Additionally, 46 and 23 pathways were found to be upregulated and downregulated, respectively, in SSS samples, relative to the NT samples (Supplementary Tables S7 and S8). We then investigated the dysregulated pathways shared between the two murine models. Eleven KEGG pathways were commonly upregulated in both models, including TNF signaling pathway, IL-17 signaling pathway, RIG-I-like receptor signaling pathway, apoptosis, and necroptosis (Supplementary Fig. S1). Lipoic acid metabolism and Wnt signaling pathway, were commonly downregulated in the two murine models (Supplementary Fig. S1). Interestingly, the Rap1 signaling pathway was dysregulated in a contradictory manner in the two murine models (downregulated in the CHS model and upregulated in the SSS model) (Supplementary Fig. S1). We further investigated commonly dysregulated DEGs within these prioritized pathways. In total, 32 DEGs were commonly upregulated in the CHS and SSS samples, including *Cxcl1*, *Cxcl2*, *Cxcl3*, *Csf3*, *Il1b*, *Mmp13*, *S100a8*, and *S100a9* (Fig. 1c). *Sfrp2* and *Wnt10b* were commonly downregulated in both murine models (Fig. 1c), while *Fgf7* and *Itgam* were downregulated in the CHS model and upregulated in the SSS model (Fig. 1c). Using quantitative polymerase chain reaction (qPCR), we further validated the expression patterns of *Cxcl1*, *Cxcl2*, *Cxcl3*, and *Csf3*, all of which exhibited significant upregulation in the SSS samples, compared with the NT samples (one-tailed *t*-test: *P* = 1.2 × 10^−4^ for *Cxcl1*; *P* = 8.8 × 10^−3^ for *Cxcl2*; *P* = 3.7×10^−6^ for *Cxcl3*; *P* = 6.7 × 10^−3^ for *Csf3*). Significant or marginal upregulation of *Cxcl1*, *Cxcl2*, and *Cxcl3* was also observed in the CHS samples, compared with the VT samples (one-tailed *t*-test: *P* = 5.4 × 10^−2^ for *Cxcl1*; *P* = 1.4 × 10^−3^ for *Cxcl2*; *P* = 6.8 × 10^−2^ for *Cxcl3*) (Fig. 1d).

### Comparison of pathway dysregulation between murine models and human AD

**Figure 2:**
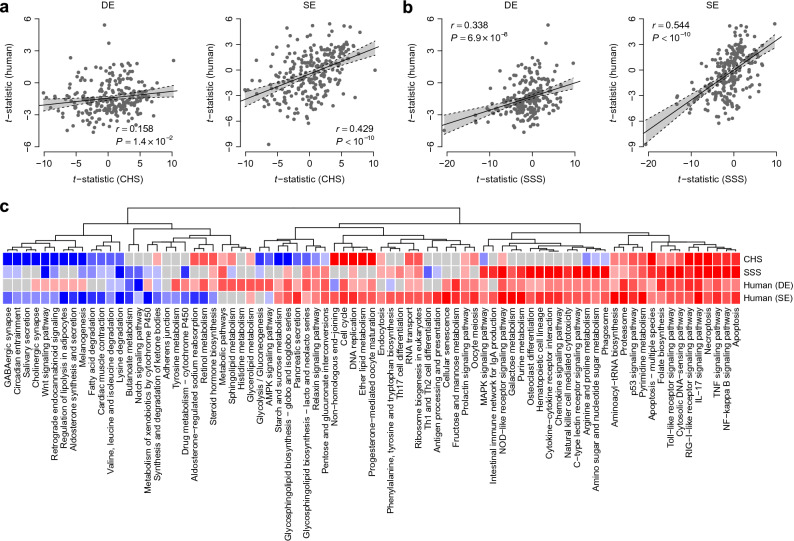
Kyoto Encyclopedia of Genes and Genomes (KEGG) pathway-level comparison between human cohorts and (**a**) contact hypersensitivity (CHS) model or (**b**) skin-scratching stimulation (SSS) model. Each dot represents one KEGG pathway. (**c**) Heatmap of the t-statistic. The red color indicates a positive *t*-statistic, and the blue color indicates a negative *t*-statistic.

To evaluate the extent to which the CHS and SSS murine models translationally recapitulated the pathology of human AD, we compared the transcriptomic profiles of the murine models with those of the German (DE)^13^ and Swedish (SE) AD cohorts^14^. For both the human cohorts, KEGG pathway scores were computed for the control skin and AD lesional skin samples using the *FAIME* algorithm. We utilized a Student’s *t*-test to prioritize the dysregulated KEGG pathways between the control and AD skin samples, and recorded the *t*-statistic value for each comparison. For a given pathway, a positive t-statistic suggested the upregulation of a potential pathway in AD skin, relative to the control. A negative t-statistic suggested the downregulation of a potential pathway in AD. The t-statistics of both human AD cohorts were positively correlated with those of the VT and CHS samples (*r* = 0.158, *P* = 1.4 × 10^−2^ for DE; *r* = 0.429, *P* < 10−^-10^ for SE) (Fig. 2a). A similar positive correlation was observed when comparing the human AD cohorts and the SSS model (*r* = 0.338, *P* = 6.9 × 10^−8^ for DE; *r* = 0.544, *P* < 10^−10^ for SE) (Fig. 2b). The results suggest that the pathways dysregulated in the murine models were more likely to be dysregulated in human AD skin, and that many pathways shared similar dysregulation patterns in murine models and human AD. Figure 2c illustrates several KEGG pathways commonly upregulated in the human AD cohorts and both murine models, including TNF signaling pathway, IL-17 signaling pathway, RIG-I-like receptor signaling pathway, necroptosis, and apoptosis. In contrast, the Wnt signaling pathway was commonly downregulated in human AD lesional skin from the SE cohort (but not the DE cohort) and in both murine models (Fig. 2c). We also observed several pathways exhibiting upregulation in both human cohorts but not in the murine models, such as those related to fructose and mannose metabolism (Fig. 2c).

To evaluate the effect of age on the translational value of the two murine models, one more AD cohort from the United States (US1)^15^ was investigated, which included both pediatric and adult subjects. We found that the *t*-statistics of the KEGG pathways of both the pediatric and adult groups were positively correlated with those of the VT and CHS samples (*r* = 0.526, *P* < 10^−10^ for the pediatric group; *r* = 0.348, *P* = 2.5 × 10^−8^ for the adult group) (Supplementary Fig. S2a). A similar positive correlation was observed when comparing the US1 cohort with the SSS model (*r* = 0.295, *P* = 2.9 × 10^−6^ for the pediatric group; *r* = 0.479, *P* < 10^−10^ for the adult group) (Supplementary Fig. S2b). These results suggest that the translational power of the two murine models is not likely to be affected by the age of the AD patients.

### Translational contribution of murine models to AD biomarker development

**Figure 3:**
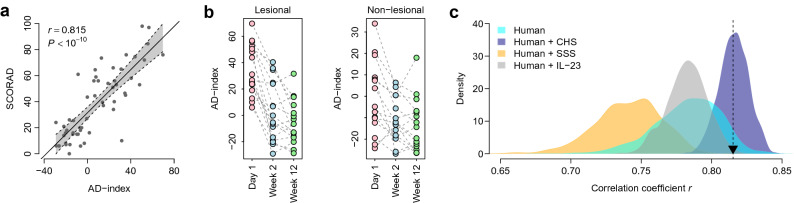
Prediction of atopic dermatitis (AD) severity using the 36 gene-based AD index. (**a**) Correlation between AD index and SCORAD index for lesional samples from the US2 cohort. (**b**) Comparison of pre- and post-treatment AD indexes. (**c**) Evaluation of the translational value of murine models in AD biomarker development. The cyan area indicates the distribution of correlation coefficients *r* for 1,000 resampled gene signatures randomly selected from the human gene pool containing AD-related genes commonly dysregulated in the German (DE) and Swedish (SE) cohorts. The navy and orange areas indicate the distribution of *r* for 1,000 resampled gene signatures randomly selected from the human + contact hypersensitivity (CHS) and human + skin-scratching stimulation (SSS) models, respectively. The grey area indicates the distribution of *r* for 1,000 resampled gene signatures randomly selected from the Human + IL-23 gene pool. The black triangle represents the *r*-value of our 36-gene signature.

To understand whether incorporation of the transcriptomic information from the murine models may potentially aid in the development of biomarkers for human AD, we focused on dysregulated DEGs shared between the murine models and the human AD cohorts. As shown in Figure 1b, we identified 292 commonly upregulated and 293 commonly downregulated genes in the CHS and SSS models. We mapped these genes to their corresponding human orthologs and found that 36 of the genes were also dysregulated in the DE and SE cohorts (FDR < 10% and FC > 1.5). We designated these 36 genes as a 36-gene signature (Table 1) and assigned a weight of 1 and -1 to upregulated and downregulated DEGs in human lesional skin, respectively. To validate the diagnostic power of the 36-gene signature, we investigated its predictive performance using the US2 cohort^16^ for independent validation. To statistically assess the predictive power of the 36-gene signature, a severity score (AD index) was assigned to each human sample. AD index scores were significantly correlated with the SCORAD index scores for both lesional (*r* = 0.815, P < 10^−10^) (Fig. 3a) and non-lesional (*r* = 0.639, *P* = 4.8 × 10^−7^) samples (Supplementary Fig. S3). AD index scores of lesional samples were significantly higher than those of non-lesional samples (*t*-test: *P* = 5.4 × 10^−5^) (Supplementary Fig. S4). Finally, clinical treatment outcome was associated with AD index scores in both lesional and non-lesional skin samples. AD index scores of human skin subjected to the 2-week treatment were significantly lower than baseline values (paired *t*-test: *P* = 3.4 × 10^−6^ for lesional samples and *P* = 5.2 × 10^−3^ for non-lesional samples) (Fig. 3b). However, AD index scores of lesional skin subjected to the 12-week treatment were only marginally lower than those of the skin subjected to the 2-week treatment (paired *t*-test: *P* = 5.4 × 10^−2^). AD index scores of non-lesional skin between the 2- and 12-week time points did not differ significantly (paired *t*-test: *P* = 3.7 × 10^−1^) (Fig. 3b). These results suggest that the 36 gene-based AD index may potentially serve as a proxy for an anti-AD therapeutic response.

**Table 1:** Thirty-six-gene atopic dermatitis signature.

Gene symbol	Gene description	Weight
*AQP3*	Aquaporin 3 (Gill blood group)	1
*DSC2*	Desmocollin 2	1
*FABP5*	Fatty acid binding protein 5 (psoriasis-associated)	1
*FSCN1*	Fascin actin-bundling protein 1	1
*GALNT6*	Polypeptide *N*-acetylgalactosaminyltransferase 6	1
*GCH1*	GTP cyclohydrolase 1	1
*HBEGF*	Heparin-binding EGF-like growth factor	1
*ISG15*	ISG15 ubiquitin-like modifier	1
*KLK6*	Kallikrein-related peptidase 6	1
*KRT16*	Keratin 16, type I	1
*KRT6A*	Keratin 6A, type II	1
*KRT6B*	Keratin 6B, type II	1
*MAPK6*	Mitogen-activated protein kinase 6	1
*NCAPG*	Non-SMC condensin I complex, subunit G	1
*OAS3*	2′-5′-Oligoadenylate synthetase 3, 100-kDa	1
*RAB31*	RAB31, member RAS oncogene family	1
*S100A8*	S100 calcium binding protein A8	1
*S100A9*	S100 calcium binding protein A9	1
*SELE*	Selectin E	1
*SLC7A5*	Solute carrier family 7 (amino acid transporter light chain, L system), member 5	1
*SPRR1A*	Small proline-rich protein 1A	1
*SPRR1B*	Small proline-rich protein 1B	1
*TNC*	Tenascin C	1
*TUBB2A*	Tubulin, beta 2A class IIa	1
*TUBB3*	Tubulin, beta 3 class III	1
*UPP1*	Uridine phosphorylase 1	1
*ZWILCH*	Zwilch kinetochore protein	1
*BTC*	Betacellulin	-1
*CBX7*	Chromobox homolog 7	-1
*FAM117A*	Family with sequence similarity 117, member A	-1
*FRZB*	Frizzled-related protein	-1
*KRT15*	Keratin 15, type I	-1
*PIK3C2G*	Phosphatidylinositol-4-phosphate 3-kinase, catalytic subunit type 2 gamma	-1
*RYR1*	Ryanodine receptor 1 (skeletal)	-1
*TNNI2*	Troponin I type 2 (skeletal, fast)	-1
*VIPR1*	Vasoactive intestinal peptide receptor 1	-1

To determine whether the transcriptomic information from the murine model can contribute to the development of AD skin biomarkers, a resampling test was conducted following the scheme suggested by Venet et al.^17^. We generated a human gene pool (designated as Human in Fig. 3c) containing AD-related genes commonly dysregulated in the DE and SE cohorts. We then artificially constructed 1,000 random gene signatures, identical in size to that of the 36-gene signature, by randomly selecting genes from the human AD-related gene pool. For each resampled signature, we calculated a severity score based on the gene expression within the resampled signature for all the lesional samples. The correlation between SCORAD index scores and gene expression-based severity scores was recorded for each random gene signature, which measured the predictive power of the random gene set. The correlation coefficient r of our 36-gene signature was significantly higher than that of the artificial gene signatures (right-tailed: *P* = 0.046) (Fig. 3c). The resampling test suggested that including the transcriptomic information from the murine model improved the predictive accuracy of the AD severity gene signature.

To determine whether the CHS or SSS murine model had a greater potential to improve the performance of the AD gene signature, we conducted two more rounds of the resampling test. We generated a gene pool (Human + CHS) containing the DEGs commonly dysregulated in both the CHS murine model and human cohorts, and artificially constructed 1000 random gene signatures by randomly selecting 36 genes from the Human + CHS gene pool. We computed the severity score for each resampled signature, and the correlation between the SCORAD index scores and severity scores was recorded for each random gene signature. We also generated a gene pool (Human + SSS) containing the DEGs commonly dysregulated in both the SSS murine model and the human cohorts. A 1,000-time resampling test based on the Human + SSS gene pool was conducted using the aforementioned method. The predictive power of the Human + CHS signature was significantly higher than that of the signatures generated from the Human + SSS gene pool and the human AD-related gene pool (*t*-test: *P* < 10^−10^) (Fig. 3c). This finding indicated that the incorporation of the CHS murine model’s transcriptomic information substantially benefited the development of AD biomarkers.

A previous study suggests that the transcriptome of IL-23-injected mice show strong homology with the human AD transcriptome and may best represent the AD phenotype^18^. To determine whether the IL-23-injected murine model had a potential to improve the performance of the AD gene signature, we generated a gene pool (Human + IL-23) containing the DEGs commonly dysregulated in both the IL-23-injected murine model and human cohorts. A 1,000-time resampling test based on the Human + IL-23 gene pool was conducted using the aforementioned method. We found that the predictive power of the Human + IL-23 signatures was significantly higher than that of the Human + SSS signatures (*t*-test: *P* < 10^−10^), but significantly lower than that of the Human + CHS signatures (*t*-test: *P* < 10^−10^) (Fig. 3c). In addition, we did not find significant difference between Human and Human + IL-23 gene pools (*t*-test: *P* = 0.780) (Fig. 3c). These findings further suggest the superior translational value of the CHS murine model.

### Superior predictive power of the 36-gene signature

**Figure 4:**
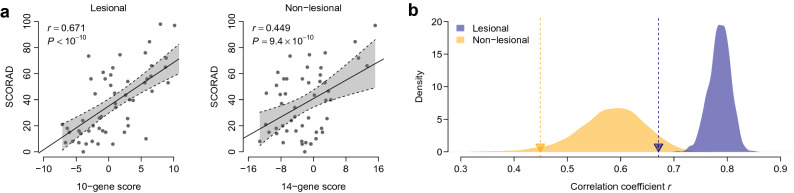
Comparison between the predictive power of the 36-gene signature and several published atopic dermatitis (AD) marker genes. (**a**) Correlation between SCORAD and severity scores computed for published marker genes. (**b**) Predictive power of our 36-gene signature compared with that of published AD marker genes. The navy- and orange-colored areas indicate the distribution of *r* for 1000 resampled gene signatures, randomly selected from our 36-gene signature, with a size identical to that of the published 10- and 14-gene signatures, respectively. The navy and orange triangles represent the *r*-values of the published 10- and 14-gene signatures, respectively.

We compared the predictive power of our 36-gene signature against the following AD severity biomarkers published by Ungar et al.^19^: a 10-gene signature for lesional skin, and a 14-gene signature for non-lesional skin (Supplementary Table S9). The 10-gene- and 14-gene-based severity scores were significantly correlated with the SCORAD index scores for both lesional and non-lesional samples from the US2 cohort (*r* = 0.671, *P* < 10^−10^ for lesional samples; *r* = 0.449, *P* = 9.4 × 10^−10^ for non-lesional samples) (Fig. 4a). To compare the performances of the published 10-gene signature and our proposed 36-gene signature, a resampling test was performed 1,000 times by randomly selecting ten genes from our proposed 36-gene signature. The predictive power of the random 10-gene signature was significantly higher than that of the published 10-gene signature for lesional samples from the US2 cohort (left-tailed: *P* < 0.001) (Fig. 4b). We also applied a resampling test to compare the performance of our 36-gene signature with that of the published 14-gene signature for non-lesional samples. As shown in Figure 4b, the predictive power of the random 14-gene signature was significantly higher than that of the published 14-gene signature for non-lesional samples from the US2 cohort (left-tailed: *P* = 0.023). These results indicated the superior AD severity-predicting power of our proposed 36-gene signature.

## Discussion

We integrated the transcriptomic information obtained for the murine models with that of human AD cohorts and identified a commonly dysregulated 36-gene signature. The 36-gene signature demonstrated sufficient diagnostic power to accurately predict the severity of AD and treatment outcomes in AD patients.

AD is diagnosed based on its clinical features; therapeutic strategies for AD are limited to the hydration of the skin, topical corticosteroid application, or suppression of the immune system. Therefore, a reliable method for the molecular diagnosis of AD is urgently required. The significant role of ion channel in AD has been determined in previous studies, for example, various transient receptor potential (TRP) channels including *TRPA1*, *TRPV1-4*, and *TRPM8*, have been shown to be responsible for the transmission of itch sensation^20–22^. Although the alterations of the transcriptome and integrative analysis of murine and human transcriptomes in disease conditions have been extensively studied, the number of channels in each subfamily among AD animal models largely varies and datasets from regions of interest and approaches for transcriptome analysis differ. In our study, to diminish the discrepancies between various animal AD models and improve the accuracy of AD diagnosis, we identified commonly dysregulated ion channel gene signature by including two animal AD models. The informative transcriptomes of the CHS and SSS models obtained by us will serve as primary resources for providing insights into the molecular changes associated with AD and AD-related biological studies.

In our previous study^7^, keratinization was found to be a commonly upregulated biological process in both the CHS and SSS murine models, although not all the observed skin lesions were severe. Our 36-gene signature included four KRT genes, among which *KRT6A*, *KRT6B*, and *KRT16* were upregulated and *KRT15* was downregulated in AD samples. The *KRT6* and *KRT16* keratin gene pair is constitutively expressed and activated in epidermal hyperproliferation^23,24^. However, *KRT15* encodes a type I keratin, which does not serve as a natural type II expression partner, and its expression is not compatible with keratinocyte activation. In accordance with our study findings, *KRT15* is known to be downregulated in the hyperproliferating epidermis to maintain the hyperplastic phenotype^25,26^.

Skin barrier dysfunction in AD is associated with alterations in key genes involved in keratinocyte differentiation and formation of structural proteins for skin barrier elements. We found that genes encoding structural proteins (TUBB, KRT, DSC, and FSCN) and epidermal differentiation complex components (SPRR1A and SPRR1B) known to be associated with AD^27–30^ were upregulated in AD samples. We also found that genes encoding the Ca^2+^-binding proteins S100A8 and S100A9, which are members of an inflammatory protein complex, were upregulated in AD samples. This is consistent with previous reports of their upregulation in AD^31^ and psoriasis^32^. The intracellular concentration of Ca^2+^ regulates keratinocyte differentiation, and alterations in the extracellular Ca^2+^ gradient in the epidermis may be responsible for upregulating a group of S100 proteins, including S100A8 and S100A9^33^.

We observed that *Sfrp2* was downregulated in both murine models, whereas *Sfrp4* was only downregulated in the CHS model. SFRP family proteins encoded by *Sfrp* genes bind Wnt ligands, thereby inhibiting the Wnt signaling pathway and subsequently controlling cell proliferation and differentiation^34^. SFRP4, in particular, which is reportedly downregulated in the lesional skin of murine psoriasis models and human psoriasis patients, has also been reported to inhibit keratinocyte hyperproliferation and epidermal hyperplasia^35^.

A previous study has identified genes that are differentially expressed in AD compared to normal skin specimens from several types of animal AD models and humans with AD^18^. Among 6 common AD-like murine models, an IL-23-injected mouse model showed a transcriptomic profile with the highest similarity to the human AD transcriptome. This model shows remarkable innate immune activation and some epidermal alterations, increased neutrophil counts, and sparse amounts of eosinophils and mast cells, which can be found in human AD patients^18^. In our study, we found many pathways with a similar dysregulation pattern in both the CHS and SSS murine models and human AD patients and revealed that including information obtained from murine models improve the accuracy of the gene signature for predicting AD severity.

In another previous study, based on meta-analysis derived atopic dermatitis (MADAD) transcriptome, Ewald et al. identified a robust AD signature composed of 19 genes^36^. Sixteen of these nineteen human genes can be successfully mapped to mouse orthologs. We found that the expression fold changes of these MADAD genes were positively correlated with those of the corresponding mouse orthologous genes in both the CHS and SSS models (Supplementary Fig. S5). Moreover, 6 and 7 genes out of the 16 mouse orthologs were significantly dysregulated in the CHS and SSS models, respectively (Supplementary Fig. S6); this suggests the strong intrinsic connection between the MADAD and CHS/SSS transcriptomes.

Although our study was limited to European and American AD patients, recent studies have reported a unique skin phenotype in Asian AD patients, which is a combination of that observed in European and American atopic and psoriasis patients characterized by increased TH17/TH22 polarization. Hence, our 36-gene signature is expected to further improve our understanding of AD in Asian patients^37^.

Animal models do not completely reflect the transcriptomic and gene pathways activated in human AD skin, resulting in inconsistent non-clinical and clinical AD trial results. The focus of our study was to integrate AD diagnostic criteria to overcome these inconsistencies, and our 36-gene signature was validated for use in further diagnostic and translational studies involving AD. The findings of our study provide a useful tool for AD diagnosis or for screening compounds in the development of targeted AD therapies.

## Methods

### Murine model transcriptome data

The details of our experiment have been described previously^7^. The experiments were approved by the ethics committee of Chung-Ang University, Seoul, Korea (review numbers: 2018-00082 and 2018-00083). The CHS model was generated using a method modified from a local lymph node assay^38,39^, and the SSS model was generated using a method described previously, with modifications^5,6^. All methods were conducted in accordance with IACUC guidelines and regulations for animal testing.

Briefly, total RNAs were extracted from dorsal skin tissues (four samples/group) using TRIzol Reagent (Invitrogen, Carlsbad, CA, USA) according to the manufacturer’s instructions. Expression of all annotated mouse mRNAs in the Ensembl database^40^ was quantified using the Sailfish pipeline^41^ with default settings.

### Quantitative polymerase chain reaction (qPCR)

All reactions were performed according to the manufacturer’s instructions. cDNA was synthesized using the RNA to cDNA EcoDry Premix (Double Primed) (Clontech Laboratories Inc., Kusatsu, Japan), and quantitative polymerase chain reaction (qPCR) was performed using LightCycler FastStart DNA Master SYBR Green I (Roche, Basel, Switzerland) on a LightCycler 2.0 instrument (Roche).

### Determination of DEGs using mouse RNA-seq data

The edgeR algorithm^42^ was employed with default settings to identify DEGs (CHS vs. VT and SSS vs. NT) using the mouse RNA-seq data. Genes with FDR < 5% and FC values > 2 were deemed to be differentially expressed.

### Human microarray data

The following three human AD cohorts from the Gene Expression Omnibus database^43^ were investigated in this study: DE, based on the Illumina HumanHT-12 V3.0 expression beadchip (GSE60709)^13^; SE, based on the Affymetrix Human Genome U133A Array (GSE6012)^14^; US1, based on the Affymetrix Human Genome U133 Plus 2.0 Array (GSE107361)^15^; and US2, based on the Affymetrix Human Genome U133 Plus 2.0 Array (GSE58558)^16^. Fourteen control and twelve AD lesional skin samples from the DE cohort, as well as ten control and ten AD lesional skin samples from the SE cohort, were included. The DE and SE cohorts were used to prioritize DEGs/pathways between the lesional AD and control samples. The US1 cohort was used to evaluate the effect of age on the translational value of our murine models. In total, there were 18 pediatric control samples, 19 pediatric AD lesional samples, 11 adult control samples, and 20 adult lesional sample from the US1 cohort. The US2 cohort was used as the validation dataset; it contained 56 lesional and 53 non-lesional skin biopsy specimens obtained from 19 AD patients at three separate time points: day 1 (baseline), week 2 (after 2 weeks of cyclosporine treatment), and week 12 (after 12 weeks of cyclosporine treatment). For a gene with multiple probes/probesets, the geometric mean of all probes/probesets mapped to the gene was used to measure the expression level. The SAM algorithm^44^ was used to compare the log2-transformed gene expression between lesional AD and control samples. FDR was controlled using the q-value method^45^. Genes with FDR < 10% were deemed to be differentially expressed.

### Pathway score

The FAIME algorithm^46^ was applied to compute the gene expression-based pathway scores for samples from the murine models and human cohorts. The FAIME tool calculated the pathway scores using the rank-weighted gene expression of individual samples, converting the transcriptomic data of each sample to the pathway-level information. Student’s t-tests were performed to prioritize the dysregulated pathways between the control and AD samples.

### AD index

We followed a scoring scheme used in our previous studies^47–50^ to assign each human patient an AD index, which is a linear combination of weighted gene expression values:

$$AD=\sum_{i=1}^{n}{w}_{i}({e}_{i}-{\mu }_{i})/{\tau }_{i}$$

where n is the number of genes; *w*_*i*_ is the weight of gene *i* (1 and − 1 for upregulated and downregulated genes, respectively); *e*_*i*_ is the expression level of gene *i*; and *μ*_*i*_ and *τ*_*i*_ are the mean and standard deviation of the gene expression values for gene *i* across all samples, respectively. A higher AD index implies a more severe AD status.

## Data availablity

Human cohort datasets related to this study can be found at the Gene Expression Omnibus database. RNA sequencing data are available from the corresponding author upon request.

## Supplementary Information

Supplementary Figures.

Supplementary Tables.
