# Supplementary material for: Transcriptomic insight into the translational value of two murine models in human atopic dermatitis
Source: Sci Rep. 2021 Mar 23;11:6616. doi: 10.1038/s41598-021-86049-w (PMC7988112; doi:10.1038/s41598-021-86049-w)
Supplement: Supplementary file 1 — Supplementary Figures. [file 41598_2021_86049_MOESM1_ESM.pdf]

Transcriptomic insight into the translational value of two murine models in human atopic dermatitis

Young-Won Kim[1,+], Eun-A Ko[2,+], Sung-Cherl Jung[2], Donghee Lee[1], Yelim Seo[1], Seongtae Kim[1], Jung-Ha Kim[3], Hyoweon Bang[1], Tong Zhou[4,*], and Jae-Hong Ko[1,*]

[1]Department of Physiology, College of Medicine, Chung-Ang University, Seoul 06974, Korea
[2]Department of Physiology, School of Medicine, Jeju National University, Jeju 63243, Korea
[3]Department of Family Medicine, College of Medicine, Chung-Ang University Hospital, Seoul 06973, Korea
[4]Department of Physiology and Cell Biology, University of Nevada, Reno School of Medicine, Reno, NV 89557, USA

[*]corresponding.akdongyi01@cau.ac.kr; tongz@med.unr.edu
[+]these authors contributed equally to this work

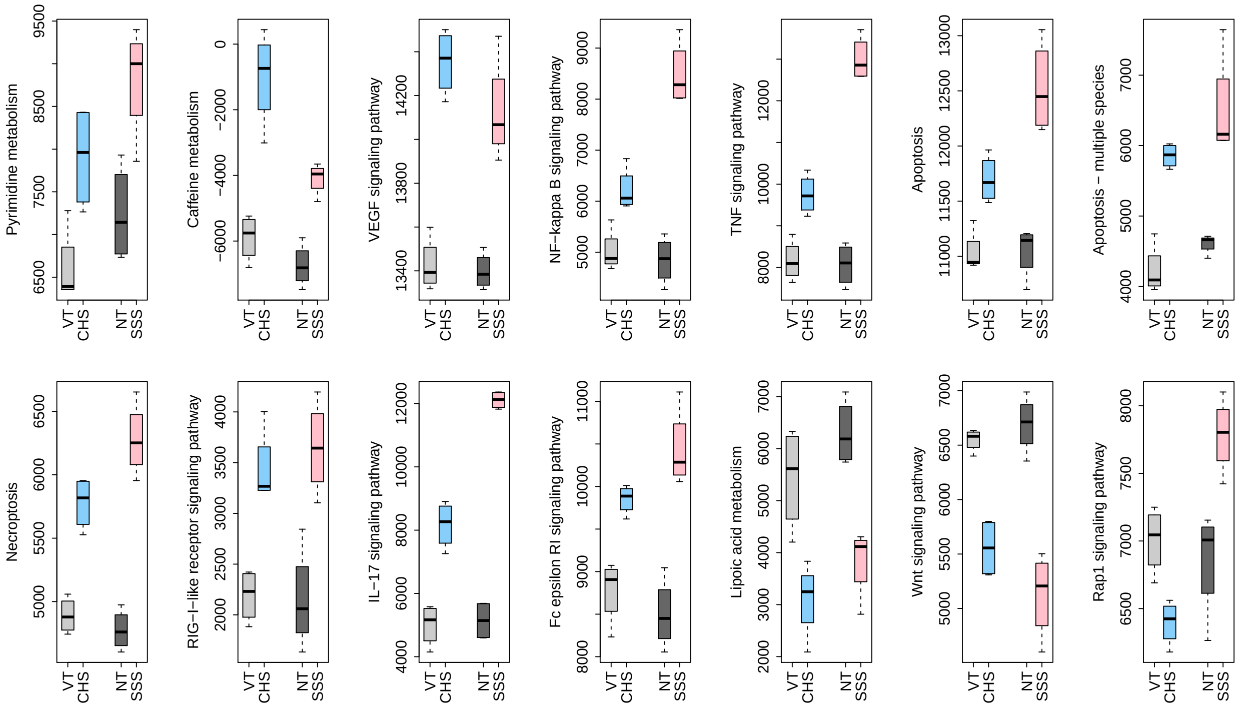

Supplementary Figure S1. The common dysregulated KEGG pathways in both the CHS and SSS models. In total, 2 and 11 pathways were found to be commonly downregulated and upregulated in both models, respectively. One pathway (Rap1 signaling pathway) was found to be dysregulated in opposite directions in the two murine models.

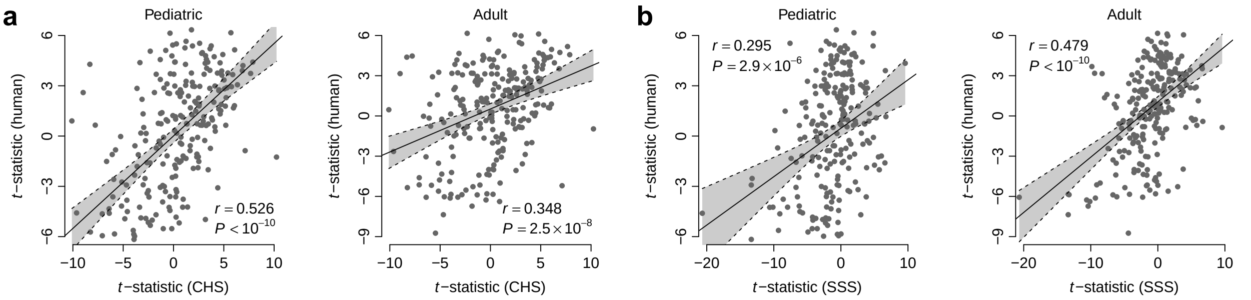

Supplementary Figure S2. Pathway level comparison of the murine models with the pediatric and adult human AD in the US1 cohort. (a) Comparison between the CHS model and human AD. (b) Comparison between the SSS model and human AD.

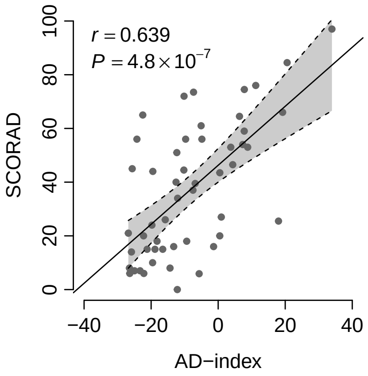

Supplementary Figure S3. The correlation between AD-index and SCORAD score for the non-lesional samples from the US2 cohort.

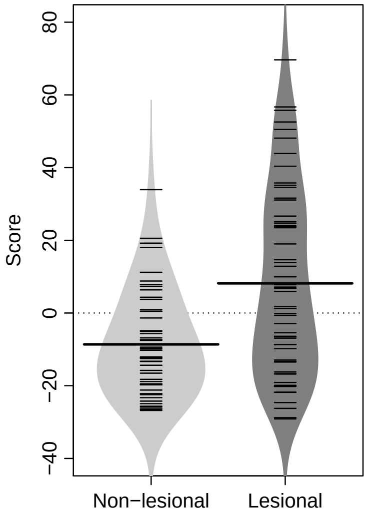

Supplementary Figure S4. Comparison of AD-index between the lesional and non-lesional samples from the US2 cohort.

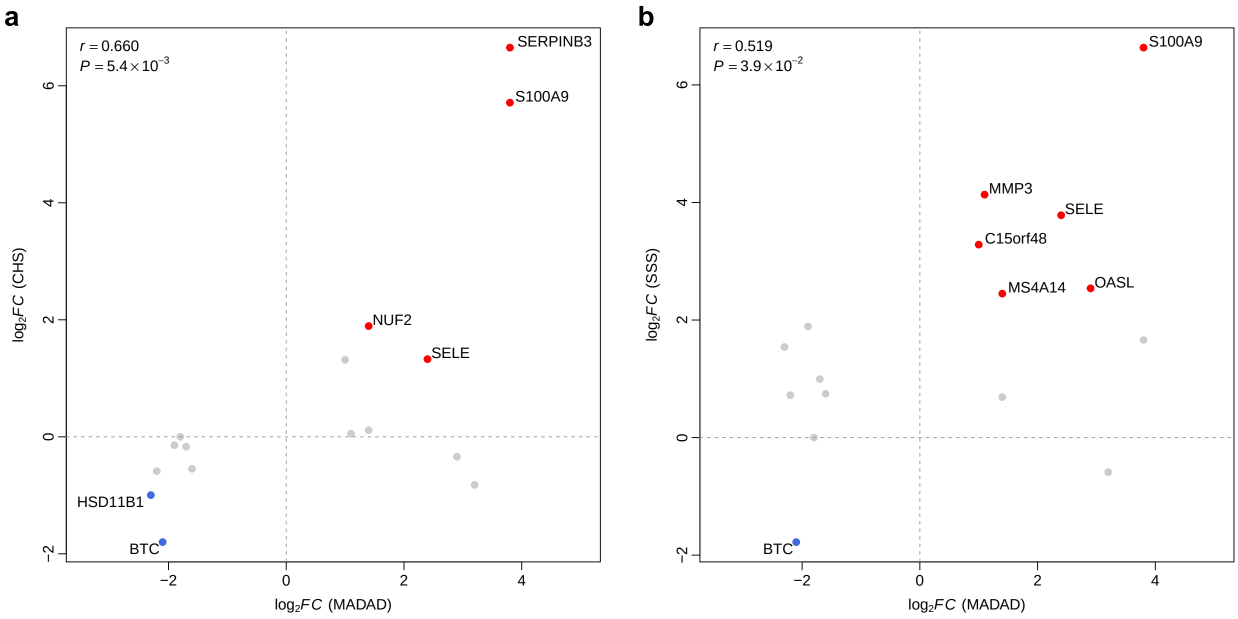

Supplementary Figure S5. Comparison in gene expression fold change between the MADAD and CHS/SSS transcriptomes. The red/blue dots denote the genes commonly upregulated/downregulated in the MADAD and murine model transcriptomes. Only the 19 genes identified by Ewald et al. were included in this figure. (a) Correlation in gene expression fold change between the MADAD and CHS data. (b) Correlation in gene expression fold change between the MADAD and SSS data.
