# Supplementary material for: Transcriptomic insight into the translational value of two murine models in human atopic dermatitis
Source: Sci Rep. 2021 Mar 23;11:6616. doi: 10.1038/s41598-021-86049-w (PMC7988112; doi:10.1038/s41598-021-86049-w)
Supplement: Supplementary file 2 — Supplementary Tables. [file 41598_2021_86049_MOESM2_ESM.pdf]

Transcriptomic insight into the translational value of two murine models in human atopic dermatitis

Young-Won Kim[1,+], Eun-A Ko[2,+], Sung-Cherl Jung[2], Donghee Lee[1], Yelim Seo[1], Seongtae Kim[1], Jung-Ha Kim[3], Hyoweon Bang[1], Tong Zhou[4,*], and Jae-Hong Ko[1,*]

[1]Department of Physiology, College of Medicine, Chung-Ang University, Seoul 06974, Korea
[2]Department of Physiology, School of Medicine, Jeju National University, Jeju 63243, Korea
[3]Department of Family Medicine, College of Medicine, Chung-Ang University Hospital, Seoul 06973, Korea
[4]Department of Physiology and Cell Biology, University of Nevada, Reno School of Medicine, Reno, NV 89557, USA

[*]corresponding.akdongyi01@cau.ac.kr; tongz@med.unr.edu
[+]these authors contributed equally to this work

Supplementary Table S1. The genes upregulated in the CHS samples compared with the VT controls

Gene	logFC	logCPM	LR	PValue	FDR
Lpo	6.8907	5.1203	782.6235	3.2365E-172	7.0655E-168
Gk	3.3754	5.8172	606.7256	5.7669E-134	6.2949E-130
Stfa2	6.6995	6.4030	533.1258	5.9008E-118	4.294E-114
Klk6	5.3019	8.8230	513.7003	9.9343E-114	5.4219E-110
Slc5a8	2.7303	5.9622	459.6439	5.7463E-102	2.50896E-98
Sprr2f	5.4802	4.9058	357.3950	1.03949E-79	3.7822E-76
Sprr2a1	4.9062	5.3165	352.0379	1.52534E-78	4.75711E-75
Lce3f	7.5007	3.5000	339.9123	6.66748E-76	1.81947E-72
Serpinb11	4.4976	4.8124	328.9507	1.62681E-73	3.94611E-70
Tcf23	7.6751	4.3653	321.8869	5.62204E-72	1.22735E-68
Slpi	5.7019	6.0873	305.3014	2.30577E-68	4.57611E-65
Sprr2d	5.8732	8.0865	297.0386	1.45536E-66	2.64766E-63
Rptn	7.0508	10.1947	287.9160	1.41462E-64	2.37559E-61
Sprr2a2	6.2388	11.6373	278.7670	1.39411E-62	2.17392E-59
Sprr2a3	6.4293	6.9259	275.3664	7.68022E-62	1.11778E-58
Ehf	2.1065	6.6961	255.5977	1.56366E-57	2.13352E-54
Smox	2.3627	6.6902	242.8275	9.51022E-55	1.22128E-51
Sprr2e	6.2158	6.2135	233.1604	1.21938E-52	1.47891E-49
Slc5a1	2.0937	6.8120	229.0461	9.62446E-52	1.10585E-48
Sox7	2.2338	5.1398	220.7417	6.23163E-50	6.80214E-47
Lce3d	5.7038	4.9117	219.3845	1.23211E-49	1.28087E-46
Lce3a	6.2337	3.6853	216.3661	5.61132E-49	5.56821E-46
Galnt6	3.1818	5.8326	215.2998	9.58674E-49	9.09948E-46
Stfa3	6.6862	8.5916	214.3347	1.55672E-48	1.36327E-45
Lce3b	6.8055	4.0825	214.3290	1.56116E-48	1.36327E-45
Slc28a3	1.8405	5.6853	213.8771	1.95901E-48	1.64489E-45
2610528A11Rik	6.6274	6.2106	211.8733	5.36018E-48	4.33401E-45
Serpinb3a	4.7753	5.3240	208.3895	3.08481E-47	2.40516E-44
Lce3e	6.4650	4.1036	205.5184	1.30518E-46	9.8253E-44
Sprr2b	7.7949	5.7286	204.8769	1.80153E-46	1.31097E-43
Chil1	4.4002	9.2985	191.2278	1.71525E-43	1.20792E-40
Sprr2h	4.8230	7.5693	190.8326	2.09221E-43	1.42734E-40
Il1f6	3.2371	6.5816	188.0866	8.31769E-43	5.50253E-40
Pla2g4e	3.0696	6.3300	185.8925	2.50586E-42	1.60898E-39
Elf3	2.9536	3.2794	184.6513	4.67644E-42	2.9169E-39
Scnn1b	1.9060	4.5482	183.9944	6.50613E-42	3.94543E-39
Plbd1	1.7030	7.7224	179.8576	5.20615E-41	3.07177E-38
Defb3	7.7359	4.3116	176.6792	2.57346E-40	1.47845E-37
2310046K23Rik	1.8591	9.7824	174.1545	9.15899E-40	5.12692E-37
Il1f9	1.8474	7.8063	171.8122	2.97417E-39	1.62323E-36
Ammecr1	1.7548	5.7906	170.9190	4.66064E-39	2.48162E-36
Slc26a9	2.7700	5.2294	168.6780	1.43852E-38	7.47723E-36
Il1f8	2.1833	6.5076	166.8497	3.6079E-38	1.83172E-35
Slc37a2	2.3621	7.1127	166.2590	4.85614E-38	2.40942E-35
Sprr2i	6.2566	5.7352	164.3026	1.29912E-37	6.30247E-35
Il1a	1.9904	6.3080	163.7304	1.73243E-37	8.22186E-35
Ankk1	6.6191	2.3953	163.2716	2.18213E-37	1.01358E-34
Gm5416	7.6192	3.6149	159.3098	1.6012E-36	7.28247E-34
Ptges	1.9700	7.5167	157.5843	3.81481E-36	1.69961E-33
BC100530	7.0442	9.7929	155.7815	9.44941E-36	4.1258E-33
Klk9	2.7708	7.9433	154.3417	1.95012E-35	8.34767E-33
Pinlyp	3.5299	5.1431	154.0588	2.2484E-35	9.43939E-33
Tgm5	2.2852	5.9994	153.9487	2.37652E-35	9.78903E-33
Padi1	4.2829	3.0512	152.9619	3.90484E-35	1.57864E-32
Ckap4	1.6283	6.7008	150.6287	1.26343E-34	5.01488E-32
Lce3c	4.5513	5.2115	150.5763	1.29716E-34	5.05682E-32

Psors1c2	1.9652	6.5775	150.3056	1.48646E-34	5.69313E-32
Car12	1.9988	7.2012	149.9628	1.76643E-34	6.64877E-32
Prss27	2.9447	5.9031	149.7026	2.0136E-34	7.45065E-32
Klk13	5.7758	7.1950	149.1228	2.69586E-34	9.8089E-32
Mab21l3	2.0274	4.0422	147.6564	5.63961E-34	2.01833E-31
Gm9774	5.1028	2.4088	146.8054	8.65513E-34	3.04758E-31
Slc34a2	2.2912	6.4256	146.2164	1.16423E-33	4.03433E-31
Ero1l	1.4172	5.9532	146.1765	1.18781E-33	4.05173E-31
Endou	2.8400	8.4750	145.2690	1.87565E-33	6.2996E-31
Plet1	1.8047	6.6140	144.8012	2.37367E-33	7.85144E-31
Krt16	6.5994	11.7948	144.4335	2.85641E-33	9.30722E-31
Gjb2	5.9404	8.7512	143.5056	4.55709E-33	1.46303E-30
Rbp2	3.3094	6.3650	143.0926	5.61014E-33	1.775E-30
BC117090	7.3289	2.8086	142.9497	6.02875E-33	1.8802E-30
Pde12	1.4047	6.3567	140.9050	1.68775E-32	5.18946E-30
Trim62	1.3397	5.5864	140.0092	2.64977E-32	8.03432E-30
Cers3	1.5684	6.9671	139.2751	3.83469E-32	1.14678E-29
Teddm3	2.1390	8.0080	138.7450	5.00796E-32	1.47742E-29
Krt84	4.6249	4.1070	138.6131	5.35188E-32	1.55782E-29
Prss22	2.0586	4.5903	137.3833	9.94191E-32	2.85581E-29
Tnc	3.4341	6.2747	133.0244	8.93068E-31	2.53202E-28
Gsdmc	4.2394	7.1631	131.0679	2.39281E-30	6.6971E-28
Cdhr1	6.0071	2.7155	130.3721	3.39739E-30	9.38842E-28
Nfe2l3	1.3409	5.4349	127.2308	1.65374E-29	4.51286E-27
Krt6a	6.3512	11.4772	126.8060	2.04852E-29	5.52113E-27
Aldh3b3	2.9951	3.8493	126.7040	2.15651E-29	5.74131E-27
Cnfn	1.9202	9.0961	125.8919	3.2469E-29	8.54014E-27
Gsdma	1.5381	7.9815	125.7907	3.41691E-29	8.88032E-27
Fchsd1	1.6489	5.4195	123.1688	1.28078E-28	3.28949E-26
Il1f5	1.9899	8.8280	122.8794	1.48188E-28	3.76174E-26
Shb	1.4140	5.0366	122.2354	2.05014E-28	5.14443E-26
Tprg	1.3057	5.9334	121.6473	2.75755E-28	6.84091E-26
Gjb6	5.2746	5.6779	120.9346	3.9494E-28	9.68757E-26
Sprr1b	5.7275	9.3239	120.7983	4.2302E-28	1.02611E-25
Aox4	3.7595	3.2613	119.7190	7.28885E-28	1.7486E-25
Degs2	4.6156	5.4798	119.5903	7.77719E-28	1.84548E-25
Glrx	2.5863	8.3851	119.3864	8.6191E-28	2.02113E-25
Fxyd4	3.4219	2.2976	119.3673	8.70261E-28	2.02113E-25
Slc7a11	3.3149	5.2285	119.3212	8.90715E-28	2.04686E-25
Tmem50b	1.2120	6.2356	118.3784	1.4327E-27	3.25806E-25
Sdr9c7	1.4910	7.6725	118.1087	1.64142E-27	3.69421E-25
Ttc22	2.1051	5.4250	117.8331	1.88607E-27	4.20151E-25
Degs1	1.5568	7.9627	117.2856	2.4857E-27	5.48134E-25
S100a9	5.7116	8.9635	115.6328	5.71982E-27	1.24869E-24
Ckmt1	1.4535	7.9281	115.1575	7.2689E-27	1.57116E-24
Vstm5	1.5704	5.0252	114.6264	9.50123E-27	2.03354E-24
Scnn1a	1.3319	6.5431	113.8322	1.41817E-26	3.00583E-24
Bcl3	2.2080	4.6758	113.0094	2.14753E-26	4.50796E-24
Olah	5.0722	1.1573	112.2295	3.18258E-26	6.61703E-24
Itgb8	2.0030	3.7911	112.0403	3.50115E-26	7.21072E-24
Oit1	8.5530	1.0351	111.8970	3.76363E-26	7.67886E-24
Trex2	2.3242	7.3805	111.6183	4.33175E-26	8.75615E-24
Krt90	5.0287	1.1214	110.0482	9.56373E-26	1.91547E-23
Flg	2.8971	11.7233	109.6842	1.14912E-25	2.28059E-23
Slco1a5	8.6746	1.1468	109.0431	1.58791E-25	3.12304E-23
Gsdmc2	4.3041	3.8407	108.9112	1.69723E-25	3.30823E-23
Cd44	1.3399	8.1761	108.8555	1.74561E-25	3.37242E-23
Il1b	3.5624	2.2863	107.8144	2.95171E-25	5.65253E-23

Gja1	1.7370	8.3874	107.7167	3.10074E-25	5.88628E-23
Klk7	1.5517	10.1160	107.4000	3.638E-25	6.84665E-23
Slc39a6	1.2262	6.8888	106.7822	4.96877E-25	9.27122E-23
Blmh	1.9857	9.2302	105.7033	8.5644E-25	1.58449E-22
Avil	1.4827	5.0310	105.1924	1.10837E-24	2.03334E-22
Serpinb3b	1.7308	7.7709	104.6637	1.44731E-24	2.63301E-22
Dsg1b	2.1419	7.8603	104.3821	1.66834E-24	3.01004E-22
Acp7	1.4500	6.5793	104.0495	1.97324E-24	3.53097E-22
Rab31	2.0334	6.4657	103.3341	2.83137E-24	5.02533E-22
Rrm2	1.5286	5.0897	102.9162	3.49628E-24	6.15543E-22
Psat1	1.4831	6.6505	102.6607	3.97761E-24	6.94681E-22
Gm5478	7.6467	4.4532	102.2308	4.94163E-24	8.49455E-22
Cpm	1.6858	7.2275	102.1366	5.18228E-24	8.83863E-22
Tubb3	6.2207	4.2429	102.1068	5.26064E-24	8.90272E-22
Uox	6.0515	3.9232	101.8095	6.11252E-24	1.02648E-21
Pnpla1	1.2872	6.8403	101.6890	6.49606E-24	1.08256E-21
Gm28042	2.5736	2.1799	101.3611	7.66545E-24	1.26776E-21
Stom	1.6673	6.7910	101.2991	7.90907E-24	1.29822E-21
Gpr35	2.1081	3.8022	101.2761	8.0017E-24	1.30362E-21
Gdpd1	1.3880	4.7694	99.1648	2.32343E-23	3.75725E-21
Cxcr2	1.8185	5.1855	98.9711	2.56213E-23	4.11279E-21
Psrc1	1.7779	3.5848	98.5325	3.19735E-23	5.09499E-21
Pglyrp1	3.1789	1.9928	97.7301	4.79489E-23	7.58531E-21
Bace2	1.4322	4.9166	97.2948	5.97373E-23	9.38219E-21
Bcl2l15	2.7779	2.3266	96.3614	9.57167E-23	1.49257E-20
Tgm3	3.0416	6.9961	96.0222	1.136E-22	1.75886E-20
Mocos	1.3384	4.9425	95.6316	1.3838E-22	2.12744E-20
Atp11b	1.2089	6.7292	95.5175	1.46588E-22	2.23788E-20
Top2a	1.6704	6.0626	95.4561	1.51204E-22	2.29232E-20
Klk8	2.1575	7.2433	95.1988	1.72197E-22	2.59258E-20
Myo5b	2.0355	5.7200	95.0714	1.83644E-22	2.74599E-20
Fabp5	3.9136	9.6989	94.6241	2.30203E-22	3.41876E-20
Cemip	5.2319	3.9470	93.6298	3.80407E-22	5.57361E-20
Smpdl3b	1.9282	3.6643	93.5452	3.97033E-22	5.77842E-20
Trim25	1.0713	6.7256	93.3139	4.4624E-22	6.45157E-20
Epn3	1.4060	7.2632	93.0523	5.09311E-22	7.31498E-20
Pcdh1	1.1230	7.8166	92.7518	5.92808E-22	8.45855E-20
Elovl7	1.6902	6.7954	92.7123	6.04772E-22	8.57323E-20
Capn12	1.7291	4.1370	92.5080	6.70524E-22	9.44401E-20
Tmem229b	1.7530	6.8430	92.4481	6.91127E-22	9.67179E-20
Defb6	1.6242	6.9146	92.3434	7.28697E-22	1.01326E-19
Klk11	1.4727	6.5110	92.2635	7.5869E-22	1.04829E-19
Pglyrp3	2.3145	4.4700	92.1368	8.08852E-22	1.11057E-19
Slc10a6	1.7457	5.0611	92.0403	8.49301E-22	1.15882E-19
Kif11	1.8767	4.2897	91.8809	9.2054E-22	1.24785E-19
Urah	2.0993	8.0557	91.8692	9.25988E-22	1.24785E-19
Dhrs9	1.3656	5.3894	91.8227	9.4802E-22	1.26971E-19
Ccl8	2.3226	6.1724	91.7296	9.93691E-22	1.32276E-19
Hbegf	2.5806	5.0804	91.6358	1.04191E-21	1.37854E-19
Slc6a20a	1.4073	5.4666	91.5989	1.0615E-21	1.39601E-19
Ttc39a	1.5638	4.7053	91.5189	1.10531E-21	1.44491E-19
Kctd11	1.4586	7.3132	91.1853	1.30826E-21	1.70004E-19
Il17c	6.9803	0.9633	91.1372	1.34046E-21	1.73158E-19
Bdh1	1.9082	4.9807	90.0170	2.36124E-21	3.03225E-19
Ivl	1.4554	8.6989	89.4519	3.1418E-21	4.01104E-19
Gm11992	1.1579	5.6374	88.8515	4.25589E-21	5.40177E-19
Shcbp1	2.2401	2.6488	88.5134	5.0491E-21	6.37149E-19
Ass1	2.4374	4.9826	88.4962	5.09329E-21	6.39033E-19

Zc3h12a	1.7661	6.9273	88.3997	5.34779E-21	6.67129E-19
Tgm1	1.3462	8.1273	88.1349	6.11396E-21	7.58374E-19
Sprr1a	2.9888	9.3969	88.1062	6.20315E-21	7.6509E-19
Rars	1.2029	6.8297	87.7923	7.27012E-21	8.91651E-19
Mcm3	1.2273	4.9110	87.7502	7.42638E-21	9.05729E-19
Sprr2j-ps	5.8748	2.8052	87.3075	9.28927E-21	1.12663E-18
Slc22a23	1.0646	7.1269	87.1583	1.00176E-20	1.20826E-18
Tmprss4	1.3189	7.8931	86.2160	1.61315E-20	1.93498E-18
Ppard	1.3223	6.6474	86.1550	1.66367E-20	1.98468E-18
Macc1	1.2561	5.2753	86.1118	1.70048E-20	2.01756E-18
Pla2g4f	1.2179	7.2001	85.8760	1.91578E-20	2.26073E-18
Smpd1	1.1646	7.6846	85.7501	2.04175E-20	2.39643E-18
Atp13a4	2.6130	4.1461	85.6680	2.1283E-20	2.48464E-18
Krt17	1.7679	10.6127	85.3001	2.5635E-20	2.97679E-18
Mxd1	1.2493	7.2205	84.6975	3.47688E-20	4.01607E-18
Stfa1	6.6080	7.1264	84.6351	3.58835E-20	4.12302E-18
Scnn1g	1.6692	3.9173	84.5048	3.83277E-20	4.3808E-18
Fetub	5.5438	4.5362	84.0270	4.8807E-20	5.54951E-18
Shf	1.6889	3.7282	83.5680	6.15602E-20	6.92743E-18
Bub1	1.8325	2.8719	82.4462	1.08584E-19	1.21564E-17
Pld2	1.0542	6.6158	82.4209	1.09982E-19	1.22501E-17
Plekhg1	1.3034	5.4856	82.1657	1.2514E-19	1.38677E-17
Cd24a	2.1823	7.8302	81.9210	1.41639E-19	1.56168E-17
Frrs1	1.2379	5.1313	81.8701	1.4533E-19	1.59432E-17
Tmem86a	1.3410	5.8517	81.6722	1.60636E-19	1.75342E-17
Has3	2.5288	5.7575	81.5770	1.68565E-19	1.83082E-17
Nkpd1	1.5047	6.8173	81.3703	1.87147E-19	2.02257E-17
Mcm5	1.6982	5.2067	81.3432	1.89734E-19	2.04043E-17
Fam43a	1.5368	5.9222	80.0338	3.6806E-19	3.91957E-17
Lypd3	1.8237	9.2485	79.3916	5.09435E-19	5.39877E-17
Arl5a	1.1675	6.8958	79.2229	5.54834E-19	5.85149E-17
Ptk6	1.1331	5.9161	79.1507	5.75476E-19	6.04001E-17
Uhrf1	1.4623	5.0791	79.0190	6.15151E-19	6.39493E-17
Casp14	2.7297	9.4427	78.9462	6.38235E-19	6.60346E-17
Fam83g	1.1423	8.1419	78.2120	9.25537E-19	9.53085E-17
Slc9a3r1	1.3898	6.3153	78.1030	9.78038E-19	1.00242E-16
Aadac	1.2450	4.5418	77.8192	1.12919E-18	1.15193E-16
Nipal1	1.4947	5.0944	77.7097	1.19353E-18	1.21191E-16
Maoa	1.1980	5.6407	77.6947	1.20266E-18	1.21553E-16
Aqp3	3.3587	8.7586	77.5701	1.28098E-18	1.28871E-16
Tfap2a	1.2112	5.7922	77.2099	1.53723E-18	1.53467E-16
Apol8	1.9855	2.8491	77.2069	1.53952E-18	1.53467E-16
Hip1r	1.1093	7.8245	77.0963	1.6282E-18	1.6157E-16
Atp1a1	1.4453	8.4333	76.9750	1.73132E-18	1.71025E-16
Galnt3	1.8294	2.8168	76.7942	1.89733E-18	1.8658E-16
Adgrf1	6.3507	1.0716	76.7748	1.91603E-18	1.87573E-16
Mcm2	1.2769	4.9182	76.5449	2.15259E-18	2.09791E-16
Mki67	1.6074	6.5056	76.4280	2.28387E-18	2.21596E-16
Krt1	2.2857	13.4438	75.8745	3.0228E-18	2.90708E-16
Proser2	1.4811	4.3284	75.7966	3.14439E-18	3.01075E-16
Ephx3	1.2174	6.9999	75.6801	3.33558E-18	3.17987E-16
Lig1	1.2187	4.5539	75.3295	3.98364E-18	3.7648E-16
Rab38	1.1192	5.6067	75.1733	4.31156E-18	4.05714E-16
Chaf1b	1.5225	2.9849	74.9055	4.93793E-18	4.62661E-16
Sult2b1	1.6258	6.9269	74.5782	5.82825E-18	5.43746E-16
Mdfi	1.8159	3.2242	74.0273	7.7046E-18	7.12708E-16
Dsc1	1.5903	9.6205	73.8083	8.60831E-18	7.89613E-16
Tmem125	1.2556	4.6751	73.7583	8.829E-18	8.06469E-16

Zfp185	1.0719	6.4168	73.3462	1.08793E-17	9.78318E-16
Nipal4	1.0070	7.5792	73.3443	1.08896E-17	9.78318E-16
Pla2g4d	6.5197	2.9406	73.0129	1.28807E-17	1.15245E-15
Saa1	3.3582	1.1265	72.6780	1.52627E-17	1.35448E-15
Slc2a1	1.5997	6.6296	72.5822	1.6022E-17	1.4161E-15
Tmigd1	1.8011	3.5546	72.5552	1.62422E-17	1.42977E-15
Gpr160	1.0148	5.1252	72.1243	2.02064E-17	1.77159E-15
Ceacam12	2.2085	2.5069	71.6243	2.60331E-17	2.27332E-15
Gsdmc3	4.5921	0.7518	71.2575	3.13523E-17	2.71608E-15
Igsf3	2.0815	5.4792	71.0285	3.52107E-17	3.03828E-15
Id1	1.5142	5.7516	70.6551	4.25458E-17	3.65676E-15
Aqp9	1.2000	5.3609	70.2247	5.29184E-17	4.53043E-15
Slc25a48	1.6887	5.4029	70.1598	5.46885E-17	4.66369E-15
Egln3	1.6643	7.7620	69.6860	6.95376E-17	5.90691E-15
Tmprss11g	2.3055	3.8909	69.6521	7.07444E-17	5.98613E-15
Gtf2f1	1.1495	6.0320	69.5128	7.59213E-17	6.39937E-15
Ide	1.2171	9.3184	68.8679	1.05284E-16	8.84018E-15
Sptlc1	1.1459	6.5833	68.5470	1.23887E-16	1.03623E-14
Abca12	1.0482	9.1946	68.2447	1.44415E-16	1.20333E-14
Otub2	1.6750	5.7659	68.0621	1.58426E-16	1.31506E-14
Pgam1	1.3339	8.1715	67.7424	1.86317E-16	1.54071E-14
Dennd2d	1.0057	4.9702	67.3307	2.29574E-16	1.87709E-14
Dsg3	1.4343	7.2305	66.8012	3.00308E-16	2.42815E-14
Lrrc4	3.1472	2.3752	66.7626	3.06257E-16	2.46712E-14
Tubb2a	1.5137	7.4723	66.5854	3.35055E-16	2.67934E-14
Vav3	1.2970	5.4833	66.0562	4.38256E-16	3.46651E-14
Tuba8	1.0917	7.2080	65.9428	4.64198E-16	3.65845E-14
Garem1	1.1122	4.9828	65.8541	4.85569E-16	3.81311E-14
Ppp4r1	1.0933	5.9624	65.8364	4.89938E-16	3.83363E-14
Ovol1	1.4435	7.3285	65.7250	5.18437E-16	4.04214E-14
B4galnt1	1.4712	4.2726	65.5046	5.7978E-16	4.50434E-14
Atp6v0a4	1.1734	5.9677	65.2111	6.72894E-16	5.2092E-14
Kif2c	1.9765	3.2740	64.9658	7.62085E-16	5.87883E-14
Cgref1	2.8369	3.3209	64.7897	8.33344E-16	6.40589E-14
Them5	1.4953	8.1267	64.5366	9.47567E-16	7.23298E-14
Smpdl3a	1.0531	7.4361	64.3300	1.05231E-15	8.00453E-14
Ada	1.8252	3.6150	63.8810	1.32168E-15	9.98394E-14
Asf1b	1.8739	3.2905	63.7540	1.40969E-15	1.0612E-13
Klk14	6.5770	3.4775	63.6461	1.48907E-15	1.11711E-13
Rps6ka4	1.2836	7.0124	63.2841	1.78946E-15	1.3333E-13
Pla2g4b	4.3729	6.0643	62.9750	2.09351E-15	1.54404E-13
Il19	5.0091	1.4765	62.8501	2.23056E-15	1.63342E-13
Slc25a13	1.3236	4.1406	62.8448	2.23654E-15	1.63342E-13
Rdh12	1.2269	5.9996	62.8443	2.23715E-15	1.63342E-13
Tacc3	1.5002	4.5875	62.0746	3.30693E-15	2.37479E-13
Tmem79	1.0249	6.1464	61.6779	4.04486E-15	2.89519E-13
Calm4	1.7649	11.5133	61.5944	4.22012E-15	3.01077E-13
Ddx39	1.6289	5.5608	61.3157	4.86193E-15	3.44613E-13
Mpp7	1.2180	5.9284	61.1946	5.17033E-15	3.64108E-13
Slc7a5	1.4731	5.1849	61.1765	5.21815E-15	3.66294E-13
Socs1	1.9099	2.1627	60.9163	5.95526E-15	4.16696E-13
Cstb	1.3698	7.0969	60.8664	6.10831E-15	4.2604E-13
Plpp6	1.2912	5.5960	60.6599	6.78367E-15	4.7014E-13
Mcm4	1.1453	5.4484	60.5049	7.33963E-15	5.07061E-13
Ak2	1.2138	6.9668	60.1640	8.72722E-15	6.01022E-13
Cbr3	1.2660	5.6332	60.1336	8.86329E-15	6.08473E-13
Dbf4	1.5738	3.5144	59.9606	9.67754E-15	6.6022E-13
Entpd7	1.4973	3.2913	59.9362	9.79814E-15	6.66365E-13
Ralgapa2	1.0359	5.6911	59.9272	9.8432E-15	6.6735E-13

Fhl2	3.8388	3.8892	59.7139	1.09699E-14	7.3915E-13
Slc25a5	1.0891	8.3820	59.4219	1.27243E-14	8.54717E-13
Dusp5	1.3225	5.2730	59.2957	1.35671E-14	9.05759E-13
Pmaip1	1.5770	3.7378	59.2897	1.36087E-14	9.05765E-13
Ddias	2.1224	2.3144	59.0625	1.52742E-14	1.01353E-12
Cep55	1.6479	3.0115	58.8196	1.72808E-14	1.1432E-12
Epha2	1.0551	6.4349	58.7767	1.7662E-14	1.16489E-12
Mapk6	2.0051	7.4720	58.6739	1.8609E-14	1.21998E-12
Cdc20	1.8257	4.9337	58.2019	2.36553E-14	1.54617E-12
Pclaf	2.1876	3.7459	58.0446	2.56238E-14	1.66983E-12
Mal2	1.4791	6.7613	58.0167	2.59908E-14	1.68802E-12
Map3k6	1.2049	5.3877	58.0116	2.60576E-14	1.68802E-12
Pfkfb4	1.5887	4.6333	57.8934	2.76708E-14	1.78195E-12
Sh2d5	3.5307	2.1693	57.7657	2.9527E-14	1.89589E-12
Phlda2	2.7504	1.2472	57.7436	2.98604E-14	1.91168E-12
Wfdc12	5.3656	4.1746	57.6930	3.0639E-14	1.95009E-12
Ctsd	1.1653	10.4903	57.5674	3.26592E-14	2.06065E-12
Lrrc28	1.1916	6.6408	57.4453	3.47518E-14	2.17794E-12
Skil	1.0840	5.1316	57.4415	3.48175E-14	2.17794E-12
Bub1b	1.6277	3.9188	57.4173	3.5249E-14	2.19863E-12
Igfl3	3.9162	1.1606	57.4034	3.54985E-14	2.20788E-12
Abcc3	1.3464	3.6966	57.3727	3.60571E-14	2.23626E-12
Prep	1.0236	6.5991	57.1259	4.08796E-14	2.52103E-12
Ndc80	1.7664	2.4172	57.1007	4.14061E-14	2.5463E-12
Slc26a10	1.4021	3.7878	57.0863	4.17104E-14	2.55781E-12
Slc23a4	2.9244	1.6217	57.0752	4.19455E-14	2.56502E-12
Cyp4f39	1.0290	6.4193	56.8931	4.60163E-14	2.80609E-12
Kntc1	1.4405	2.7008	56.8773	4.63879E-14	2.82088E-12
Cdh3	1.2594	5.9332	56.8616	4.67594E-14	2.83557E-12
Adm2	3.7753	0.5337	56.4596	5.73637E-14	3.44988E-12
Elf5	1.7463	3.3837	56.2526	6.37328E-14	3.78084E-12
Trim13	1.0725	4.1518	56.2086	6.51761E-14	3.85599E-12
Lipn	1.0308	5.5219	56.0747	6.97702E-14	4.11663E-12
Ap1m2	1.0489	6.0544	55.9961	7.26145E-14	4.26142E-12
Ncapg	2.0890	2.8485	55.8631	7.76956E-14	4.53522E-12
Kpna2	1.3478	6.2922	55.7204	8.35449E-14	4.85071E-12
Birc5	1.7744	3.7391	55.6718	8.5638E-14	4.95906E-12
Ugcg	1.0229	7.0822	55.5288	9.21004E-14	5.30513E-12
Dnah8	1.7731	3.3791	55.3991	9.83818E-14	5.65203E-12
Pi15	2.1912	4.2257	55.3817	9.92571E-14	5.68735E-12
Fam162a	1.7031	6.5443	55.1098	1.13981E-13	6.51393E-12
Phlda1	1.4982	4.5019	54.6437	1.44485E-13	8.21422E-12
Tmem165	1.1057	6.0662	54.4916	1.56114E-13	8.85227E-12
Plk1	2.0682	3.9194	54.4583	1.58779E-13	8.98006E-12
Serpinb6c	2.6172	4.5383	54.4353	1.60649E-13	9.04604E-12
Pfkl	1.0812	6.8991	54.4338	1.60774E-13	9.04604E-12
Acot7	1.1676	4.3355	54.2759	1.74225E-13	9.75259E-12
Pld1	1.2271	5.6442	54.1804	1.82903E-13	1.01861E-11
Fam84a	1.0981	7.0217	54.1197	1.88642E-13	1.0479E-11
Krt6b	8.3514	11.6163	54.0049	1.9999E-13	1.10531E-11
Fam83a	2.8335	2.6096	53.5054	2.57876E-13	1.42164E-11
Trpv3	1.0578	5.7668	53.2309	2.96562E-13	1.62669E-11
Postn	1.0843	9.3630	53.2087	2.99924E-13	1.6394E-11
Il1rn	1.0402	7.1656	53.2057	3.0038E-13	1.6394E-11
Cfap43	1.1380	5.8338	53.1964	3.01811E-13	1.6431E-11
Rbp1	2.9338	5.4010	53.0068	3.324E-13	1.7962E-11
Ncapd2	1.0336	4.7026	52.7925	3.70707E-13	1.99825E-11
Sqle	1.1336	7.5054	52.4322	4.45371E-13	2.37144E-11

Kif20a	1.2610	4.1101	52.3766	4.58162E-13	2.43361E-11
Hyal1	1.0988	4.8970	52.3044	4.75312E-13	2.50641E-11
Klk12	3.9147	2.6135	52.2532	4.8787E-13	2.56643E-11
Chaf1a	1.2170	3.7548	52.2370	4.919E-13	2.58141E-11
Atp2a2	1.0565	8.1378	52.1675	5.09617E-13	2.66797E-11
Smim3	2.1649	2.6314	51.6247	6.71901E-13	3.47589E-11
Tpx2	1.6538	4.4081	51.5669	6.91995E-13	3.57138E-11
Aaas	1.0409	4.3878	51.4488	7.34903E-13	3.76612E-11
Atp6v1a	1.0511	7.3334	51.1078	8.74317E-13	4.43889E-11
Eif6	1.0541	6.9248	51.0819	8.85927E-13	4.48739E-11
Lcn2	2.3838	4.4890	50.9778	9.34169E-13	4.69904E-11
Ckap2l	1.0585	4.5748	50.9706	9.37581E-13	4.70536E-11
Krt79	1.2027	9.1172	50.8655	9.89151E-13	4.92625E-11
Tdh	4.6553	5.1296	50.8626	9.90621E-13	4.92625E-11
Cdk5r1	1.2753	3.4975	50.7502	1.04902E-12	5.19302E-11
Rab25	1.1094	7.8969	50.6753	1.08981E-12	5.37059E-11
Cwh43	1.3850	5.5099	50.6419	1.10852E-12	5.45047E-11
Mybl2	2.1187	2.9848	50.4929	1.19595E-12	5.86129E-11
Tmem40	1.2224	6.5766	50.3634	1.27756E-12	6.19787E-11
Synpr	2.6328	0.6474	50.3511	1.28557E-12	6.22291E-11
Myd88	1.0088	5.8788	50.2833	1.33078E-12	6.42749E-11
Bdkrb2	1.6931	3.1077	50.0036	1.53461E-12	7.34694E-11
Tbc1d2	1.1447	4.7998	49.9254	1.597E-12	7.62891E-11
Car13	1.0293	6.9505	49.5048	1.97887E-12	9.3711E-11
Dnase1l2	1.1990	6.9936	49.4795	2.00453E-12	9.47204E-11
Myo1b	1.0616	5.4537	49.3248	2.16898E-12	1.0205E-10
Nod2	1.5859	5.3450	49.2614	2.24028E-12	1.05177E-10
Serinc2	1.2389	6.3645	49.0029	2.55586E-12	1.19224E-10
Mthfd1l	1.4121	2.7633	48.9539	2.6205E-12	1.21719E-10
Cdca8	1.5783	4.1187	48.8869	2.7115E-12	1.25679E-10
Cenpf	1.0941	4.3260	48.8009	2.83317E-12	1.3104E-10
Rgs20	1.6039	3.5806	48.6990	2.98417E-12	1.37442E-10
Ccl20	2.9626	3.7209	48.6931	2.99324E-12	1.37569E-10
Dlgap5	1.5890	2.9609	48.4289	3.42475E-12	1.56414E-10
Grhl3	1.1280	7.4587	48.2976	3.662E-12	1.669E-10
Ppif	1.2750	6.8881	48.0225	4.21354E-12	1.90766E-10
Slc5a10	1.8432	1.6967	47.6914	4.98866E-12	2.24089E-10
Calml3	1.6965	8.2037	47.6372	5.12853E-12	2.29899E-10
Abhd12b	1.4091	5.5165	47.6067	5.20902E-12	2.32552E-10
Clspn	1.8947	3.0841	47.3864	5.82854E-12	2.58099E-10
Ereg	1.3379	6.7932	47.2682	6.19095E-12	2.72489E-10
Bok	1.5601	5.7953	47.2582	6.22246E-12	2.73325E-10
Knl1	1.5450	2.8939	47.2355	6.29501E-12	2.75404E-10
Asprv1	1.3705	11.4379	47.2244	6.33079E-12	2.76415E-10
1810037I17Rik	1.0714	6.4561	47.1588	6.5463E-12	2.85254E-10
Wdhd1	1.4352	3.2574	47.0030	7.08788E-12	3.08238E-10
Akr1d1	5.4612	3.2394	46.9395	7.3212E-12	3.17121E-10
Tnfaip2	1.1198	5.7712	46.7992	7.86472E-12	3.38648E-10
Ofcc1	4.6510	-0.2052	46.7758	7.9592E-12	3.42042E-10
Cntn2	3.5342	-0.0046	46.7598	8.02421E-12	3.44158E-10
Krt14	2.2168	12.8732	46.7319	8.13915E-12	3.48404E-10
C330027C09Rik	1.6349	3.3134	46.7152	8.20889E-12	3.50701E-10
Prl2c3	8.6722	1.1431	46.6167	8.63229E-12	3.6807E-10
Rhof	1.6401	4.1188	46.4988	9.16758E-12	3.87863E-10
Dnmt3l	2.7957	1.0232	46.4780	9.26534E-12	3.91241E-10
Ccne1	2.2200	2.1668	46.2906	1.0195E-11	4.28839E-10
Ckap2	1.6080	3.8143	46.2296	1.05175E-11	4.40707E-10
Atp6v1b2	1.1971	7.3816	46.1990	1.06831E-11	4.45934E-10

Ltb4r1	1.0299	4.8528	46.1819	1.07766E-11	4.48121E-10
Snn	1.7475	4.7885	46.0480	1.15391E-11	4.78918E-10
Kif18b	1.5042	2.8391	45.7937	1.31388E-11	5.41394E-10
Higd1a	1.0913	6.6409	45.7387	1.35129E-11	5.55554E-10
Mafb	1.4317	7.2403	45.6228	1.43364E-11	5.86103E-10
Saa3	4.4372	3.7843	45.5288	1.5041E-11	6.1261E-10
Slc35f2	1.9367	3.4689	45.4619	1.55637E-11	6.32059E-10
Hrnr	1.0342	11.1255	45.4603	1.55764E-11	6.32059E-10
Kif4	1.0105	3.9248	45.4208	1.58938E-11	6.43742E-10
Cdca2	1.4960	2.6278	45.2480	1.73602E-11	7.01833E-10
Tex101	3.3034	1.0443	45.0510	1.91967E-11	7.71791E-10
Nccrp1	1.0650	8.8473	45.0174	1.95292E-11	7.83717E-10
Lpar5	1.2356	3.6823	44.9364	2.03534E-11	8.15294E-10
Incenp	1.3204	4.7248	44.7963	2.18636E-11	8.72587E-10
E2f8	1.3538	4.2688	44.7636	2.22313E-11	8.8564E-10
Tgfa	1.2250	6.3531	44.7384	2.25195E-11	8.95489E-10
1110008P14Rik	1.1105	5.2344	44.7186	2.27485E-11	9.02949E-10
BC016579	1.1950	4.2776	44.5363	2.49681E-11	9.85674E-10
Cst6	1.1780	9.5135	44.4841	2.56431E-11	1.00902E-09
Iffo2	1.0025	8.4188	44.4511	2.60788E-11	1.02397E-09
Hif1a	1.1372	6.8339	44.4371	2.62661E-11	1.02947E-09
Kif15	1.8034	2.8831	44.4051	2.66995E-11	1.04458E-09
Tk1	1.5104	3.5569	44.3023	2.81391E-11	1.09502E-09
Areg	2.8193	3.3689	44.2644	2.8689E-11	1.11443E-09
Aurkb	1.8106	3.4303	44.1707	3.00945E-11	1.16488E-09
Fam83b	1.0393	5.9795	43.8944	3.46572E-11	1.32971E-09
Gm5414	4.2587	0.3022	43.8703	3.50868E-11	1.34383E-09
Gpx1	1.2387	8.4354	43.8649	3.51846E-11	1.34521E-09
Sema4g	1.0831	4.3660	43.8197	3.60064E-11	1.37182E-09
Bnc1	1.1305	4.4821	43.7902	3.65526E-11	1.39021E-09
Fblim1	2.2123	4.0069	43.7114	3.80547E-11	1.43986E-09
Eno1	1.4013	8.2032	43.7018	3.82412E-11	1.44437E-09
Arrdc4	1.0253	6.2808	43.6933	3.84075E-11	1.44814E-09
Ralbp1	1.0891	8.0246	43.5297	4.17564E-11	1.56899E-09
Rbm47	1.0465	4.0402	43.2991	4.69793E-11	1.75918E-09
Asns	1.5081	5.1723	43.2330	4.85942E-11	1.81034E-09
Kyat1	1.2584	5.6991	42.9987	5.47749E-11	2.03366E-09
1810011O10Rik	1.3019	5.4528	42.9399	5.64488E-11	2.08517E-09
Hmmr	1.3274	3.9012	42.8432	5.93083E-11	2.1834E-09
Eno1b	1.4307	8.0539	42.8181	6.00741E-11	2.20417E-09
Lgals7	1.2211	11.1709	42.7072	6.35792E-11	2.32495E-09
Smc2	1.4743	4.1860	42.5621	6.84724E-11	2.49137E-09
Ace2	1.2047	6.4732	42.4722	7.16925E-11	2.59987E-09
Epgn	2.1470	5.0264	42.3589	7.59686E-11	2.72775E-09
H60c	1.0722	5.8913	41.9760	9.24024E-11	3.27473E-09
Slc39a8	1.2275	6.4167	41.6904	1.0693E-10	3.72907E-09
Nrg1	2.7430	1.4961	41.6644	1.08367E-10	3.76712E-09
Csta1	1.2826	7.5849	41.5587	1.14383E-10	3.96365E-09
Hells	1.1001	3.5874	41.4387	1.21626E-10	4.20128E-09
5430419D17Rik	1.4535	2.1917	41.4337	1.21934E-10	4.20529E-09
Tmprss11b	5.3614	2.2522	41.3155	1.29538E-10	4.45347E-09
Brca1	1.4671	2.4677	41.3069	1.30106E-10	4.46596E-09
Atad2	1.0077	4.3373	41.2836	1.31666E-10	4.51242E-09
Foxm1	1.1625	3.9866	41.1829	1.38632E-10	4.7437E-09
Sfxn1	1.0411	5.3458	41.1338	1.42157E-10	4.85671E-09
Ebp	1.1236	5.9245	41.1064	1.44161E-10	4.91748E-09
Ccnb1ip1	2.3210	0.9182	41.0824	1.45942E-10	4.97044E-09
Mpzl2	1.0973	6.6399	41.0601	1.47617E-10	5.01967E-09

Klk10	1.7152	6.8141	40.9355	1.57338E-10	5.32535E-09
Syt8	1.8894	1.8379	40.9142	1.59065E-10	5.36715E-09
Rnase2b	1.9833	4.3632	40.7370	1.7416E-10	5.83142E-09
Gtse1	1.5327	2.9127	40.6557	1.81555E-10	6.0491E-09
Tfrc	1.0716	6.0893	40.4226	2.04561E-10	6.75608E-09
Sfn	1.5534	10.1720	40.2497	2.23484E-10	7.32996E-09
S100a8	6.1874	7.9559	40.1674	2.331E-10	7.58393E-09
Phgdh	1.1878	5.8611	40.0987	2.4145E-10	7.83223E-09
Fam171a2	1.0127	3.7239	40.0288	2.50251E-10	8.09627E-09
Zdhhc13	1.0965	4.9328	40.0281	2.50331E-10	8.09627E-09
Slc39a2	2.3180	3.0627	40.0087	2.52831E-10	8.16504E-09
Prss12	1.4214	4.5442	39.8847	2.69407E-10	8.66189E-09
Slc16a6	1.0272	4.9672	39.8461	2.74784E-10	8.82177E-09
Cd200	1.0598	4.3456	39.5628	3.17679E-10	1.01393E-08
Cdt1	1.2045	3.6363	39.5155	3.25464E-10	1.03574E-08
Cdsn	1.1345	10.6353	39.5006	3.27953E-10	1.04215E-08
Rnpep	1.0556	5.6601	39.2953	3.64315E-10	1.14767E-08
Dusp14	1.4336	6.4101	39.2247	3.77734E-10	1.18652E-08
Knstrn	1.3043	3.6226	39.0871	4.05322E-10	1.26952E-08
Tuba1c	1.2232	7.5094	38.8835	4.49871E-10	1.39505E-08
Fignl1	1.6802	2.3309	38.8274	4.62972E-10	1.43364E-08
Shmt2	1.1930	6.3144	38.7303	4.86602E-10	1.50255E-08
Spc25	1.7645	2.2620	38.5380	5.36989E-10	1.64649E-08
Lelp1	7.0401	-0.3216	38.4399	5.64673E-10	1.72529E-08
Capn2	1.0074	7.2710	38.3779	5.82873E-10	1.77269E-08
Sprr2k	5.4088	1.4869	38.1721	6.47709E-10	1.95576E-08
Uchl3	1.0956	5.9159	38.1563	6.52973E-10	1.96893E-08
Rcc1	1.0501	4.9454	38.0886	6.76029E-10	2.02725E-08
Uck2	1.2047	6.6554	37.9771	7.15782E-10	2.13765E-08
E2f7	1.4330	2.9097	37.8998	7.44734E-10	2.21503E-08
Aldh3b2	1.1358	7.2310	37.8486	7.64547E-10	2.27086E-08
Gsto1	2.1903	6.4606	37.6794	8.33825E-10	2.45658E-08
Orc1	1.8903	2.2563	37.6270	8.56485E-10	2.51588E-08
Zfp92	1.2676	3.1173	37.2477	1.04039E-09	3.00035E-08
Slit1	2.1467	1.9413	37.1344	1.1026E-09	3.14504E-08
Spc24	1.6399	2.8651	37.1328	1.10352E-09	3.14504E-08
Ell2	1.2433	5.9003	37.0365	1.15936E-09	3.28276E-08
Ccnf	1.4205	3.8660	36.9652	1.20254E-09	3.40061E-08
Ptprz1	2.7399	4.8044	36.8601	1.26915E-09	3.57507E-08
Cpa4	1.1628	8.2397	36.7693	1.32965E-09	3.73106E-08
Fndc11	2.6521	0.7634	36.7594	1.33645E-09	3.74532E-08
Cenpe	1.5866	3.6795	36.6805	1.39161E-09	3.88992E-08
Nusap1	1.1486	3.7856	36.6481	1.415E-09	3.94016E-08
S100a16	1.0884	8.0056	36.4916	1.53322E-09	4.21559E-08
Cit	1.2663	2.9665	36.3460	1.65219E-09	4.53698E-08
Fgfbp1	1.2961	7.0880	36.2323	1.75144E-09	4.77351E-08
Zwilch	1.5740	2.5824	36.0927	1.88148E-09	5.10879E-08
Melk	1.6872	2.8794	35.8581	2.12221E-09	5.68465E-08
Ccnb1	1.6894	4.3317	35.8457	2.13579E-09	5.71403E-08
Cdk1	1.8634	4.0102	35.8398	2.14232E-09	5.72448E-08
S100a7a	3.7116	1.7974	35.7608	2.23087E-09	5.93929E-08
Twf1	1.2381	7.3197	35.7557	2.23674E-09	5.94765E-08
Ier3	1.0575	5.7790	35.6665	2.34158E-09	6.21885E-08
Chek1	2.0654	1.4045	35.6232	2.39417E-09	6.35079E-08
Prr11	1.5252	3.0179	35.5583	2.4753E-09	6.54137E-08
Hsd17b2	1.6216	2.8756	35.3666	2.73126E-09	7.14943E-08
Trim59	1.0834	3.8366	35.2452	2.90698E-09	7.59118E-08
Tchhl1	6.9916	-0.3619	35.2213	2.94284E-09	7.6665E-08

Exo1	1.7204	1.6336	34.9344	3.4101E-09	8.78936E-08
Nuf2	1.8931	2.9354	34.8217	3.61322E-09	9.26911E-08
Cdh16	2.0623	0.9241	34.6138	4.02048E-09	1.02297E-07
Pitpnm3	1.5507	3.5599	34.4954	4.27258E-09	1.07957E-07
Sgo1	1.4981	2.3589	34.2144	4.9363E-09	1.22879E-07
Ticrr	1.5325	2.4993	34.1630	5.06822E-09	1.25732E-07
Stac2	2.2347	3.3793	34.1504	5.10128E-09	1.26122E-07
Cdca5	2.3010	1.6086	34.1038	5.22492E-09	1.28819E-07
Cenpn	1.5227	1.5855	34.0096	5.48415E-09	1.34221E-07
Sec14l2	1.0151	5.9649	34.0041	5.49966E-09	1.34449E-07
Fscn1	1.8997	5.4882	33.9639	5.61441E-09	1.36879E-07
Orai2	1.0164	3.5597	33.8955	5.81517E-09	1.40744E-07
Serpinb3d	6.6524	-0.6279	33.8871	5.84033E-09	1.41196E-07
Polr3g	1.1493	6.0010	33.8606	5.92047E-09	1.42975E-07
Mad2l1	1.0328	3.0727	33.8446	5.96941E-09	1.43857E-07
Ska3	2.0441	1.5404	33.6843	6.48204E-09	1.55334E-07
4930427A07Rik	2.2518	0.7685	33.3855	7.55845E-09	1.77844E-07
Kif22	1.8126	3.2975	33.1951	8.33596E-09	1.93804E-07
Mis18bp1	1.4525	2.3891	33.1545	8.51204E-09	1.97478E-07
Jakmip2	2.4582	3.0208	33.1044	8.73414E-09	2.022E-07
Spag5	1.4009	3.2162	33.0392	9.03206E-09	2.08214E-07
Nek2	1.4516	3.1137	33.0282	9.08333E-09	2.09175E-07
Rad54l	2.7805	0.9002	32.9111	9.64715E-09	2.21007E-07
Rell2	1.2921	3.5957	32.9110	9.64771E-09	2.21007E-07
Mastl	1.5700	2.4900	32.8272	1.00727E-08	2.29777E-07
Ces2e	2.2935	0.8743	32.7739	1.03527E-08	2.35804E-07
Rps12	1.4262	4.3154	32.7269	1.06062E-08	2.4094E-07
Fam83c	1.0863	5.8046	32.7184	1.06522E-08	2.41735E-07
Atp12a	2.0698	7.5466	32.4229	1.24017E-08	2.79403E-07
Ccna2	1.1436	3.9716	32.4160	1.24456E-08	2.80102E-07
Wnt4	1.0881	6.6526	32.3107	1.3139E-08	2.94494E-07
Nt5dc2	2.0520	4.9532	32.0738	1.48425E-08	3.2963E-07
Aurka	1.6934	3.3407	31.8942	1.62802E-08	3.56482E-07
Cks1b	1.1536	3.9277	31.8576	1.65902E-08	3.62907E-07
Poc1a	1.4071	2.0085	31.8487	1.66657E-08	3.64193E-07
Igsf9b	1.5511	2.4872	31.5287	1.96514E-08	4.25182E-07
Lce1g	1.6749	7.0387	31.5247	1.96918E-08	4.25635E-07
Gsdmc4	3.0272	0.6860	31.4849	2.01001E-08	4.33602E-07
Cyp2b10	1.1468	5.9352	31.4082	2.09099E-08	4.49295E-07
Pttg1	1.5057	3.3161	31.3050	2.20516E-08	4.67388E-07
Idi1	1.2005	6.9652	31.2711	2.244E-08	4.74237E-07
Pglyrp4	1.4531	5.8626	31.2219	2.30156E-08	4.84525E-07
Ephb2	1.0298	4.3782	31.2200	2.30378E-08	4.84525E-07
H2afx	1.0116	4.3400	31.1948	2.33394E-08	4.90396E-07
Ncaph	1.2387	3.1966	31.1133	2.43401E-08	5.08973E-07
Ect2	1.2869	3.2924	31.0719	2.48648E-08	5.18952E-07
Ppa1	1.0266	5.8477	31.0691	2.49E-08	5.19189E-07
Fam46a	1.0111	6.5490	30.9941	2.5882E-08	5.36509E-07
2610528J11Rik	1.0723	3.9072	30.9621	2.63113E-08	5.43941E-07
Ccnb2	1.5644	3.7668	30.9083	2.70511E-08	5.5765E-07
Lss	1.0293	5.5624	30.7074	3.00017E-08	6.11548E-07
Gch1	1.1481	2.8522	30.4216	3.47631E-08	6.9753E-07
Gm20390	1.2091	6.4732	30.2400	3.81753E-08	7.56953E-07
Cxcl2	3.1990	0.1444	30.1792	3.9391E-08	7.78936E-07
Trip13	1.7304	1.5981	30.1075	4.08752E-08	8.03915E-07
Slc5a9	1.1105	4.6567	29.9087	4.52874E-08	8.796E-07
Hpx	2.7326	0.2540	29.8361	4.70162E-08	9.06723E-07
Tmem95	1.6076	1.5548	29.8110	4.76289E-08	9.15305E-07

Srms	1.2027	3.3517	29.7687	4.86799E-08	9.33859E-07
Haspin	1.5201	2.0180	29.7481	4.91996E-08	9.42172E-07
Upp1	2.6268	1.1718	29.6895	5.07094E-08	9.70234E-07
Guca2b	3.2971	-0.3445	29.5676	5.40002E-08	1.02422E-06
Enah	1.5921	6.4622	29.4974	5.59923E-08	1.05833E-06
Kcnh1	2.2244	1.0055	29.3872	5.92674E-08	1.11636E-06
Ulbp1	1.1579	2.5395	29.3233	6.12553E-08	1.15182E-06
Lrr1	2.3121	0.3836	29.0810	6.94143E-08	1.2864E-06
Mms22l	1.2219	2.6871	29.0718	6.97455E-08	1.29035E-06
Defb1	1.1583	5.2741	28.9487	7.43225E-08	1.37038E-06
Pdcd1	5.7552	-1.2382	28.8297	7.90297E-08	1.45092E-06
Spire2	1.3182	2.3957	28.7186	8.36966E-08	1.52647E-06
Sult4a1	3.1918	-0.2026	28.6711	8.57737E-08	1.55914E-06
Mgst2	1.9633	2.5435	28.6325	8.75008E-08	1.58657E-06
Racgap1	1.2442	4.6368	28.6214	8.80064E-08	1.59441E-06
Spdl1	1.1800	2.6903	28.1432	1.12665E-07	1.98675E-06
Kifc1	1.2667	2.4159	28.1135	1.14403E-07	2.01252E-06
Cdca3	1.6487	3.2333	27.9973	1.21482E-07	2.12676E-06
Fancd2	1.3175	1.9088	27.9668	1.23417E-07	2.15546E-06
Ces2g	1.7470	6.0902	27.9391	1.25197E-07	2.18131E-06
Cdk17	1.0448	4.1428	27.7668	1.36854E-07	2.37305E-06
Rad51	1.0878	2.9807	27.4757	1.59084E-07	2.71577E-06
Fam3b	1.0058	3.7212	27.3494	1.6982E-07	2.86724E-06
Stil	1.5760	2.0126	27.3165	1.72727E-07	2.91408E-06
Ttc34	2.9234	0.0194	27.3032	1.73922E-07	2.93196E-06
Trib3	1.1999	3.9059	27.2964	1.74538E-07	2.93566E-06
Tmprss11a	3.2458	1.6413	27.2578	1.78055E-07	2.99009E-06
Gstm6	1.7101	1.9292	27.1404	1.89199E-07	3.16505E-06
Ercc6l	1.5145	2.5672	27.0941	1.93786E-07	3.22601E-06
Spx	3.2705	0.4261	27.0845	1.94753E-07	3.23812E-06
Cd101	1.9674	1.3884	26.8906	2.15308E-07	3.55551E-06
Avpi1	1.4741	6.2712	26.8415	2.20846E-07	3.62775E-06
Parpbp	1.5364	1.7051	26.8175	2.23605E-07	3.66205E-06
Nppb	4.7751	-0.8612	26.5503	2.5677E-07	4.14304E-06
Pbk	1.7359	2.9285	26.5385	2.58337E-07	4.16525E-06
Hoxa1	1.2551	1.6040	26.4766	2.66755E-07	4.28201E-06
Prim2	1.0098	2.9293	26.4348	2.72583E-07	4.35953E-06
Fam46b	1.3839	5.7381	26.4302	2.73234E-07	4.36674E-06
Rfng	1.0335	5.2327	26.3573	2.83744E-07	4.50503E-06
Slc20a1	1.0272	4.8458	26.3490	2.84964E-07	4.52111E-06
Il24	4.9707	-0.2607	26.2933	2.93305E-07	4.63121E-06
Akr1b8	1.3051	4.5844	26.2552	2.99145E-07	4.70845E-06
Kcnv1	3.6297	-0.1065	26.0727	3.28799E-07	5.11254E-06
Fgf3	6.5353	-0.7302	26.0689	3.29455E-07	5.1191E-06
Ncapg2	1.4394	2.9712	26.0256	3.36913E-07	5.21641E-06
Fanca	1.3011	1.9240	25.9581	3.48903E-07	5.37537E-06
Sox15	1.4636	3.1928	25.9439	3.51486E-07	5.40372E-06
Fyb2	2.3738	0.1288	25.9019	3.59211E-07	5.51472E-06
Dyrk3	1.7488	0.7407	25.7193	3.94849E-07	6.01952E-06
Cmtm1	5.3603	-1.4723	25.3350	4.81897E-07	7.14694E-06
Abcg4	1.7035	3.7562	25.2890	4.93512E-07	7.30926E-06
Hp	1.9139	6.7609	25.2709	4.98168E-07	7.36323E-06
Rnase1	1.1976	3.6951	25.2383	5.06661E-07	7.46351E-06
Eme1	1.2765	1.6249	25.0786	5.50408E-07	8.04281E-06
Gjb1	2.5056	0.9746	24.8933	6.05915E-07	8.7717E-06
Slc2a9	1.1172	2.6887	24.8912	6.0659E-07	8.77565E-06
Fbxl22	1.3501	1.5657	24.8749	6.11748E-07	8.84441E-06
Dph2	1.8529	1.5596	24.7945	6.37785E-07	9.14214E-06

2210017I01Rik	1.4972	4.4870	24.7563	6.50549E-07	9.30067E-06
Kcnq2	2.9048	-0.2724	24.7210	6.6259E-07	9.44807E-06
Mcm10	2.0601	1.8802	24.5555	7.22006E-07	1.01954E-05
Rpl27	1.1616	4.4288	24.1929	8.71532E-07	1.19965E-05
Crabp2	1.0486	6.3929	24.1076	9.10995E-07	1.24611E-05
Gm38119	3.1581	0.7504	23.9910	9.67858E-07	1.31238E-05
Tmprss11d	3.1447	1.8800	23.9503	9.88554E-07	1.33629E-05
Olfr1388	1.7446	1.7686	23.8857	1.02226E-06	1.37622E-05
Recql4	1.2960	2.3205	23.8672	1.03217E-06	1.38666E-05
Tmem254a	1.6429	1.7849	23.8232	1.05603E-06	1.4135E-05
Fer1l4	4.1107	-1.0435	23.7724	1.08429E-06	1.44777E-05
Iqgap3	1.1763	3.3009	23.7031	1.12402E-06	1.49716E-05
U90926	4.8042	-0.8458	23.7014	1.125E-06	1.49755E-05
Lgals4	1.2908	5.8693	23.6296	1.16775E-06	1.54747E-05
Cd274	1.0071	3.8433	23.4793	1.26262E-06	1.65851E-05
2010005H15Rik	4.4693	-0.1407	23.4169	1.30425E-06	1.70702E-05
Ripk3	1.1256	3.5582	23.0897	1.54615E-06	1.98786E-05
Nme1	1.1310	4.1569	23.0819	1.55244E-06	1.99361E-05
Kcnc3	1.0710	3.1809	23.0309	1.59422E-06	2.03886E-05
Atp13a5	1.3164	3.3914	23.0063	1.61475E-06	2.0615E-05
Slc1a1	1.0024	4.1574	22.9509	1.66197E-06	2.1193E-05
Vsnl1	1.4947	1.5177	22.9194	1.68934E-06	2.15169E-05
Lce1f	1.0857	7.9610	22.8521	1.74955E-06	2.21932E-05
Esco2	1.5608	1.8064	22.7631	1.83248E-06	2.31778E-05
Dnah10	2.1458	0.6544	22.6982	1.89545E-06	2.38409E-05
Styk1	1.0446	2.1345	22.5364	2.06203E-06	2.56091E-05
Kifc5b	1.1493	2.4959	22.4969	2.10481E-06	2.60636E-05
Serpina9	3.4005	1.5202	22.4514	2.15534E-06	2.65987E-05
Dsc2	2.8403	4.2409	22.3415	2.28219E-06	2.80532E-05
Syt12	1.0163	2.9988	22.3223	2.30513E-06	2.83193E-05
Ccl3	2.6736	-0.2268	22.1703	2.49501E-06	3.03954E-05
Sprr3	3.5009	-0.6163	21.9385	2.8153E-06	3.39188E-05
Blm	1.2577	2.0022	21.8829	2.89811E-06	3.48013E-05
Ush2a	2.7302	-0.5606	21.8563	2.93858E-06	3.51326E-05
Cenph	1.1749	1.8617	21.7329	3.1337E-06	3.7241E-05
Cmtm2a	5.4444	-1.4354	21.4161	3.69654E-06	4.29023E-05
Rps6kb2	1.1785	5.3956	21.4024	3.723E-06	4.31635E-05
Aldh1l2	2.2688	2.4172	21.2283	4.07711E-06	4.67475E-05
Sgo2a	1.5354	1.5644	21.2025	4.13214E-06	4.7248E-05
Etv4	1.2515	3.4609	21.0416	4.49418E-06	5.08882E-05
Gm5615	1.0921	2.1612	21.0392	4.49981E-06	5.09256E-05
Serpinb12	1.8959	6.4183	21.0310	4.51905E-06	5.10904E-05
Apol9b	1.5825	2.2982	20.9423	4.73338E-06	5.31829E-05
Tspan3	1.1389	6.5836	20.9322	4.75827E-06	5.34351E-05
Lrp8	2.1301	1.7976	20.8121	5.0661E-06	5.65429E-05
Rbbp8nl	1.1072	2.8317	20.7911	5.12201E-06	5.70503E-05
Tmem51	1.1566	3.8323	20.7680	5.18413E-06	5.76834E-05
Glt1d1	2.3843	0.5255	20.7138	5.33308E-06	5.92501E-05
Slc18a1	3.6570	-0.7733	20.6910	5.39677E-06	5.97146E-05
Dhcr24	1.0987	8.8177	20.6742	5.44444E-06	6.01201E-05
Cenpm	1.7175	1.3464	20.6586	5.48914E-06	6.04914E-05
Clec4n	1.3409	2.1159	20.5733	5.73905E-06	6.29911E-05
Sowahb	1.0588	2.4936	20.5346	5.85626E-06	6.40521E-05
Cpn1	3.3490	-0.1680	20.4589	6.09271E-06	6.6207E-05
Tmem139	1.1189	1.9662	20.4487	6.12519E-06	6.64277E-05
Lipg	1.2444	3.6852	20.4200	6.21777E-06	6.73314E-05
Wdr62	1.5342	3.6727	20.2337	6.85337E-06	7.34492E-05
Gp1bb	1.3683	3.3888	20.0792	7.43018E-06	7.89335E-05

Gml	4.2999	-1.1830	19.9849	7.8056E-06	8.22714E-05
Mgarp	2.1276	2.5279	19.8438	8.40351E-06	8.79047E-05
Fam83d	1.1257	2.9362	19.8348	8.44332E-06	8.82631E-05
Oas1a	1.2244	4.2879	19.5044	1.00366E-05	0.00010282
Selp	1.1780	2.8206	19.4511	1.0321E-05	0.000105387
Olfr509	2.0595	-0.0103	19.3040	1.1147E-05	0.000112298
Adamts18	5.8879	-1.1733	19.2952	1.11989E-05	0.000112769
Traip	1.2848	2.2777	19.1920	1.18208E-05	0.000118105
Alox8	3.4936	5.4854	19.1553	1.20501E-05	0.000119931
Aldh1l1	1.0690	3.8544	19.1549	1.20529E-05	0.000119931
Bcl2a1b	1.4711	0.7910	19.0339	1.28415E-05	0.000126623
Grip1	1.7812	2.0143	18.9130	1.36816E-05	0.000133879
Chtf18	1.3014	1.9291	18.8763	1.39476E-05	0.000136298
Prg4	1.8568	3.2964	18.8571	1.40886E-05	0.000137307
Rnf152	1.2119	3.1887	18.8550	1.41039E-05	0.000137395
Tmem92	1.9029	0.2104	18.8224	1.43475E-05	0.000139519
Serpinb1a	1.0628	5.4930	18.8193	1.43706E-05	0.000139681
Timeless	1.2568	2.4287	18.7170	1.51627E-05	0.000146209
Ska1	1.7130	1.5671	18.3861	1.8037E-05	0.00016958
Ippk	1.3950	3.8764	18.3565	1.83193E-05	0.000171865
Myl7	3.8499	-1.4393	18.2806	1.90641E-05	0.000177706
Gzmb	2.7565	-0.3123	18.1893	1.99998E-05	0.000185321
Il17f	3.9988	-0.8860	18.1756	2.01444E-05	0.000186186
Celf5	1.5268	0.5038	18.1731	2.01703E-05	0.000186331
Bfsp1	1.7304	2.0439	18.1725	2.01771E-05	0.000186331
Gm12248	1.2909	1.9697	18.0834	2.11434E-05	0.000193861
9130230L23Rik	2.1695	-0.4308	17.9126	2.31287E-05	0.000209686
Lrat	2.1370	2.1495	17.9049	2.32219E-05	0.000210355
Prr9	2.2830	1.5941	17.7584	2.50803E-05	0.000224409
BC030867	2.5961	0.9891	17.6401	2.66895E-05	0.000237046
Fabp12	4.9013	-1.7090	17.6177	2.70058E-05	0.00023966
Trim58	3.9388	-1.3919	17.6088	2.71327E-05	0.000240689
Col6a5	1.7107	2.4718	17.5833	2.74995E-05	0.000243573
Il4ra	1.0719	4.9457	17.4787	2.90543E-05	0.000255348
Otud7a	2.2033	-0.6290	17.4116	3.00986E-05	0.000263782
Gm340	1.0752	1.6659	17.4019	3.02518E-05	0.000264806
Usp3	1.0320	4.7125	17.2150	3.33796E-05	0.00028746
Zbtb7c	1.0118	4.4387	17.1478	3.45807E-05	0.000296284
Stx11	1.1965	3.2755	17.0903	3.56436E-05	0.000304316
Gsta1	1.5662	2.5147	16.9892	3.75931E-05	0.000318469
Oas1g	2.1169	1.8433	16.9667	3.80412E-05	0.000321891
Fosl1	1.7487	3.5455	16.9558	3.82608E-05	0.000323498
Sim2	2.5622	-0.8280	16.9436	3.85074E-05	0.000324828
Mos	4.6890	-0.9644	16.9117	3.9159E-05	0.000329053
Ahsg	2.6057	-0.5049	16.8555	4.03364E-05	0.000338166
Dscc1	1.5725	0.6555	16.8087	4.13444E-05	0.000345555
Epop	1.3228	2.8086	16.7955	4.16319E-05	0.000347426
Spink4	1.8346	1.0506	16.7918	4.17134E-05	0.000347973
Gmnn	1.0863	3.0930	16.5462	4.74782E-05	0.000392315
Mmp13	3.4614	2.0787	16.5325	4.7824E-05	0.000394873
Cenpa	1.4390	2.3311	16.4120	5.09611E-05	0.00041746
2300002M23Rik	7.1754	-0.2362	16.3604	5.23691E-05	0.000428031
Olfr656	2.5109	-1.0245	16.3268	5.33034E-05	0.000433907
Sptbn4	1.5115	1.2997	16.2768	5.47289E-05	0.000442842
Mfsd2a	1.0481	4.2638	16.2281	5.61557E-05	0.000452374
Samd12	5.6185	-1.3291	16.2030	5.69037E-05	0.000457556
Il23a	2.7884	-0.7654	16.1821	5.75357E-05	0.000462127
Krt82	4.2519	-1.2351	16.1561	5.83319E-05	0.000467319

Rad18	1.2631	2.3462	15.9278	6.58043E-05	0.000519
Ankrd37	1.2014	2.1731	15.9096	6.64424E-05	0.000523459
Hist1h2ah	4.6731	-1.8112	15.8847	6.73204E-05	0.000529611
Ugt1a7c	1.1004	3.8527	15.8001	7.04005E-05	0.000550076
Entpd3	1.5224	0.8626	15.7679	7.16084E-05	0.00055821
Fhad1	3.5852	-0.0153	15.7228	7.3333E-05	0.000569727
Cyp4a12a	4.9128	-1.7079	15.7089	7.3874E-05	0.000572504
Mlkl	1.1520	2.1882	15.6282	7.70943E-05	0.000594926
Mansc1	1.0704	3.7527	15.5208	8.16004E-05	0.000625059
Depdc1b	1.6051	1.2893	15.4745	8.36265E-05	0.000637448
Gzmc	1.2348	2.1161	15.3556	8.90566E-05	0.000672694
Il13ra2	1.3962	0.7920	15.2817	9.26115E-05	0.000696692
Elfn1	3.0152	-1.1669	15.1522	9.91831E-05	0.000741022
Gm5724	5.2976	-1.5175	15.0421	0.000105139	0.000777414
Cdkn3	1.3811	1.4732	15.0148	0.000106669	0.000786186
Slfn4	1.8612	1.7492	14.8362	0.000117261	0.000854445
Nell1	1.7888	0.5749	14.8299	0.000117655	0.000856176
Il12b	2.7625	-0.3888	14.8093	0.00011895	0.000863581
Dppa2	5.0217	-1.6579	14.8014	0.000119445	0.00086575
Mtfr2	1.1601	1.0706	14.7225	0.000124548	0.00089618
Rdh16	1.0375	1.6544	14.7165	0.00012495	0.000897822
Cd59b	1.3174	1.8853	14.4519	0.000143786	0.001011601
Pitx1	2.1153	2.6594	14.4204	0.000146207	0.001025902
Rad51ap1	1.1516	1.3021	14.3863	0.00014888	0.001041732
Troap	1.1180	1.8959	14.3078	0.00015522	0.001078486
Cotl1	1.0075	5.9792	14.2139	0.000163161	0.001126846
Nmu	5.4110	-0.4641	14.1808	0.000166058	0.001144685
Tnfrsf9	1.5848	1.1596	14.1742	0.000166644	0.001147633
Adamts4	2.1901	0.8075	14.1579	0.00016809	0.00115577
Serpina3n	1.0918	5.7023	14.1519	0.000168625	0.001159088
Podnl1	3.5023	-1.6431	14.1194	0.000171564	0.001175952
Kif24	1.0260	1.7313	14.0648	0.000176619	0.001204555
B3gnt7	1.3975	0.5336	14.0545	0.000177586	0.001209985
Defb14	1.1028	3.4552	14.0438	0.000178598	0.001215772
Igf2bp2	2.4942	2.2664	13.9963	0.000183169	0.001240698
Fgfbp3	1.0403	1.8173	13.9130	0.000191472	0.001289733
Ttll2	1.2420	1.3651	13.9065	0.000192129	0.001293363
Ceacam16	2.2993	-0.9801	13.8255	0.000200591	0.001342874
Fanci	1.1557	2.7884	13.7436	0.000209533	0.001394185
Zfp541	2.8842	-1.2414	13.7134	0.000212929	0.001412906
Sorcs2	1.3546	3.2130	13.3652	0.000256338	0.001666503
Cenpp	1.2788	0.7689	13.2945	0.000266183	0.001723322
Gm4491	4.3880	-1.9271	13.2868	0.000267276	0.001729374
Carmil2	1.1378	0.5980	13.2150	0.00027772	0.00178847
Kif14	1.7036	2.3486	13.1766	0.000283461	0.001818997
Dthd1	4.3846	-1.9280	13.1023	0.000294934	0.001882148
Rgs3	1.0895	3.2454	13.0847	0.00029771	0.001895946
Ngf	1.0897	2.0405	13.0713	0.000299857	0.001907954
Rp1l1	4.3903	-1.9268	13.0559	0.000302336	0.001921483
Gins1	1.0986	2.2337	12.9758	0.000315535	0.001992031
Jmjd4	1.0707	2.6658	12.8463	0.000338144	0.00211156
Adam12	1.5906	2.4777	12.8120	0.000344406	0.002145733
Depdc1a	1.4577	1.6950	12.7740	0.000351479	0.002185459
Rdh9	1.1924	1.6681	12.7402	0.000357875	0.002218679
Tpcn2	1.3462	2.4554	12.5389	0.000398564	0.002433179
Grp	2.9773	-1.4801	12.5261	0.000401309	0.002447883
Scd2	1.1712	7.0429	12.4872	0.000409741	0.002496528
Chil4	4.4044	-1.9250	12.4442	0.0004193	0.002549788

Olfr11	2.8322	-1.3838	12.4059	0.000427984	0.002593202
Il33	1.2061	6.4279	12.3782	0.000434381	0.002627592
Mxd3	1.0692	1.4077	12.2875	0.000456014	0.002742488
Nsl1	1.0191	1.5106	12.1814	0.000482676	0.002881406
Slc4a9	1.0756	0.8410	12.1535	0.000489957	0.002919282
Amn	2.2829	-0.9409	12.1193	0.000499033	0.002964457
Arg1	1.4460	5.6706	12.0768	0.000510535	0.00301911
Cyp2s1	1.4340	4.4036	12.0255	0.000524774	0.003094635
Il17a	3.4821	-1.6175	11.9243	0.000554068	0.003241117
Sele	1.3274	2.0069	11.8826	0.000566609	0.003307388
Prss51	2.2207	-1.1721	11.8344	0.00058145	0.003385871
Kif26b	1.1983	3.4568	11.8143	0.000587791	0.003414599
Gsdma3	1.0543	2.0700	11.7986	0.000592758	0.003437052
Oip5	1.5576	-0.2711	11.7863	0.000596676	0.003457811
Olfr1316	1.9866	-0.0037	11.7370	0.000612697	0.003536695
Gm3336	2.1954	-1.0132	11.6816	0.000631206	0.003632016
Pax8	3.2803	-1.7078	11.6789	0.000632131	0.003635037
Lif	1.2085	1.9576	11.6774	0.000632634	0.003636396
Cdc25c	1.1301	2.1236	11.6622	0.000637843	0.003660556
Cdc6	1.2395	1.9860	11.5254	0.000686507	0.003906969
Erfe	1.1682	1.1408	11.4403	0.000718666	0.004063506
Hist1h2ag	4.5991	-1.8507	11.4337	0.000721253	0.004074969
Prl2c2	5.6732	-1.3256	11.4046	0.000732633	0.004130711
Isg15	1.1341	3.6619	11.3917	0.000737712	0.004153982
Alg9	1.0295	3.3400	11.2854	0.00078117	0.004359338
Spink12	4.6842	-1.8131	11.2844	0.000781591	0.004360572
Clcf1	1.5349	2.4070	11.2759	0.000785189	0.004378409
Slc26a4	3.7919	-0.2295	11.2000	0.000817957	0.00453334
Dynlt1a	1.7375	1.1745	11.1994	0.000818234	0.004533725
Slc29a4	2.0547	-0.8336	11.1097	0.000858777	0.004728364
Kcnmb4	1.5176	0.6503	10.9985	0.000911869	0.00498423
Mroh3	5.7406	-1.2419	10.9504	0.000935854	0.005105106
B4galnt3	1.8076	1.9624	10.9333	0.000944505	0.005142015
Trank1	4.2721	-1.9758	10.9301	0.000946166	0.005149778
Isx	3.9073	-1.4299	10.8983	0.000962548	0.005231112
Fbxo5	1.0410	2.5969	10.8409	0.000992847	0.005371709
Havcr1	2.4549	-1.2512	10.7998	0.00101511	0.005477227
Mboat4	1.0617	0.4661	10.7763	0.001028102	0.005531206
Clca1	1.2765	3.6705	10.7198	0.001059949	0.005675678
Antxrl	1.6274	-0.4966	10.7192	0.001060309	0.005676216
Tmprss11e	1.7667	2.6882	10.6807	0.001082587	0.005777063
Rps27	2.1725	1.2724	10.6600	0.001094801	0.005827993
Rundc3a	1.9910	2.1602	10.5940	0.001134577	0.006003141
Dctd	1.0664	1.7398	10.5345	0.001171661	0.006169448
Adgrg7	2.7843	-1.5739	10.4065	0.001255706	0.006540994
Pgbd1	1.1126	1.1719	10.3573	0.001289648	0.006687483
Aunip	1.9372	-0.4070	10.3443	0.001298771	0.0067284
Trem1	2.4445	-1.2476	10.3394	0.001302213	0.006741432
Stfa2l1	4.4154	-1.9215	10.2592	0.001360016	0.006997527
B3galt5	2.1675	-0.3939	10.1849	0.001415945	0.007246387
Hist1h2ae	2.2618	-1.0070	10.1762	0.00142268	0.007270255
Nup210l	1.2922	-0.1928	10.1600	0.001435185	0.007322159
Aqp5	2.8199	2.0705	10.1438	0.001447836	0.007374641
Cthrc1	1.2864	2.1536	10.1393	0.00145142	0.007389448
Snap25	6.0086	-1.0524	10.1108	0.001474064	0.007487274
Dgkq	1.0537	3.2805	10.0736	0.001504051	0.00763071
Mobp	3.1845	-1.7389	9.9314	0.001624794	0.008146731
Mip	4.0514	-2.0486	9.9240	0.001631382	0.008172256

Krt4	1.1533	1.4258	9.8847	0.001666579	0.008316136
2310034C09Rik	4.2448	-1.9858	9.8706	0.001679376	0.008370422
Ptgdr2	1.6042	-0.5153	9.7494	0.001793834	0.008849987
Mei1	4.9239	-1.7078	9.6504	0.001893115	0.009247841
Pif1	1.5805	1.3120	9.6472	0.001896428	0.009261949
Igf2bp1	2.5891	-0.9682	9.6392	0.001904688	0.009293974
4930578G10Rik	4.0542	-2.0486	9.6072	0.001938207	0.009430132
Gnat1	4.9835	-1.6468	9.6028	0.001942856	0.009450642
Mmp8	2.4419	-1.0382	9.5209	0.002031426	0.009828914
Hist1h2ap	4.8884	-1.7450	9.5021	0.002052397	0.009916424
Fbxo48	1.5962	-0.5161	9.4914	0.002064371	0.009968432
Cplx3	4.0244	-2.0521	9.4853	0.00207121	0.009992615
Ina	2.7179	-1.3140	9.4117	0.002156052	0.010350911
4930507D05Rik	1.6880	-0.9215	9.3954	0.002175264	0.010423292
Sell	1.0860	0.8355	9.3681	0.002207977	0.010559092
Dna2	2.1585	1.4723	9.3637	0.002213246	0.010577358
Shisa6	2.5029	-1.4368	9.3562	0.002222287	0.010613598
Ntrk1	2.3747	0.7080	9.3514	0.002228158	0.010636981
Kcnip2	1.0960	1.1916	9.3498	0.002230086	0.010643856
Kcnd2	2.1262	-1.0695	9.2285	0.002382807	0.011254665
Steap1	1.4539	-0.5197	9.1842	0.002441082	0.011505021
Clca3b	3.2201	-1.0380	9.1718	0.002457682	0.011570769
Spink10	3.9448	-2.0837	9.1632	0.002469341	0.01161564
Oas3	1.2168	3.1005	9.1038	0.002550845	0.011944978
Pglyrp2	1.2952	0.8118	9.0804	0.002583705	0.012080718
Atp7b	1.9384	-1.1771	9.0147	0.002678135	0.012466175
Cxcl3	2.6353	-1.3630	8.9473	0.002778747	0.012860574
Ptpn5	1.3218	1.4282	8.9034	0.002846407	0.013104156
Gpr39	1.6903	-0.4894	8.8751	0.002890919	0.013269819
Arhgef39	1.1742	0.6955	8.8744	0.00289196	0.013269819
Trim6	1.2096	0.5986	8.8381	0.002950022	0.013504284
Cd6	3.4153	-1.5646	8.8189	0.002981252	0.0136073
Uba7	4.5929	-0.0271	8.6518	0.003267422	0.014716543
Artn	1.5698	-0.0560	8.6488	0.003272746	0.014731407
Cd5	1.6815	-0.3905	8.6222	0.0033209	0.014914205
1700016H13Rik	3.3167	-1.3164	8.6171	0.003330171	0.014946742
Klrb1c	1.9928	-1.3346	8.6113	0.003340771	0.014981999
4933405L10Rik	1.7365	-0.9081	8.5865	0.003386673	0.015153608
Muc5b	4.0754	-2.0474	8.5717	0.003414276	0.015247217
Il22	3.8216	-2.1168	8.5660	0.003424963	0.015274844
Clrn3	3.0381	-1.8155	8.5594	0.003437344	0.015308171
Adamts13	1.3673	0.4739	8.3522	0.003852284	0.016887393
Tmem132d	1.8200	-0.3034	8.3435	0.003870751	0.016954728
Zfp365	1.5352	2.3587	8.2350	0.004108993	0.017844326
Crnn	2.1141	0.3571	8.1999	0.004189168	0.018124003
Rem2	1.4318	0.4156	8.1423	0.004324487	0.018631908
Gm5483	3.8773	-2.1049	8.1418	0.004325556	0.018632838
Pacsin1	2.1489	-1.3978	8.0755	0.004486733	0.01918329
Cd4	1.3552	1.3713	8.0518	0.00454594	0.01940223
Ldhal6b	1.8502	-1.2129	8.0327	0.004594138	0.019565863
Hspb9	1.9625	-1.1305	8.0158	0.004637005	0.01971382
Timp1	1.0848	2.0627	7.9756	0.00474127	0.020094478
Ces2c	2.8925	-1.5102	7.8897	0.004971835	0.020901238
Kbtbd6	1.1300	-0.1096	7.8730	0.005017748	0.021053713
Gm10110	3.9039	-2.0900	7.8096	0.00519683	0.021692543
Saa2	2.4344	-1.7119	7.6475	0.005685011	0.023408048
Cecr6	3.8461	-2.1141	7.6456	0.005691159	0.023428945
Spink7	4.5904	-1.8665	7.6125	0.005796623	0.023764523
Cyp19a1	3.6364	-2.1562	7.6025	0.005828758	0.023882812

Gpr3	1.3414	-0.1203	7.5726	0.005926348	0.024219037
Creb3l3	2.0665	0.6107	7.5108	0.006132902	0.024927832
Lactbl1	3.5903	-2.1832	7.4999	0.006170083	0.025060293
Gm7361	3.8255	-2.1149	7.4144	0.006470279	0.026066187
Gm19345	2.4809	-0.7304	7.4115	0.006480847	0.026094314
Hist1h3h	1.7073	-1.0883	7.4074	0.00649558	0.026139172
Ttpa	1.2747	1.7709	7.3948	0.006541323	0.026308701
Olfr734	3.8570	-2.1144	7.3712	0.006627653	0.026553184
Gls2	1.4802	0.6588	7.3683	0.006638275	0.02658598
Gbx2	3.5871	-2.1837	7.3487	0.006711102	0.026833345
Csf3	3.5940	-2.1826	7.3474	0.006716019	0.026833346
Pnma5	2.4610	-1.7160	7.3294	0.006783526	0.027078287
Spesp1	4.2740	-1.9847	7.2678	0.007019996	0.027904867
Tekt5	1.2886	-0.6083	7.2531	0.007077683	0.028093256
Fpr1	2.4453	-1.7145	7.2525	0.007080126	0.028097842
Gm5934	3.5915	-2.1818	7.2314	0.007164102	0.028374368
Rph3a	2.4840	-1.7068	7.2258	0.007186287	0.028441595
Muc13	2.8726	-0.7438	7.2120	0.007241608	0.028607825
Oxt	2.7885	-1.9124	7.1983	0.007297155	0.028765732
Ninj2	3.5768	-2.1849	7.1878	0.007339925	0.028908156
Slc10a4	3.5830	-2.1841	7.1841	0.007355429	0.02896399
Rims2	1.5530	-0.9760	7.1791	0.007375663	0.029017421
A930017K11Rik	3.5929	-2.1828	7.1788	0.007376973	0.029017421
Bhlha15	1.8314	-1.2091	7.1274	0.007591549	0.029764924
Ccl25	1.0483	0.2640	7.0699	0.007838904	0.030620394
Gsta4	1.5856	4.1296	7.0650	0.007860609	0.030676611
Ppp1r14d	2.4578	-1.7124	7.0268	0.008029856	0.031253303
Rnf183	2.1340	-1.4011	7.0013	0.0081449	0.03162777
Pcdha8	3.6791	-2.1545	6.9505	0.008379518	0.032360385
Csf2	2.2825	-1.5259	6.9068	0.00858689	0.033015215
Col2a1	2.4057	-1.2508	6.8627	0.008801491	0.033733381
Ephx4	1.0405	0.7280	6.8607	0.008811394	0.033765411
Lama1	1.0958	2.8173	6.8508	0.008860371	0.033916521
Ndp	1.6540	-0.4941	6.8401	0.008913573	0.034062873
Vpreb3	3.7882	-2.1179	6.7988	0.009121662	0.03469251
Ddx11	1.0503	2.1123	6.7542	0.009352479	0.035373177
Gprc5a	1.0899	0.4002	6.7450	0.009401203	0.035501915
Gm867	2.7548	-1.9236	6.7421	0.009416182	0.035530368
Cfap54	1.9547	-0.4640	6.6687	0.009812319	0.036781034
Gm21762	3.4774	-2.2140	6.6258	0.010051182	0.037534614
Gsg1	1.9423	-1.4873	6.5508	0.010483899	0.038864663
Gucy2c	1.6300	-1.1762	6.5368	0.010566481	0.039097772
Olfr1382	2.3128	-1.7627	6.5299	0.010607654	0.039203605
2810459M11Rik	2.0841	-1.6206	6.4738	0.010947662	0.040274468
Gzma	1.6671	-1.0513	6.4692	0.010975689	0.040365611
Gm10273	5.2864	-1.5596	6.4516	0.011085304	0.040707025
Cib3	2.7060	-1.9691	6.4448	0.011127535	0.040834155
Dpf1	1.3126	-0.6353	6.4248	0.011253811	0.041194155
Cxcl1	1.1689	0.7298	6.4178	0.011298093	0.041342384
Ifng	3.4175	-2.2245	6.4014	0.011402889	0.041628173
Nsg2	1.8721	0.0129	6.3610	0.011665535	0.042358488
Smim22	3.4065	-2.2280	6.3420	0.011791379	0.042746196
Btbd19	1.4519	0.4343	6.3363	0.011829262	0.042862177
Chil3	2.2800	-1.5304	6.3354	0.011835039	0.042875992
Nrsn2	3.5809	-2.1837	6.3203	0.01193611	0.043184821
Scrt1	1.7562	-1.3998	6.3107	0.012001362	0.043363998
Otp	3.6210	-2.1812	6.2953	0.012106006	0.04366818
4930452B06Rik	3.6277	-2.1809	6.2914	0.012132302	0.043742409

Syce2	1.0355	0.9537	6.1743	0.01296212	0.046162488
Gm12253	2.2045	-1.5711	6.1616	0.013055645	0.046427395
9830107B12Rik	1.1018	0.8939	6.1289	0.013298756	0.047123054
Olfr39	3.3587	-2.2409	6.1123	0.013424505	0.047476166
Kcnrg	1.0342	0.5939	6.0899	0.013595972	0.048012564
Lypd1	1.4769	-0.4879	6.0394	0.013990347	0.049198337
Mkrn2os	3.3419	-2.2455	6.0313	0.014054576	0.049360593

Supplementary Table S2. The genes downregulated in the CHS samples compared with the VT controls

Gene	logFC	logCPM	LR	PValue	FDR
Fcrls	-2.2031	3.9482	102.5085	4.29525E-24	7.44203E-22
Krtap3-2	-2.4250	5.0242	94.5789	2.35516E-22	3.47401E-20
Nkd2	-1.9641	3.6416	84.0154	4.90927E-20	5.55307E-18
Timd4	-3.5948	2.2493	80.2682	3.26881E-19	3.49811E-17
Ccl24	-2.3690	3.3266	76.0868	2.71472E-18	2.62234E-16
Wisp2	-1.7592	3.8375	75.5859	3.49851E-18	3.32069E-16
Osr2	-1.6862	3.1503	74.0311	7.68978E-18	7.12708E-16
Lyve1	-2.4152	3.9931	73.9697	7.93238E-18	7.30683E-16
Krtap3-3	-3.4521	1.6190	73.7246	8.98115E-18	8.16948E-16
Reck	-1.7552	4.4047	73.3501	1.08575E-17	9.78318E-16
Il1f10	-2.1520	3.1976	71.2905	3.08319E-17	2.68164E-15
Prrx1	-1.2748	5.6738	67.5381	2.06655E-16	1.70245E-14
Igfbp6	-1.6903	6.0910	67.2111	2.43942E-16	1.98712E-14
Upk3b	-3.6961	0.6548	67.0889	2.59539E-16	2.10632E-14
Prss23	-1.3583	5.8231	66.5860	3.34954E-16	2.67934E-14
Tesc	-2.2558	4.0008	66.2709	3.93024E-16	3.12004E-14
Crlf1	-1.6614	4.1482	64.7679	8.4259E-16	6.45424E-14
Lrrn4cl	-1.4963	5.1223	63.6333	1.49875E-15	1.12052E-13
Gfpt2	-1.5885	5.2373	62.6903	2.41902E-15	1.76032E-13
Islr2	-2.1799	3.4972	62.3647	2.85393E-15	2.0699E-13
Efhd1	-1.8112	3.4183	62.1961	3.10898E-15	2.24742E-13
Gpx3	-1.5179	8.5929	62.1351	3.20685E-15	2.31052E-13
Ildr2	-2.0084	3.2063	61.5214	4.37964E-15	3.1144E-13
Arhgap20	-1.9407	2.9118	61.2959	4.91111E-15	3.46972E-13
Pcolce2	-1.8134	5.8653	60.6611	6.77957E-15	4.7014E-13
Adrb2	-1.1396	4.6374	60.0092	9.44168E-15	6.46148E-13
Hspa2	-1.2600	3.5975	59.8451	1.02625E-14	6.93622E-13
Clec3b	-1.5974	7.5201	58.0051	2.61439E-14	1.6886E-12
Ltbp4	-1.0780	7.4594	57.7051	3.04513E-14	1.94381E-12
Cyp4v3	-1.6768	4.2109	57.6768	3.08921E-14	1.96048E-12
Mgl2	-2.3999	4.7683	57.6552	3.12335E-14	1.9764E-12
Gypc	-1.3458	3.5865	56.7967	4.83265E-14	2.92248E-12
Igfbp3	-1.2252	7.3046	56.7731	4.891E-14	2.9496E-12
Gpc3	-1.5083	6.2155	56.4173	5.86137E-14	3.51537E-12
Lgr6	-1.3208	5.0695	55.8451	7.84115E-14	4.5648E-12
Lce1m	-1.9006	9.4342	54.1936	1.8168E-13	1.01439E-11
Igfbp5	-1.4116	8.4828	54.0473	1.95722E-13	1.08447E-11
Arsb	-1.2701	4.4724	53.1791	3.04483E-13	1.65353E-11
Plscr4	-1.0932	4.0479	53.0602	3.23482E-13	1.75234E-11
Ar	-1.1037	4.9039	52.6422	4.00191E-13	2.14658E-11
Pi16	-1.6389	7.7134	52.1411	5.16527E-13	2.69768E-11
Epm2a	-1.4695	4.9433	51.8265	6.06273E-13	3.15884E-11
Dennd2a	-1.5610	3.6716	51.6630	6.58928E-13	3.41688E-11
Gstm1	-1.2380	7.0328	51.4805	7.23104E-13	3.71437E-11
Camk2n1	-1.4766	3.6425	51.2413	8.16801E-13	4.17602E-11
Dok2	-1.6227	2.8763	51.1724	8.45977E-13	4.30502E-11
Scara3	-1.6210	5.6082	50.7622	1.04261E-12	5.17298E-11
Plpp3	-1.2751	7.1313	50.4905	1.19744E-12	5.86129E-11
Steap3	-1.3539	5.7897	50.2733	1.3376E-12	6.44615E-11
Slc4a4	-1.5329	3.5110	50.1249	1.44268E-12	6.92199E-11
Ntn1	-1.2888	5.2186	49.5511	1.9327E-12	9.17235E-11
Ehbp1	-1.0828	4.0745	49.2104	2.29928E-12	1.07716E-10
Slc7a2	-1.7703	5.9174	49.1255	2.40093E-12	1.12237E-10
Cpxm2	-1.1974	5.2597	48.7815	2.86125E-12	1.32059E-10
Medag	-1.1784	5.0483	48.5622	3.19971E-12	1.46442E-10
Wnt10b	-1.9707	2.6484	47.8490	4.60347E-12	2.07641E-10

Cebpd	-1.3494	4.9200	47.5710	5.30471E-12	2.36341E-10
Tmem35a	-2.0815	2.3918	47.4587	5.61738E-12	2.49762E-10
Ndn	-1.2253	3.7053	47.4222	5.72303E-12	2.53942E-10
Timp2	-1.3864	8.8147	47.3607	5.90538E-12	2.60972E-10
Klf2	-1.4700	4.4191	47.3374	5.97601E-12	2.6356E-10
Tbxas1	-2.0952	2.0783	46.5710	8.83568E-12	3.76007E-10
Dpp4	-1.3200	6.4452	46.1891	1.07375E-11	4.47347E-10
Tmem177	-1.5256	4.1239	46.0435	1.15655E-11	4.79101E-10
Ccdc149	-1.6747	2.4517	45.8602	1.26999E-11	5.251E-10
Rilpl1	-1.3549	6.2535	45.5435	1.49288E-11	6.09177E-10
Slc17a8	-2.6316	1.1897	44.8489	2.1284E-11	8.51009E-10
Gadd45g	-1.2647	3.6646	43.9031	3.4505E-11	1.32619E-09
Npepl1	-1.0759	5.0679	43.8381	3.56697E-11	1.36137E-09
Plce1	-1.2708	3.3739	43.5438	4.14585E-11	1.56048E-09
Tbc1d4	-1.2227	4.3298	43.2948	4.70824E-11	1.76003E-09
Sfrp4	-2.5199	5.5623	43.2621	4.78757E-11	1.78662E-09
Wfdc21	-1.4561	6.3260	43.1153	5.16083E-11	1.91935E-09
Smoc2	-1.0571	7.0015	42.9496	5.61675E-11	2.07829E-09
P2rx2	-1.9179	2.9916	42.8199	6.00175E-11	2.20417E-09
Fndc1	-1.7666	7.2531	42.7694	6.15877E-11	2.25591E-09
A930018M24Rik	-1.6811	3.5382	42.6542	6.53253E-11	2.38481E-09
Ptgis	-1.9710	2.4346	42.6218	6.64139E-11	2.4205E-09
Btc	-1.8000	5.6925	42.5306	6.95837E-11	2.52759E-09
Fbp2	-2.3455	5.7994	42.3957	7.45544E-11	2.69282E-09
Map4k2	-1.0454	3.8922	42.3621	7.58466E-11	2.72775E-09
Adgrd1	-1.6467	5.6561	42.1330	8.52714E-11	3.04674E-09
Gprc5c	-1.2150	4.1464	41.9836	9.20433E-11	3.26731E-09
Ttc9	-1.1343	4.6293	41.9190	9.51319E-11	3.35513E-09
Lama2	-1.2844	6.0836	41.8553	9.82856E-11	3.46076E-09
Ebf3	-1.3319	2.8307	41.6847	1.07244E-10	3.73406E-09
Anxa3	-1.4848	5.0694	40.9200	1.5859E-10	5.35941E-09
Pink1	-1.2376	8.0425	40.7083	1.76738E-10	5.90867E-09
Dnm1	-1.3001	4.9992	40.6961	1.7784E-10	5.93643E-09
Fbxo31	-1.1372	6.7106	40.6534	1.81769E-10	6.0491E-09
Ramp1	-1.3107	4.8115	40.4392	2.02835E-10	6.70922E-09
Emilin2	-1.2499	5.8520	40.3855	2.08479E-10	6.87508E-09
Npr3	-1.2701	4.0914	40.3008	2.17716E-10	7.16886E-09
Dact1	-1.4351	3.2615	40.2161	2.27363E-10	7.44162E-09
Fcna	-2.2134	4.8305	40.1866	2.30829E-10	7.52968E-09
Dcn	-1.2914	9.6141	40.1844	2.31088E-10	7.52968E-09
6030419C18Rik	-1.9702	2.8051	39.9200	2.6458E-10	8.53183E-09
Serpinf1	-1.2685	5.7844	39.9060	2.66488E-10	8.58067E-09
Coro2b	-1.1381	3.9609	39.8420	2.7536E-10	8.82728E-09
Cuedc1	-1.3400	5.1204	39.4939	3.29088E-10	1.04423E-08
Mturn	-1.2820	4.8616	39.4605	3.34765E-10	1.0607E-08
Gstt1	-1.3259	4.0532	39.4342	3.39304E-10	1.07353E-08
Lrrc17	-1.2338	4.0360	39.2274	3.77203E-10	1.18652E-08
Mt4	-1.9595	5.3723	38.9008	4.45889E-10	1.38467E-08
Bicc1	-1.0690	4.5693	38.8171	4.65426E-10	1.43919E-08
Gsn	-1.0572	11.3967	38.6899	4.96775E-10	1.53179E-08
Dpep1	-1.1541	6.3997	38.6274	5.1295E-10	1.57944E-08
Mapk1ip1	-1.0734	4.2684	38.4113	5.73011E-10	1.74712E-08
Slc43a1	-1.1662	4.1809	38.2483	6.2291E-10	1.8861E-08
Ulk2	-1.0403	5.3217	38.2429	6.24624E-10	1.88867E-08
AW551984	-1.7194	4.2949	38.0822	6.78272E-10	2.03119E-08
Plekho2	-1.0779	4.5990	37.9322	7.32446E-10	2.18443E-08
Txnip	-1.0055	9.4302	37.9289	7.33714E-10	2.18523E-08
Nova1	-1.4739	5.1413	37.7176	8.17653E-10	2.41545E-08

Islr	-1.3306	5.7273	37.7049	8.22986E-10	2.42792E-08
Kcnj16	-2.1309	2.7493	37.6370	8.52122E-10	2.5071E-08
Trim63	-1.4430	7.2724	37.6249	8.5741E-10	2.51588E-08
Folr2	-1.6508	5.4656	37.5478	8.91982E-10	2.60681E-08
Abca8a	-1.6647	6.0876	37.4651	9.30628E-10	2.71249E-08
Tek	-1.0432	5.0008	37.2355	1.0469E-09	3.01517E-08
Gpc6	-1.4525	4.1319	37.2056	1.0631E-09	3.04975E-08
Chrdl1	-1.7647	4.9907	37.1505	1.09353E-09	3.12693E-08
Fgfr1	-1.1455	6.6441	37.1492	1.0943E-09	3.12693E-08
Dpt	-1.0432	8.6653	37.0582	1.14658E-09	3.25499E-08
Cd34	-1.1028	7.4405	37.0531	1.14955E-09	3.2592E-08
Cyp26b1	-1.0012	6.6307	36.7992	1.30945E-09	3.67911E-08
4931408C20Rik	-3.7842	-0.0748	36.7503	1.34267E-09	3.75792E-08
Itgbl1	-1.1462	4.5368	36.6332	1.4258E-09	3.96517E-08
Camk2b	-1.1015	5.6718	36.5402	1.49552E-09	4.13797E-08
Cped1	-1.0158	5.1644	36.3215	1.67304E-09	4.58271E-08
Spsb1	-1.1903	4.6434	36.3165	1.67737E-09	4.58879E-08
Cd209b	-1.4409	5.5402	36.0943	1.87993E-09	5.10879E-08
Ccl9	-1.4986	4.8062	36.0716	1.90197E-09	5.15799E-08
Iffo1	-1.7852	2.0877	36.0311	1.9419E-09	5.25976E-08
Gm9747	-1.0730	4.5599	36.0024	1.97076E-09	5.3313E-08
Zrsr1	-1.0655	3.4944	35.8979	2.07935E-09	5.59042E-08
Rab12	-1.0348	5.7553	35.8859	2.09214E-09	5.611E-08
Pik3ip1	-1.1723	5.4435	35.7703	2.22006E-09	5.92255E-08
Cmbl	-1.4081	4.8781	35.6122	2.40777E-09	6.37141E-08
Fam180a	-1.5739	2.1412	35.5204	2.52397E-09	6.65469E-08
Kcnq5	-1.3402	3.7988	35.5037	2.54572E-09	6.70394E-08
Cd209f	-1.5865	4.6186	35.4463	2.62186E-09	6.89611E-08
Gm9780	-1.2826	2.9896	35.3526	2.751E-09	7.19247E-08
Mfsd7b	-1.2521	3.8548	35.2428	2.91061E-09	7.59159E-08
Bach2	-1.0635	3.1055	35.1230	3.09525E-09	8.05393E-08
Hcn4	-2.1641	1.0904	35.0542	3.20653E-09	8.31376E-08
Abca9	-1.3459	5.4336	35.0142	3.27309E-09	8.47627E-08
Gabbr1	-1.1539	4.2406	35.0031	3.29185E-09	8.51473E-08
Dennd4b	-1.2987	5.2509	34.9663	3.35468E-09	8.66699E-08
Ear2	-2.9749	1.6181	34.9566	3.37131E-09	8.69965E-08
Fbxo32	-1.3747	8.4045	34.7025	3.84134E-09	9.79677E-08
Adcy2	-1.2250	4.3476	34.5959	4.05752E-09	1.0312E-07
Car8	-2.2487	2.0753	34.5775	4.09623E-09	1.03982E-07
Klf9	-1.2332	6.0640	34.5727	4.10625E-09	1.04115E-07
F830045P16Rik	-2.5069	2.5915	34.5065	4.24821E-09	1.0759E-07
Arl3	-1.1377	4.1261	34.4754	4.31666E-09	1.08722E-07
Tpbgl	-1.5837	4.4973	34.4349	4.40756E-09	1.10727E-07
Crispld1	-1.8825	2.0056	34.3029	4.7168E-09	1.18088E-07
Mpdz	-1.0560	5.3052	34.2909	4.74602E-09	1.18683E-07
Cd209a	-1.1148	3.3715	34.2112	4.94447E-09	1.22941E-07
Phf24	-1.1514	3.8366	34.0776	5.2958E-09	1.30194E-07
Cpq	-1.1883	4.6103	34.0722	5.31034E-09	1.30405E-07
Acsl6	-1.5381	3.3134	34.0438	5.38842E-09	1.32174E-07
C1qtnf1	-1.3457	5.2319	34.0096	5.48417E-09	1.34221E-07
Pdk4	-1.4134	9.1920	33.9484	5.65919E-09	1.37451E-07
Bmp4	-1.2892	3.7902	33.9481	5.66022E-09	1.37451E-07
Cldn23	-1.2301	5.1078	33.9165	5.75288E-09	1.39391E-07
Zfhx4	-1.3009	3.0950	33.7430	6.2895E-09	1.51218E-07
Klf15	-1.1284	4.8786	33.7148	6.38136E-09	1.5309E-07
St6galnac6	-1.2970	4.1231	33.6591	6.56669E-09	1.57068E-07
Aldh1a2	-2.3802	0.9775	33.5645	6.89393E-09	1.64662E-07
P2rx6	-1.5710	2.5398	33.4919	7.15617E-09	1.69443E-07

Sema3b	-1.2792	4.0948	33.4417	7.34311E-09	1.73118E-07
Ogn	-1.5271	6.9318	33.3834	7.56673E-09	1.77844E-07
Cacna1e	-1.4087	2.8248	33.3563	7.67279E-09	1.79775E-07
Cpeb1	-1.1154	3.5378	33.3558	7.67489E-09	1.79775E-07
Glt8d2	-1.7347	2.5927	33.3107	7.85481E-09	1.83596E-07
Spock2	-1.1567	3.8787	33.2716	8.01427E-09	1.86923E-07
Opcml	-1.7539	3.3182	33.2242	8.21223E-09	1.91335E-07
Podxl2	-1.3439	3.9764	33.1721	8.43513E-09	1.95901E-07
Smco1	-1.5168	3.3379	33.1161	8.68189E-09	2.01204E-07
Krt36	-2.1934	2.0582	33.0827	8.83193E-09	2.04183E-07
Asb11	-1.4383	5.5885	33.0632	8.92131E-09	2.05879E-07
Sorbs3	-1.0800	6.0838	33.0190	9.12615E-09	2.0994E-07
Pamr1	-1.2249	4.5101	32.8493	9.95875E-09	2.27654E-07
Dhdh	-1.0078	4.4818	32.4596	1.21697E-08	2.74459E-07
Nadk2	-1.0736	4.0576	32.2770	1.33684E-08	2.99023E-07
Rspo3	-2.1732	1.1905	32.1678	1.41412E-08	3.15338E-07
Sfrp2	-2.1629	5.4538	32.1144	1.45354E-08	3.23138E-07
Mb	-1.3537	8.5951	31.9871	1.552E-08	3.42585E-07
Gmpr	-1.5727	5.8765	31.9724	1.56375E-08	3.44482E-07
Spock3	-3.3208	0.2129	31.9015	1.6219E-08	3.55857E-07
Slc38a4	-1.2293	3.8720	31.7768	1.72943E-08	3.76423E-07
Lyz1	-2.9397	4.8411	31.6856	1.81263E-08	3.93746E-07
Tnxb	-1.0407	8.8145	31.6609	1.83584E-08	3.98392E-07
Fgl2	-1.0821	5.7076	31.5296	1.96424E-08	4.25182E-07
Col4a6	-2.2772	4.3704	31.5107	1.98342E-08	4.2829E-07
Repin1	-1.0357	3.9466	31.4826	2.01236E-08	4.3368E-07
Akr1c14	-2.1407	4.6452	31.4058	2.09356E-08	4.49381E-07
Arhgef6	-1.0445	4.3356	31.3585	2.14519E-08	4.5734E-07
Fhod3	-1.2264	4.1711	31.2818	2.23162E-08	4.72079E-07
Pfn2	-1.5502	5.8083	31.2520	2.26618E-08	4.78229E-07
Mmp23	-1.3885	3.2357	31.2386	2.28186E-08	4.80843E-07
Osr1	-1.5459	2.8652	31.1598	2.3764E-08	4.98838E-07
Asb16	-1.4421	5.2472	30.9765	2.61177E-08	5.40451E-07
Cbx7	-1.0438	5.5511	30.9262	2.68026E-08	5.5305E-07
Gem	-1.0618	3.7286	30.9038	2.71135E-08	5.5841E-07
Samd4	-1.3436	4.9186	30.8851	2.7377E-08	5.62784E-07
Vamp5	-1.4055	4.9342	30.7789	2.89161E-08	5.91075E-07
Tspyl4	-1.2279	2.3786	30.5866	3.1929E-08	6.46007E-07
Cdk14	-1.2311	3.4648	30.5845	3.1963E-08	6.46097E-07
Tmeff2	-1.2907	4.0446	30.5555	3.24452E-08	6.54027E-07
Plac9b	-1.1363	3.4346	30.4254	3.46957E-08	6.9682E-07
Tmod2	-1.0887	3.8494	30.3842	3.54412E-08	7.10483E-07
Fam13c	-1.2340	2.2876	30.3714	3.56745E-08	7.14505E-07
Micu1	-1.0213	6.6252	30.3683	3.57313E-08	7.14986E-07
Aamdc	-1.3469	3.9492	30.3454	3.61564E-08	7.21911E-07
Wfikkn2	-1.6950	2.4722	30.3114	3.67963E-08	7.32938E-07
Car11	-2.1957	0.9702	30.1241	4.05273E-08	7.98511E-07
D7Ertd443e	-1.3980	3.2845	29.9907	4.34129E-08	8.46202E-07
Dnajc27	-1.2640	4.3531	29.9103	4.52506E-08	8.796E-07
Gm15737	-2.1686	1.2747	29.8973	4.55537E-08	8.832E-07
Dbn1	-1.1260	4.0715	29.8845	4.58569E-08	8.8829E-07
Isl1	-1.5892	1.5866	29.8611	4.64125E-08	8.98255E-07
Ly6c1	-1.1275	6.5697	29.8565	4.65241E-08	8.99618E-07
Asap3	-1.0000	4.6100	29.8476	4.67384E-08	9.02961E-07
Fcgrt	-1.0269	6.7708	29.8372	4.69878E-08	9.06723E-07
Nhlrc1	-1.4341	2.1780	29.8290	4.71878E-08	9.0923E-07
Foxo6	-1.8017	1.5181	29.6636	5.1392E-08	9.82433E-07
Efcab2	-1.5029	2.3589	29.6142	5.27179E-08	1.00426E-06

Aldh1a1	-1.2591	5.9765	29.5750	5.37937E-08	1.02208E-06
Zfp608	-1.1611	2.6090	29.5451	5.463E-08	1.03437E-06
Zfpm2	-2.3480	0.5054	29.4329	5.78865E-08	1.09311E-06
Bhlha9	-5.7109	-1.1365	29.2721	6.28934E-08	1.17957E-06
Klhl38	-1.3295	4.8777	29.2459	6.37506E-08	1.1936E-06
Fxyd1	-1.2081	6.2960	29.1922	6.5543E-08	1.22296E-06
Gfra2	-1.3025	3.3900	29.1530	6.68822E-08	1.24689E-06
Emx2	-1.7620	1.7432	28.9437	7.45129E-08	1.37273E-06
Drp2	-1.1698	3.2307	28.8367	7.87455E-08	1.44705E-06
B230118H07Rik	-1.1188	3.9317	28.8272	7.91312E-08	1.45092E-06
Svil	-1.1097	6.6924	28.8233	7.92895E-08	1.45216E-06
Galnt15	-1.3533	6.9123	28.7616	8.18595E-08	1.49671E-06
Pnck	-1.5834	1.7669	28.7267	8.33455E-08	1.52261E-06
Asrgl1	-1.1611	4.0062	28.7239	8.3467E-08	1.52355E-06
Cfp	-1.0804	5.3466	28.6793	8.54128E-08	1.55387E-06
Cul9	-1.1158	3.9215	28.6682	8.59042E-08	1.56021E-06
Fam57b	-1.3272	4.6725	28.6624	8.61599E-08	1.56356E-06
Calml4	-1.5608	2.4107	28.6038	8.88061E-08	1.60757E-06
Rab11fip4	-1.9551	6.3894	28.5966	8.91384E-08	1.61225E-06
Fgd4	-1.2315	3.0971	28.5493	9.13411E-08	1.64799E-06
Aox1	-1.0936	4.7941	28.5290	9.23037E-08	1.66261E-06
F13a1	-1.2807	6.8584	28.4111	9.80995E-08	1.75686E-06
Lrrk2	-1.1026	2.7977	28.4043	9.84451E-08	1.7616E-06
Arhgap44	-1.0813	3.8209	28.3813	9.96211E-08	1.77682E-06
Aldh3a1	-1.0651	5.8378	28.3734	1.0003E-07	1.7812E-06
Prkab2	-1.2898	6.5446	28.3321	1.02187E-07	1.81517E-06
1500011K16Rik	-1.0008	4.2662	28.3263	1.02493E-07	1.81912E-06
Ube2d1	-1.0766	5.9327	28.3186	1.02903E-07	1.82492E-06
Ifitm6	-2.2880	5.3420	28.1917	1.09877E-07	1.94072E-06
Nqo2	-1.0202	4.2466	28.0670	1.17189E-07	2.0582E-06
Lepr	-1.0744	3.7466	28.0535	1.18008E-07	2.07093E-06
Aebp1	-1.0534	7.6184	28.0160	1.20319E-07	2.1081E-06
Tigar	-1.1284	5.3719	27.9144	1.26804E-07	2.20578E-06
Scara5	-1.2785	6.8338	27.8607	1.30369E-07	2.26418E-06
Itgam	-1.2466	4.3566	27.8526	1.30921E-07	2.27197E-06
Mrgprb2	-1.2436	3.5991	27.7157	1.40515E-07	2.42881E-06
Ptp4a3	-1.4075	8.3345	27.6573	1.44828E-07	2.49545E-06
Fn3k	-1.8242	1.8625	27.6049	1.48799E-07	2.55782E-06
2210011C24Rik	-2.4000	0.3211	27.5618	1.52155E-07	2.61139E-06
Twf2	-1.0724	5.7971	27.4699	1.59559E-07	2.72135E-06
Socs2	-1.0099	3.2062	27.4218	1.63579E-07	2.77691E-06
Adssl1	-1.5668	8.0342	27.3865	1.66591E-07	2.82364E-06
Dnajb5	-1.2131	5.2741	27.3536	1.6945E-07	2.86321E-06
Vldlr	-1.0537	6.5557	27.2963	1.74545E-07	2.93566E-06
Cdh15	-1.4092	2.7325	27.2867	1.75417E-07	2.94806E-06
Serping1	-1.0613	8.0520	27.2204	1.81535E-07	3.0415E-06
Gabra3	-1.3906	3.1688	27.1982	1.83634E-07	3.07432E-06
Peg10	-2.1984	0.5975	27.1019	1.93007E-07	3.21644E-06
Dmpk	-1.4753	5.4852	27.0779	1.95415E-07	3.24418E-06
Pla2g16	-1.0104	6.2927	27.0676	1.96468E-07	3.25919E-06
Asb18	-1.4053	2.9338	27.0152	2.01862E-07	3.34106E-06
Twist1	-1.2106	3.0114	26.8710	2.17495E-07	3.58891E-06
Tgfb2	-1.1809	3.4525	26.8545	2.19363E-07	3.60994E-06
St6galnac4	-1.1586	3.0095	26.8441	2.20544E-07	3.62553E-06
Cbs	-1.4001	4.0396	26.8358	2.21496E-07	3.6357E-06
Dcaf6	-1.0209	6.1118	26.8234	2.22925E-07	3.65366E-06
Otud1	-1.3252	5.6896	26.7665	2.29579E-07	3.74305E-06
C4b	-1.3804	6.8272	26.6931	2.38469E-07	3.87929E-06

Ebf2	-1.4338	3.0690	26.6763	2.40558E-07	3.91036E-06
Timp3	-1.1413	6.5016	26.6572	2.42943E-07	3.94033E-06
Slit3	-1.1461	6.0035	26.6332	2.45986E-07	3.98377E-06
Kbtbd12	-1.5641	5.2559	26.5700	2.54153E-07	4.1078E-06
Basp1	-1.1026	2.5007	26.5027	2.63172E-07	4.23696E-06
Gal3st1	-1.0933	4.5142	26.4970	2.63947E-07	4.24562E-06
Adam33	-1.3725	2.6145	26.4458	2.71032E-07	4.34108E-06
Mlycd	-1.2054	5.4563	26.4029	2.77126E-07	4.4257E-06
Tbc1d19	-1.1846	2.8934	26.3726	2.81508E-07	4.48256E-06
Prob1	-1.3426	5.6469	26.3666	2.82381E-07	4.48666E-06
Nos1	-1.5717	5.1361	26.3437	2.85757E-07	4.5304E-06
Tbx15	-1.1846	6.2463	26.3043	2.91634E-07	4.61353E-06
Traf5	-1.2281	2.2793	26.1970	3.083E-07	4.84208E-06
Tmod4	-1.4300	6.5414	26.1848	3.10262E-07	4.8694E-06
Gstp2	-1.4538	4.6436	26.1675	3.13043E-07	4.90951E-06
Tmem37	-1.2336	3.1518	26.1274	3.19609E-07	4.99126E-06
Pygo1	-1.3936	3.4691	26.1009	3.24026E-07	5.04594E-06
Cd55	-1.0319	5.7797	26.1008	3.24053E-07	5.04594E-06
Wnt9a	-1.0500	2.9217	26.0130	3.39126E-07	5.23212E-06
Serpina5	-2.7306	-0.2420	25.9450	3.51287E-07	5.40372E-06
Patz1	-1.3024	3.1517	25.9042	3.58785E-07	5.51206E-06
Fermt2	-1.0891	6.3181	25.8587	3.67354E-07	5.62786E-06
Cd248	-1.3570	5.9222	25.8516	3.68697E-07	5.64052E-06
Mettl23	-1.2108	2.6183	25.8111	3.76516E-07	5.75208E-06
Tmem246	-1.4222	3.1554	25.7063	3.97521E-07	6.04759E-06
Ank2	-1.3023	3.3811	25.6834	4.02273E-07	6.11136E-06
Rtn2	-1.5202	7.6996	25.6812	4.0274E-07	6.1142E-06
Zfp334	-1.2363	2.0804	25.6714	4.04772E-07	6.13651E-06
Efemp1	-1.1427	6.7355	25.4520	4.53534E-07	6.81425E-06
Sprn	-1.6341	0.8708	25.4407	4.56183E-07	6.84463E-06
Sgcd	-1.1279	2.2568	25.4060	4.64467E-07	6.95458E-06
Mest	-1.4086	3.4202	25.4011	4.65658E-07	6.9581E-06
Hdac9	-1.2574	2.4353	25.3974	4.66554E-07	6.96671E-06
Triqk	-2.1108	0.0850	25.3683	4.7363E-07	7.05789E-06
Mrln	-2.0185	2.4955	25.3656	4.743E-07	7.06306E-06
Lvrn	-1.7169	3.4605	25.3622	4.75135E-07	7.06586E-06
Hoxa10	-2.8017	-0.0858	25.3431	4.79871E-07	7.12658E-06
Nanp	-7.3097	0.1116	25.3246	4.84482E-07	7.18039E-06
Npy1r	-1.3439	3.5705	25.2650	4.99695E-07	7.37583E-06
Stbd1	-1.3799	3.3564	25.1844	5.21027E-07	7.65447E-06
Rai2	-1.1089	2.8360	25.1620	5.27094E-07	7.73319E-06
Prkaa2	-1.3849	6.1475	25.1512	5.30062E-07	7.77152E-06
Rgs6	-1.9900	0.7924	25.1317	5.35441E-07	7.83985E-06
Pgam2	-1.4849	7.8553	25.0732	5.51937E-07	8.05437E-06
Trim54	-1.5456	6.0923	25.0388	5.61893E-07	8.19418E-06
Asb10	-1.2151	4.5694	25.0329	5.636E-07	8.21359E-06
2310002L09Rik	-1.5576	4.4593	25.0135	5.69295E-07	8.28552E-06
Coro6	-1.3249	6.4486	24.9891	5.76556E-07	8.37445E-06
Pla1a	-2.0411	4.5295	24.9568	5.86306E-07	8.5104E-06
Tsc22d3	-1.3582	7.4207	24.9530	5.87457E-07	8.52145E-06
Pltp	-1.0576	6.7714	24.8565	6.17605E-07	8.89962E-06
Zbtb20	-1.1086	5.6277	24.8277	6.26895E-07	9.0097E-06
Pard3b	-1.1872	3.0012	24.7924	6.38493E-07	9.14628E-06
BC067074	-1.0116	2.9283	24.6950	6.71583E-07	9.55759E-06
Pitx2	-1.4728	2.5089	24.6764	6.78084E-07	9.64382E-06
Slit2	-1.1814	3.0662	24.6438	6.89648E-07	9.78914E-06
Klhl33	-1.5382	4.6898	24.6407	6.90787E-07	9.79894E-06
Gkap1	-1.1348	2.9743	24.6032	7.04338E-07	9.9673E-06

Kazald1	-1.6391	2.0466	24.5967	7.06717E-07	9.98599E-06
Stxbp6	-1.4010	3.3437	24.5147	7.37465E-07	1.04003E-05
Slc27a2	-2.2784	0.6623	24.4955	7.44833E-07	1.04771E-05
Rragd	-1.4512	5.9616	24.4341	7.68966E-07	1.07888E-05
Eef1a2	-1.4349	8.9162	24.4068	7.79942E-07	1.09287E-05
Art5	-1.3786	4.0167	24.3956	7.84478E-07	1.09852E-05
Pfkm	-1.4853	10.0604	24.3328	8.10484E-07	1.12986E-05
Cib2	-1.2786	5.5331	24.2971	8.2562E-07	1.1473E-05
Mlxipl	-1.1717	3.9649	24.2888	8.29203E-07	1.15082E-05
Klhl31	-1.4506	6.3784	24.2299	8.54939E-07	1.18352E-05
Rpl3l	-1.3959	6.8481	24.2192	8.59688E-07	1.18709E-05
Prrg1	-1.5040	1.2046	24.2155	8.6136E-07	1.18843E-05
Il31ra	-1.2481	3.5232	24.1615	8.85872E-07	1.21785E-05
Pbxip1	-1.1502	7.2945	24.1228	9.03858E-07	1.23868E-05
Galnt16	-1.1872	4.5982	24.1102	9.09783E-07	1.24523E-05
Mtss1l	-1.0956	5.4348	24.0911	9.18831E-07	1.25604E-05
Mettl22	-1.1522	4.4166	24.0852	9.21672E-07	1.25914E-05
Sdc2	-1.0851	4.7749	24.0767	9.25746E-07	1.26312E-05
Col14a1	-1.1424	6.7742	24.0556	9.35927E-07	1.27622E-05
Abcc9	-1.2736	6.1654	24.0413	9.42891E-07	1.28411E-05
Epdr1	-1.2507	4.9853	24.0366	9.45233E-07	1.28623E-05
Sebox	-1.7589	1.6805	24.0311	9.47941E-07	1.28777E-05
Nipsnap2	-1.0285	7.1479	23.9431	9.92277E-07	1.34049E-05
Pf4	-1.0147	3.9663	23.9372	9.95282E-07	1.34372E-05
Slc25a4	-1.2586	9.5448	23.8714	1.02992E-06	1.38534E-05
Acyp2	-1.3684	4.4298	23.8547	1.03889E-06	1.39483E-05
Ehbp1l1	-1.0204	6.7969	23.8383	1.04776E-06	1.40588E-05
Rps6ka2	-1.1981	5.6035	23.8286	1.05308E-06	1.41042E-05
Nectin3	-1.3614	5.4946	23.7243	1.1117E-06	1.48166E-05
Cacng1	-1.4281	5.9511	23.6913	1.13093E-06	1.50453E-05
Clec4b1	-2.6543	0.3185	23.6840	1.13521E-06	1.50839E-05
Zfp358	-1.0158	4.4108	23.6501	1.15541E-06	1.53335E-05
Gpr1	-1.6958	2.4090	23.6387	1.16229E-06	1.54155E-05
Ism1	-1.1544	3.3840	23.6280	1.16876E-06	1.54747E-05
Kctd12	-1.5285	7.6057	23.6278	1.16888E-06	1.54747E-05
Prkg1	-1.1348	3.1318	23.5316	1.22882E-06	1.62289E-05
Atp1a2	-1.1567	9.2385	23.5272	1.23158E-06	1.62456E-05
Ackr2	-1.2631	4.7803	23.5236	1.23393E-06	1.62668E-05
Themis2	-1.2688	3.0945	23.4917	1.25455E-06	1.64989E-05
Magix	-1.4127	2.3360	23.3679	1.33791E-06	1.74898E-05
Acss1	-1.3489	6.2647	23.3036	1.3834E-06	1.80304E-05
Abca6	-1.6660	3.5003	23.2476	1.42429E-06	1.85302E-05
Gyg	-1.1588	7.0386	23.2089	1.45321E-06	1.88727E-05
Rasgrp3	-1.3523	3.7601	23.1951	1.4637E-06	1.89976E-05
Gapdh	-1.1055	8.4549	23.1685	1.48409E-06	1.92052E-05
Zic1	-1.0905	2.7601	23.1042	1.53452E-06	1.9764E-05
Bmp8a	-3.8102	-0.0235	23.1023	1.53604E-06	1.9772E-05
Snrpn	-1.3404	3.3521	23.1012	1.53695E-06	1.97721E-05
Prdm8	-1.8213	2.0090	23.0770	1.55643E-06	1.99704E-05
Nid1	-1.1549	7.6824	23.0664	1.56498E-06	2.00617E-05
Mylk2	-1.4182	9.1361	23.0405	1.58625E-06	2.03105E-05
Gng4	-1.2678	4.1440	23.0321	1.59319E-06	2.03875E-05
Prf1	-1.3604	2.3660	22.9321	1.67822E-06	2.13878E-05
Abcb4	-1.3982	4.8184	22.9174	1.69116E-06	2.15276E-05
Kif19a	-1.8302	0.1935	22.8160	1.78277E-06	2.25884E-05
Olfml1	-1.2324	3.5802	22.7675	1.8283E-06	2.31517E-05
Wbscr17	-1.2274	2.8767	22.7646	1.8311E-06	2.31737E-05
Mfap5	-1.0278	5.5563	22.7476	1.84735E-06	2.33253E-05

Cox8b	-1.3286	5.5632	22.7425	1.85225E-06	2.33737E-05
Rbm24	-1.3349	5.2523	22.7401	1.85456E-06	2.33894E-05
Fam117a	-1.0088	3.8960	22.7242	1.87001E-06	2.35706E-05
Tlr4	-1.1823	3.0928	22.6643	1.92921E-06	2.42328E-05
Shisa3	-1.7172	0.7337	22.6271	1.96696E-06	2.46502E-05
Klhdc1	-1.0232	3.9745	22.6072	1.98743E-06	2.4864E-05
Adgra3	-1.2187	6.6724	22.5962	1.99884E-06	2.4957E-05
Cilp	-1.2138	6.5773	22.5557	2.04138E-06	2.54079E-05
Amot	-1.3422	4.8253	22.5362	2.06224E-06	2.56091E-05
Eya4	-1.7177	3.1260	22.5115	2.08893E-06	2.58963E-05
Enpep	-1.5362	3.9719	22.4982	2.10338E-06	2.60607E-05
Camk2a	-1.5150	5.8361	22.4880	2.11456E-06	2.61591E-05
Atp6v0e2	-1.1612	4.4380	22.4875	2.11513E-06	2.61591E-05
Plac8	-1.2554	5.0396	22.4866	2.11612E-06	2.61591E-05
Akap17b	-1.6224	2.0723	22.4522	2.15438E-06	2.65987E-05
Cxcl13	-2.1801	4.5026	22.3774	2.23993E-06	2.75958E-05
Ccl6	-1.1623	5.5108	22.3575	2.26329E-06	2.78679E-05
Fam171b	-1.4312	2.4470	22.3506	2.27147E-06	2.79371E-05
Myf6	-1.4497	4.3033	22.2933	2.34025E-06	2.87184E-05
Adgre4	-1.7489	1.4801	22.2325	2.41555E-06	2.9576E-05
Klhl30	-1.3558	4.0674	22.2263	2.42337E-06	2.96551E-05
Ak1	-1.4347	7.7235	22.2006	2.45594E-06	3.00032E-05
Hrasls	-1.6809	3.2574	22.1402	2.5344E-06	3.08581E-05
Aldoa	-1.2185	12.3941	22.0688	2.63046E-06	3.1992E-05
Fn1	-1.6375	8.5960	22.0477	2.6596E-06	3.22744E-05
Scn1b	-1.1680	7.2876	22.0019	2.72374E-06	3.29978E-05
Six1	-1.5353	3.8997	21.9987	2.72835E-06	3.30352E-05
Mlf1	-1.5915	5.8687	21.9903	2.74033E-06	3.31256E-05
Fat4	-1.0087	3.2349	21.9689	2.77102E-06	3.34222E-05
Tmem229a	-1.3331	3.0844	21.9014	2.87028E-06	3.45241E-05
Adamtsl1	-1.6537	3.4158	21.8971	2.87661E-06	3.45811E-05
Pnpla7	-1.2009	5.0195	21.8637	2.92725E-06	3.50547E-05
Scn7a	-1.2218	4.8352	21.8606	2.93199E-06	3.5073E-05
Cdnf	-1.0242	3.6021	21.8201	2.99451E-06	3.578E-05
Ptprd	-1.0730	2.3040	21.8191	2.99601E-06	3.578E-05
Tspyl3	-1.0206	1.9685	21.7908	3.04056E-06	3.6213E-05
Pkia	-1.3646	6.5870	21.7644	3.08269E-06	3.66747E-05
Pygm	-1.5727	10.9552	21.7066	3.17701E-06	3.76833E-05
Lrrn1	-1.1012	2.5946	21.7016	3.18528E-06	3.77103E-05
Cmya5	-1.3369	9.4905	21.6752	3.22947E-06	3.81507E-05
Rcsd1	-1.2359	5.9313	21.6643	3.24778E-06	3.83462E-05
Rcan2	-1.0698	4.4323	21.6401	3.28906E-06	3.87708E-05
Art1	-1.2653	6.6517	21.6281	3.30962E-06	3.895E-05
Clec10a	-1.2445	5.9384	21.5969	3.36401E-06	3.9494E-05
Tnfrsf11b	-1.5049	3.3939	21.5261	3.49039E-06	4.07916E-05
Adamts20	-1.9146	0.4516	21.4947	3.54814E-06	4.13559E-05
Pdgfd	-1.3573	2.6194	21.4893	3.5581E-06	4.14497E-05
Ttc7b	-1.0039	4.5378	21.4665	3.60071E-06	4.19238E-05
Slc38a3	-1.6286	3.9062	21.4601	3.61277E-06	4.20418E-05
Macrod1	-1.1058	5.8041	21.4553	3.62178E-06	4.21017E-05
Irf4	-1.2808	4.6353	21.4069	3.71432E-06	4.30857E-05
Sh3bgr	-1.1944	5.7870	21.3861	3.7548E-06	4.3486E-05
Pde4dip	-1.3128	10.0356	21.3713	3.78395E-06	4.38003E-05
Greb1	-1.8484	1.8834	21.3394	3.84744E-06	4.4441E-05
Msrb3	-1.0125	5.4575	21.2529	4.02492E-06	4.62722E-05
Sobp	-1.4802	2.3285	21.2529	4.02505E-06	4.62722E-05
Cavin4	-1.5247	5.1334	21.2327	4.06761E-06	4.66632E-05
Phkg1	-1.6104	5.9127	21.1378	4.27404E-06	4.87241E-05

Nptxr	-1.0068	2.2188	21.1178	4.31892E-06	4.9133E-05
Tmem132c	-2.1016	1.6887	21.0988	4.36191E-06	4.95705E-05
Pcdh20	-1.5550	1.7544	21.0767	4.41273E-06	5.00958E-05
Rcn3	-1.0128	5.2128	21.0311	4.51889E-06	5.10904E-05
Klhl29	-1.0291	2.5458	21.0078	4.57416E-06	5.16065E-05
Synm	-1.3242	7.9632	20.9969	4.60019E-06	5.18733E-05
Art3	-1.0048	6.0114	20.9850	4.62883E-06	5.21693E-05
Phkb	-1.1297	6.9782	20.9803	4.64024E-06	5.22695E-05
Skint6	-1.0982	3.4822	20.9585	4.69343E-06	5.27884E-05
Tnni2	-1.4930	10.5585	20.8795	4.89099E-06	5.47565E-05
Tubb4a	-1.4321	2.1653	20.8206	5.04387E-06	5.63236E-05
Tnni3k	-2.9496	-0.6638	20.7988	5.10162E-06	5.69104E-05
Mapk12	-1.1377	5.3587	20.7954	5.11059E-06	5.69813E-05
Unc45b	-1.3970	6.3202	20.7055	5.35632E-06	5.93874E-05
Cadm3	-1.2246	5.7196	20.6704	5.4554E-06	6.01803E-05
Dkk2	-1.3046	2.4999	20.6048	5.6453E-06	6.21139E-05
Cdh9	-2.0369	0.9920	20.6040	5.64775E-06	6.21139E-05
Synpo2	-1.1035	7.5321	20.5549	5.79456E-06	6.35364E-05
Katnal2	-1.3664	2.9116	20.5382	5.84517E-06	6.39949E-05
Fbxo40	-1.3984	5.9405	20.5199	5.90132E-06	6.44159E-05
Psd2	-3.2585	-0.7358	20.4878	6.00127E-06	6.53109E-05
Abca8b	-1.4715	4.2508	20.4751	6.04114E-06	6.57121E-05
Plcl2	-1.1491	4.4576	20.4426	6.14466E-06	6.66058E-05
Myoz1	-1.6316	8.6896	20.4006	6.281E-06	6.79151E-05
Adam22	-1.2747	2.0394	20.3935	6.3042E-06	6.81322E-05
Six4	-1.2647	3.0165	20.3483	6.45518E-06	6.9626E-05
Hfe2	-1.4392	6.6904	20.3279	6.52424E-06	7.03362E-05
Dcaf12l1	-2.8122	-0.7602	20.1717	7.07921E-06	7.55358E-05
Cox6a2	-1.2865	7.1600	20.1707	7.08312E-06	7.55406E-05
Trim72	-1.0447	5.6800	20.1574	7.13254E-06	7.60305E-05
Txlnb	-1.3153	7.6542	20.1247	7.25523E-06	7.72252E-05
Ms4a4d	-1.1426	3.9483	20.0943	7.37172E-06	7.83505E-05
Asb5	-1.3353	5.6100	20.0209	7.66E-06	8.09417E-05
Ccdc27	-1.5035	0.9460	20.0149	7.68418E-06	8.11186E-05
Draxin	-1.7168	1.3163	20.0060	7.71995E-06	8.14569E-05
Svip	-1.1421	3.5255	19.9842	7.80846E-06	8.22714E-05
Dtna	-1.1465	4.4073	19.9485	7.95564E-06	8.37817E-05
Bmpr1b	-1.0753	1.8595	19.9328	8.02122E-06	8.4391E-05
1110002E22Rik	-1.4514	5.7392	19.9288	8.03794E-06	8.45262E-05
Ablim3	-1.0549	4.2721	19.9249	8.05441E-06	8.46326E-05
Atp1b4	-1.7951	2.2200	19.9206	8.07254E-06	8.47675E-05
Esr1	-1.0453	4.3215	19.9159	8.09229E-06	8.49341E-05
Syt3	-1.4978	1.1709	19.8610	8.32819E-06	8.72422E-05
S100a3	-1.8018	0.2893	19.8176	8.51934E-06	8.89884E-05
Syne1	-1.1313	5.8627	19.8089	8.55843E-06	8.92943E-05
Colec12	-1.0519	5.6188	19.8074	8.56499E-06	8.92943E-05
Pid1	-1.0962	3.5034	19.7799	8.68898E-06	9.03282E-05
Mpo	-1.6940	2.1883	19.7319	8.91035E-06	9.24533E-05
Lep	-1.2049	3.9110	19.7195	8.96802E-06	9.30075E-05
Gm5849	-2.5644	4.9097	19.7162	8.98344E-06	9.31232E-05
Ankrd23	-1.9383	8.1911	19.7018	9.0516E-06	9.36519E-05
Cacna1a	-1.3496	2.5264	19.6414	9.34247E-06	9.64328E-05
Dmd	-1.1841	5.5619	19.6207	9.44389E-06	9.72957E-05
Lrrc75a	-1.1286	2.0613	19.6190	9.45242E-06	9.73377E-05
Casc4	-1.0021	2.6758	19.5992	9.55109E-06	9.83073E-05
Flrt2	-1.0085	5.2999	19.5445	9.82832E-06	0.000100875
AI464131	-1.1326	5.5154	19.4422	1.03688E-05	0.000105727
Eno3	-1.4607	10.1683	19.4412	1.03744E-05	0.000105734

C1qa	-1.0621	6.1446	19.4363	1.0401E-05	0.000105907
Sptb	-1.3128	7.0643	19.4294	1.04388E-05	0.000106243
Abcc6	-2.9574	-0.9124	19.4088	1.05522E-05	0.000107197
Speg	-1.2473	6.4773	19.3809	1.0707E-05	0.000108516
Cldn3	-1.5202	2.0160	19.3639	1.08032E-05	0.000109333
Acta1	-1.4893	13.4449	19.3631	1.08076E-05	0.000109333
Ly6c2	-1.2012	3.1190	19.3255	1.10227E-05	0.000111149
Asb14	-1.2434	3.9551	19.3058	1.11368E-05	0.000112247
Jdp2	-1.1383	2.6524	19.2874	1.12443E-05	0.000113174
Cd300lb	-1.2530	2.3296	19.2866	1.12495E-05	0.000113174
Tnnc2	-1.5277	10.5132	19.2856	1.1255E-05	0.000113177
Sec31b	-1.3109	4.0531	19.2611	1.14006E-05	0.000114431
Cdkn1c	-1.2654	3.6121	19.2214	1.16399E-05	0.000116511
Dusp13	-1.3530	5.2148	19.1795	1.18983E-05	0.0001187
Eya1	-1.5878	2.7774	19.1685	1.19669E-05	0.000119238
Sgcg	-1.4219	5.4708	19.1427	1.21299E-05	0.000120586
Slc25a34	-1.1798	4.2949	19.1102	1.23383E-05	0.000122419
Ltc4s	-1.1253	3.0427	19.1096	1.23423E-05	0.000122419
Sctr	-1.2993	1.3819	19.0720	1.25878E-05	0.000124458
Frzb	-1.4262	2.6454	19.0707	1.25963E-05	0.000124486
Slc6a2	-1.1988	3.9615	19.0055	1.30341E-05	0.000128348
Pdk2	-1.2625	7.5542	18.9809	1.32031E-05	0.000129895
Cacna2d1	-1.3617	6.3706	18.8820	1.3906E-05	0.000135953
Dusp27	-1.4155	4.0241	18.8480	1.41558E-05	0.000137839
Fmo2	-1.0129	5.5583	18.7809	1.46632E-05	0.000142082
Krt31	-5.0619	-1.5117	18.7789	1.46782E-05	0.000142165
Fitm1	-1.3686	5.1782	18.7436	1.49521E-05	0.000144433
Zfp189	-1.1184	1.4459	18.7394	1.49851E-05	0.000144688
Kcnj11	-1.2776	5.2055	18.6736	1.55113E-05	0.000149048
Slc15a2	-2.1587	0.8254	18.6466	1.57325E-05	0.00015097
Slc16a7	-1.0333	2.4658	18.5691	1.63857E-05	0.000156356
Rbm20	-1.2358	1.6447	18.5240	1.67778E-05	0.000159667
Vit	-1.4867	3.3101	18.5073	1.69254E-05	0.000160932
Scn4a	-1.2746	6.8239	18.4999	1.69917E-05	0.000161443
Grin2d	-1.0157	2.4048	18.4937	1.70469E-05	0.000161875
Nexn	-1.3147	5.7056	18.4898	1.70819E-05	0.000162137
Bin1	-1.0894	7.1087	18.4560	1.73868E-05	0.000164459
Cd209g	-1.2107	3.7072	18.4196	1.77225E-05	0.000167344
Srpx	-1.0296	3.8761	18.3931	1.79708E-05	0.000169126
Ctgf	-1.0734	5.4829	18.3880	1.80194E-05	0.000169488
Abca5	-1.3379	6.5169	18.3850	1.80477E-05	0.000169608
Ifi207	-1.1283	5.0293	18.3608	1.82782E-05	0.000171553
Fez1	-1.3952	3.9336	18.3492	1.839E-05	0.000172306
Col4a5	-1.1917	4.3325	18.3138	1.87343E-05	0.000175156
Ckm	-1.6536	11.7717	18.3005	1.88661E-05	0.000176162
Zfp57	-1.1376	2.1835	18.2380	1.94947E-05	0.000181487
Schip1	-1.0501	4.7850	18.2260	1.96182E-05	0.000182447
Ucp3	-1.3281	5.6156	18.2202	1.96785E-05	0.000182809
Nkd1	-1.0882	1.8756	18.2164	1.97175E-05	0.000183036
Phka1	-1.5238	6.7140	18.2162	1.97196E-05	0.000183036
Capn3	-1.2965	5.6290	18.2087	1.97972E-05	0.000183677
Tbx18	-1.4368	1.2778	18.2070	1.98147E-05	0.000183761
Siglec1	-1.5163	4.7096	18.1910	1.99822E-05	0.000185237
Adamts14	-1.1391	5.1570	18.1771	2.01283E-05	0.000186179
Cpt1b	-1.1931	7.0051	18.1766	2.01341E-05	0.000186179
Slc11a1	-1.0622	2.2915	18.1690	2.0214E-05	0.000186456
Bpgm	-1.0438	4.8135	18.1688	2.02162E-05	0.000186456
Pdlim7	-1.2609	6.1154	18.1656	2.02501E-05	0.000186611

Tnnt3	-1.4935	11.5966	18.1298	2.06348E-05	0.000189755
Asphd2	-2.8365	-0.6419	18.1105	2.08444E-05	0.000191521
Fgfr4	-1.1577	1.5264	18.0336	2.17042E-05	0.000198585
Akap12	-1.6273	4.3383	18.0098	2.19771E-05	0.000200662
Rspo1	-1.3583	1.4049	18.0060	2.20215E-05	0.000200899
Ifi204	-1.1119	4.3940	17.9979	2.21144E-05	0.000201662
Galnt9	-1.7433	0.0813	17.9708	2.24314E-05	0.000204383
Tpm1	-1.3629	10.6434	17.9664	2.24838E-05	0.000204774
Chrnb1	-1.2942	3.3920	17.9651	2.24989E-05	0.000204827
Mapt	-1.0057	4.1364	17.9609	2.25494E-05	0.0002052
Eid2	-1.1429	1.5800	17.9593	2.25677E-05	0.000205281
Atp2a1	-1.6133	13.0405	17.9012	2.32673E-05	0.000210592
Skint11	-1.1682	2.9846	17.8815	2.351E-05	0.000212525
Syngr1	-1.2665	4.3780	17.8804	2.35233E-05	0.000212557
Jsrp1	-1.3113	6.1687	17.8329	2.41176E-05	0.000217567
Fndc4	-1.1290	1.8337	17.8321	2.41276E-05	0.000217567
Ddo	-1.3656	2.8625	17.8183	2.4304E-05	0.000218759
Bank1	-2.3288	-0.0150	17.8146	2.43508E-05	0.000219037
Xk	-1.6263	1.4221	17.8075	2.44418E-05	0.000219584
Rapsn	-1.2134	2.9638	17.7971	2.4576E-05	0.000220644
Pknox2	-1.1647	3.3216	17.7968	2.458E-05	0.000220644
Fam131b	-1.7011	1.3603	17.7660	2.49806E-05	0.000223872
Rbfox1	-1.5895	5.6640	17.7481	2.52164E-05	0.000225522
Aph1c	-1.0742	2.6709	17.7377	2.53558E-05	0.000226676
Creb5	-1.1871	3.8887	17.7171	2.56307E-05	0.000228759
Omd	-1.8341	2.5369	17.7013	2.58451E-05	0.00023039
Mlip	-1.1743	5.2568	17.6547	2.64864E-05	0.000235625
Dusp10	-1.2497	3.1111	17.6521	2.65223E-05	0.000235753
Runx1t1	-1.5345	1.0304	17.6387	2.67097E-05	0.000237129
Loxl1	-1.0469	5.8780	17.5686	2.77124E-05	0.000245332
Rorb	-2.0300	-0.2678	17.5582	2.7865E-05	0.000246384
Trim55	-1.3692	3.6878	17.5414	2.81123E-05	0.000248151
Lmcd1	-1.2463	6.2779	17.5314	2.82596E-05	0.000249065
Fsd2	-1.3701	5.3893	17.4491	2.95102E-05	0.00025925
Paqr7	-1.2372	5.2019	17.4154	3.00377E-05	0.00026346
Arsi	-1.2626	2.8917	17.4008	3.02698E-05	0.000264858
Lrrc2	-1.2389	5.5835	17.3922	3.04076E-05	0.000265957
Trim66	-1.5333	0.7803	17.3705	3.07558E-05	0.00026868
Trim2	-1.4197	6.5101	17.3695	3.07718E-05	0.000268712
Ano5	-1.3302	4.8511	17.3567	3.09808E-05	0.000270201
Slamf9	-1.7806	1.6009	17.3270	3.14686E-05	0.000273484
Per1	-1.0048	6.7026	17.3099	3.17524E-05	0.000275315
Ccer2	-1.4181	1.9659	17.3005	3.19095E-05	0.000276435
Tpm2	-1.2364	11.2896	17.2942	3.20165E-05	0.000276923
Adamtsl4	-1.1345	5.7990	17.2741	3.23567E-05	0.000279533
B3galt1	-1.2552	1.7401	17.2008	3.36299E-05	0.000289387
Tcap	-1.4390	9.7204	17.1935	3.3759E-05	0.000290139
Ppp1r3a	-1.5246	6.2905	17.1933	3.37617E-05	0.000290139
Tcea3	-1.3489	5.5616	17.1353	3.48099E-05	0.00029813
Cap2	-1.2454	5.8146	17.0957	3.55432E-05	0.000303696
Cacna1s	-1.4087	7.9953	17.0823	3.57953E-05	0.000305491
Csprs	-1.0237	3.2732	17.0773	3.58896E-05	0.000306177
Plin5	-1.1112	3.0400	17.0669	3.6086E-05	0.000307612
Eepd1	-1.1368	5.5252	17.0660	3.61028E-05	0.000307635
Plekhf1	-1.0616	3.4794	17.0077	3.72277E-05	0.000316303
Gng7	-1.0125	1.6789	17.0032	3.7316E-05	0.00031686
Pde4a	-1.1988	3.3874	17.0015	3.73502E-05	0.000317027
Fabp3	-1.1910	6.8458	16.9977	3.74261E-05	0.000317547

Gm973	-1.2350	2.3404	16.9895	3.75869E-05	0.000318469
Nr4a1	-1.0264	6.4295	16.9748	3.78798E-05	0.000320649
Trpc1	-1.4847	1.2311	16.9574	3.82282E-05	0.000323347
Fndc5	-1.3160	3.5107	16.9465	3.84483E-05	0.000324706
Mpz	-1.0911	5.1513	16.9436	3.85072E-05	0.000324828
C1qtnf7	-1.3651	2.5290	16.9291	3.8802E-05	0.000326556
St3gal1	-1.0404	5.0709	16.8991	3.94198E-05	0.000330863
Cd82	-1.1590	7.0410	16.8249	4.09919E-05	0.000343266
Tmem182	-1.2261	6.2465	16.8165	4.11728E-05	0.000344502
Wnk2	-1.0002	6.5743	16.7969	4.16014E-05	0.000347426
Cdc14a	-1.0923	2.7841	16.7413	4.28382E-05	0.000356675
Ank1	-1.4801	5.2672	16.7250	4.32079E-05	0.000359478
Vipr1	-1.6472	3.7632	16.7105	4.35395E-05	0.000362099
Hist1h4h	-1.2601	2.0309	16.7071	4.36172E-05	0.000362608
Srpk3	-1.5623	2.4442	16.5927	4.63296E-05	0.000383405
Fam131a	-1.1551	3.2065	16.5429	4.75629E-05	0.000392867
Ttn	-1.3668	11.5904	16.5223	4.80803E-05	0.000396689
Cox7a1	-1.3068	4.9529	16.4329	5.0402E-05	0.000413501
Hhatl	-1.4413	5.5671	16.3647	5.22505E-05	0.000427381
Fam169b	-1.2028	1.4109	16.3444	5.28125E-05	0.000431009
Ctxn3	-1.4012	4.1136	16.3431	5.28488E-05	0.000431145
Vsig4	-1.8173	0.8539	16.3237	5.33909E-05	0.00043443
Slc43a3	-1.0620	5.7739	16.3173	5.35714E-05	0.000435032
Edn3	-1.9545	-0.1419	16.3167	5.35902E-05	0.000435032
Shisa4	-1.2762	5.0779	16.3088	5.38147E-05	0.000436577
Tmem28	-2.3013	0.5594	16.2932	5.42587E-05	0.000439689
Gnao1	-1.0677	2.6572	16.2787	5.46738E-05	0.000442829
Fgf7	-1.1825	2.7298	16.2783	5.46873E-05	0.000442829
Lrrc3b	-1.7718	0.7666	16.2776	5.4707E-05	0.000442829
Tmem233	-1.5548	5.0389	16.2618	5.51664E-05	0.000445886
Zfp775	-1.0330	2.7693	16.2447	5.56663E-05	0.000449261
Hrc	-1.2409	7.8880	16.2357	5.5931E-05	0.000450897
Hs3st5	-1.7210	0.7502	16.2248	5.62515E-05	0.00045298
Cfh	-1.0810	6.1949	16.1624	5.81375E-05	0.000466275
Ms4a8a	-1.9111	1.4849	16.1584	5.82594E-05	0.000467081
Smyd1	-1.1869	6.6012	16.1022	6.00154E-05	0.000479224
Zfp449	-1.1242	1.5283	16.0941	6.02706E-05	0.000481085
Obscn	-1.2962	9.4600	16.0890	6.04335E-05	0.000482033
Sh2d7	-1.2464	0.8799	16.0822	6.06515E-05	0.000483595
Mypn	-1.3223	7.1008	16.0551	6.15247E-05	0.000489663
Cacng6	-1.3777	4.0323	16.0215	6.26265E-05	0.000497164
E2f6	-1.1843	3.6653	16.0112	6.29674E-05	0.000499185
Klhl41	-1.2350	8.4779	16.0039	6.32118E-05	0.000500899
Neurl1a	-1.2302	5.4478	15.9816	6.39598E-05	0.00050636
Smarcd3	-1.5737	4.6922	15.9498	6.50446E-05	0.000514489
Retnla	-3.0062	7.8754	15.9375	6.54675E-05	0.000516897
Tnfrsf14	-1.1206	1.6874	15.8911	6.70952E-05	0.00052822
Lmntd1	-2.8441	-0.3462	15.8802	6.74809E-05	0.000530683
Ryr1	-1.3956	10.1521	15.8754	6.76519E-05	0.000531836
Pnmal2	-1.5838	0.4469	15.8566	6.8328E-05	0.000536379
Reep1	-1.1163	4.2118	15.8404	6.89148E-05	0.000540402
Dusp23	-1.0721	3.5489	15.8341	6.91433E-05	0.000541805
Enkur	-1.7386	0.0358	15.8265	6.94225E-05	0.000543602
Scn4b	-1.1571	6.5448	15.8189	6.97042E-05	0.000545294
Plekhb1	-1.4942	2.8936	15.8059	7.01838E-05	0.00054858
Mef2c	-1.0834	6.5717	15.7623	7.18182E-05	0.000559352
Rasd2	-1.2940	2.0933	15.7220	7.3367E-05	0.000569742
Sema6c	-1.0847	4.3093	15.7148	7.36469E-05	0.000571555

Sspn	-1.0123	5.4395	15.7134	7.36988E-05	0.000571755
Cystm1	-1.1684	4.8308	15.7012	7.41785E-05	0.00057466
Cd5l	-2.8118	1.0212	15.6845	7.48345E-05	0.000579536
Agl	-1.2883	7.8652	15.6159	7.75987E-05	0.000598304
Pde4d	-1.1552	5.3463	15.6155	7.76142E-05	0.000598304
Rbm38	-1.0647	5.4138	15.6124	7.77437E-05	0.000598879
Katnal1	-1.0363	2.1836	15.5418	8.06995E-05	0.00061968
Slc24a3	-1.6390	2.7432	15.5387	8.08338E-05	0.000620275
Krt15	-1.0095	9.0549	15.5369	8.09095E-05	0.000620638
Mylpf	-1.4444	10.6427	15.5040	8.23324E-05	0.000629782
Ifi211	-1.0611	3.2291	15.4452	8.4932E-05	0.000646271
Pgm2	-1.1393	7.6160	15.4429	8.5036E-05	0.00064644
Myom2	-1.1578	9.0926	15.4008	8.69497E-05	0.000658411
Des	-1.1629	10.6858	15.3313	9.02076E-05	0.000680484
Dhrs7c	-1.4269	5.1566	15.3070	9.13784E-05	0.000688603
Scrn1	-1.1559	2.6009	15.2903	9.2191E-05	0.000693863
Fgf18	-1.4455	1.0789	15.2778	9.27995E-05	0.000697867
Smarca1	-1.3809	1.8638	15.2324	9.50575E-05	0.00071408
Tmem100	-1.5895	5.1634	15.1758	9.79533E-05	0.000733088
Casr	-1.5462	3.7870	15.1689	9.83119E-05	0.000735445
Atp1b2	-1.4217	6.2744	15.1684	9.83356E-05	0.000735445
Golga7b	-1.2098	3.1252	15.1600	9.87727E-05	0.000738461
Cfl2	-1.0549	6.2029	15.1361	0.000100032	0.000745397
Armcx4	-1.1000	2.9806	15.1272	0.000100507	0.000748609
Gria3	-4.8444	-0.5129	15.1099	0.000101432	0.00075395
Pm20d2	-1.1557	2.1293	15.0961	0.000102172	0.000758161
Rnf32	-2.9851	-1.2104	15.0480	0.000104809	0.000775361
Tmod1	-1.0316	5.6869	15.0416	0.000105169	0.000777414
Amy1	-1.1189	4.4501	15.0216	0.000106287	0.000783899
Hlf	-1.0955	5.4020	15.0020	0.000107396	0.000791013
Lbx1	-1.5200	2.9461	14.9832	0.000108471	0.000798122
Gm35315	-6.5891	-0.4331	14.9755	0.000108914	0.000801114
Xirp2	-1.3039	8.6344	14.9441	0.000110743	0.000813476
Tmem38a	-1.2243	8.2436	14.9375	0.000111134	0.00081607
Slc8a3	-1.3946	4.9446	14.8995	0.000113393	0.000830003
Gm20431	-8.6488	1.1265	14.8913	0.000113885	0.000833187
Oxtr	-1.4509	3.0730	14.8793	0.000114614	0.000837954
Lmod1	-1.2911	5.9809	14.8660	0.000115425	0.000843324
Tbx4	-1.4095	0.8325	14.8582	0.000115901	0.00084623
Cav3	-1.0483	3.9438	14.8450	0.000116716	0.000851048
Pdlim3	-1.3104	7.6933	14.8040	0.000119283	0.000865482
Btbd8	-1.0717	1.9993	14.8032	0.00011933	0.000865482
Slc9a4	-3.0141	-1.0343	14.7779	0.000120944	0.000874858
Stard9	-1.0720	3.5850	14.7665	0.000121678	0.000879006
Gm7276	-2.7330	-0.7991	14.7420	0.000123273	0.000888113
Jph2	-1.4816	6.5766	14.7308	0.000124002	0.000892842
D2Bwg1423e	-1.2883	0.4409	14.7195	0.000124748	0.000897026
Mybpc2	-1.7008	10.3349	14.7117	0.000125266	0.000899566
Fhl3	-1.0930	7.1330	14.7098	0.000125396	0.000900201
Sgca	-1.1891	6.1171	14.6947	0.000126399	0.000907104
AI854703	-1.0498	1.3517	14.6846	0.000127081	0.000911103
Cacng4	-2.0282	-0.6685	14.6601	0.000128742	0.0009215
Myod1	-2.0414	1.7384	14.6480	0.000129573	0.000927144
Tll1	-1.0210	3.0152	14.6466	0.000129666	0.000927504
Slc2a4	-1.0730	6.9373	14.6357	0.000130418	0.000931048
Lrrc74b	-1.1141	1.6945	14.6095	0.000132243	0.000942846
1700019D03Rik	-1.4500	2.6599	14.6077	0.000132375	0.000943476
Cd46	-1.0901	2.5243	14.6017	0.000132794	0.000946159

Lsamp	-1.6551	1.5046	14.5882	0.000133747	0.000952323
Akr1c19	-1.9322	0.1601	14.4918	0.000140771	0.000994554
Wasf3	-1.4472	0.5914	14.4635	0.000142905	0.001007024
Tcea2	-1.2478	0.9975	14.4589	0.000143255	0.001009061
Gm16253	-1.3410	0.5530	14.4584	0.000143287	0.001009061
Otof	-2.8535	-1.0028	14.4485	0.000144042	0.001013
Ldb3	-1.0404	8.6390	14.4481	0.000144078	0.001013
Dnajc6	-1.5611	1.8035	14.4461	0.000144231	0.001013498
Glrb	-2.1252	0.3682	14.3926	0.000148384	0.001038924
Dact2	-1.2325	2.6714	14.3721	0.000150009	0.001048621
St3gal5	-1.0695	3.2132	14.3683	0.000150311	0.001050398
Cacnb1	-1.0869	5.8714	14.3481	0.000151936	0.001060392
Sel1l3	-1.0226	3.5035	14.3412	0.000152493	0.001063601
Cyb5r2	-1.3061	2.3058	14.3268	0.000153663	0.00106971
Slc4a10	-2.8141	-0.1472	14.3240	0.000153893	0.001070972
Stum	-1.1087	1.9151	14.3134	0.000154763	0.001076339
Rxfp1	-1.4723	1.2314	14.2939	0.000156374	0.001085815
Abcb1a	-1.0433	2.2375	14.2617	0.00015907	0.001102083
Srl	-1.2100	8.4604	14.2559	0.000159563	0.001105144
Tmcc2	-1.3393	3.6203	14.2525	0.000159851	0.001106085
Nrap	-1.2606	9.0902	14.1475	0.000169019	0.001160698
Zfp651	-1.1423	4.3600	14.1159	0.000171886	0.00117779
Necab3	-1.9020	-0.1479	14.1108	0.000172354	0.001180261
Kcnc1	-1.2681	4.7560	14.0951	0.000173798	0.001188652
Stac3	-1.2680	5.4250	14.0707	0.000176064	0.001201517
Riiad1	-2.4051	0.2588	14.0700	0.000176129	0.001201586
Ppp1r27	-1.2793	3.4516	14.0405	0.000178918	0.00121757
Mrc1	-1.0453	6.6089	13.9817	0.000184597	0.001249824
Cryaa	-1.5715	0.4715	13.9389	0.000188854	0.001273783
Diras2	-1.1724	1.0445	13.8556	0.000197406	0.001325209
Rnf165	-1.0586	1.5437	13.8541	0.000197561	0.001325838
Wfdc1	-1.0637	3.2820	13.8487	0.000198134	0.001328461
Mss51	-1.6531	3.2998	13.8354	0.000199542	0.001337082
Calr3	-1.0664	1.0166	13.8277	0.000200364	0.001342142
Vnn1	-1.2512	1.7276	13.8237	0.000200782	0.00134374
Myot	-1.1672	8.2069	13.8108	0.000202169	0.001351365
Ppp1r1a	-1.4379	4.2876	13.7872	0.000204722	0.00136759
Zfp977	-2.9663	-1.2438	13.7765	0.000205891	0.00137414
Gm996	-1.9242	-0.2897	13.7704	0.000206565	0.001378216
Fgf9	-1.4931	0.5152	13.7660	0.000207051	0.001379773
Rab3b	-2.1716	-0.2196	13.7060	0.000213768	0.001418042
Atp1a3	-1.1583	1.7569	13.6942	0.000215116	0.001425248
Ifi205	-1.0507	4.5528	13.6082	0.000225199	0.001486192
Fgf2	-1.1203	3.9064	13.6055	0.000225522	0.001487875
Neb	-1.2433	10.0059	13.6022	0.000225919	0.001490045
Adra1b	-2.2216	-0.9146	13.5711	0.000229689	0.001510798
9330159F19Rik	-1.0842	4.1697	13.5242	0.000235505	0.001544862
Rnf150	-1.1248	4.5032	13.4992	0.000238662	0.001564636
Krt71	-5.2498	1.2560	13.4949	0.000239218	0.001567805
Ttll11	-1.8703	0.1462	13.4523	0.000244707	0.001600898
Plpp7	-1.4056	4.1359	13.4192	0.000249059	0.001623045
Mertk	-1.2190	2.7812	13.4076	0.000250602	0.001632125
Myl1	-1.3689	10.5003	13.3573	0.000257418	0.001672029
Nr3c2	-1.1710	2.6907	13.2999	0.000265419	0.001719642
Mill2	-1.3156	1.5007	13.2820	0.000267971	0.001733356
Abca4	-1.2191	0.6029	13.2766	0.000268737	0.001737278
Rgs11	-3.0450	-0.5838	13.2733	0.000269208	0.001739812
Filip1	-1.4965	4.1805	13.2671	0.0002701	0.001744029

Rhebl1	-1.0004	1.5846	13.2622	0.000270815	0.001747994
Jam3	-1.0001	2.1615	13.2618	0.000270875	0.001747994
Ppp1r9a	-1.0521	2.0503	13.2154	0.000277665	0.00178847
Itm2a	-1.2276	6.4820	13.2057	0.000279102	0.001795249
Kcnj8	-1.0515	2.6401	13.1633	0.000285483	0.001830902
Wscd1	-1.3544	0.2048	13.1601	0.000285974	0.001832435
Hist1h2be	-1.5382	0.3141	13.1328	0.000290171	0.001856057
Prss36	-1.0504	1.5371	13.1127	0.000293303	0.001873893
Nr1h4	-2.2732	-0.8905	13.1023	0.00029494	0.001882148
Fam110b	-1.2720	1.0570	13.0281	0.000306847	0.001944496
Gm3839	-1.1985	1.0253	13.0174	0.00030861	0.00195453
Zc4h2	-1.2473	0.5310	13.0112	0.000309626	0.00195926
Slc13a4	-1.3149	1.0270	12.9999	0.000311515	0.001969502
Pdzd7	-1.3558	1.3546	12.9858	0.000313856	0.001983151
Dbp	-1.0430	5.1362	12.9794	0.000314936	0.001989401
Pvalb	-1.9696	9.9050	12.9676	0.000316921	0.001999048
Myadml2	-1.3958	1.7859	12.9581	0.000318543	0.002008115
Egf	-1.2133	4.1414	12.9568	0.000318763	0.002008927
Frs3	-1.3951	1.5337	12.9534	0.000319343	0.002011999
Pgm2l1	-1.0314	3.4990	12.9491	0.000320075	0.00201603
P4htm	-1.3660	2.4920	12.9394	0.000321732	0.002023443
Klhl40	-1.2851	5.8474	12.9242	0.000324366	0.002036764
Zfp354c	-1.0992	1.8285	12.8629	0.00033516	0.002096525
Bco1	-1.1163	1.3985	12.8381	0.000339637	0.002119717
Bves	-1.2664	3.9013	12.8380	0.000339644	0.002119717
Actn3	-1.9024	10.1814	12.8289	0.000341307	0.002128881
Trdn	-1.2880	7.6140	12.7728	0.000351696	0.002186182
Ptgds	-1.4990	0.5438	12.7630	0.00035354	0.002196393
Ednrb	-1.1005	4.7909	12.7415	0.000357623	0.002218359
Ampd1	-1.6742	7.0105	12.6769	0.000370197	0.002285571
Cacna1g	-1.1812	2.3033	12.6763	0.000370328	0.00228573
Myog	-1.5309	1.9666	12.6589	0.00037379	0.002304491
Gm45808	-5.0946	-1.2806	12.6244	0.00038074	0.002343373
Siglech	-1.5979	1.1607	12.5902	0.000387778	0.002376637
Pik3ap1	-1.0904	1.9027	12.5521	0.000395758	0.002419043
Alpk2	-1.0025	4.6843	12.4116	0.000426671	0.002586687
Tceal1	-1.3655	1.3624	12.4094	0.000427189	0.002589108
Mndal	-2.3162	0.3629	12.3680	0.000436766	0.00263982
Brsk1	-1.2245	0.8956	12.3615	0.000438288	0.002647557
Adra1a	-1.2533	0.7714	12.3396	0.00044344	0.002675717
Scx	-1.0546	1.8009	12.3147	0.000449394	0.002706404
Jph1	-1.2365	6.0095	12.2702	0.000460246	0.002763377
Tlr11	-1.7048	-0.0854	12.2137	0.00047439	0.002840484
Tekt1	-2.6921	-0.8532	12.1955	0.000479041	0.002863618
En1	-1.2436	0.7178	12.1824	0.000482415	0.002880637
Sult2a7	-3.3131	-1.5042	12.1491	0.00049111	0.002924554
Pacrg	-1.4493	0.1360	12.1092	0.00050175	0.00297655
Morn4	-1.0525	1.0659	12.1023	0.0005036	0.002985089
Abra	-1.4380	6.1185	12.0222	0.000525707	0.003099299
Zfp536	-1.7855	-0.5927	12.0194	0.000526503	0.003103157
Smtnl1	-1.0561	5.3500	11.9778	0.000538391	0.003164678
Mrgprb3	-1.6666	3.4232	11.9694	0.000540804	0.003174588
Prokr1	-1.5786	0.1175	11.9547	0.000545096	0.003197203
Prkag3	-1.2338	1.9729	11.9431	0.000548516	0.003213809
3425401B19Rik	-1.0152	7.2125	11.9336	0.000551303	0.003227553
Gabre	-1.0477	1.4258	11.9336	0.000551305	0.003227553
Lrch2	-2.5104	-0.9271	11.8705	0.000570287	0.003327081
Aspa	-1.0287	2.1037	11.8317	0.000582323	0.003390054

Krt28	-2.3233	-0.4894	11.8223	0.000585269	0.003402663
Wnt11	-1.2879	1.4117	11.8200	0.000585964	0.003405797
Cep85l	-1.2476	4.9492	11.8076	0.000589879	0.003422179
Smpx	-1.1902	6.5322	11.7874	0.000596343	0.003456923
Padi2	-1.5699	4.5373	11.7823	0.000597962	0.00346355
Synpo2l	-1.1685	4.7913	11.7433	0.000610637	0.003526672
Gm2115	-2.5584	1.3290	11.7329	0.000614049	0.00353982
Fam196b	-2.5851	-1.1568	11.6786	0.000632231	0.003635037
Zfp641	-1.1647	3.1241	11.6613	0.000638153	0.003661371
Tmtc1	-1.1336	4.4784	11.6372	0.000646473	0.003702293
Myom1	-1.1081	8.9329	11.6318	0.000648347	0.003709138
Batf3	-1.7954	-0.5977	11.6272	0.000649955	0.003717362
Cldn10	-1.4666	0.6129	11.6096	0.000656127	0.003748732
Habp2	-1.1654	3.1631	11.5956	0.000661096	0.003774159
Enho	-1.4441	1.9088	11.5850	0.000664849	0.003791966
Sema3d	-1.2246	6.6145	11.5711	0.000669835	0.003817061
Slc6a15	-2.7203	-1.3792	11.4938	0.000698284	0.003962632
Gm10134	-1.8715	-0.6903	11.4636	0.000709723	0.004019186
Haao	-1.5029	-0.0026	11.4520	0.000714152	0.004041123
Myoc	-1.1726	5.1699	11.4374	0.000719818	0.004067911
Casq1	-1.5239	8.6976	11.4245	0.000724807	0.00409293
Spns3	-1.5034	0.3201	11.4064	0.00073192	0.004127756
Krt25	-6.3457	0.7204	11.3993	0.000734722	0.00414142
Kcna1	-1.1293	0.9458	11.3674	0.000747439	0.004200086
Arhgef9	-1.0449	2.2949	11.3492	0.000754789	0.004232673
Krt12	-1.5603	0.4301	11.3374	0.000759623	0.004251045
Wnt5b	-1.2900	1.7041	11.3021	0.000774183	0.004326982
Dand5	-1.0202	1.5746	11.2990	0.000775471	0.004333073
Ppm1j	-1.1057	2.9741	11.2651	0.000789793	0.004398463
Pitx3	-1.1539	0.6194	11.2548	0.000794161	0.004419405
Nfe2	-1.7991	0.0776	11.2064	0.000815167	0.004520241
Inmt	-1.6653	5.0360	11.2031	0.000816625	0.004527108
Egfl6	-1.0127	1.7698	11.1551	0.000837998	0.004625623
Hist1h3i	-4.9514	-1.6174	11.1451	0.000842556	0.004646082
Fer1l6	-1.7303	-0.1387	11.0533	0.000885277	0.00485986
Cers1	-1.2790	2.0070	10.9602	0.000930921	0.005080731
Myl4	-1.0428	1.3151	10.9444	0.000938883	0.005117794
Th	-1.3721	1.1225	10.9181	0.000952299	0.005177991
Gpd1	-1.0413	7.0073	10.8849	0.000969538	0.005262552
Bex1	-1.3510	1.0601	10.8368	0.000995015	0.005380774
Ccdc157	-1.0748	2.5964	10.8100	0.001009515	0.005449734
Flg2	-1.0544	12.2118	10.7840	0.001023785	0.005514059
Lgi3	-1.2297	0.5264	10.7746	0.00102904	0.00553461
Ccrl2	-1.1206	1.8842	10.7586	0.001037961	0.005577094
Ddit4l	-1.5254	5.8895	10.7478	0.001044008	0.005605442
C6	-1.2030	2.3869	10.7228	0.001058239	0.005669306
Vgll2	-1.0127	4.0249	10.7057	0.001068086	0.005715047
Pls1	-1.2418	0.4988	10.6953	0.001074101	0.005743006
Osbpl6	-1.1419	2.2703	10.6929	0.001075466	0.005746086
Asb4	-1.5314	0.7369	10.6900	0.001077189	0.005753879
Hist1h1e	-1.3681	1.5084	10.6771	0.001084708	0.005786963
Adamts16	-2.0204	-0.8638	10.6730	0.001087087	0.005795409
Cdh23	-2.5450	-0.3936	10.6501	0.001100674	0.005853549
Chadl	-2.2694	-0.9167	10.6323	0.001111314	0.005901507
Fam19a5	-1.1478	1.2765	10.6027	0.001129196	0.005983369
Nxpe3	-1.0541	1.3379	10.6000	0.001130884	0.005986498
Dpep3	-1.8779	0.1917	10.5664	0.001151597	0.006082873
Smim18	-3.2122	-1.5535	10.5452	0.001164904	0.006144244

Gpr173	-1.5571	-0.3896	10.5399	0.001168238	0.006154393
Cfap57	-1.2817	5.8843	10.5228	0.001179096	0.00620112
Agbl1	-1.2074	3.1564	10.5152	0.001183946	0.00622213
Emilin3	-2.3799	-1.2648	10.5014	0.001192836	0.006258302
Hspb2	-1.1377	3.1469	10.5009	0.001193144	0.006258418
Cyp2w1	-1.1727	2.3221	10.4824	0.001205154	0.006313825
Asb12	-1.3715	3.6762	10.4269	0.001241917	0.006481544
Scd3	-1.0694	5.8692	10.4056	0.001256354	0.006541664
Qprt	-1.5000	0.5062	10.4044	0.001257155	0.006543479
Usp13	-1.0167	7.2747	10.3958	0.001263032	0.006568188
Cyr61	-1.0073	5.6546	10.3585	0.001288764	0.006686076
Ppfia2	-1.0445	1.9760	10.3560	0.001290569	0.006690669
Letm2	-1.1277	1.4627	10.3485	0.001295798	0.006714592
Sypl2	-1.2446	5.9340	10.3406	0.001301379	0.00673871
Slc12a5	-1.0325	1.2873	10.2962	0.00133307	0.006876712
Bdh2	-1.3593	0.6136	10.2949	0.00133399	0.006879836
Eml6	-1.1515	0.7859	10.2602	0.001359273	0.006995352
Clic5	-1.0972	5.5819	10.2078	0.001398475	0.007171742
Umad1	-1.1354	2.3256	10.1963	0.001407255	0.007211687
Orm2	-1.1459	0.5915	10.1785	0.0014209	0.007262856
Stab2	-1.6335	0.2184	10.1703	0.001427241	0.007290147
A730017C20Rik	-2.1376	0.3909	10.1641	0.001431997	0.007309312
Best3	-1.5979	0.9840	10.1548	0.001439241	0.007341137
Krt35	-4.3499	-1.8341	10.1214	0.001465564	0.007449295
1500015O10Rik	-2.5315	-0.5162	10.1164	0.001469579	0.007467964
Rxrg	-1.0610	2.5631	10.0924	0.001488859	0.007557144
Pifo	-4.1213	-1.9437	10.0851	0.001494734	0.007585203
1810010H24Rik	-1.3757	0.7039	10.0524	0.001521531	0.007708643
Nnt	-1.5883	4.1528	10.0418	0.001530294	0.007745851
Clec12a	-1.8284	0.8878	10.0407	0.001531181	0.007748542
Gipc2	-1.2398	1.0249	10.0360	0.001535121	0.007763084
Myo18b	-1.1405	6.5213	10.0129	0.001554494	0.007851164
Olfr1315-ps1	-4.1375	-1.9339	9.9813	0.001581418	0.007967676
Fgd2	-1.4752	2.2285	9.9578	0.001601692	0.008049386
Popdc2	-1.3398	3.1244	9.9296	0.001626458	0.008153207
Ror2	-1.0186	1.1113	9.8567	0.00169217	0.008422654
Krt2	-1.4673	1.5234	9.8264	0.001720204	0.008539443
Gm10226	-4.2779	-1.8857	9.8263	0.001720327	0.008539443
Olfr1505	-1.5545	1.1629	9.8086	0.001737016	0.008614447
Lmx1a	-1.2210	0.3314	9.7771	0.001767017	0.008745353
6430571L13Rik	-1.6399	1.9253	9.7636	0.001780027	0.008797775
Nat8f3	-1.1516	1.0066	9.7318	0.001811022	0.00892097
Fam161a	-1.0311	1.3405	9.7248	0.001817971	0.008948847
Klhl34	-1.2937	2.7424	9.7115	0.001831209	0.009001831
Greb1l	-1.0825	0.5164	9.6967	0.001845971	0.009064192
Rec8	-1.5429	0.9197	9.6704	0.001872613	0.009170261
Nim1k	-1.1783	0.7647	9.6664	0.001876707	0.009188247
Vstm2b	-2.2992	-1.3071	9.6607	0.001882481	0.009214451
Gpr17	-1.6919	0.7409	9.6145	0.00193047	0.00939877
Dmrt2	-1.1086	2.1836	9.5677	0.001980334	0.00961016
Phf11b	-1.4709	0.9314	9.5632	0.001985148	0.009630616
Msc	-1.6055	-0.4844	9.5113	0.002042117	0.009871888
Xlr4c	-1.6750	-0.3350	9.4820	0.002075005	0.010006503
Mpp3	-1.1596	1.5589	9.4764	0.002081348	0.010031539
Kcng4	-1.0835	1.0035	9.4510	0.002110364	0.010154588
Zfp600	-2.5480	-1.2058	9.4409	0.002121966	0.010208161
Krt87	-2.9413	-1.2413	9.4334	0.002130693	0.010243375
Tnfrsf13c	-1.3756	-0.0558	9.4105	0.002157455	0.010353792

Msln	-2.0944	0.1067	9.3980	0.002172206	0.010413138
Adgrv1	-1.3934	0.0349	9.3448	0.002236204	0.010666065
Matn4	-1.4224	0.1207	9.3295	0.002254991	0.010734562
Amdhd1	-1.1801	0.6387	9.3239	0.002261855	0.010753169
Vtcn1	-1.2893	0.1574	9.3089	0.002280391	0.01082244
Pde10a	-2.9683	1.1032	9.2854	0.002309882	0.010945741
1700113H08Rik	-2.4500	-1.0006	9.2377	0.002370766	0.011209917
Smad6	-1.0457	1.7605	9.2353	0.002373954	0.011217703
Kcnab1	-5.4003	-1.3081	9.2236	0.002389133	0.011279664
Serpinb6e	-1.6243	2.0657	9.2053	0.002413166	0.011380823
1810010D01Rik	-4.0033	-1.9858	9.1904	0.002432927	0.01146906
Kcnu1	-1.3228	1.1827	9.1707	0.002459165	0.011575255
Pfn4	-1.5665	-0.5167	9.1498	0.002487396	0.011682946
Cd226	-1.3903	-0.2839	9.1117	0.002539766	0.011902497
Gm765	-2.5786	0.0618	9.0946	0.002563662	0.011997277
Yipf7	-1.1699	2.9936	9.0934	0.002565404	0.01200286
Foxd3	-1.2944	-0.0161	9.0652	0.002605228	0.012171377
Hk3	-1.4108	1.5587	9.0522	0.002623831	0.01225259
Gm45208	-4.0095	-1.9814	9.0252	0.002662756	0.012405171
Sel1l2	-3.9664	-2.0006	9.0115	0.002682894	0.012485664
Pde8b	-1.0372	1.0917	9.0107	0.002684022	0.012488253
Uts2r	-2.0298	-0.8471	8.9516	0.002772268	0.012844096
Gm6592	-4.0947	-1.9471	8.9495	0.002775477	0.012855162
Rasl10a	-1.0069	2.5303	8.9426	0.002785893	0.012879886
Erbb4	-3.0543	-1.6327	8.9247	0.002813359	0.01297116
Lenep	-5.4088	-1.2912	8.8885	0.002869669	0.013197335
Olfr1	-4.1465	-1.9402	8.8794	0.00288404	0.013255046
Sv2c	-1.4753	1.1150	8.8774	0.002887225	0.013266894
Zbtb16	-1.0976	5.3424	8.8763	0.002889015	0.013269819
Zfp354b	-1.2159	0.0988	8.8751	0.002890785	0.013269819
Dleu7	-2.9333	-1.6875	8.8336	0.002957355	0.013518166
Grik5	-2.4957	2.2231	8.8310	0.002961545	0.013534171
Slc23a1	-1.0405	1.1762	8.8229	0.002974742	0.013580427
Slc22a12	-4.1305	-1.9583	8.8172	0.002984082	0.013617368
C77370	-2.0652	-0.8215	8.8106	0.002994886	0.013660961
Mpp4	-1.6389	-0.5015	8.7714	0.003059925	0.013931432
BC048679	-2.0508	-0.5505	8.7512	0.003094048	0.014065989
Fgf4	-3.9476	-2.0045	8.7006	0.003181077	0.014416874
C7	-1.1145	1.3200	8.6845	0.003209347	0.014523891
Gabrb2	-2.6440	-1.4088	8.6784	0.003219979	0.014556919
Fbxw10	-4.6879	-1.6502	8.6509	0.003268916	0.014717646
Adamts6	-1.4763	0.5706	8.6410	0.003286837	0.014776553
Dapl1	-1.1739	6.6359	8.6218	0.003321555	0.014914205
Trpm3	-1.4741	-0.2297	8.6080	0.003346953	0.014997398
4930404N11Rik	-1.0132	0.9415	8.6000	0.003361687	0.015051062
Slc9a2	-1.1390	2.1026	8.5769	0.003404616	0.015214164
Itgb1bp2	-1.0366	5.2567	8.5653	0.003426266	0.015277536
Mag	-1.3864	0.2664	8.5482	0.003458648	0.015393626
Gspt2	-1.3168	0.7337	8.5397	0.003474816	0.015449837
Catsper4	-1.2298	0.5753	8.5307	0.003492004	0.015513623
Trmo	-1.1402	0.5645	8.5010	0.003549532	0.015740367
A630033H20Rik	-1.2971	0.4649	8.4919	0.003567321	0.015803203
Ankdd1a	-1.0863	0.3913	8.4460	0.003658398	0.016180406
Mzb1	-1.4731	-0.5782	8.4411	0.003668355	0.016221158
Flnc	-1.2300	8.8856	8.4226	0.003705777	0.016360127
Tlr8	-1.0382	2.0635	8.4061	0.003739667	0.016479748
Myh4	-1.6525	13.4648	8.3988	0.003754667	0.016529166
Me3	-1.0545	1.2765	8.3851	0.003783126	0.016637677
Hoxa9	-1.3064	2.1059	8.3792	0.003795366	0.016688143

Nptx2	-1.2826	0.6236	8.3734	0.003807558	0.016725274
Col24a1	-1.0028	0.8826	8.3733	0.003807641	0.016725274
Hbb-bs	-1.1794	6.8014	8.3624	0.003830719	0.016809735
Ppp2r2b	-1.4245	-0.1041	8.3419	0.003874148	0.016962802
Adamts19	-1.4071	0.4127	8.3378	0.003882794	0.01699384
Thpo	-1.0750	1.5798	8.3067	0.003949879	0.01723562
F5	-1.0446	0.5876	8.2985	0.00396789	0.017307294
Gprasp2	-1.4556	0.1579	8.2940	0.003977565	0.017340306
Gm14085	-1.2953	0.9566	8.2882	0.003990401	0.017384641
Slc26a7	-1.7839	-0.2326	8.2650	0.004041716	0.017580135
1700028P14Rik	-2.0295	-0.8643	8.2508	0.004073489	0.017711279
Nap1l2	-2.4685	-1.2092	8.2170	0.004150008	0.017983093
Rgag4	-1.2414	1.1683	8.2044	0.004178972	0.018090647
Crip3	-1.3463	1.2792	8.1893	0.004213727	0.018219426
Rbm11	-1.4588	-0.6334	8.1872	0.004218645	0.018237077
3300002I08Rik	-3.8626	-2.0326	8.1689	0.0042614	0.018389134
Ccna1	-2.4860	-1.5030	8.1446	0.004319054	0.018612174
Olfr1150-ps1	-1.0315	0.3433	8.1351	0.0043416	0.01869457
Slc12a8	-1.3539	-0.3356	8.1287	0.004357113	0.018755457
Neto1	-1.8784	-0.5351	8.0970	0.004433975	0.019011411
Hrk	-4.0394	-1.9898	8.0896	0.004451908	0.01906426
Rgs1	-3.9152	-2.0226	8.0870	0.004458457	0.019084157
Gm11627	-2.1414	-1.3887	8.0867	0.004459177	0.019084157
Fgf16	-1.3153	0.1623	8.0862	0.004460281	0.01908514
Best1	-1.3758	0.1573	8.0777	0.004481437	0.019168152
Carmil3	-1.1448	0.7154	8.0666	0.004508902	0.019270527
Chrna10	-1.2318	1.6900	8.0395	0.004576914	0.019507732
Dupd1	-1.7112	1.4881	8.0286	0.004604343	0.019595928
Gm42417	-2.6449	-0.2288	8.0183	0.004630809	0.019695149
Pgr	-2.5096	-0.6846	8.0020	0.004672522	0.019841632
Cd300ld3	-4.7668	-1.7253	7.9608	0.004780158	0.020223958
Hbb-bt	-1.1191	2.0514	7.9426	0.004828512	0.020389021
Myoz3	-1.1209	3.4961	7.9373	0.004842693	0.020440996
Hba-a1	-1.0983	6.9425	7.9329	0.004854381	0.020478453
Cubn	-1.3094	-0.0013	7.9229	0.004881181	0.020575607
Agr3	-2.8491	-1.7252	7.9095	0.004917553	0.020700943
Nlrp1a	-1.4089	1.0526	7.9059	0.004927502	0.020734828
Prr15l	-1.3543	0.5251	7.9049	0.004929991	0.020741306
Pcnx2	-3.7918	-2.0570	7.8725	0.005019191	0.021055717
Naa11	-3.8196	-2.0555	7.8338	0.005127954	0.021437831
Penk	-1.0250	3.7539	7.7792	0.005285078	0.022023007
Luzp2	-2.4276	-1.5281	7.7782	0.005288057	0.022031215
Ptx3	-1.3998	0.7927	7.7541	0.005359117	0.022288985
Espnl	-2.1097	-0.9930	7.7250	0.005446196	0.022608081
Gm13285	-1.0018	0.5406	7.7184	0.005465984	0.022672001
Amhr2	-1.0318	0.9515	7.6868	0.005562684	0.023021602
Krt33a	-3.7877	-2.0614	7.6699	0.005615078	0.023181311
A1bg	-2.1840	-1.1569	7.6695	0.005616245	0.023181744
Tbx5	-1.6167	-0.0982	7.6622	0.00563906	0.023267118
Irx6	-2.3733	-1.5497	7.6579	0.005652339	0.023304291
Gbgt1	-1.2050	0.8601	7.6527	0.005668863	0.023354773
Defb13	-4.1834	-1.8942	7.6520	0.005671053	0.023359388
Ttc9b	-1.1341	1.0274	7.6496	0.005678394	0.023385212
Gm4951	-1.1698	1.3072	7.6241	0.005759307	0.023642616
Spink2	-1.9922	-0.6045	7.6174	0.005780763	0.023712861
Shc2	-1.0206	2.3980	7.5746	0.0059195	0.024200113
Ccdc74a	-1.6660	-0.4082	7.5606	0.005965653	0.024365981
4930486L24Rik	-1.1790	-0.0555	7.5570	0.005977761	0.02440174

Crygn	-1.1571	0.8912	7.5443	0.006019985	0.024560325
Fcer2a	-1.0312	0.0551	7.5387	0.006038673	0.024613566
Usp44	-2.8097	-1.3396	7.5208	0.006099009	0.024799306
Dnajc12	-1.0500	1.9419	7.4818	0.006232528	0.025271606
Mafa	-1.4319	1.6672	7.4794	0.006240962	0.025296405
Slamf7	-1.1063	0.9776	7.4602	0.006307862	0.025515461
Hepacam2	-1.4716	1.3450	7.4511	0.006339789	0.025630359
Unc79	-1.2153	0.4497	7.4434	0.006366803	0.025720519
9130204L05Rik	-3.7526	0.4522	7.4241	0.006435629	0.025955333
Trim30d	-1.6825	-0.1683	7.3910	0.006554964	0.026348999
Ctnna3	-1.2849	0.9387	7.3856	0.006574961	0.026400217
Olfr816	-1.4445	0.2430	7.3593	0.006671733	0.026686765
Mup13	-4.1136	-1.9399	7.3330	0.006770077	0.027029544
Grik2	-1.2562	-0.3192	7.3215	0.006813587	0.027188344
Nacad	-1.0548	0.3778	7.2999	0.006895917	0.027476686
Sall4	-1.6164	-0.8153	7.2742	0.00699525	0.027816629
Wfdc9	-3.8791	-2.0098	7.2640	0.0070352	0.027955122
S100g	-1.7518	-0.7444	7.2580	0.007058669	0.028027973
Krt73	-3.1299	-0.4540	7.2499	0.007090464	0.028123533
Wbp2nl	-2.2971	-1.5869	7.2333	0.007156439	0.02835945
Megf10	-1.0113	0.2272	7.2326	0.007159185	0.028365184
Hspb3	-1.0196	1.4922	7.2281	0.007176917	0.02840966
Kynu	-1.0829	0.1442	7.2243	0.007192385	0.028460568
Lsmem1	-1.0765	2.7338	7.2215	0.007203713	0.028493841
Pex5l	-1.1093	0.3317	7.2213	0.007204232	0.028493841
Lin28a	-2.4410	-1.2299	7.2032	0.007277304	0.028713325
Cnksr2	-1.2606	0.5677	7.1825	0.007361716	0.028983522
Gm10036	-3.8736	0.5412	7.1492	0.007499747	0.029463197
Spta1	-2.5281	-1.4403	7.1318	0.007573003	0.029708217
Asb15	-1.1300	3.8170	7.1298	0.007581458	0.029736041
Zfp456	-1.0541	0.2203	7.0922	0.00774194	0.030278603
Stc1	-1.0494	0.6176	7.0798	0.007795756	0.030467088
Gsto2	-1.3560	-0.6389	7.0459	0.007944717	0.030966098
Foxp2	-1.0958	1.2884	7.0353	0.0079919	0.031133327
Prdm6	-1.6602	-0.5451	7.0293	0.008018458	0.031214506
Chrdl2	-1.6430	-0.2009	7.0193	0.008063551	0.031373262
Cul7	-1.0118	3.5386	6.9751	0.008265034	0.032008861
Klf14	-2.1729	-1.1098	6.9715	0.008281723	0.03206212
Pik3c2g	-1.0499	1.9113	6.9661	0.008306734	0.03214263
BC049352	-4.1512	-1.9235	6.9581	0.008343843	0.032256852
Actbl2	-4.1970	-1.8920	6.9106	0.008568714	0.032951137
Trim43a	-2.6773	-1.7981	6.8865	0.008684719	0.03333265
Gm42791	-1.1005	0.0974	6.8508	0.008860155	0.033916521
Zc2hc1c	-1.1887	0.8012	6.8396	0.008915774	0.03406375
Retnlg	-1.3546	0.3385	6.8305	0.008961431	0.034221886
Rsph10b	-1.0905	-0.0211	6.8235	0.008996572	0.034312396
Olfr118	-3.6371	-2.1083	6.8136	0.009046486	0.034466637
Olfr339	-3.7555	-2.0645	6.8124	0.009052731	0.034473136
Plac9a	-1.5704	0.6012	6.7878	0.009178421	0.0348295
C1ql3	-2.2318	-1.3421	6.7817	0.009209662	0.03491164
Col22a1	-1.0935	0.8643	6.7796	0.00922031	0.034933806
Apba2	-1.1128	0.2656	6.7552	0.00934741	0.035360128
Krt24	-1.1331	0.7349	6.7534	0.009356721	0.035377566
Siglecf	-1.6276	-0.9441	6.7510	0.009369657	0.035419737
Gm11559	-3.5714	-2.1271	6.7503	0.009373158	0.035420704
Sat2	-1.0370	0.5770	6.7456	0.0093977	0.035501156
Serpinb9b	-1.0339	0.1057	6.7452	0.009399792	0.035501915
Amph	-4.1626	-1.9550	6.7445	0.009403944	0.035501915

F2rl3	-1.7571	-0.7610	6.7389	0.009433403	0.035556045
Bend7	-1.2979	-0.2429	6.7302	0.009479188	0.035703961
Patl2	-1.9125	-1.2745	6.6990	0.009646523	0.036239989
Avp	-2.7402	-1.7770	6.6987	0.009648061	0.036239989
Mfsd4b1	-1.1400	0.5005	6.6475	0.009929562	0.037143981
D630003M21Rik	-1.0695	0.2607	6.6349	0.009999911	0.037375117
Gm2564	-2.1824	-1.0856	6.6298	0.010028512	0.037456364
Nhlh1	-2.3536	-1.5492	6.6073	0.010156168	0.037874837
Dok5	-1.3512	-0.2909	6.5841	0.010289279	0.038279696
Nog	-1.3143	0.8702	6.5673	0.010386701	0.03855804
Ugt8a	-1.1005	0.2522	6.5660	0.010394321	0.038571888
Olfr1264	-2.6802	-1.7458	6.5632	0.010411074	0.038620929
Zfp286	-1.2012	0.0329	6.5308	0.010602041	0.039189495
Gm14322	-1.8255	-1.2838	6.5041	0.010762554	0.039708859
Fgf10	-1.1040	1.7398	6.4951	0.01081704	0.039882925
Dyx1c1	-1.2400	0.0594	6.4454	0.011123694	0.040827397
Prr29	-1.1610	0.3100	6.4437	0.011134486	0.040846406
Mymx	-2.8780	-1.7226	6.4318	0.011209196	0.041079061
C530008M17Rik	-1.0273	1.0069	6.4308	0.011215927	0.041089933
Atp2b3	-1.4598	0.7740	6.4289	0.011227795	0.041119611
Gpha2	-1.5117	-0.5354	6.4062	0.011371955	0.041543032
Adra2c	-1.2794	-0.5892	6.4042	0.011384944	0.041576567
Cdhr4	-2.6263	-1.8138	6.3989	0.01141895	0.041679837
Gipc3	-1.7250	-1.0368	6.3836	0.011518124	0.041950646
Prss55	-1.9641	-1.0772	6.3714	0.011597262	0.042175549
Xlr3c	-1.5014	-0.4989	6.3697	0.011608442	0.042209176
Sag	-1.3298	-0.5368	6.3681	0.011618894	0.042233114
Zfp618	-1.1970	0.3173	6.3668	0.011627471	0.042257255
Marco	-2.3039	-1.5867	6.3643	0.011644073	0.042310448
Myh13	-3.6223	-2.1152	6.3452	0.011769694	0.042674672
Fbxo16	-2.6029	-1.8281	6.3262	0.011896731	0.04306376
Aifm3	-2.0679	-1.1700	6.3180	0.011951948	0.043213478
Ubap1l	-1.3560	-0.7358	6.3135	0.011982231	0.043308622
Gdf6	-2.5973	-1.8269	6.2950	0.012107714	0.04366818
Gm7697	-3.4838	-2.1508	6.2909	0.012135994	0.043748496
Smim10l2a	-1.5378	-0.7451	6.2860	0.012169847	0.043848566
Mstn	-1.4727	6.8991	6.2837	0.012185651	0.043876787
Pipox	-3.5534	-2.1286	6.2832	0.01218863	0.043880273
Gpr12	-3.6046	-2.1225	6.2489	0.012426792	0.044594016
Scgb1a1	-1.2733	0.6972	6.2468	0.012441835	0.04462259
Slc7a13	-1.6795	-0.7629	6.2258	0.012589862	0.045064646
Nmb	-1.1315	0.5949	6.2158	0.012661493	0.045261677
Rasgef1c	-1.1197	-0.2007	6.1720	0.012978905	0.046207189
Muc3a	-1.1541	-0.4180	6.1653	0.013027949	0.046344004
Gabra1	-3.8620	-2.0562	6.1509	0.01313424	0.04666478
Bcl2l14	-1.3323	1.7462	6.1453	0.013175922	0.04677131
Zfp493	-1.1444	0.0483	6.1441	0.013185396	0.046789723
Fndc7	-2.5669	-0.9618	6.1355	0.013249038	0.046985013
Rspo2	-2.5751	-1.4629	6.1210	0.013358479	0.047319318
Ddn	-1.3549	-0.5411	6.1151	0.013402831	0.047437938
C2cd4c	-2.1803	-1.6351	6.1104	0.013438827	0.047508445
4921507P07Rik	-1.4666	-0.3933	6.0963	0.013546477	0.047861004
Apol7c	-2.3270	-1.5838	6.0337	0.014035154	0.049332063
Olfr981	-3.5143	-2.1358	6.0322	0.01404732	0.049350987
Tnfsf13b	-1.6640	0.4197	6.0283	0.014078617	0.049437075

Supplementary Table S3. The genes upregulated in the SSS samples compared with the NT controls

Gene	logFC	logCPM	LR	PValue	FDR
Csf3	8.9393	3.6539	365.1879	2.08906E-81	4.56062E-77
Prg4	4.9614	6.8756	328.2434	2.31938E-73	2.53172E-69
Timp1	5.7229	5.9870	257.7889	5.2058E-58	3.78826E-54
Cyp7b1	5.0848	4.2819	241.1165	2.24528E-54	1.22542E-50
Cxcl1	5.9574	4.8689	236.5545	2.21817E-53	9.68497E-50
Il19	7.8325	2.5644	218.4276	1.99236E-49	7.2492E-46
Cxcl5	10.1819	6.7462	217.9264	2.56273E-49	7.99243E-46
Tnc	6.9508	9.4735	211.0002	8.31083E-48	2.26792E-44
Il6	9.1314	3.8465	204.7164	1.95285E-46	4.73695E-43
Plaur	5.2809	5.3421	203.3749	3.83154E-46	8.36463E-43
Serpina3n	4.4823	8.2924	198.5296	4.37225E-45	8.67732E-42
Sh2d5	5.7477	3.2209	197.2328	8.38917E-45	1.5262E-41
Ppbp	8.0744	4.3680	194.4818	3.34279E-44	5.61357E-41
Cxcl3	9.8112	5.5466	190.3081	2.72314E-43	4.24635E-40
Ccl7	5.1575	4.9184	186.0953	2.26301E-42	3.29359E-39
Ccl8	3.1341	6.1918	185.8107	2.61102E-42	3.56258E-39
Il4ra	3.9149	6.5873	183.2243	9.58209E-42	1.23051E-38
Lrg1	4.2632	7.8952	179.3734	6.64107E-41	8.05451E-38
Fgf23	8.6308	3.3507	178.8882	8.47576E-41	9.73865E-38
Adamts4	7.4743	4.8935	177.3713	1.81716E-40	1.98352E-37
Car4	5.1901	6.0130	172.7617	1.84507E-39	1.91808E-36
Pdpn	3.4473	6.5693	166.1765	5.06186E-38	5.02297E-35
Mcoln2	3.8214	4.2850	164.9486	9.38745E-38	8.91033E-35
Msr1	5.0082	4.8366	158.8294	2.03896E-36	1.85469E-33
Mmp3	4.1331	7.3425	157.6698	3.65411E-36	3.19092E-33
Clec4e	11.6684	4.1371	157.2186	4.58554E-36	3.85027E-33
Fpr1	8.3851	3.5935	154.8444	1.51428E-35	1.22438E-32
Slc39a14	3.8990	6.9182	152.4787	4.9796E-35	3.88249E-32
Rtn4rl2	4.8583	2.8545	152.0982	6.03052E-35	4.53974E-32
Ccl2	5.0395	4.3878	149.7387	1.97732E-34	1.4389E-31
Cemip	5.4261	2.6796	148.5294	3.63423E-34	2.55932E-31
Retnlg	7.9239	6.9566	144.8998	2.25879E-33	1.54099E-30
Fpr2	7.2522	3.4629	143.8113	3.90703E-33	2.58468E-30
Ptx3	6.6190	6.7377	138.2682	6.36707E-32	4.08822E-29
Il17ra	2.6736	4.3481	138.1694	6.69163E-32	4.17386E-29
Bcat1	4.6035	3.3344	137.3266	1.02295E-31	6.20333E-29
Cyp4f18	7.5763	4.6946	136.7899	1.34043E-31	7.9089E-29
Crabp1	4.0770	4.0822	135.0825	3.16724E-31	1.81958E-28
Stfa2l1	8.4485	3.6407	134.9103	3.45434E-31	1.93363E-28
Entpd3	3.8598	2.5382	134.6371	3.96377E-31	2.16333E-28
Upp1	5.2363	4.0562	134.4739	4.30349E-31	2.29145E-28
Bcl3	2.7455	4.9200	134.1544	5.05476E-31	2.62739E-28
Apln	4.6849	3.0063	130.8695	2.64436E-30	1.34253E-27
Ccl9	3.0756	7.1973	130.7901	2.75224E-30	1.36555E-27
Treml4	7.7566	2.4895	124.9006	5.35086E-29	2.59588E-26
Sprr2f	6.3570	3.8873	123.7100	9.74994E-29	4.6272E-26
Mmp8	9.4901	5.3160	123.0617	1.35178E-28	6.27886E-26
Chil3	10.2031	2.6882	122.4144	1.87322E-28	8.51962E-26
Trem1	9.5850	5.1198	122.2667	2.01802E-28	8.99088E-26
Lilrb4a	4.5227	4.9918	121.2637	3.34578E-28	1.46083E-25
Lcn2	6.7881	8.2708	120.8221	4.17985E-28	1.78922E-25
Gm5483	8.3409	3.8868	119.4916	8.17415E-28	3.43173E-25
S100a9	6.6346	9.5585	118.9219	1.08934E-27	4.48705E-25
Lrrc32	2.7383	5.2076	117.9204	1.80491E-27	7.29684E-25
Cd14	4.9899	7.1235	116.9326	2.96996E-27	1.17886E-24
Ms4a4c	6.1439	3.4926	115.3085	6.73603E-27	2.62597E-24

Il24	10.0630	2.5449	114.6906	9.19871E-27	3.52311E-24
Gm5416	6.9309	3.0296	113.5936	1.59951E-26	6.02049E-24
Slc7a11	3.9707	4.9735	110.2781	8.5166E-26	3.15129E-23
Mrgpra2b	10.8922	3.3681	109.6562	1.1655E-25	4.21739E-23
Pf4	3.1842	6.0790	109.6343	1.17842E-25	4.21739E-23
Wfdc17	3.7165	5.8182	108.7368	1.85331E-25	6.52574E-23
Ms4a6d	4.2024	5.8156	108.4270	2.16689E-25	7.5088E-23
Ccl12	6.3478	1.8834	108.0106	2.67341E-25	9.11926E-23
Gm10309	7.4597	2.2070	107.8619	2.8818E-25	9.67886E-23
Slfn1	5.5846	3.8574	106.8669	4.76086E-25	1.57476E-22
Ccr5	3.5164	4.3889	106.8235	4.86631E-25	1.58562E-22
Mlkl	2.9355	3.1548	105.0376	1.1984E-24	3.84738E-22
Ccl4	7.3538	4.5674	104.7828	1.36282E-24	4.31186E-22
2010005H15Rik	7.2333	1.9871	102.9674	3.40713E-24	1.06259E-21
Snai1	3.6110	3.5666	102.1224	5.21954E-24	1.6049E-21
B4galt5	2.3105	5.2621	101.2259	8.20708E-24	2.48845E-21
Lbp	2.6539	7.3908	101.1266	8.62881E-24	2.58049E-21
Olfm4	7.9441	2.6722	99.2191	2.26057E-23	6.66898E-21
Ifi204	2.2587	5.9224	97.5360	5.28864E-23	1.53942E-20
Plek	3.7485	6.0951	97.4436	5.54122E-23	1.59171E-20
Samsn1	4.5555	4.1944	95.9785	1.16136E-22	3.29267E-20
Nlrp3	5.6453	4.5718	95.3329	1.60914E-22	4.50373E-20
Cfb	2.8416	7.0718	94.5285	2.41594E-22	6.67626E-20
Bst1	3.6732	4.2379	93.9924	3.16732E-22	8.64323E-20
Cd300lf	5.7678	4.5133	93.4596	4.14567E-22	1.11733E-19
Mt2	3.2288	9.4092	92.9883	5.26052E-22	1.40052E-19
Olfr1033	3.1448	6.9539	92.8572	5.62059E-22	1.47835E-19
Slfn2	2.8874	5.4967	92.8231	5.71825E-22	1.48613E-19
Ddah1	3.1563	5.1170	92.7023	6.07843E-22	1.56116E-19
Glis3	3.6615	3.0786	92.5510	6.56106E-22	1.66552E-19
Osm	6.4153	3.8317	92.3776	7.1622E-22	1.79722E-19
Prok2	9.7633	2.2580	90.9961	1.43958E-21	3.5713E-19
S100a8	8.5868	8.9146	90.5807	1.77584E-21	4.35601E-19
Mcemp1	6.7486	3.7860	90.4800	1.86855E-21	4.53247E-19
Ifi202b	2.5649	7.1475	90.2673	2.08062E-21	4.99143E-19
Slc15a3	4.6069	4.1168	90.1900	2.16354E-21	5.13395E-19
Slfn4	6.5334	6.7781	90.1067	2.25655E-21	5.29706E-19
Ccl6	2.8394	7.5574	89.9593	2.43109E-21	5.64608E-19
Cd38	2.5991	3.8931	89.8368	2.58638E-21	5.9435E-19
Lce3b	9.7105	2.1898	88.5115	5.05415E-21	1.14934E-18
Serpinh1	2.0661	7.9139	88.3888	5.37751E-21	1.21027E-18
Adamts9	4.0065	4.8820	87.7278	7.51096E-21	1.67318E-18
Srgn	4.3564	6.9978	87.1316	1.01535E-20	2.239E-18
Hp	6.3509	10.3347	86.3203	1.53027E-20	3.34073E-18
Zfp729a	2.2104	7.7315	85.9327	1.86163E-20	4.02388E-18
Tmem8	3.5432	5.3058	85.5318	2.2801E-20	4.88009E-18
Mt1	2.8621	9.1171	84.9751	3.02145E-20	6.40401E-18
Lrrc59	1.9094	7.2334	84.8236	3.26209E-20	6.84756E-18
Adamts1	2.4389	6.7987	84.6882	3.49337E-20	7.26321E-18
Csf2rb2	2.6141	4.9306	83.7815	5.52599E-20	1.13809E-17
Mmp19	2.7378	5.6774	83.6159	6.00864E-20	1.22593E-17
Csf2rb	2.7383	6.4802	83.4440	6.55472E-20	1.32496E-17
Tlr13	3.8656	4.1361	82.8687	8.76868E-20	1.75623E-17
Gjb2	4.9344	6.8723	82.5377	1.03672E-19	2.05751E-17
Cd177	7.5330	3.8043	82.4782	1.0684E-19	2.10129E-17
Pla2g4d	6.0088	0.8355	81.9782	1.37599E-19	2.68208E-17
Tmem173	2.3331	4.6366	81.8817	1.44483E-19	2.79134E-17
Csf3r	5.7834	5.8974	81.3945	1.84872E-19	3.54031E-17

Hdc	4.9166	6.1530	81.3622	1.87917E-19	3.56731E-17
Selp	4.9539	5.8124	81.0672	2.18172E-19	4.10596E-17
Gldc	3.2053	2.7344	80.9564	2.30752E-19	4.3056E-17
Fcgr1	4.4622	3.7230	80.6258	2.72776E-19	5.04659E-17
Layn	2.5831	2.7983	80.5717	2.80346E-19	5.14305E-17
Cdhr1	5.6687	1.8128	80.4773	2.94069E-19	5.34985E-17
Uox	6.3176	1.1029	80.1649	3.44438E-19	6.2144E-17
Smim3	3.0004	3.6066	79.1775	5.67722E-19	1.0159E-16
Fcer1g	2.3896	5.8281	78.9545	6.35583E-19	1.12808E-16
Fkbp10	2.0234	5.0810	78.3794	8.50338E-19	1.4932E-16
Thbs1	3.0968	8.8115	78.3687	8.54977E-19	1.4932E-16
Inhba	4.2266	3.1562	78.0872	9.85907E-19	1.7082E-16
Ifitm1	4.5068	6.2217	77.9743	1.04391E-18	1.79445E-16
Krt6a	5.4182	10.6899	77.8679	1.1017E-18	1.879E-16
Serpina3m	4.9695	2.0464	77.5858	1.27082E-18	2.15064E-16
Isyna1	1.5634	5.2668	77.5299	1.30729E-18	2.19534E-16
Ccr1	4.5714	4.9468	77.0513	1.66572E-18	2.7759E-16
Sprr2e	5.1519	3.8396	77.0243	1.68868E-18	2.79284E-16
Klk6	4.6639	7.6543	76.9529	1.75083E-18	2.85588E-16
Oasl1	2.5391	2.7951	76.9505	1.75295E-18	2.85588E-16
Phlda1	1.9176	4.6164	76.9131	1.7865E-18	2.88897E-16
Adgra2	2.4801	5.6822	76.8120	1.88031E-18	3.01831E-16
Ripk3	2.1941	4.4247	76.7611	1.92936E-18	3.07444E-16
BC117090	6.3763	1.1640	76.6247	2.06738E-18	3.27051E-16
Prl2c3	9.0988	1.6141	76.5580	2.13838E-18	3.35849E-16
Fcgr4	4.2007	2.4080	76.2305	2.52411E-18	3.93599E-16
Padi4	3.4241	2.9016	76.1139	2.67762E-18	4.14575E-16
Ifi205	1.9491	5.5981	75.8411	3.07432E-18	4.72644E-16
Mmp10	5.8635	1.7574	75.6610	3.36791E-18	5.1416E-16
Clec4n	3.9541	3.9237	75.5551	3.55359E-18	5.38739E-16
Gprc5a	3.3838	2.3165	74.8466	5.08743E-18	7.65956E-16
Tinagl1	2.1585	5.7809	74.6978	5.48568E-18	8.2026E-16
Helz2	1.9752	4.8685	74.5591	5.88488E-18	8.73964E-16
Stx11	3.0926	4.5042	74.3655	6.49121E-18	9.57497E-16
Trem3	6.3672	1.9646	74.1493	7.24254E-18	1.06115E-15
Hmga2	4.5354	2.0573	73.9528	8.00077E-18	1.16443E-15
Sec24d	1.6811	5.7340	73.9370	8.06514E-18	1.16603E-15
Msn	1.9537	8.0215	73.6735	9.21671E-18	1.32375E-15
Sprr2i	5.7087	3.1410	73.5392	9.86586E-18	1.40772E-15
Klk14	5.3343	1.2874	73.3287	1.09761E-17	1.55597E-15
A4galt	1.5631	4.2758	72.8952	1.36722E-17	1.92566E-15
Steap4	3.4132	7.5027	72.3154	1.83411E-17	2.56669E-15
Glipr2	2.5973	4.9238	72.2818	1.86563E-17	2.59418E-15
Col8a1	3.3860	4.8974	72.1005	2.04512E-17	2.82576E-15
Hck	3.2535	3.8397	71.8501	2.32181E-17	3.1879E-15
Trim30b	6.3443	3.4869	71.2383	3.16591E-17	4.31968E-15
Sprr1b	5.0020	7.7963	71.1900	3.24427E-17	4.39911E-15
Fcgr2b	2.5224	6.6984	70.9574	3.65016E-17	4.91894E-15
Sprr2j-ps	5.2666	1.4177	70.9274	3.70608E-17	4.96365E-15
Lgmn	2.0663	8.0510	70.6452	4.27611E-17	5.69218E-15
Efemp2	2.0229	5.3030	70.6149	4.34228E-17	5.74523E-15
Emilin1	2.4568	4.6974	70.1272	5.56009E-17	7.31219E-15
Gch1	2.4863	3.2762	69.9263	6.15623E-17	8.0477E-15
Col18a1	1.5533	6.8407	69.9139	6.19518E-17	8.05041E-15
Rarres2	1.9433	7.8563	69.1780	8.99677E-17	1.15683E-14
Steap1	4.8226	1.5389	69.1754	9.00831E-17	1.15683E-14
Clec4d	6.8569	5.2435	68.9153	1.02783E-16	1.3122E-14
Ms4a4a	3.1686	5.1208	68.7396	1.1236E-16	1.42612E-14

Sprr2h	4.0832	6.0581	68.6431	1.17997E-16	1.48864E-14
Sell	5.7881	4.8499	68.6322	1.18649E-16	1.48864E-14
Galnt6	2.0572	4.6204	68.5471	1.23881E-16	1.5454E-14
Fscn1	2.4042	5.9952	68.4933	1.27307E-16	1.57912E-14
Tmem252	2.2766	3.6274	68.3816	1.34731E-16	1.66176E-14
Tm4sf1	2.4636	4.6099	68.2082	1.47112E-16	1.80427E-14
Stc1	2.6471	2.5724	68.0576	1.5879E-16	1.93662E-14
Plvap	1.8357	6.6518	67.7858	1.82255E-16	2.21044E-14
Ecscr	1.9890	4.2184	67.6570	1.94565E-16	2.34672E-14
Ifitm3	1.6564	8.1639	67.4628	2.14697E-16	2.5753E-14
Eif1a	1.5483	6.0553	66.9618	2.76813E-16	3.30225E-14
F10	4.0433	2.5717	66.9507	2.78376E-16	3.30284E-14
Sele	3.7824	3.4935	66.6665	3.21546E-16	3.79441E-14
Nek6	1.6506	5.6721	66.4899	3.51691E-16	4.12783E-14
Gk	2.6084	4.4275	66.4283	3.62846E-16	4.23599E-14
Ngp	8.6620	1.1962	66.1908	4.09309E-16	4.75299E-14
Stfa1	5.6964	6.1345	66.0653	4.36221E-16	5.03869E-14
Ccl21a	1.5219	5.8351	65.8927	4.76157E-16	5.47105E-14
Chil1	2.8565	6.8315	65.8115	4.96191E-16	5.67138E-14
Rnd1	3.2347	2.8329	65.7940	5.0061E-16	5.69209E-14
Alpl	3.6568	4.1407	65.7550	5.10608E-16	5.77569E-14
Aldh18a1	1.5244	4.7004	65.7443	5.13376E-16	5.77707E-14
Arpc1b	1.6510	7.3761	65.0970	7.13017E-16	7.9825E-14
Igfbp7	1.6731	7.4716	64.4789	9.75706E-16	1.08677E-13
Slpi	4.7825	5.2976	64.3206	1.05736E-15	1.17174E-13
Csgalnact1	1.8658	3.6463	63.6325	1.49931E-15	1.65311E-13
Lce3d	5.7215	3.7847	63.3259	1.75186E-15	1.92185E-13
Tnfaip6	4.5706	4.4078	63.0601	2.00499E-15	2.18855E-13
Ctps	1.6382	5.4857	62.8979	2.17709E-15	2.35528E-13
Lyn	2.5893	5.2219	62.8958	2.17932E-15	2.35528E-13
Adam12	3.7228	3.7246	62.7672	2.32636E-15	2.50181E-13
Itgam	3.0095	6.5554	62.2673	2.99862E-15	3.20896E-13
Saa1	5.8669	3.1202	62.1545	3.17539E-15	3.38156E-13
Rgs16	2.7921	2.3691	62.0902	3.28087E-15	3.47693E-13
Tubb3	4.8471	2.7788	61.8322	3.74001E-15	3.94435E-13
Il1b	6.9374	7.1172	61.8076	3.78707E-15	3.97479E-13
Aif1	2.6361	2.6868	61.1157	5.38162E-15	5.62135E-13
Nus1	1.4410	6.1180	61.0608	5.53384E-15	5.75282E-13
Pi15	2.2474	4.3070	60.7034	6.63556E-15	6.86544E-13
Lox	2.4957	6.4855	60.3500	7.94043E-15	8.17677E-13
Zbp1	1.8837	3.7844	59.6181	1.15172E-14	1.18043E-12
Sod3	1.4257	7.4464	59.4514	1.25349E-14	1.27874E-12
Mark1	1.7347	3.5107	59.2375	1.39743E-14	1.4166E-12
Cd300ld	4.8220	2.3632	59.2317	1.40161E-14	1.4166E-12
Pglyrp1	4.2737	3.3971	59.2032	1.42199E-14	1.43057E-12
Osmr	1.6557	6.5990	58.8770	1.67839E-14	1.68077E-12
Cd53	3.0246	5.9893	58.4506	2.08455E-14	2.07798E-12
Slc41a2	2.0756	3.3592	58.4100	2.12806E-14	2.11171E-12
Klra17	7.6604	1.7010	58.2611	2.2954E-14	2.26746E-12
Tlr2	2.6848	4.1642	58.1000	2.49127E-14	2.44986E-12
Kdelr3	2.1208	3.6452	57.6104	3.19538E-14	3.12818E-12
Aplnr	2.6573	3.5248	57.3320	3.68119E-14	3.58768E-12
Col4a2	2.5624	8.3764	57.1521	4.0338E-14	3.9089E-12
Rab31	1.8079	6.0214	57.1459	4.0466E-14	3.9089E-12
Runx1	1.6105	4.6042	57.1309	4.07741E-14	3.92132E-12
Dhx58	2.1252	2.9098	56.4641	5.72346E-14	5.48021E-12
Il18rap	4.8055	3.8616	56.4296	5.82474E-14	5.55283E-12
Sox18	1.7250	4.0287	56.1319	6.77685E-14	6.4324E-12
Gda	2.3097	7.1354	56.0431	7.08999E-14	6.7005E-12

Hcls1	2.3954	5.2044	56.0222	7.1656E-14	6.74277E-12
Nxpe5	1.4307	4.6325	55.9853	7.3014E-14	6.84107E-12
C1qtnf6	1.7708	3.7254	55.9309	7.50624E-14	7.00293E-12
Sema6b	2.2500	4.1459	55.6731	8.55823E-14	7.95042E-12
Fgr	3.4493	4.1300	55.5988	8.888E-14	8.22177E-12
Hhipl1	3.4361	2.1528	55.3445	1.0115E-13	9.31735E-12
Krt16	4.6921	9.6942	55.2878	1.04113E-13	9.54999E-12
Tmem198b	2.1618	4.2649	54.9818	1.21648E-13	1.11117E-11
A730049H05Rik	7.1709	-0.1390	54.8551	1.29755E-13	1.18028E-11
Olr1	3.4487	0.3466	54.8216	1.31985E-13	1.19065E-11
Cxcl12	2.4128	7.9110	54.4889	1.5633E-13	1.40446E-11
Efhd2	1.6061	6.5405	54.3678	1.66267E-13	1.48761E-11
Fblim1	1.7870	4.0134	54.3044	1.71713E-13	1.53007E-11
Entpd1	2.4741	6.1009	54.2704	1.74715E-13	1.55049E-11
Cd80	3.1057	1.9681	54.1271	1.87929E-13	1.661E-11
Gm5150	6.3870	1.4304	54.0364	1.96813E-13	1.73251E-11
Mterf4	1.4113	5.6670	53.8815	2.12949E-13	1.86702E-11
Cotl1	1.6802	6.2874	53.8206	2.19661E-13	1.91816E-11
Gm5478	8.0554	2.7859	53.6534	2.39171E-13	2.08022E-11
Cd300c2	3.2105	1.4195	53.6209	2.43162E-13	2.10654E-11
Defb3	8.3625	0.9045	53.5482	2.5232E-13	2.17723E-11
Praf2	1.4897	3.8665	53.4832	2.60815E-13	2.24168E-11
Olfr1217	3.2261	2.2104	53.3992	2.72205E-13	2.32776E-11
Galk1	1.7903	3.6454	53.3938	2.72963E-13	2.32776E-11
Tarm1	4.1744	2.3825	53.3536	2.78605E-13	2.36662E-11
Saa3	7.8403	8.6721	53.3371	2.8095E-13	2.37729E-11
Lgals9	1.2719	5.8186	53.0604	3.23441E-13	2.72627E-11
Tspan11	1.7747	3.7566	52.6959	3.8941E-13	3.25717E-11
Clec5a	3.3807	1.3007	52.6235	4.0403E-13	3.36655E-11
Il13ra2	4.1167	1.9532	52.6074	4.07343E-13	3.38126E-11
Tlr1	5.7012	1.8930	52.4570	4.39779E-13	3.63667E-11
Tnfrsf1b	2.0033	5.0073	52.3141	4.72964E-13	3.89633E-11
Tspan4	1.9228	5.7843	52.2557	4.87241E-13	3.99885E-11
Cdr2l	1.4488	4.0312	52.1363	5.17777E-13	4.23356E-11
Ms4a6b	2.8338	4.6174	52.1275	5.20107E-13	4.23674E-11
Rpl39l	2.5319	3.0558	52.0959	5.28541E-13	4.28943E-11
Pgm1	1.6688	3.9311	51.9959	5.56175E-13	4.48039E-11
Rtp4	1.6922	3.5765	51.9654	5.64881E-13	4.53379E-11
Sirpb1b	7.3978	0.0595	51.9423	5.71558E-13	4.57058E-11
Il33	2.0927	7.0119	51.8341	6.03933E-13	4.81185E-11
Cyp20a1	1.8563	3.5921	51.6667	6.57686E-13	5.22107E-11
Lcp2	2.5512	4.1917	51.5870	6.84926E-13	5.41761E-11
B430306N03Rik	4.3214	1.9715	51.4513	7.3395E-13	5.76845E-11
Sprr2k	6.0347	0.2044	51.4497	7.34565E-13	5.76845E-11
C5ar1	2.8912	4.8014	51.3773	7.62158E-13	5.96369E-11
Sdf2l1	1.9643	4.9726	51.3645	7.67116E-13	5.98104E-11
Serpine1	2.2772	6.5170	51.1208	8.6854E-13	6.74772E-11
Tcf23	4.4485	2.5573	50.9608	9.42281E-13	7.29466E-11
Svep1	2.1354	6.4309	50.9323	9.56063E-13	7.3752E-11
Il1r1	2.0329	6.9006	50.9158	9.6415E-13	7.41139E-11
Lpgat1	2.2973	6.8106	50.5379	1.16884E-12	8.95333E-11
Hif1a	1.4087	7.0090	50.4820	1.20261E-12	9.17978E-11
Lce3f	6.3031	1.9244	50.3707	1.2728E-12	9.68174E-11
Vmp1	1.3833	5.9045	50.3206	1.30569E-12	9.89743E-11
Sdcbp	1.5323	7.1237	50.1389	1.43239E-12	1.08202E-10
Ccr7	2.3826	1.5926	50.0203	1.52162E-12	1.14547E-10
Stom	1.7063	6.7789	49.8375	1.67021E-12	1.253E-10
Hgf	3.4578	1.3754	49.7197	1.77357E-12	1.32599E-10
Mmp9	2.0356	5.7052	49.4224	2.06376E-12	1.53767E-10

Sirpb1c	5.4681	1.7326	49.1769	2.33895E-12	1.73679E-10
Nos3	2.0547	4.6466	49.1206	2.40701E-12	1.78127E-10
F7	4.4936	0.9316	49.0092	2.54764E-12	1.87897E-10
Pdia5	1.8417	3.5370	48.9691	2.6003E-12	1.91135E-10
Spp1	5.3390	6.9976	48.7257	2.94384E-12	2.15661E-10
Serpinb1a	2.1724	6.1465	48.5898	3.15512E-12	2.30366E-10
Cxcr2	3.4392	5.8188	48.5364	3.24213E-12	2.3593E-10
Akr1b8	2.0191	5.2226	48.1467	3.95484E-12	2.85888E-10
Vcan	2.9615	6.3063	48.0484	4.15828E-12	2.99602E-10
Cthrc1	1.9590	2.3794	47.9820	4.30147E-12	3.08899E-10
Nipsnap3b	1.8392	3.5338	47.9614	4.3469E-12	3.11138E-10
Gja5	2.1100	2.5654	47.8636	4.56926E-12	3.25986E-10
Socs3	1.7734	7.3960	47.6685	5.04748E-12	3.58239E-10
Bcl2a1b	2.7453	1.7694	47.6604	5.06817E-12	3.58239E-10
Cd63	1.5041	7.8349	47.6595	5.07058E-12	3.58239E-10
A530032D15Rik	3.7042	1.5641	47.6095	5.20155E-12	3.66306E-10
Ptgir	2.5010	3.6905	47.5883	5.25801E-12	3.69092E-10
Itgb2l	7.9619	0.5604	47.5201	5.44431E-12	3.79952E-10
Krt6b	8.1589	9.8227	47.5189	5.44753E-12	3.79952E-10
Cyyr1	1.8329	4.4939	47.3717	5.87234E-12	4.08278E-10
Aldh3b1	1.8514	3.6498	47.3099	6.06051E-12	4.20022E-10
Rasip1	1.4604	5.0189	47.2089	6.3811E-12	4.40841E-10
Hilpda	1.7676	4.0196	47.1867	6.45377E-12	4.44455E-10
Cp	2.6792	6.5290	47.0872	6.7899E-12	4.66133E-10
Lgi2	2.1629	5.4149	46.8661	7.6008E-12	5.18541E-10
Lce3a	4.7721	1.4739	46.5012	9.15614E-12	6.18847E-10
Pgf	2.6108	1.7249	46.4767	9.27136E-12	6.24701E-10
Cntn2	4.5781	0.5655	46.3721	9.77993E-12	6.5694E-10
B3gnt5	4.1693	1.2053	46.3343	9.9702E-12	6.67667E-10
Ets1	1.4218	5.2341	46.2922	1.01868E-11	6.80086E-10
Dab2	2.5745	7.2218	46.2746	1.02785E-11	6.84118E-10
Icam1	1.6715	5.2921	46.1546	1.0928E-11	7.24178E-10
Itgb2	2.3745	5.7792	46.1512	1.09468E-11	7.24178E-10
Cyba	1.7736	5.1284	46.0166	1.17255E-11	7.73355E-10
Esam	1.5651	4.9801	46.0079	1.17775E-11	7.7444E-10
Ptgs2	2.2637	4.9362	45.9277	1.22699E-11	8.04396E-10
Chsy1	1.7406	3.8309	45.8034	1.30736E-11	8.5452E-10
Nfkbid	2.4182	3.7310	45.7432	1.34817E-11	8.78564E-10
Tmem176a	2.2657	4.2107	45.6699	1.39959E-11	9.09356E-10
Cd68	1.6879	6.5442	45.5748	1.46916E-11	9.51318E-10
Tnfrsf26	2.6694	2.8970	45.5699	1.47288E-11	9.51318E-10
Clec4a2	2.6187	3.6944	45.5568	1.4828E-11	9.54895E-10
Coro1a	2.0106	6.1092	45.4360	1.57705E-11	1.01261E-09
Kcnab2	1.5275	3.8052	45.2545	1.7302E-11	1.10768E-09
Spatc1	5.3260	1.4890	45.2190	1.76185E-11	1.12465E-09
Steap2	1.9062	3.2539	45.0760	1.8953E-11	1.2063E-09
Col4a1	2.5050	8.9923	45.0339	1.93649E-11	1.22894E-09
Nppb	5.2346	0.1580	45.0073	1.96303E-11	1.24217E-09
Ifi211	2.4982	4.5748	44.9521	2.01911E-11	1.27396E-09
A530064D06Rik	3.8646	2.2314	44.9161	2.0566E-11	1.29388E-09
Plscr1	3.2996	2.6959	44.7940	2.1889E-11	1.37316E-09
Krt42	6.3336	-0.7904	44.5288	2.50647E-11	1.56339E-09
Asns	1.4342	5.1883	44.4347	2.62983E-11	1.63567E-09
AB124611	2.4902	3.3991	44.3207	2.78746E-11	1.72878E-09
Ms4a6c	3.0530	4.8132	44.2187	2.93666E-11	1.81615E-09
Alox5ap	2.2322	5.8014	44.1760	3.00144E-11	1.85097E-09
Tgm2	1.7941	8.4114	44.1544	3.03463E-11	1.86617E-09
Fkbp14	1.6550	3.4701	44.0893	3.13724E-11	1.92385E-09
Saa2	6.6756	1.4692	43.9992	3.28518E-11	2.00893E-09

Ms4a8a	2.0974	2.7190	43.9024	3.45174E-11	2.10489E-09
Bend4	3.2197	1.9973	43.7437	3.74321E-11	2.27627E-09
Mrgpra2a	6.8024	0.9016	43.4866	4.26871E-11	2.58164E-09
Tubb6	2.5673	6.8957	43.4865	4.26903E-11	2.58164E-09
Fam83a	2.8529	2.8746	43.4780	4.28762E-11	2.58572E-09
Acta2	1.3771	6.8880	43.0203	5.41757E-11	3.2492E-09
Sgms2	1.8514	4.5978	42.9740	5.54726E-11	3.31787E-09
Lrp8	2.2259	2.1009	42.8332	5.96131E-11	3.54608E-09
Ncf2	2.1986	5.3966	42.7223	6.30876E-11	3.74257E-09
Gsap	2.2907	2.3416	42.2480	8.04038E-11	4.74404E-09
Fgf7	1.6037	3.6821	42.1443	8.4782E-11	4.97547E-09
S100a7a	3.8675	1.9523	42.1351	8.51797E-11	4.98541E-09
Twist2	1.9819	3.8810	42.0653	8.8276E-11	5.15282E-09
Ctsz	1.3946	6.6684	41.9453	9.38614E-11	5.46423E-09
Ifi207	1.6249	6.0814	41.9140	9.53796E-11	5.52691E-09
Acod1	7.8855	4.9658	41.9126	9.54444E-11	5.52691E-09
Fkbp11	2.4023	2.8327	41.7878	1.01738E-10	5.8758E-09
Rhoj	1.1789	5.6623	41.7743	1.02443E-10	5.90087E-09
Ggt1	7.9795	0.5758	41.7029	1.06249E-10	6.10402E-09
1810055G02Rik	1.1282	4.4842	41.6146	1.11162E-10	6.36949E-09
Klk13	3.6239	4.5909	41.5457	1.15148E-10	6.58063E-09
Slc7a7	2.0970	3.7575	41.4773	1.1925E-10	6.79726E-09
Bdkrb1	3.2478	1.3214	41.3607	1.2658E-10	7.19625E-09
Sprr2d	3.8310	5.1980	41.3315	1.28484E-10	7.28554E-09
Crispld2	1.5756	6.4908	41.3004	1.30541E-10	7.38299E-09
Nedd9	1.7164	4.3665	41.2392	1.34691E-10	7.59806E-09
Gpm6b	1.9657	4.9834	41.1747	1.39215E-10	7.83302E-09
Ppp1r3b	2.6710	3.1401	41.1128	1.43691E-10	8.06407E-09
Pecam1	1.5279	6.6267	41.0984	1.44758E-10	8.10312E-09
Pdia4	1.3383	6.7485	41.0612	1.47538E-10	8.21656E-09
Far1	1.2980	5.7396	40.9494	1.56225E-10	8.67823E-09
Tubb5	1.2830	8.1892	40.8345	1.65683E-10	9.18026E-09
Jdp2	1.4968	3.8596	40.7902	1.6948E-10	9.36446E-09
St3gal4	1.3832	4.8367	40.7858	1.69865E-10	9.36446E-09
Gm13124	3.5299	1.2925	40.7631	1.71849E-10	9.44999E-09
Angptl4	2.1779	5.2842	40.5502	1.91633E-10	1.05114E-08
Nos2	4.0427	2.4560	40.5258	1.94034E-10	1.06164E-08
Gpr35	2.6676	4.1186	40.1535	2.34765E-10	1.27809E-08
Degs2	2.9494	2.8663	40.1401	2.36389E-10	1.28373E-08
Gpr84	4.4222	0.2084	40.0614	2.46107E-10	1.33319E-08
Adamts15	1.6102	6.8840	39.9577	2.59525E-10	1.4024E-08
Rab20	1.8649	3.6612	39.9342	2.62667E-10	1.41587E-08
Tmem2	1.3812	5.2798	39.9120	2.65667E-10	1.42851E-08
Ehd1	1.2045	6.8994	39.7725	2.85336E-10	1.53051E-08
Chst11	2.7083	3.8727	39.6496	3.03871E-10	1.62593E-08
Tmem176b	1.4952	5.8138	39.6337	3.06357E-10	1.63523E-08
Lrrc25	2.6399	3.5109	39.4806	3.31341E-10	1.76427E-08
Col3a1	2.1128	10.7610	39.4631	3.34324E-10	1.77582E-08
Vcam1	1.4439	3.3097	39.3739	3.49941E-10	1.84977E-08
Gfra1	1.9895	4.8884	39.3453	3.55114E-10	1.87258E-08
Ngf	2.2564	2.7719	39.3033	3.62833E-10	1.90868E-08
Akap2	2.0284	6.1987	39.2033	3.81897E-10	2.00413E-08
Me2	1.1946	3.9654	39.1816	3.86164E-10	2.02167E-08
Pdia6	1.5537	7.5983	39.1542	3.9162E-10	2.04532E-08
Lmnb1	1.6404	5.1993	39.0510	4.12886E-10	2.15125E-08
Itga5	1.9876	6.1314	38.9467	4.35529E-10	2.26382E-08
Vav1	1.7798	3.9815	38.9412	4.36762E-10	2.26484E-08
Cers2	1.4039	6.0386	38.9152	4.42618E-10	2.28976E-08
Ly6g	8.3260	0.8873	38.8283	4.62774E-10	2.38837E-08

Oas3	2.4233	3.3140	38.8210	4.64502E-10	2.39164E-08
Treml2	4.7259	1.5979	38.7947	4.70807E-10	2.4184E-08
Medag	1.8224	6.1980	38.7454	4.82838E-10	2.46281E-08
Fosl1	1.4398	3.8984	38.7366	4.8503E-10	2.46822E-08
Slc23a4	2.7340	0.4106	38.6921	4.96224E-10	2.51932E-08
Rrbp1	1.3364	7.3216	38.6585	5.04823E-10	2.55703E-08
Cpne2	1.2700	5.0722	38.6500	5.07031E-10	2.56227E-08
Pdgfra	2.0069	7.2031	38.6412	5.09315E-10	2.56787E-08
Bgn	1.2739	7.7812	38.5889	5.23161E-10	2.63159E-08
C130026I21Rik	2.5881	2.1380	38.5710	5.27978E-10	2.64972E-08
Lst1	2.8436	2.5054	38.5660	5.29331E-10	2.65042E-08
Slc7a8	2.1930	4.8313	38.1024	6.71269E-10	3.33815E-08
Itga9	1.7549	4.3849	38.0908	6.75287E-10	3.34572E-08
Artn	3.2196	1.1068	38.0891	6.75857E-10	3.34572E-08
Calu	1.2135	8.2101	37.9777	7.1558E-10	3.53435E-08
Casp4	1.4925	4.1071	37.8310	7.7147E-10	3.79323E-08
Gjb6	3.6131	3.2722	37.7499	8.04206E-10	3.94531E-08
Kcne4	2.4478	2.3955	37.6270	8.56491E-10	4.19239E-08
Dgat2l6	2.7430	2.3936	37.6193	8.59893E-10	4.19724E-08
Spi1	1.6787	4.9641	37.6161	8.61326E-10	4.19724E-08
Plac8	1.3780	5.8812	37.5124	9.08351E-10	4.41653E-08
Tnfsf14	4.4763	2.0789	37.4560	9.34971E-10	4.5258E-08
Limk1	1.4770	3.9395	37.4248	9.50049E-10	4.58861E-08
Lgals1	1.4579	8.2547	37.3744	9.74939E-10	4.69843E-08
Il1rl1	2.6697	4.2480	37.2611	1.03327E-09	4.96856E-08
Cldn15	1.5655	2.7822	37.2287	1.05057E-09	5.04068E-08
Maged1	1.4931	6.4258	37.2087	1.06139E-09	5.08139E-08
Spred3	1.5863	2.6708	37.1937	1.06957E-09	5.10934E-08
Fam167b	2.9291	0.5879	37.1841	1.07486E-09	5.12343E-08
Dynap	6.7333	-0.4799	37.0910	1.12744E-09	5.35068E-08
Lilra6	3.3481	2.8632	37.0497	1.15156E-09	5.45328E-08
Kcnn4	2.2752	2.0368	37.0391	1.15782E-09	5.47107E-08
Stat3	1.1278	8.0217	37.0150	1.17226E-09	5.50008E-08
Sprr1a	2.4247	8.3439	37.0139	1.17291E-09	5.50008E-08
Rbpms	1.1219	4.4567	37.0121	1.17399E-09	5.50008E-08
Klra2	5.0613	1.8936	37.0120	1.17404E-09	5.50008E-08
Adm	2.2610	4.5135	36.9617	1.20475E-09	5.61449E-08
Sntb2	1.6244	5.9318	36.9602	1.20567E-09	5.61449E-08
Rac2	2.2729	5.1813	36.9096	1.23734E-09	5.74731E-08
Hk3	2.7916	3.5974	36.8689	1.26342E-09	5.85601E-08
Cd163	2.3103	7.5749	36.7997	1.30909E-09	6.05481E-08
Fxyd5	1.6197	6.2786	36.7017	1.37663E-09	6.35373E-08
Sparc	1.5045	9.5587	36.6609	1.40574E-09	6.4744E-08
Cela1	2.0949	1.5644	36.5251	1.50711E-09	6.92668E-08
Jak3	1.4606	4.3851	36.3461	1.65211E-09	7.56128E-08
Cd93	2.0794	5.1556	36.3128	1.68056E-09	7.6754E-08
Adamts7	2.6459	2.4743	36.2233	1.75958E-09	8.01951E-08
Serpinb6c	2.3760	3.4312	36.2191	1.76335E-09	8.01995E-08
Tyrobp	1.9225	6.1840	36.1554	1.82197E-09	8.26931E-08
Fndc3b	1.7721	6.0212	36.1020	1.87257E-09	8.48132E-08
Gpr132	2.7172	1.7821	36.0830	1.8909E-09	8.54664E-08
Parva	1.2638	6.1192	36.0111	1.96193E-09	8.84936E-08
Ces4a	1.4963	4.8854	35.9564	2.01778E-09	9.08252E-08
Gpr171	2.1765	1.9227	35.9342	2.04093E-09	9.16782E-08
Bin2	2.0589	3.0496	35.8942	2.08331E-09	9.31984E-08
Eng	1.7059	6.4532	35.8634	2.11642E-09	9.4486E-08
Mmp12	3.5756	1.2009	35.7683	2.22235E-09	9.90123E-08
Adam8	1.8797	6.7376	35.7628	2.22863E-09	9.90526E-08
Pcsk9	5.8799	-1.1004	35.7596	2.23232E-09	9.90526E-08

Hrh2	2.2742	1.2198	35.6489	2.36283E-09	1.04066E-07
Fcgr3	2.3229	6.2922	35.6398	2.3739E-09	1.04275E-07
Cpxm1	2.0731	5.9960	35.6140	2.40548E-09	1.0545E-07
Pdgfrb	1.5735	5.9672	35.5377	2.50168E-09	1.09447E-07
Lce3e	5.8051	1.4615	35.5123	2.53445E-09	1.10659E-07
Trim30d	2.6187	1.5311	35.5062	2.54245E-09	1.10787E-07
Abcc3	1.3890	3.4239	35.4976	2.55366E-09	1.11054E-07
Fabp5	2.4497	7.9195	35.4700	2.59006E-09	1.12413E-07
Camkk2	1.4017	4.9743	35.4622	2.60053E-09	1.12643E-07
Sh2d3c	1.9472	4.1215	35.4438	2.62519E-09	1.13486E-07
Slc10a6	1.4462	4.6877	35.3659	2.7323E-09	1.17883E-07
Cyp1b1	1.8412	5.7290	35.2115	2.95777E-09	1.27109E-07
Mafb	1.0360	6.7093	35.1993	2.97623E-09	1.27651E-07
Ccl3	6.2314	4.2600	35.1558	3.04353E-09	1.30026E-07
Cald1	1.5369	6.2978	35.1175	3.10394E-09	1.32348E-07
Sbno2	1.2390	7.4787	35.0850	3.15617E-09	1.34313E-07
Nlrp12	7.5116	1.7530	35.0716	3.17801E-09	1.34979E-07
Angpt2	2.2692	3.2706	35.0576	3.20095E-09	1.35689E-07
Siglece	3.1677	2.9259	35.0508	3.21213E-09	1.35899E-07
Creb3l1	1.1816	5.0307	34.9995	3.29795E-09	1.38993E-07
Cpne8	1.2856	4.9111	34.9994	3.298E-09	1.38993E-07
Tlr4	1.9883	4.3173	34.9680	3.35175E-09	1.40987E-07
Snx20	2.0838	2.5674	34.9306	3.41669E-09	1.43442E-07
Abi3bp	2.2459	6.4271	34.9078	3.45689E-09	1.44851E-07
Gla	1.8368	3.6212	34.8868	3.4944E-09	1.45863E-07
Inhbb	2.2202	4.8824	34.8392	3.58093E-09	1.4919E-07
Apobec1	1.5424	4.3517	34.7791	3.69312E-09	1.5357E-07
Adam15	1.0323	6.6440	34.7353	3.77725E-09	1.5677E-07
Nr4a3	2.6918	4.2297	34.7215	3.80401E-09	1.57581E-07
Egfl7	1.3776	4.7922	34.6733	3.89945E-09	1.61229E-07
Creb3l3	3.3298	0.6723	34.6614	3.92333E-09	1.61909E-07
Elk3	1.2455	4.5676	34.6453	3.95587E-09	1.62945E-07
Mndal	1.9835	1.3569	34.6283	3.99065E-09	1.64068E-07
Gusb	1.3003	5.3536	34.6017	4.04549E-09	1.65698E-07
Plin2	1.3885	7.3682	34.3503	4.60326E-09	1.88191E-07
Ppa1	1.2834	6.0384	34.3131	4.69219E-09	1.91468E-07
Prdx4	1.2389	4.2367	34.2980	4.72871E-09	1.92598E-07
Dram1	1.9303	5.7925	34.2351	4.88398E-09	1.98551E-07
Cd209d	1.6564	5.4178	34.2027	4.96609E-09	2.01514E-07
Scarb1	1.3337	5.9722	34.1089	5.21112E-09	2.10674E-07
Cyth4	1.9706	5.0365	34.0453	5.38435E-09	2.16874E-07
Crem	1.5812	3.6653	34.0404	5.39785E-09	2.17017E-07
Atp1a1	1.1970	8.2450	34.0124	5.47606E-09	2.19621E-07
Snx10	1.6141	3.6693	34.0101	5.48274E-09	2.19621E-07
Slc2a3	2.5156	3.6489	33.9950	5.52537E-09	2.20924E-07
Ctss	1.6326	6.7601	33.9797	5.56904E-09	2.22263E-07
S100a4	1.1235	6.8647	33.9169	5.75168E-09	2.28716E-07
Chid1	1.0968	4.7106	33.8494	5.95483E-09	2.36363E-07
Prl2c2	6.3274	-0.7983	33.8188	6.04921E-09	2.39674E-07
Adcy4	1.5419	4.0587	33.7888	6.1433E-09	2.42961E-07
Selenon	1.6069	5.5713	33.7208	6.36161E-09	2.50686E-07
Batf	1.9064	1.6774	33.6858	6.47704E-09	2.54775E-07
Arf2	1.0253	5.1464	33.6707	6.5278E-09	2.5631E-07
BC100530	4.1131	7.6639	33.6309	6.66242E-09	2.61126E-07
Flt4	1.3114	3.4316	33.6016	6.7637E-09	2.64147E-07
Pilra	2.2506	2.2625	33.5871	6.81444E-09	2.65654E-07
4930578G10Rik	5.8291	-1.1338	33.5350	6.99925E-09	2.72372E-07
Odc1	1.8873	7.2536	33.4921	7.15528E-09	2.77948E-07
Hspa5	1.7141	9.3143	33.4825	7.19073E-09	2.78829E-07

Klk10	2.4929	6.6408	33.4133	7.45124E-09	2.88419E-07
Gm6377	2.2774	0.4185	33.4028	7.49149E-09	2.89463E-07
Arg2	2.4822	3.5364	33.3790	7.58374E-09	2.91994E-07
Slc28a2	1.9784	2.9774	33.3196	7.81905E-09	3.00524E-07
Fam43a	1.0468	5.5077	33.3094	7.86034E-09	3.0158E-07
Qsox1	1.1404	6.8891	33.3026	7.88763E-09	3.02096E-07
Kdelr2	1.0455	5.8563	33.2984	7.90468E-09	3.02219E-07
Nrg1	3.6654	2.4138	33.2837	7.96484E-09	3.03987E-07
Impdh1	1.3880	5.0781	33.1772	8.41322E-09	3.20539E-07
Ccne1	2.0792	2.1403	33.1633	8.47347E-09	3.22272E-07
Mthfd1l	1.4547	3.3450	33.1386	8.58198E-09	3.25832E-07
Gpr39	3.5473	-0.3426	33.1083	8.71674E-09	3.30373E-07
Gm9733	6.6839	-0.5073	33.0801	8.84412E-09	3.34621E-07
Pik3r5	1.6315	4.2016	33.0161	9.13982E-09	3.4521E-07
Cd44	1.1314	7.9566	32.9875	9.27513E-09	3.49716E-07
Fam71f2	3.5490	0.8140	32.9762	9.32956E-09	3.50557E-07
Alg8	1.2619	2.9036	32.9533	9.44002E-09	3.54098E-07
Ncf1	1.2108	5.2702	32.9156	9.62494E-09	3.59798E-07
Ifnar2	1.1271	5.4710	32.8250	1.00839E-08	3.76311E-07
Cd302	1.7715	5.4161	32.8086	1.01692E-08	3.78199E-07
Mmp25	2.7268	2.3834	32.7698	1.03746E-08	3.85184E-07
Cdc42ep2	1.1880	4.1729	32.6686	1.09289E-08	4.05073E-07
Mefv	4.0533	2.7427	32.6397	1.10927E-08	4.10448E-07
Trim9	4.1231	-0.0341	32.5488	1.16239E-08	4.29376E-07
Plod3	1.0866	6.1959	32.5161	1.18207E-08	4.35907E-07
Milr1	2.3220	1.7689	32.4783	1.20533E-08	4.43737E-07
Mydgf	1.0086	5.5729	32.4576	1.21822E-08	4.47728E-07
Fmnl2	1.8767	4.5530	32.4345	1.23279E-08	4.51929E-07
Lcp1	2.0433	7.3103	32.4329	1.2338E-08	4.51929E-07
Serpina3c	2.0654	4.2385	32.4080	1.24971E-08	4.56682E-07
Scamp5	1.2138	3.7392	32.4061	1.25096E-08	4.56682E-07
Olfm1	1.2626	4.8112	32.3855	1.26429E-08	4.6078E-07
Emp3	1.2309	5.4132	32.3616	1.27993E-08	4.65702E-07
Cdh5	1.6085	5.9752	32.3064	1.31679E-08	4.77523E-07
Basp1	2.0632	3.8746	32.2570	1.35073E-08	4.89017E-07
Coch	1.8861	5.2056	32.2495	1.35593E-08	4.90086E-07
Nckap1l	1.8346	5.5485	32.1747	1.40912E-08	5.0847E-07
Snn	1.3491	4.2627	32.1640	1.41693E-08	5.09931E-07
Slc22a3	3.2349	4.2851	32.1627	1.41784E-08	5.09931E-07
Ly6a	1.2861	7.5932	32.1208	1.44878E-08	5.20203E-07
Mpeg1	2.1496	5.6966	32.0996	1.46466E-08	5.25041E-07
Il11	3.3110	0.9275	32.0639	1.49185E-08	5.3391E-07
Bdkrb2	1.4376	2.6009	32.0431	1.50788E-08	5.38763E-07
Hpx	3.4448	1.5653	32.0204	1.5256E-08	5.44204E-07
Irak1bp1	2.1215	2.4135	31.9639	1.57064E-08	5.59356E-07
Pcolce	1.2258	6.6942	31.9387	1.59118E-08	5.65749E-07
Mapk6	1.3005	6.7981	31.8444	1.67027E-08	5.9265E-07
Rab3il1	1.4464	5.1151	31.8421	1.67227E-08	5.9265E-07
Ednra	1.4309	3.9080	31.8147	1.69604E-08	6.00103E-07
Phf11d	1.3892	2.7463	31.7601	1.74436E-08	6.16199E-07
Apbb1ip	1.3117	4.9027	31.7378	1.76456E-08	6.22328E-07
Fermt3	1.1160	5.0284	31.6955	1.8034E-08	6.35E-07
Xylt1	1.5926	3.3723	31.6889	1.80954E-08	6.36138E-07
Slit2	1.4602	3.9133	31.6840	1.81413E-08	6.36724E-07
Mgp	1.2016	4.3497	31.6562	1.84024E-08	6.4426E-07
Txndc5	1.0647	6.8331	31.6549	1.8415E-08	6.4426E-07
Ccr2	2.8835	5.2416	31.6476	1.84843E-08	6.4565E-07
Sdc3	1.2745	5.8714	31.6360	1.85953E-08	6.48488E-07
Sh2b2	1.5360	3.9525	31.6280	1.86714E-08	6.50022E-07

Marcksl1	1.6376	5.5532	31.6252	1.86988E-08	6.50022E-07
Fes	1.4435	4.1404	31.5789	1.91498E-08	6.64641E-07
Col5a3	1.8670	6.3040	31.4938	2.00081E-08	6.93328E-07
Ap2a2	1.1348	7.4653	31.4284	2.0693E-08	7.15927E-07
Col6a3	2.1829	7.9753	31.3887	2.11203E-08	7.28401E-07
Syk	1.6183	5.9041	31.3791	2.1225E-08	7.30855E-07
Ltf	6.6093	2.2789	31.3564	2.14745E-08	7.38282E-07
Gm9774	2.4235	0.9106	31.3452	2.15993E-08	7.41407E-07
Tfpi	1.7196	3.9844	31.2953	2.21615E-08	7.59509E-07
Cd200r1	2.7664	2.6081	31.2823	2.23099E-08	7.63397E-07
Apaf1	1.2920	3.9719	31.2734	2.24133E-08	7.65736E-07
Cdkl4	4.1216	1.3926	31.2307	2.29113E-08	7.81525E-07
Sp140	1.6861	3.0875	31.1906	2.33893E-08	7.95344E-07
Klhl2	1.1222	4.7781	31.1442	2.39549E-08	8.12049E-07
C3ar1	1.9963	4.4951	31.1136	2.43361E-08	8.23691E-07
Ifit1bl1	4.1454	1.0539	31.1085	2.43996E-08	8.24564E-07
Slc38a1	1.6524	3.5221	30.9989	2.58179E-08	8.69799E-07
Cad	1.0844	5.4969	30.9542	2.64196E-08	8.88701E-07
Podxl	1.4533	5.3397	30.9338	2.66984E-08	8.96696E-07
Cxcl14	1.2626	6.9808	30.8515	2.78543E-08	9.32648E-07
Ralb	1.0251	5.4242	30.8177	2.83447E-08	9.45545E-07
Gsdmc	2.7628	5.2818	30.8131	2.84119E-08	9.45545E-07
Fgl2	1.5459	6.6413	30.8130	2.84127E-08	9.45545E-07
Ehd3	1.2937	3.6458	30.7542	2.92875E-08	9.73173E-07
P2ry6	1.7171	4.2222	30.7017	3.00899E-08	9.98317E-07
Calr	1.2470	8.9685	30.6906	3.02634E-08	1.00255E-06
Orai2	1.4256	3.5699	30.6372	3.11068E-08	1.02737E-06
Nrp2	1.5077	5.4340	30.5763	3.20991E-08	1.05695E-06
Smyd5	1.1297	4.2881	30.5565	3.24288E-08	1.0662E-06
Glipr1	2.1530	2.5655	30.4822	3.36942E-08	1.10281E-06
Cass4	1.7256	2.1906	30.4704	3.39003E-08	1.1079E-06
Ret	1.6846	3.3413	30.4457	3.43343E-08	1.12041E-06
Pik3ap1	1.9119	3.0608	30.4116	3.49434E-08	1.13858E-06
Myo1g	1.3754	4.2496	30.3693	3.57143E-08	1.16024E-06
Mrc1	2.6900	7.8940	30.3534	3.60074E-08	1.16777E-06
Dok2	1.5890	3.9420	30.3305	3.64354E-08	1.1784E-06
Slc12a7	1.0175	5.1701	30.2798	3.73996E-08	1.2078E-06
Col12a1	2.0496	6.3597	30.2618	3.77485E-08	1.21726E-06
Themis2	2.2202	4.5348	30.1295	4.04145E-08	1.29939E-06
Capza1	1.1583	6.0191	30.0353	4.24255E-08	1.36204E-06
Serp1	1.1995	7.0306	29.9947	4.33224E-08	1.3888E-06
Jaml	1.9529	3.4317	29.9354	4.46672E-08	1.42772E-06
Clic4	1.5817	8.0437	29.9043	4.53911E-08	1.44873E-06
Stt3a	1.1011	6.6514	29.8220	4.73583E-08	1.50492E-06
Col5a2	1.6718	7.5667	29.8059	4.77537E-08	1.51528E-06
Cd300lb	2.3161	3.6923	29.7312	4.96288E-08	1.57249E-06
Bmx	2.6111	1.5898	29.7075	5.02394E-08	1.58953E-06
Sned1	1.9066	5.6571	29.6247	5.24333E-08	1.65654E-06
Cd300e	4.6553	0.6475	29.6149	5.26985E-08	1.66018E-06
Pira2	3.5343	2.9503	29.5926	5.33081E-08	1.6769E-06
Gm14548	4.0429	3.7952	29.5841	5.35432E-08	1.68187E-06
Wisp1	2.1293	1.5507	29.5803	5.36479E-08	1.68274E-06
Fcrlb	3.9322	0.0663	29.5613	5.41753E-08	1.69685E-06
Ostc	1.1837	5.7481	29.5472	5.45705E-08	1.70446E-06
Acvrl1	1.0767	5.4382	29.5471	5.45746E-08	1.70446E-06
Ms4a14	2.4493	2.4200	29.3881	5.9241E-08	1.84492E-06
Arhgap30	1.2844	5.0445	29.3017	6.19405E-08	1.9235E-06
Gclm	1.1093	4.6552	29.2734	6.28525E-08	1.94905E-06
Fam49b	1.2999	5.2335	29.1920	6.55495E-08	2.02406E-06

Spidr	1.1261	3.6010	29.1661	6.64311E-08	2.04839E-06
Loxl4	2.0254	3.4073	29.0853	6.92594E-08	2.13259E-06
Tuba1b	1.0171	8.6582	29.0782	6.95156E-08	2.13445E-06
Adgrg3	2.5124	2.6641	29.0746	6.96454E-08	2.13543E-06
Sla	1.8237	3.7535	29.0707	6.97838E-08	2.13668E-06
Lasp1	1.0165	6.5566	29.0626	7.00762E-08	2.14262E-06
Col6a4	4.4610	-0.0761	29.0203	7.16238E-08	2.18688E-06
Icam2	1.4679	2.7864	29.0108	7.19777E-08	2.19461E-06
Sptssb	5.7445	-0.0221	28.9056	7.59937E-08	2.31061E-06
Hyou1	1.2899	7.1351	28.8116	7.97706E-08	2.41871E-06
Trim30a	2.0596	4.4419	28.7554	8.21202E-08	2.48307E-06
Nnmt	2.3747	3.6004	28.7554	8.21207E-08	2.48307E-06
Ifi209	2.6792	1.1990	28.7436	8.26243E-08	2.4914E-06
Esyt1	1.5345	6.2736	28.6250	8.7842E-08	2.63417E-06
Has2	2.1491	2.1869	28.5829	8.97729E-08	2.68838E-06
Gmds	1.3950	2.9269	28.5418	9.16983E-08	2.73479E-06
Actn1	1.0997	6.9810	28.4379	9.67511E-08	2.88155E-06
Rhoh	1.7315	2.2134	28.4110	9.81044E-08	2.91787E-06
Pqlc2	1.2274	3.2327	28.3362	1.01973E-07	3.02506E-06
Elfn1	4.0413	-0.1057	28.3333	1.02124E-07	3.02506E-06
C130050O18Rik	3.2792	0.0777	28.3333	1.02124E-07	3.02506E-06
Pla2g7	2.1631	5.0558	28.3207	1.0279E-07	3.04065E-06
Eme1	1.7000	2.2660	28.2832	1.048E-07	3.09175E-06
Rasgrp2	1.6652	2.2529	28.2690	1.05575E-07	3.1104E-06
Cxcl2	7.7793	6.8179	28.1962	1.09617E-07	3.22081E-06
Awat1	1.5167	4.3518	28.1936	1.09766E-07	3.22084E-06
B4galt2	1.4747	3.2400	28.1757	1.10786E-07	3.24641E-06
Sfxn1	1.1055	5.2802	28.1298	1.13447E-07	3.31548E-06
Ms4a7	2.2599	3.7224	28.1059	1.14854E-07	3.34764E-06
Emcn	1.3130	3.7924	28.0813	1.16322E-07	3.38589E-06
Tpcn2	2.6669	2.6733	28.0733	1.16808E-07	3.39553E-06
Eps8	1.8627	5.1649	28.0682	1.17113E-07	3.39986E-06
Slc5a3	1.5652	3.9968	28.0451	1.18524E-07	3.42726E-06
Cct3	1.0761	7.1402	28.0410	1.18773E-07	3.42981E-06
Txn1	1.1022	7.0290	27.9837	1.22343E-07	3.52824E-06
P2rx1	2.5959	1.1772	27.9646	1.23556E-07	3.5585E-06
Flt3	1.7076	1.9238	27.8972	1.27938E-07	3.67502E-06
Gpr176	2.3948	1.5863	27.8726	1.29574E-07	3.71711E-06
Cfl1	1.2093	8.6017	27.8324	1.32291E-07	3.79009E-06
Thbd	1.7209	6.2706	27.7316	1.39368E-07	3.97718E-06
En1	1.9253	2.0293	27.7046	1.41327E-07	4.02782E-06
Stat4	2.6711	0.9541	27.6603	1.44602E-07	4.11578E-06
Tram1	1.1471	6.3550	27.5568	1.52551E-07	4.32512E-06
Ncf4	1.7610	4.3006	27.5095	1.56326E-07	4.42641E-06
Rbp1	1.8872	4.3458	27.5042	1.56755E-07	4.4328E-06
St3gal1	1.4986	5.8744	27.4939	1.57588E-07	4.45057E-06
Ier5l	1.4211	3.3545	27.4735	1.59259E-07	4.49197E-06
Ccnjl	4.5472	-0.3837	27.4272	1.63124E-07	4.58911E-06
Fmnl1	1.7623	5.5515	27.4141	1.64232E-07	4.61435E-06
Slc16a2	1.0057	5.0536	27.4056	1.6495E-07	4.62856E-06
Fads3	1.6643	5.9490	27.3580	1.69067E-07	4.738E-06
Itgb3	1.4858	4.4600	27.3446	1.70237E-07	4.76469E-06
Nt5dc2	1.5792	4.6304	27.3005	1.7417E-07	4.86229E-06
Glrx	1.4358	7.1941	27.2869	1.75397E-07	4.89029E-06
G0s2	2.7946	5.1597	27.2824	1.75801E-07	4.89528E-06
Tnf	1.7128	3.3150	27.2297	1.80658E-07	5.02414E-06
Cd209e	2.2988	3.1777	27.2225	1.81333E-07	5.03649E-06
Prelp	1.7661	7.2330	27.2076	1.82737E-07	5.06903E-06
Ada	1.4180	3.1019	27.1920	1.84218E-07	5.10364E-06

Ccno	3.4384	0.8005	27.1750	1.85842E-07	5.1421E-06
Serpina3k	4.0802	0.1868	27.1639	1.8692E-07	5.16539E-06
Cytip	1.7556	4.1037	27.1051	1.9269E-07	5.31749E-06
Grem2	2.4059	3.8032	27.1029	1.92912E-07	5.31749E-06
Oaf	1.2798	4.1883	27.0911	1.94092E-07	5.33453E-06
Mybpc3	3.5558	-0.7463	27.0894	1.94263E-07	5.33453E-06
Tnn	1.9834	1.6237	27.0284	2.00486E-07	5.4985E-06
Spon1	2.0340	4.2159	26.9984	2.03621E-07	5.57049E-06
Grem1	1.5045	4.8321	26.9960	2.03881E-07	5.57063E-06
Cd33	3.3081	5.1581	26.9782	2.05762E-07	5.615E-06
Ddost	1.0051	7.1296	26.9614	2.0756E-07	5.65698E-06
Wfdc18	5.0703	2.3539	26.9089	2.13278E-07	5.79835E-06
Igfl3	3.1933	0.8574	26.8568	2.191E-07	5.94922E-06
Ptprc	1.7051	5.8157	26.8115	2.24298E-07	6.07525E-06
Rnasel	1.6036	4.9459	26.7397	2.32793E-07	6.28196E-06
Bhlhe22	2.8911	0.4334	26.7084	2.36591E-07	6.37656E-06
Laptm5	1.1533	7.0085	26.6944	2.38314E-07	6.41508E-06
Mcam	1.6552	5.8020	26.6644	2.42034E-07	6.5072E-06
Rnf213	1.0338	5.3862	26.6588	2.42747E-07	6.51835E-06
B3gnt3	1.6071	2.0257	26.6365	2.45558E-07	6.58572E-06
Ackr1	1.4443	4.6790	26.6114	2.48777E-07	6.66387E-06
9830107B12Rik	3.0016	1.7454	26.5863	2.52022E-07	6.74252E-06
Sec61b	1.1202	5.5111	26.5575	2.55805E-07	6.827E-06
Tmsb10	1.0056	6.8511	26.5504	2.5675E-07	6.84385E-06
P3h1	1.3206	4.5460	26.5174	2.61176E-07	6.95333E-06
Vim	1.2053	9.6477	26.5096	2.62227E-07	6.96676E-06
Olfml3	1.5416	3.2134	26.4942	2.6433E-07	7.0026E-06
Dpy19l1	1.0559	4.1868	26.4737	2.67154E-07	7.05228E-06
Cd209a	1.9100	4.4501	26.4514	2.70255E-07	7.12552E-06
Tnfrsf9	2.1070	1.2169	26.3960	2.78112E-07	7.3062E-06
Klkb1	5.6822	-1.2231	26.3857	2.79604E-07	7.33658E-06
Mcub	1.1085	3.3222	26.3093	2.90884E-07	7.60513E-06
Rsad2	3.4106	3.3988	26.2943	2.93156E-07	7.64623E-06
Stfa3	2.9639	5.2815	26.2686	2.97074E-07	7.7364E-06
Dse	1.3957	5.0199	26.2670	2.97322E-07	7.7364E-06
Gm4070	1.6247	4.5689	26.2358	3.0217E-07	7.85319E-06
Ckap4	1.1673	6.4431	26.2283	3.0335E-07	7.87449E-06
Mgam	4.2413	1.2790	26.1855	3.10135E-07	8.00303E-06
Pde1a	1.9716	3.0753	26.1738	3.12021E-07	8.04219E-06
C5ar2	3.2560	0.7764	26.1362	3.18169E-07	8.18132E-06
Sirpb1a	6.1409	1.4005	26.0254	3.36962E-07	8.6442E-06
Klf7	1.1943	4.7813	25.9967	3.41996E-07	8.75278E-06
Surf4	1.0389	7.8869	25.9888	3.43411E-07	8.7787E-06
Itga1	1.3831	4.5849	25.8321	3.72436E-07	9.49843E-06
Fhl2	1.8918	2.6876	25.7878	3.81092E-07	9.70784E-06
Hgsnat	1.2154	5.0723	25.7502	3.88578E-07	9.8755E-06
Meox1	1.7025	1.6172	25.7355	3.91549E-07	9.93942E-06
Slc16a12	2.1774	2.0244	25.6644	4.06263E-07	1.0289E-05
Bcl6b	1.4459	2.0341	25.6452	4.10312E-07	1.03795E-05
Capn2	1.2003	7.5491	25.5876	4.22739E-07	1.06815E-05
Myd88	1.1291	5.9394	25.5646	4.27811E-07	1.07972E-05
Tubb2a	1.0444	6.9188	25.5570	4.29513E-07	1.08276E-05
Gcnt1	1.4979	2.8355	25.5508	4.30886E-07	1.08497E-05
Ampd3	1.1083	6.0724	25.5225	4.37245E-07	1.09845E-05
Slc26a4	6.1749	-0.8952	25.5203	4.37751E-07	1.09845E-05
Batf3	2.1018	0.1032	25.4765	4.478E-07	1.12109E-05
Slfn3	2.6169	0.3179	25.4112	4.63214E-07	1.15571E-05
Fst	1.5646	5.1015	25.3414	4.80281E-07	1.19419E-05
Sycp2	5.0564	-0.0815	25.2933	4.92418E-07	1.2202E-05

Erich2	4.0226	-0.7711	25.2785	4.96207E-07	1.2282E-05
Gfap	3.0263	0.3650	25.2492	5.03816E-07	1.2428E-05
Ms4a4b	4.0022	-0.0201	25.2418	5.05741E-07	1.24614E-05
Tnfaip2	1.3423	6.0308	25.2009	5.16591E-07	1.27144E-05
Evi2b	2.0781	3.6868	25.1517	5.29932E-07	1.29988E-05
Jph3	4.0274	-0.3956	25.1484	5.30831E-07	1.30062E-05
Aqp3	1.9480	7.6846	25.0744	5.51596E-07	1.34546E-05
Itga7	1.6148	6.6040	24.9600	5.85307E-07	1.41818E-05
Stfa2	3.5939	3.2921	24.9574	5.86107E-07	1.41855E-05
Gmfg	1.8845	3.8139	24.9234	5.96538E-07	1.43901E-05
Gpr182	1.2985	2.6128	24.9027	6.02984E-07	1.45135E-05
Oas1a	1.4300	4.0198	24.8905	6.06817E-07	1.45897E-05
Aldh1l1	1.7851	4.2654	24.8454	6.21171E-07	1.4902E-05
Plekho2	1.3596	5.5322	24.7845	6.41106E-07	1.53465E-05
Ifi44	2.4524	2.7942	24.7738	6.44681E-07	1.53983E-05
Lrp1	1.6097	9.2103	24.7601	6.49269E-07	1.54909E-05
Tlr6	1.8982	3.1294	24.7546	6.51116E-07	1.5518E-05
S1pr1	1.4987	5.1435	24.6766	6.78016E-07	1.60714E-05
Sec61a1	1.0078	7.3044	24.6684	6.80914E-07	1.61226E-05
Ankk1	4.5406	-0.4256	24.6446	6.89371E-07	1.63052E-05
Smox	1.2089	5.8212	24.6280	6.95343E-07	1.64286E-05
Mmrn2	1.3376	4.3720	24.6096	7.02022E-07	1.65506E-05
H6pd	1.7582	6.7240	24.5880	7.09933E-07	1.6701E-05
Fut4	1.7074	0.6917	24.5635	7.18993E-07	1.68778E-05
Hsp90aa1	1.0640	7.7471	24.5196	7.3556E-07	1.72203E-05
Sema7a	1.1650	5.0209	24.4583	7.59338E-07	1.7654E-05
Eln	1.2090	5.6092	24.4305	7.70409E-07	1.78827E-05
Epas1	1.4215	7.6560	24.2857	8.30513E-07	1.91659E-05
Lgals3bp	1.4434	6.5622	24.2817	8.32248E-07	1.91857E-05
A630001G21Rik	2.2645	0.8264	24.2719	8.36493E-07	1.92429E-05
Ripor2	2.1644	3.4442	24.2299	8.54969E-07	1.96265E-05
Galns	1.4918	3.2817	24.2178	8.60336E-07	1.97083E-05
Pdcd1	5.4441	-1.3625	24.1997	8.68475E-07	1.98739E-05
Ddx58	1.4402	3.6414	24.1866	8.74381E-07	1.99881E-05
Ehd4	1.0081	6.9434	24.1755	8.79452E-07	2.0083E-05
Ror1	1.6469	2.6869	24.1020	9.13645E-07	2.07985E-05
Ugt1a7c	1.8822	3.7313	24.0998	9.147E-07	2.08008E-05
Heatr1	1.0743	4.4412	24.0708	9.28557E-07	2.1094E-05
Myadm	1.1033	6.8357	24.0616	9.33046E-07	2.11519E-05
Defb4	5.9509	-1.0630	24.0541	9.36681E-07	2.12123E-05
Sh3tc1	1.8630	3.1807	24.0402	9.43459E-07	2.13437E-05
Tmem106a	1.0748	4.8597	23.9754	9.75723E-07	2.20507E-05
Xdh	1.1963	7.7563	23.9663	9.80366E-07	2.21328E-05
Iigp1	1.2030	4.0230	23.9555	9.85902E-07	2.22347E-05
Loxl3	1.0675	4.1827	23.9404	9.9362E-07	2.23857E-05
Lrrc4	1.9590	1.2653	23.8932	1.01831E-06	2.28476E-05
C1qtnf5	1.6687	2.8472	23.8864	1.0219E-06	2.29047E-05
Eva1b	1.1348	3.5795	23.8362	1.04891E-06	2.34859E-05
Erg	1.2157	3.4444	23.7703	1.08546E-06	2.42794E-05
Nfkbie	1.4096	3.6382	23.7648	1.08855E-06	2.43235E-05
Nfam1	1.7854	4.2498	23.6482	1.15655E-06	2.57756E-05
Ccdc88b	1.4400	3.5298	23.6473	1.15707E-06	2.57756E-05
Lix1l	1.0875	4.9965	23.6238	1.17128E-06	2.60124E-05
Pla2g4e	1.7798	5.4001	23.6098	1.17988E-06	2.61767E-05
Tfec	4.3448	2.1060	23.5961	1.18831E-06	2.63371E-05
Rnf122	1.3036	3.9933	23.5865	1.19421E-06	2.64143E-05
Gpr153	1.1663	4.7685	23.5618	1.20963E-06	2.67282E-05
Ier3	1.4976	6.6914	23.5449	1.22032E-06	2.69371E-05
Mmp1b	6.0871	-0.9679	23.5320	1.22854E-06	2.70913E-05

Olah	3.6121	-0.4469	23.4781	1.26347E-06	2.78333E-05
Serpinb11	2.5937	2.3912	23.4511	1.28129E-06	2.81691E-05
Lif	1.8491	1.7373	23.4420	1.28739E-06	2.82746E-05
Tmem171	2.6771	0.4426	23.4028	1.31387E-06	2.88272E-05
Has3	2.1336	5.4006	23.3743	1.33347E-06	2.92279E-05
Stac2	1.2173	2.5148	23.3462	1.35312E-06	2.96289E-05
Il10ra	1.3982	4.2907	23.2406	1.4295E-06	3.12108E-05
Crocc2	1.9922	0.6830	23.1849	1.47152E-06	3.20605E-05
Lrrc8c	1.2407	4.0670	23.1710	1.48214E-06	3.22599E-05
Oas2	1.1164	4.2458	23.1651	1.48672E-06	3.2295E-05
Enc1	1.1703	4.5874	23.1303	1.51385E-06	3.28518E-05
Sema3g	1.0358	4.4999	23.1270	1.51648E-06	3.28762E-05
P2rx4	1.0519	5.2290	23.1213	1.52093E-06	3.29398E-05
Col13a1	1.7096	1.0028	23.0936	1.54306E-06	3.33172E-05
Fyb	1.5750	3.7262	23.0826	1.55187E-06	3.34441E-05
Igsf6	2.1421	3.5562	23.0196	1.60355E-06	3.44558E-05
Shf	1.0885	3.2103	23.0096	1.61193E-06	3.46018E-05
Clec4a3	1.9064	3.6376	23.0016	1.61865E-06	3.47118E-05
Arpc3	1.0420	7.3118	22.9438	1.66807E-06	3.56667E-05
Ceacam10	6.3833	-0.7259	22.8901	1.71532E-06	3.66051E-05
Rnf149	1.2113	5.3320	22.8632	1.73951E-06	3.70129E-05
Runx2	2.0153	2.3523	22.7665	1.82923E-06	3.86991E-05
Cdk17	1.1048	3.8511	22.7201	1.874E-06	3.94896E-05
Ccl5	2.3797	0.9258	22.7075	1.88627E-06	3.96716E-05
Adgre5	1.2467	6.1503	22.6591	1.93439E-06	4.05419E-05
Itgal	2.0924	3.9732	22.6110	1.9835E-06	4.15165E-05
Plcb1	1.8602	2.7700	22.6060	1.98864E-06	4.15571E-05
Grrp1	1.6735	2.5519	22.6054	1.98924E-06	4.15571E-05
Cd300ld5	1.9338	3.1444	22.5311	2.06765E-06	4.29426E-05
Selplg	1.9604	5.1766	22.5296	2.0693E-06	4.29426E-05
Tuba1c	1.0808	7.4125	22.5296	2.06934E-06	4.29426E-05
Pnp	1.0517	5.7624	22.5152	2.08484E-06	4.32233E-05
Ceacam1	1.7157	3.8317	22.4902	2.11218E-06	4.3707E-05
Hsp90b1	1.0643	8.8375	22.4611	2.14439E-06	4.42801E-05
Gsdmd	1.1280	4.2142	22.4597	2.14596E-06	4.42801E-05
Slc7a5	1.3196	5.3216	22.4526	2.15395E-06	4.44013E-05
Timm8a1	1.2731	4.0315	22.4509	2.1559E-06	4.44013E-05
Gml	4.4264	-1.0161	22.3860	2.22989E-06	4.58388E-05
Ctsk	1.2800	6.4878	22.3381	2.28625E-06	4.69089E-05
Tacr1	1.7720	2.1948	22.2998	2.33231E-06	4.7809E-05
Pdia3	1.0456	8.6195	22.2933	2.34017E-06	4.79252E-05
Sema3a	2.0457	2.6245	22.2437	2.40146E-06	4.91342E-05
Arhgef2	1.0224	5.3527	22.1610	2.50715E-06	5.11458E-05
Slc6a17	1.7612	1.6940	22.1448	2.52835E-06	5.14891E-05
Plpp5	1.1347	2.9868	22.1392	2.53573E-06	5.15914E-05
Rapgef3	1.0091	3.6193	22.1373	2.53834E-06	5.15964E-05
Tnfrsf23	2.4653	2.9755	22.1330	2.54395E-06	5.16622E-05
Mthfd2	1.7294	3.6655	22.1184	2.56338E-06	5.20086E-05
AA467197	3.2825	0.5859	22.1082	2.57704E-06	5.22371E-05
Ass1	1.3469	4.2865	22.0831	2.61097E-06	5.28267E-05
Pitpnm1	1.1418	4.1601	22.0171	2.70237E-06	5.43975E-05
Ap3s1	1.0466	4.7259	22.0162	2.70356E-06	5.43975E-05
Gm20390	1.0563	6.6808	21.9977	2.72977E-06	5.48239E-05
Fmr1	1.2579	5.3801	21.9692	2.77065E-06	5.55938E-05
She	1.5481	3.1743	21.9480	2.80136E-06	5.60041E-05
Mup3	2.5959	-0.0059	21.8437	2.95788E-06	5.90252E-05
Ankrd66	5.9372	-1.0417	21.8362	2.96939E-06	5.92006E-05
2010003K11Rik	2.3716	0.1346	21.7950	3.03396E-06	6.03228E-05
Togaram2	1.2726	3.4672	21.7896	3.04251E-06	6.04377E-05

Inpp5d	1.1027	5.3013	21.7821	3.05439E-06	6.06185E-05
Lpxn	2.1956	2.7206	21.7773	3.06198E-06	6.07141E-05
Reg3g	5.5537	-1.3018	21.7318	3.13556E-06	6.17802E-05
Aldh1l2	1.6925	2.0730	21.6591	3.25661E-06	6.39919E-05
Csf2ra	1.3668	5.0529	21.6461	3.2788E-06	6.43121E-05
Piwil2	1.9105	2.3554	21.6281	3.30972E-06	6.47442E-05
Naip2	1.6339	2.4916	21.6077	3.34502E-06	6.5376E-05
Plekhs1	2.8897	-0.3241	21.5726	3.4068E-06	6.64646E-05
Trim30c	2.4287	-0.0002	21.5262	3.49035E-06	6.7973E-05
Pycr1	1.3259	1.9342	21.4956	3.5464E-06	6.9003E-05
F5	2.4412	1.8050	21.4734	3.58778E-06	6.97461E-05
Ambp	4.7682	-0.7774	21.4619	3.60931E-06	7.01021E-05
Pdss1	1.2209	3.9075	21.4501	3.63158E-06	7.04094E-05
Sat1	1.1295	6.6742	21.2946	3.93849E-06	7.54934E-05
Sema6d	1.3003	4.6232	21.2944	3.93875E-06	7.54934E-05
Tac1	5.2269	-1.4868	21.2531	4.02462E-06	7.69365E-05
S1pr3	1.4494	3.7183	21.2458	4.04001E-06	7.71631E-05
Il21r	2.0489	0.8622	21.2298	4.07372E-06	7.76711E-05
Apbb2	1.3640	3.6132	21.2151	4.10527E-06	7.82042E-05
Casp12	1.2786	3.6140	21.1739	4.19448E-06	7.96258E-05
Dusp2	1.6737	1.2487	21.1532	4.23999E-06	8.02802E-05
Wdfy4	1.6320	3.4410	21.1367	4.27648E-06	8.08311E-05
Ifitm2	1.0969	8.3536	21.1338	4.28308E-06	8.08493E-05
Trmt61a	1.2699	3.6134	21.1313	4.28856E-06	8.08493E-05
Sh3bgrl	1.6481	5.7114	21.0544	4.46417E-06	8.38704E-05
Pirb	2.1994	2.7024	21.0526	4.46848E-06	8.38791E-05
Adam9	1.0265	6.4641	21.0432	4.49047E-06	8.41472E-05
Mup18	2.9766	-0.5022	21.0155	4.55582E-06	8.52986E-05
Fam167a	1.1546	5.0455	20.9929	4.60985E-06	8.62362E-05
Eno1b	1.0352	7.6481	20.9468	4.72227E-06	8.81881E-05
Relt	2.0181	3.1371	20.8422	4.98725E-06	9.274E-05
Tie1	1.1057	4.7200	20.8257	5.03029E-06	9.33018E-05
Disp2	2.8848	0.0352	20.8052	5.08437E-06	9.4145E-05
Kcnk13	1.5585	1.6753	20.8023	5.09205E-06	9.42072E-05
Nfasc	1.6385	3.0401	20.7585	5.21006E-06	9.59028E-05
Cpn1	3.1279	-0.2632	20.7375	5.26751E-06	9.68788E-05
Fam109b	1.3249	2.0047	20.7205	5.31428E-06	9.76566E-05
Socs1	1.3791	2.2521	20.6961	5.38263E-06	9.84981E-05
Casp3	1.1232	4.3843	20.6207	5.5987E-06	0.000102366
Fkbp7	1.0438	3.1919	20.5937	5.67816E-06	0.000103645
Garem2	4.1304	-0.6984	20.5706	5.7472E-06	0.00010472
Ctbp2	1.0130	4.3845	20.5692	5.7514E-06	0.00010472
Arrb2	1.0860	5.1642	20.5566	5.7894E-06	0.000105324
C1qb	1.0510	6.4765	20.5191	5.90382E-06	0.000107226
Plet1	1.3220	6.3234	20.5065	5.94287E-06	0.000107756
Mybl2	1.2843	2.7381	20.4881	6.00024E-06	0.000108616
Pla2g4b	2.1854	3.4754	20.4437	6.14113E-06	0.000110879
Dusp9	2.1839	0.6344	20.4423	6.14558E-06	0.000110879
Gm15056	3.8024	-0.5752	20.4376	6.16075E-06	0.000111061
Man2a1	1.1647	7.0912	20.4166	6.22858E-06	0.000112192
Zfp281	1.2281	3.9963	20.3672	6.39174E-06	0.000114941
Nid2	1.9305	4.7627	20.2715	6.71925E-06	0.000120434
Apobr	1.6628	4.1277	20.2455	6.8112E-06	0.000121682
Rbms3	1.1579	3.9560	20.2033	6.9634E-06	0.000124096
Krt90	3.2890	-0.2334	20.1995	6.97719E-06	0.000124139
Cc2d2b	4.4962	-0.9690	20.1888	7.01621E-06	0.000124465
Nucb2	1.0590	5.3613	20.1552	7.14044E-06	0.000126528
Fth1	1.0992	11.0795	20.1434	7.18484E-06	0.000127212
Htr7	2.5721	0.5570	20.1409	7.19435E-06	0.000127277

Myo1f	1.4154	4.3330	20.1195	7.27497E-06	0.000128391
Hpcal4	5.2268	-1.4867	20.1038	7.33517E-06	0.000129349
Zyx	1.0539	7.2083	20.1015	7.3439E-06	0.000129398
Ly96	1.3392	1.8189	20.0951	7.36834E-06	0.000129724
Traf1	1.5285	2.7075	20.0655	7.48363E-06	0.000131436
Rdh10	1.0748	4.0912	20.0522	7.53579E-06	0.00013214
Clmp	1.1666	5.3549	20.0404	7.58241E-06	0.000132791
Ctsb	1.0035	9.8145	20.0192	7.6669E-06	0.000134115
Tph1	2.5625	2.0515	20.0129	7.692E-06	0.000134447
Renbp	1.0456	3.7696	20.0068	7.71655E-06	0.000134768
Lipg	1.3609	3.7382	19.9835	7.81125E-06	0.000136313
Sec23b	1.3523	5.0108	19.9367	8.00496E-06	0.000139359
Cd200	1.0724	4.4926	19.8192	8.51252E-06	0.000147373
Slc11a1	2.2227	3.5578	19.8157	8.52803E-06	0.000147524
Arl11	1.9050	2.0106	19.7964	8.61454E-06	0.000148903
Dnmt3l	2.4854	0.2299	19.7566	8.79597E-06	0.000151918
Tagln2	1.0121	7.6319	19.7469	8.84072E-06	0.000152571
Was	1.2932	3.3034	19.6935	9.09085E-06	0.000156516
Col15a1	1.4944	5.7307	19.6706	9.20056E-06	0.00015828
Tnfrsf13b	1.6649	1.9984	19.5981	9.55652E-06	0.000163246
Exoc3l2	1.0478	3.2670	19.5923	9.58555E-06	0.000163614
Mup19	6.4914	-0.6546	19.5642	9.72744E-06	0.000165906
B3glct	1.2128	3.3565	19.5431	9.83579E-06	0.000167362
Tsku	1.0966	3.7574	19.5347	9.87909E-06	0.000167968
Dennd3	1.7392	2.9248	19.5125	9.99429E-06	0.000169794
Tmem88	1.5161	2.6305	19.4963	1.00796E-05	0.00017111
Entpd7	1.3970	3.0898	19.4884	1.01212E-05	0.000171683
Flt1	1.3075	5.4501	19.4727	1.0205E-05	0.000172837
Atp6v0d2	3.8332	2.6180	19.4666	1.02374E-05	0.00017325
Ipcef1	3.4742	1.6588	19.4546	1.0302E-05	0.000174209
Il27ra	2.0874	1.0130	19.3802	1.07113E-05	0.000180015
S100a6	1.0490	8.6024	19.2886	1.12377E-05	0.000187848
Npy	3.4681	-0.8022	19.2855	1.12556E-05	0.000187927
Nrcam	2.0966	2.7168	19.2848	1.12596E-05	0.000187927
Fstl1	1.2621	8.2048	19.2575	1.14221E-05	0.000190202
Lrat	2.1617	1.3907	19.2543	1.14412E-05	0.000190375
Itih3	2.0811	-0.3155	19.2463	1.14894E-05	0.000191032
Avpr1a	1.2975	2.7729	19.2034	1.17505E-05	0.000195077
Syde1	1.0319	4.0219	19.1814	1.18865E-05	0.000196736
Lce3c	2.5967	3.8445	19.1672	1.19751E-05	0.000197752
Cpne7	2.5613	0.6394	19.1105	1.23365E-05	0.000203413
Fmnl3	1.2536	3.9921	19.0398	1.28021E-05	0.000210613
Irx1	1.2428	4.2081	19.0197	1.29373E-05	0.000212516
Gml2	5.0709	-1.5669	18.9817	1.3198E-05	0.000216149
Pon1	1.4946	1.3360	18.9387	1.34984E-05	0.000219586
Hspa13	1.0364	4.6953	18.9241	1.36025E-05	0.00022095
Creld2	1.0983	5.4819	18.9186	1.36419E-05	0.000221425
Lhfp	1.3469	4.6889	18.8586	1.40778E-05	0.000227822
Shank3	1.2456	4.4282	18.8127	1.44204E-05	0.000233022
Cd209c	1.0541	2.9389	18.8042	1.44848E-05	0.00023389
Klk12	3.0249	1.0552	18.7866	1.46189E-05	0.000235697
Gapt	2.4272	0.6031	18.7791	1.46763E-05	0.000236108
Irf8	1.2139	3.8383	18.7693	1.47522E-05	0.00023708
Wdr4	1.0382	3.7532	18.7506	1.4898E-05	0.00023897
Ica1	1.3685	1.5165	18.7473	1.49237E-05	0.000239206
Pappa2	1.9061	3.1189	18.7164	1.51675E-05	0.000242403
Adgrg5	3.3262	1.3358	18.7130	1.5194E-05	0.000242471
Mrap	2.4722	3.7368	18.6842	1.54254E-05	0.000245745
Cd274	1.1597	3.7804	18.6314	1.58584E-05	0.000251237

Rptn	2.6981	4.7264	18.6282	1.58855E-05	0.000251484
Apobec3	1.8737	2.0980	18.6213	1.59432E-05	0.000252214
Fbxl5	1.0455	6.1018	18.6150	1.59958E-05	0.000252863
Nfe2	2.3307	2.1519	18.5538	1.65174E-05	0.000259979
Trim6	1.6878	1.1064	18.5241	1.67768E-05	0.000263007
Arhgef15	1.0158	3.6868	18.5232	1.67849E-05	0.000263007
Pcsk5	1.7580	3.9852	18.5013	1.69792E-05	0.000265383
Raet1d	2.5291	0.9593	18.4983	1.70055E-05	0.000265556
Rgs7	2.0103	0.4017	18.4477	1.74634E-05	0.000271734
Chst2	1.4288	2.0060	18.2902	1.89685E-05	0.000293065
Slc26a7	1.8989	1.2833	18.2558	1.93134E-05	0.000297761
Slc1a3	1.7542	4.7208	18.2387	1.94875E-05	0.000299811
Prss12	1.0760	4.1613	18.2274	1.96039E-05	0.000301178
Arid5a	1.1511	4.3213	18.2181	1.96997E-05	0.000301799
Gbp7	1.4822	3.4592	18.2132	1.97506E-05	0.000302233
H2-Q10	3.1798	3.8616	18.2012	1.98755E-05	0.000303853
Lhfpl2	1.7934	4.3529	18.1875	2.00187E-05	0.000305828
Tspan3	1.2000	6.7559	18.1728	2.01736E-05	0.000307785
Ubd	1.5874	0.7536	18.1686	2.02182E-05	0.000308014
Col1a1	1.1992	9.5130	18.1402	2.05226E-05	0.000311998
Serpina3f	1.9550	1.0744	18.1279	2.06558E-05	0.000313804
Srm	1.1670	5.7846	18.1102	2.08486E-05	0.000316293
Sash3	1.4604	3.2674	18.0690	2.13045E-05	0.000322537
Ccnyl1	1.0060	4.6910	18.0395	2.16366E-05	0.000327338
Trabd2b	1.0335	2.7842	18.0362	2.1674E-05	0.000327676
Ddx39	1.1818	5.5416	18.0178	2.18852E-05	0.000330184
Sgpp2	1.4650	2.8600	17.9631	2.25226E-05	0.00033863
Adamts5	1.6272	6.1628	17.9355	2.28513E-05	0.000342821
Bid	3.2274	0.6502	17.9345	2.28642E-05	0.000342821
Stambpl1	1.0157	3.5550	17.8846	2.34715E-05	0.000350377
Btla	2.4733	0.3604	17.8839	2.34804E-05	0.000350377
Ctsc	1.2799	6.8108	17.8550	2.38387E-05	0.00035548
Gm21188	3.5104	0.6661	17.8074	2.44431E-05	0.000362511
P2ry2	1.7825	4.7474	17.7324	2.54257E-05	0.000376063
Gpr183	1.5821	1.8432	17.7077	2.57584E-05	0.000380726
Tmem156	2.5529	1.1706	17.6858	2.60571E-05	0.000384879
Rgl1	1.3317	5.5507	17.6710	2.62606E-05	0.000387623
Dmp1	2.2124	0.7276	17.6482	2.6577E-05	0.000391236
Icos	1.8918	0.2092	17.6454	2.66165E-05	0.000391553
Gne	1.1643	3.6098	17.6436	2.66408E-05	0.000391646
Pcdhgc3	1.3803	6.1698	17.5779	2.75779E-05	0.000404062
Stat5a	1.0235	5.4760	17.5538	2.7929E-05	0.000408658
Wfdc12	2.9314	0.9708	17.5494	2.79933E-05	0.000409325
Cfp	1.1801	6.1424	17.5426	2.80935E-05	0.000410515
Dhrs9	1.1440	4.3704	17.4686	2.92095E-05	0.000425115
Abca13	4.9782	-0.6386	17.4576	2.93795E-05	0.000426485
Thbs4	2.2009	5.9804	17.4574	2.93818E-05	0.000426485
Rasl12	1.3753	2.9061	17.4167	3.0017E-05	0.000434838
Prr16	2.2518	2.2258	17.4116	3.00976E-05	0.000435428
Gm21762	5.8232	-1.1079	17.3360	3.13191E-05	0.000451007
Gpr85	2.6400	0.0407	17.3274	3.14623E-05	0.000452638
Mmp2	1.1208	8.4452	17.3267	3.14738E-05	0.000452638
Tha1	1.3415	2.0447	17.3215	3.1559E-05	0.000453565
Flot1	1.0219	6.3491	17.3016	3.18913E-05	0.000458031
Glra1	2.4615	-0.8013	17.2917	3.20578E-05	0.000459524
Serpina1a	1.8400	0.4581	17.2843	3.2184E-05	0.000461029
Drd1	3.7165	-0.3319	17.2762	3.23206E-05	0.000462683
Lpcat4	1.4347	1.9626	17.2357	3.30173E-05	0.000472038
Ednrb	1.8795	5.9359	17.1750	3.40887E-05	0.000486717

Cd59b	1.4698	1.9266	17.1569	3.44155E-05	0.000489782
Gbp3	1.1429	4.0153	17.1545	3.44593E-05	0.000490085
Camp	3.7798	-1.4019	17.1486	3.45663E-05	0.000491288
Slc24a5	1.2960	1.0465	17.1136	3.52084E-05	0.000499114
Nrros	1.1863	4.9343	17.0770	3.58937E-05	0.00050751
Rab44	1.6929	4.3822	17.0707	3.6013E-05	0.000508868
Ccdc80	1.4907	7.4047	17.0127	3.71307E-05	0.000523982
Lhx1	5.5400	-1.2880	17.0074	3.7234E-05	0.000525101
Tifa	1.3367	3.2088	16.9398	3.85841E-05	0.00054204
Has1	3.1562	2.0043	16.9032	3.93346E-05	0.000551518
Endou	1.4557	6.5964	16.8903	3.96027E-05	0.000554565
Tal1	1.4324	2.0718	16.8620	4.01975E-05	0.000561813
Hsd11b1	1.5409	5.2785	16.8578	4.02879E-05	0.000562716
Bcl2l11	1.2989	3.7393	16.8449	4.05624E-05	0.000566188
Slfn5	1.0895	6.0807	16.8191	4.11182E-05	0.000572482
Serpina9	2.5050	-0.8974	16.8061	4.14008E-05	0.000576049
Apold1	1.0669	3.6174	16.7693	4.22116E-05	0.000585836
Col6a2	1.2952	7.7484	16.7358	4.29616E-05	0.00059511
Ifi44l	1.7258	1.7398	16.7271	4.31593E-05	0.000597092
Dsc2	1.7689	3.2161	16.6712	4.44501E-05	0.000613006
Zfp608	1.1016	3.4776	16.6597	4.4721E-05	0.000615965
Manf	1.2194	6.4220	16.6481	4.49947E-05	0.000618609
Dyrk3	2.0697	0.7493	16.6461	4.50418E-05	0.000618609
Aqp1	1.1729	7.8290	16.6349	4.53099E-05	0.000621723
Sox11	2.3840	0.6384	16.6213	4.56355E-05	0.000625797
Gpr65	2.7611	1.7068	16.4925	4.88426E-05	0.000665179
Fasl	3.2744	-1.2478	16.4659	4.95328E-05	0.000672622
Atp1a3	1.8543	2.6168	16.4491	4.99735E-05	0.000677622
Cnnm2	1.3435	3.3859	16.3899	5.15585E-05	0.000695657
H60b	3.4349	-0.5474	16.3577	5.24426E-05	0.000705842
Smpdl3b	1.1663	2.6728	16.3500	5.26554E-05	0.000707833
Pparg	1.5002	4.3389	16.3134	5.36817E-05	0.000719414
Ly9	1.2417	1.5443	16.2795	5.46522E-05	0.000729732
Samd9l	1.1972	4.7379	16.2611	5.51861E-05	0.000735961
Tgfbr2	1.0833	8.1428	16.2541	5.53886E-05	0.00073776
Cd101	1.4928	0.8245	16.2449	5.56581E-05	0.000740446
Kif17	2.3346	-0.7268	16.2037	5.68827E-05	0.000755357
Chka	1.1248	3.6834	16.1310	5.91096E-05	0.000781127
Celsr3	1.7345	2.0147	16.1135	5.96571E-05	0.000786933
Gbp2	1.0216	4.3412	16.1113	5.97277E-05	0.000787389
Htr2b	2.0351	0.3880	16.0545	6.15462E-05	0.00080892
Clec4a1	1.3328	4.2887	16.0514	6.16453E-05	0.000809364
Armcx2	1.4632	3.2465	16.0441	6.1885E-05	0.000811905
Vstm4	1.1964	2.1045	15.9450	6.52088E-05	0.000849493
Pilrb2	2.1431	1.3749	15.9006	6.67584E-05	0.000866193
Iqgap2	1.0721	4.1370	15.8986	6.68283E-05	0.000866347
Gimap8	1.2784	2.3522	15.8730	6.77377E-05	0.000876056
Fam169b	1.6793	2.5650	15.8678	6.79247E-05	0.000877919
Lep	2.6377	5.5967	15.8564	6.83355E-05	0.00088222
Bik	1.4068	0.8229	15.8494	6.85894E-05	0.000884974
Gbp5	1.4965	1.7149	15.8360	6.90773E-05	0.000889691
Lyve1	1.5412	5.0877	15.8158	6.98161E-05	0.000896562
Rgcc	1.6782	5.7531	15.7842	7.09925E-05	0.000910063
Gm17455	4.6861	-1.7516	15.7672	7.16342E-05	0.000916674
Gja4	1.1044	3.2402	15.7615	7.18492E-05	0.000918349
Loxl1	1.1635	6.6345	15.7393	7.26983E-05	0.000927572
Adcy5	1.6084	4.4150	15.7133	7.37052E-05	0.000938226
Slc2a10	1.7146	2.2030	15.6552	7.60039E-05	0.000964675
Mmp13	4.3601	4.1540	15.6318	7.69487E-05	0.000974966

Bcl2l10	3.9730	-1.2849	15.5970	7.83793E-05	0.000991942
Olfr558	1.6515	0.5386	15.5448	8.05728E-05	0.00101558
Clcf1	1.9513	3.6635	15.4808	8.33491E-05	0.001045143
Tmem263	1.1569	3.6202	15.4761	8.35534E-05	0.001046503
Gm694	2.2647	-0.7725	15.4547	8.45045E-05	0.001055387
Klhl6	1.3047	1.8900	15.4505	8.4693E-05	0.001057138
Fcor	1.8977	1.5996	15.4261	8.57932E-05	0.001067817
Erfe	1.1231	1.4004	15.4097	8.65415E-05	0.001074681
Azin2	1.0228	2.3181	15.3648	8.86225E-05	0.001096157
Prss22	1.1411	3.9438	15.3517	8.9241E-05	0.001098859
Drd2	4.6217	-1.7789	15.3185	9.08231E-05	0.001113284
Tes	1.1273	4.5803	15.3071	9.13708E-05	0.001118672
Bcl2a1d	2.5310	-0.9659	15.2356	9.48967E-05	0.001153503
Msl3l2	1.3020	1.3123	15.2258	9.53929E-05	0.001158888
Igf2bp2	3.4079	1.3485	15.2008	9.66632E-05	0.001172363
Tmem51	1.1029	3.9347	15.1678	9.83651E-05	0.0011897
Trpv2	1.0671	3.6847	15.1535	9.91151E-05	0.001197445
Masp1	1.8514	2.6444	15.1381	9.99268E-05	0.001205915
Rad18	1.2513	1.9964	15.1352	0.00010008	0.001207096
Akna	1.0566	4.5512	15.0959	0.000102185	0.001231128
Tnfsf9	1.2337	1.0265	15.0882	0.000102605	0.001234141
Loxl2	1.3169	5.7627	15.0833	0.000102871	0.001235985
Ifit1	1.7020	3.3997	15.0636	0.000103946	0.001247522
Gprin1	2.3052	-0.2642	15.0245	0.000106127	0.001270899
Lilr4b	4.1766	3.1144	14.9883	0.000108179	0.001290758
Ctse	1.8869	2.9518	14.9880	0.000108199	0.001290758
Pcbp3	1.5101	3.3404	14.9766	0.000108854	0.001297156
Atp8b4	1.4575	2.6394	14.9617	0.000109713	0.001305259
P2ry10	1.3842	1.5884	14.9143	0.000112506	0.001336298
Emilin2	1.4964	6.9119	14.8646	0.00011551	0.001366769
5830411N06Rik	2.1146	0.4812	14.8227	0.000118108	0.001392229
Muc13	3.6693	-1.0067	14.7273	0.000124237	0.001456617
Pcdhb22	1.1921	1.7685	14.7122	0.000125236	0.001466747
Ncapg	1.3853	2.3855	14.7095	0.000125412	0.00146803
Galnt3	1.2274	2.6096	14.7078	0.000125523	0.001468536
Ptprz1	1.7699	4.3021	14.7040	0.000125779	0.00147039
Vill	1.1980	2.8623	14.6943	0.000126427	0.001476744
Kcns3	1.8296	1.8399	14.6870	0.000126917	0.001481673
Slc5a8	1.1512	3.8924	14.6590	0.000128819	0.001496676
Gzmb	2.6361	-0.3759	14.5331	0.000137722	0.001589117
Ercc6l	1.3555	2.3384	14.4946	0.000140563	0.001617625
Trip13	1.3032	1.5160	14.4761	0.000141946	0.001630105
Rrad	3.3826	5.7577	14.4441	0.000144381	0.001652844
Trps1	1.2592	3.7302	14.4117	0.000146886	0.001673631
Bcl2l15	1.9525	1.8299	14.4092	0.000147078	0.001674315
Fndc4	1.1043	2.7678	14.3748	0.000149792	0.001702292
Phf11b	1.3636	1.1580	14.3672	0.000150398	0.001707399
Gnat1	4.7555	-1.7227	14.3446	0.000152219	0.001725456
Hey2	1.5611	-0.0030	14.3355	0.000152957	0.001732851
Ggt5	1.3288	3.3895	14.3332	0.000153137	0.001733319
Mup9	2.2466	1.5215	14.3280	0.000153564	0.001736125
CT571246.5	2.2466	1.5215	14.3280	0.000153564	0.001736125
Pear1	1.4576	3.3480	14.3202	0.0001542	0.00174241
Gper1	1.8597	0.1979	14.2824	0.000157327	0.001774071
Cacna1d	2.5712	0.7072	14.1701	0.000167006	0.001863957
Tmprss11b	2.6456	-0.5018	14.1585	0.000168037	0.001872601
Retnlb	4.8181	-1.6794	14.1416	0.000169554	0.001885646
Trpm2	2.2858	-0.2766	14.1164	0.000171835	0.001909309
Bmper	1.8265	3.0416	14.0841	0.000174819	0.001936312

Dnajb9	1.4091	5.7921	14.0225	0.00018064	0.001989779
Ropn1l	2.0441	-0.1003	13.9978	0.000183028	0.002011298
Phox2a	3.8749	-1.3400	13.9868	0.000184102	0.002020679
Kdelc1	1.2582	3.1379	13.9813	0.00018464	0.002025568
Dbx2	3.9324	-1.3202	13.9617	0.000186574	0.002043698
Lypd1	2.0259	-0.6426	13.9589	0.000186849	0.002045687
Gm11110	1.5655	0.4445	13.8960	0.000193208	0.002105921
Smoc1	1.4455	4.3963	13.8693	0.000195973	0.002131681
Il7r	1.0852	3.6894	13.8365	0.000199421	0.002160576
Trpm1	2.9379	1.8329	13.8063	0.000202655	0.002191264
Myl3	2.0488	-0.6451	13.7620	0.000207491	0.002235801
Nrp1	1.2215	5.1009	13.7327	0.000210753	0.002264245
Lama4	1.4391	6.4547	13.7101	0.000213299	0.002284854
Tmem108	1.6774	0.0135	13.6857	0.000216091	0.002309092
Akr1c13	1.1106	1.9106	13.6521	0.00022	0.002345128
Bcl2a1a	2.3822	-0.0354	13.6460	0.000220713	0.002349618
Stard8	1.0185	5.5545	13.6425	0.000221121	0.002351336
Heg1	1.0071	6.5458	13.5983	0.000226395	0.002396908
Chst7	1.2725	1.8359	13.5925	0.000227095	0.002400045
Unc5c	1.6729	-0.1109	13.5922	0.000227131	0.002400045
Plod2	1.5228	3.3776	13.5501	0.000232279	0.002443587
Btk	1.1579	3.0714	13.5494	0.000232371	0.002443587
Sftpa1	4.4833	-1.8352	13.4994	0.00023864	0.002498676
1700012B09Rik	5.1156	-1.5346	13.4700	0.000242414	0.002530915
Ifit1bl2	2.1435	0.0452	13.4612	0.000243547	0.002540317
Hsh2d	2.1969	-0.5534	13.4583	0.000243928	0.002542112
Stab1	1.9163	5.9631	13.4111	0.000250144	0.002588097
Krt86	5.1253	-1.5333	13.3917	0.000252741	0.00261002
Gprc5b	1.0513	5.2951	13.3585	0.000257256	0.002651629
Gpr4	1.0358	2.6341	13.3518	0.000258177	0.002658614
Defb14	1.4966	3.4103	13.3035	0.000264915	0.002721578
Cd200r4	2.1572	0.6147	13.2664	0.000270207	0.00276554
Fads2	1.2462	2.4756	13.2592	0.000271241	0.002774813
Tpsab1	2.0740	5.7090	13.2486	0.000272778	0.002788944
Golm1	1.0551	3.2647	13.2220	0.000276677	0.002826456
2610528A11Rik	3.0560	3.9678	13.1275	0.000290998	0.002958913
Gal	3.0649	-1.3628	13.0898	0.000296908	0.00300361
Ifi214	5.4764	-1.3193	13.0568	0.000302182	0.003042868
Cdh11	1.2319	2.9166	13.0318	0.000306253	0.003081014
Kcnh1	2.1244	0.7944	13.0271	0.000307012	0.003086045
I830077J02Rik	2.1005	1.3289	13.0168	0.000308704	0.003097108
Pygl	1.3915	5.6035	12.9635	0.000317626	0.003179322
Cxcl10	2.1856	1.8610	12.8925	0.000329896	0.003287068
Mcm10	1.6763	1.8146	12.8681	0.000334237	0.003320583
Gm21320	2.1589	7.7928	12.8380	0.00033965	0.003359719
Cxcr4	1.4826	3.2177	12.8143	0.000343979	0.003394845
Gpr27	1.1512	2.2598	12.7837	0.000349654	0.003436871
Scimp	1.3208	0.8509	12.7481	0.000356373	0.003487217
Tnfaip8l1	1.0559	1.9567	12.6890	0.000367816	0.003578341
Cks1b	1.1737	4.1426	12.6822	0.000369154	0.003586559
Ldlrad3	1.2061	4.5895	12.6810	0.000369396	0.003587319
Slc2a6	1.8079	1.8926	12.6787	0.000369846	0.003590089
Mgst1	1.0047	6.9280	12.6649	0.000372579	0.003606997
Scn5a	1.3676	0.7781	12.6168	0.0003823	0.003691279
B3gnt7	1.5632	0.3910	12.6117	0.000383341	0.003698063
Itga2b	1.1290	2.4187	12.6105	0.00038359	0.003698828
4930519G04Rik	4.3806	-1.8780	12.6072	0.000384262	0.003702045
Lat2	1.2446	1.8259	12.5913	0.000387556	0.003730479
Car5b	1.1792	4.4615	12.5791	0.000390078	0.003753104

Ptgis	1.2888	3.7453	12.5737	0.00039121	0.003762339
Spib	1.4475	0.3681	12.5498	0.000396246	0.003799433
Clvs1	1.2273	3.0136	12.4881	0.000409549	0.003907723
Fyb2	2.3558	-0.8607	12.4853	0.000410172	0.003911958
Dclk1	1.1986	3.6308	12.4747	0.000412505	0.003929705
Cd24a	1.0148	7.1668	12.4209	0.000424551	0.004029727
Fcrla	5.0882	-1.5374	12.4100	0.000427034	0.004040995
Avpr2	1.8838	1.0366	12.4050	0.000428187	0.004048399
R3hdml	5.1862	-1.4912	12.3634	0.000437821	0.004116311
Plscr2	1.3500	1.4363	12.2885	0.00045576	0.004255644
Pilrb1	2.0039	1.0418	12.2780	0.000458341	0.004272434
Rnf125	1.9130	2.4418	12.2529	0.000464524	0.00431717
Slc4a7	1.5580	2.5448	12.2385	0.000468142	0.004345241
Clec12b	2.0049	-0.6732	12.1704	0.000485545	0.004480106
Notch4	1.0065	3.9455	12.1684	0.000486053	0.004482904
Arhgap4	1.8269	2.0807	12.1582	0.00048872	0.004499891
Kcng2	2.6663	-1.2804	12.1549	0.000489586	0.004505964
Tnfsf4	4.3512	-1.8900	12.1291	0.000496414	0.004563035
Ptger2	2.3590	0.7302	12.1146	0.000500288	0.00459285
Pfkfb4	1.1600	4.0091	12.0556	0.000516382	0.004715653
Speer4d	4.4458	-1.8520	12.0552	0.000516473	0.004715653
Nts	5.0244	-1.5806	12.0177	0.000526982	0.004781602
Tox2	1.7335	0.8111	11.9991	0.000532256	0.00482345
Gspt2	1.4363	1.4756	11.9917	0.00053439	0.004838767
Tmem119	1.1038	3.2150	11.9489	0.00054679	0.004936714
Tfpi2	2.2784	0.9691	11.9048	0.000559882	0.005042405
Ifi47	1.1365	2.9098	11.8780	0.000568	0.005100785
Slc25a10	1.0098	4.8172	11.8611	0.000573187	0.005136801
F13a1	1.1926	7.3117	11.8564	0.00057462	0.00514753
Fam46c	1.1683	1.6522	11.8357	0.000581045	0.005196557
Rras2	1.1696	5.3285	11.8122	0.000588436	0.005260502
Lamc2	1.3558	7.0203	11.7774	0.000599553	0.005346746
Nepn	2.8632	-0.7376	11.7638	0.000603951	0.005377185
Sh2d1b1	1.6934	0.0750	11.7503	0.000608343	0.005411876
Atoh8	1.5271	2.4097	11.7449	0.000610111	0.005417755
Kdr	1.0646	4.7167	11.7177	0.000619086	0.005476659
Cd52	1.1772	4.7071	11.6608	0.000638322	0.005616766
Gjc1	1.0027	3.0028	11.6566	0.000639744	0.005620219
Ly6c2	1.0437	3.6036	11.6519	0.000641364	0.005629919
Adam30	2.5623	-1.1077	11.6440	0.000644115	0.005647261
Fcrl1	2.9103	-0.8925	11.6208	0.000652179	0.005701927
Cd6	4.3794	-1.8746	11.6052	0.000657673	0.005738473
Ccnb1ip1	1.5229	0.8137	11.5955	0.000661122	0.00575936
Arxes2	2.0228	3.0697	11.5893	0.000663317	0.005773876
Tg	3.6472	-1.1441	11.5859	0.000664541	0.005782218
Msi1	1.7949	-0.3316	11.5835	0.000665387	0.005784972
Cracr2a	1.5668	1.1475	11.5712	0.000669809	0.005818785
Sox17	1.1541	3.0232	11.5694	0.00067045	0.005821769
St6galnac5	1.4584	4.0942	11.5688	0.000670686	0.005821769
Bend6	1.4946	1.1754	11.5438	0.00067976	0.005884153
Cfi	2.8064	-1.4887	11.4932	0.000698506	0.006013045
Marc1	1.7797	0.5874	11.4720	0.000706527	0.006066093
Spry4	1.2795	2.4832	11.4677	0.000708152	0.006074525
Kcnh4	2.1222	-0.7231	11.4499	0.000714964	0.006116133
Marcks	1.0842	6.3358	11.4092	0.000730831	0.006239641
Frem1	1.8388	0.8212	11.3972	0.000735552	0.006272593
Gbp2b	1.5662	0.0487	11.3617	0.000749755	0.006371313
Fcer1a	2.2793	1.1042	11.3571	0.000751592	0.006383597
Btnl2	1.0370	1.7347	11.3566	0.000751785	0.006383597

Tmem268	1.0229	4.0535	11.3415	0.000757956	0.006425995
Scn3b	1.4701	1.4955	11.3394	0.000758788	0.006430548
Tbc1d10c	1.5286	1.8813	11.2851	0.000781297	0.006593158
Il1rap	1.3059	5.2971	11.2797	0.000783603	0.006609197
Gzma	2.9235	-1.1396	11.2175	0.000810313	0.006790768
Slc10a4	4.3471	-1.8909	11.2072	0.000814827	0.006820739
Ly6i	3.1792	-1.2837	11.1863	0.000824034	0.006876713
Wap	6.2214	-0.8766	11.1812	0.000826312	0.006893093
Fabp4	1.3812	10.0998	11.1757	0.00082877	0.006910952
Sh2d6	2.0964	-0.2969	11.1618	0.000834981	0.006949477
Kcnd1	1.2998	1.4388	11.1397	0.000845012	0.007008912
Sidt1	1.8025	0.2045	11.1307	0.000849127	0.007040373
Fbxo39	4.1866	-1.9500	11.1198	0.000854111	0.007079005
Rbm46	2.6306	-0.8619	11.0970	0.000864692	0.007145004
Havcr1	4.4617	-1.8369	11.0927	0.0008667	0.007154061
Gimap5	1.4272	0.6247	11.0879	0.000868951	0.00716454
Mlph	1.0198	2.8904	11.0237	0.000899548	0.007377174
Moap1	1.1191	1.1336	11.0217	0.000900539	0.007379751
Cd1d1	1.6600	3.7219	11.0131	0.000904703	0.007401157
Epsti1	1.0074	2.9440	10.9333	0.000944495	0.00766801
Elf3	1.1664	2.1282	10.9253	0.000948615	0.007698596
Pstpip1	1.1508	3.3513	10.9037	0.000959711	0.007775182
Npy2r	4.2088	-0.6643	10.9035	0.000959804	0.007775182
Arhgap9	1.0107	4.0091	10.8964	0.000963496	0.007787516
Cep55	1.2071	2.6856	10.8928	0.000965382	0.007799871
Adm2	1.9532	-0.2368	10.8410	0.000992788	0.007975396
C1rl	1.4396	2.6695	10.8327	0.000997229	0.008003859
P2ry12	1.9143	1.8982	10.8251	0.00100136	0.008031111
Rgs8	4.1726	-1.9521	10.7570	0.001038881	0.008292432
Slco4c1	2.8507	-0.3844	10.7425	0.001047036	0.008354474
Bfsp1	1.7868	2.0762	10.7215	0.001058999	0.008425291
Eno2	1.1851	2.5291	10.7121	0.001064391	0.008452788
Sncg	1.1026	5.4919	10.6718	0.00108781	0.008601224
Ikbip	1.0466	3.4843	10.6563	0.001096944	0.008657766
Gm12253	2.7165	-1.2478	10.6504	0.001100476	0.008673104
Zfp697	2.5314	2.5955	10.6109	0.001124257	0.008809641
Fbxo27	1.1262	1.2512	10.6005	0.001130548	0.008849408
Tbx2	1.0517	2.0347	10.5918	0.001135933	0.008885185
Foxs1	1.4111	0.4248	10.5871	0.001138816	0.008891804
E2f7	1.1211	2.8109	10.5462	0.001164276	0.009054141
Gulp1	1.2916	2.1569	10.5422	0.001166812	0.009061781
Olfr1378	2.2368	-0.6509	10.5380	0.001169469	0.009079189
Gm6594	4.3239	-1.8933	10.5008	0.001193209	0.009233936
Xylb	6.0041	-0.9870	10.4387	0.001234053	0.009492815
Dock2	1.0489	4.4689	10.4289	0.001240564	0.009529473
Cd300ld3	5.9595	-1.0157	10.3555	0.001290862	0.009846544
Lipa	1.1208	5.3843	10.3047	0.001326895	0.010079142
Cmklr1	1.2299	4.9959	10.2707	0.001351574	0.010238103
Capn12	1.1013	3.2088	10.2592	0.001360049	0.010277338
Chrm3	1.4923	-0.3907	10.2472	0.001368887	0.010333395
Blk	1.7166	-0.2866	10.2392	0.001374835	0.010367539
Fbxl22	1.4212	2.0269	10.2193	0.001389771	0.010455995
Map7d3	1.8677	0.4189	10.2125	0.001394925	0.010486435
Mup7	2.2540	1.7735	10.1713	0.00142643	0.010681157
Retn	1.3577	5.6560	10.1349	0.001454906	0.010851086
Strip2	1.3372	0.9816	10.1250	0.001462728	0.010887199
Myo7a	1.3985	1.9731	10.1205	0.001466301	0.010899152
Samd12	2.7486	-1.5106	10.1057	0.001478131	0.010975877
Astl	3.2975	-1.2140	10.0456	0.001527128	0.011263085

Spc25	1.0350	2.1917	10.0272	0.001542415	0.011345166
Etv1	1.3233	2.1789	10.0251	0.001544177	0.011350477
Snap25	4.8994	-1.6709	9.9973	0.001567727	0.011474562
Elf5	1.7814	3.0385	9.9481	0.001610139	0.011740465
Kmo	1.8075	1.6356	9.9337	0.00162278	0.01180897
Adap1	1.7292	1.8630	9.9294	0.001626593	0.011822905
Lao1	5.3106	2.3525	9.8600	0.001689126	0.012194218
Pik3cg	1.3271	2.9099	9.8498	0.001698496	0.012241622
Tmem200b	1.1406	1.7004	9.8436	0.001704257	0.012258861
Gm4951	1.3797	1.3724	9.8377	0.001709704	0.01229399
Hey1	1.3529	1.9708	9.8256	0.001720994	0.012352142
Aadacl4	1.0773	3.4758	9.7642	0.001779393	0.012694746
Gm45692	3.5750	-1.8354	9.7580	0.001785418	0.012729409
Tlr8	1.9881	2.8083	9.7496	0.001793612	0.012771152
Gm19345	2.9507	-1.4053	9.7317	0.001811167	0.012879343
Hoxd10	1.3713	1.1299	9.7297	0.001813098	0.012888877
Sp9	3.9954	-2.0137	9.7258	0.001816951	0.012899466
Rprml	2.2591	-0.1968	9.7212	0.001821568	0.012926851
Mup17	2.2565	4.2688	9.7154	0.001827282	0.012955957
Enpp2	1.1007	5.7934	9.7070	0.001835667	0.013000358
Dnah7a	4.1266	-1.9654	9.6754	0.001867541	0.013181467
Lin7a	1.5571	-0.3806	9.6464	0.001897229	0.013347857
Olfr870	4.2121	-1.9467	9.6415	0.001902297	0.013374894
Ttpa	1.4039	1.6647	9.6310	0.001913167	0.013442496
Sprr3	3.0946	-0.5612	9.6059	0.001939564	0.013597502
Hc	3.6653	-0.6201	9.6032	0.001942429	0.013608848
Adam32	4.2125	-1.9479	9.5960	0.001950018	0.013644503
Gm7879	1.0611	0.7863	9.5574	0.001991486	0.013864644
Rps26	1.1468	1.5368	9.5468	0.002002968	0.013925728
Gpr55	1.9091	0.1492	9.4812	0.002075894	0.014291656
Nfil3	1.2723	4.7769	9.4607	0.002099266	0.014434352
Bag2	1.0152	3.4714	9.4423	0.002120337	0.014570058
Pacsin1	1.8521	-0.7490	9.4276	0.002137478	0.01465784
Tdgf1	3.9908	-2.0141	9.4150	0.002152156	0.014733059
St8sia4	1.6039	3.0211	9.4065	0.00216218	0.014797036
Ankrd10	1.1708	4.9014	9.3947	0.00217615	0.014864683
Enkd1	1.2451	1.1538	9.3848	0.002187904	0.014926989
Aqp5	2.1151	0.9958	9.3787	0.002195271	0.014959079
Oit1	4.0058	-2.0120	9.3569	0.002221527	0.015110341
A930003A15Rik	2.4093	-1.0043	9.3555	0.002223159	0.015114853
P2ry13	1.2223	2.2682	9.3187	0.002268281	0.015388077
Dll4	1.1618	2.4123	9.3105	0.002278442	0.015428248
Prr5l	1.3785	1.8892	9.2895	0.002304772	0.015587199
Nova2	1.2525	1.8922	9.2779	0.002319299	0.015656344
2210407C18Rik	5.3734	-1.3689	9.2762	0.002321475	0.015666192
Bub1	1.1876	2.6081	9.2613	0.002340462	0.015745648
Ntng2	1.1876	2.4190	9.2266	0.002385295	0.015997964
Hist1h3c	3.7479	-1.4161	9.2150	0.002400388	0.016079435
Ptgfr	1.2430	4.6011	9.2001	0.002420037	0.016176311
Lin28a	2.9234	-0.1883	9.1751	0.002453274	0.016358404
Gm16486	3.8988	-2.0451	9.1745	0.002454134	0.016359147
Areg	1.0673	3.1551	9.1620	0.002470924	0.01643093
Orc1	1.0583	2.1708	9.1572	0.00247746	0.016469377
Prkar2b	1.2946	5.1729	9.1417	0.002498457	0.016573627
Prkd1	1.0402	2.2723	9.1363	0.00250594	0.016618216
Hoxd4	1.0196	1.8742	9.0685	0.002600546	0.01714664
Tspan32	1.0461	1.9447	9.0424	0.002637849	0.017350673
Nox3	3.1035	-1.7273	9.0293	0.00265683	0.017438742
Slamf1	2.6282	-1.3053	9.0286	0.002657861	0.017440267

Serpina1b	1.1662	2.3444	9.0264	0.00266106	0.017450768
Tirap	1.1408	4.5672	9.0228	0.002666344	0.017474919
Gm2000	1.3983	4.6921	9.0194	0.002671294	0.017489981
Plin1	1.8896	6.3963	9.0190	0.002671847	0.017489981
Cadps2	1.2940	0.6802	9.0163	0.002675758	0.017505088
Sox5	1.1626	1.2747	8.9743	0.002738007	0.017864681
Gucy2c	1.9736	-1.0723	8.9642	0.002753208	0.01793117
Acot5	1.1210	1.4986	8.9588	0.002761409	0.017973861
Proz	1.1826	0.2400	8.9129	0.002831601	0.018348673
Tbc1d30	1.3609	0.0871	8.9051	0.002843701	0.018410686
Gpsm3	1.1162	3.6401	8.9021	0.002848432	0.018430384
Kitl	1.0362	5.0411	8.8854	0.002874612	0.018536936
4933415A04Rik	4.4292	-1.8381	8.8794	0.002884033	0.018566014
Mov10	1.0386	3.1643	8.8719	0.002895959	0.018621997
Gbp4	3.7083	-0.4330	8.8665	0.002904477	0.018665772
Cxcr1	3.9830	-2.0145	8.8618	0.00291199	0.018699962
Ccl17	1.7926	-0.5921	8.8550	0.002922822	0.018729137
Gca	1.3082	1.7606	8.8358	0.002953762	0.018876925
Glt1d1	1.4006	-0.1821	8.8318	0.002960193	0.018909139
Igfals	1.3455	1.8485	8.8263	0.002969122	0.018936284
Cox4i2	1.6922	0.2073	8.7940	0.003022258	0.019216828
Gfra2	1.0420	3.7341	8.7937	0.003022793	0.019216828
Pid1	1.0143	4.2548	8.7813	0.003043396	0.019330925
Mcpt8	4.1024	-1.9779	8.7696	0.003062863	0.019426312
Pcdhb14	1.0869	0.9307	8.7421	0.003109474	0.019664436
Ccr3	1.9916	-0.4602	8.7234	0.003141527	0.019827316
Cda	1.6322	-0.3201	8.6931	0.00319418	0.020093304
0610040J01Rik	2.1009	0.2866	8.6835	0.003211027	0.020143658
G530012D18Rik	1.7485	-0.4880	8.6582	0.00325595	0.020349454
Abcd2	1.4688	5.4792	8.6563	0.003259351	0.02036488
Aldh1a7	1.3499	1.9881	8.6286	0.003309274	0.020635461
Ppp1r3c	1.5950	5.8872	8.6265	0.003313013	0.02065288
Cd69	1.2901	0.2816	8.5870	0.00338562	0.021009517
Cldn2	4.5652	-1.7834	8.5680	0.003421136	0.02118174
Zeb2	1.0521	5.6370	8.5673	0.003422611	0.021184863
Rasgef1a	1.3092	0.9874	8.5571	0.003441796	0.021276351
Arsj	1.4618	0.7401	8.5524	0.003450757	0.021298694
Fbn2	1.5802	0.6653	8.5421	0.003470345	0.02137066
Kng2	1.0777	1.0843	8.5368	0.003480331	0.021414628
Ikzf3	1.4510	-0.1660	8.4991	0.003553163	0.021758512
Izumo1r	3.7942	-2.0773	8.4975	0.003556406	0.02176616
Zfand4	2.1971	1.1179	8.4966	0.003558163	0.02177081
Best1	1.2475	0.9403	8.4882	0.003574594	0.02185909
Foxf2	2.4589	-1.4922	8.4870	0.003576944	0.021861949
Col6a5	1.1365	1.6520	8.4778	0.003595069	0.021959693
Cacna1b	2.7752	-1.5069	8.4772	0.00359633	0.021960536
Vnn3	1.5877	1.8927	8.4727	0.00360511	0.02199641
Shroom1	2.7970	-0.1831	8.4636	0.00362321	0.022088327
C430049E01Rik	3.0915	-1.7288	8.4532	0.003643993	0.022165512
Ceacam16	2.0933	-0.6058	8.4381	0.003674498	0.022325374
Trim46	1.1512	1.1747	8.4130	0.003725472	0.022560552
Zwilch	1.1899	2.3379	8.3937	0.003765177	0.02278835
Mup2	4.3819	-1.8626	8.3825	0.003788588	0.022904643
Fbln2	1.1789	7.0566	8.3722	0.003810031	0.022964327
Chtf18	1.2070	1.9133	8.3640	0.00382732	0.023049438
Hbegf	1.6713	5.5015	8.3563	0.003843497	0.023113388
Clstn2	1.5201	0.3720	8.3519	0.003852782	0.023148203
Xab2	1.3261	4.1155	8.3435	0.003870716	0.023214726
Muc5ac	3.8029	-2.0760	8.3355	0.003887703	0.023279634

Nrxn3	4.3331	-1.8826	8.3250	0.003910218	0.023392611
Uchl1	4.5352	-1.8305	8.3086	0.003945672	0.023515688
Fam71a	4.2210	-1.9479	8.3050	0.003953637	0.023543878
Kcnj9	2.0399	-1.1929	8.3036	0.003956706	0.023555726
Gm15448	2.8239	-0.5368	8.2870	0.003993014	0.023694346
Cldn17	3.7941	-2.0773	8.2712	0.004027962	0.023841181
Lrmda	1.0320	1.0183	8.2474	0.004081149	0.024086389
Hoga1	1.5060	2.7257	8.2467	0.004082714	0.024089118
Sik2	1.1424	3.1263	8.2405	0.004096535	0.024151079
Pag1	1.0842	3.6630	8.2143	0.004156181	0.024456492
Pcdhga3	1.7107	-0.1916	8.2123	0.004160817	0.024477176
Rgs1	2.2604	-1.4721	8.1996	0.004189955	0.024608801
Gdf3	2.8691	-0.9016	8.1798	0.004235841	0.024831536
Pbk	1.2444	2.8370	8.1525	0.00430028	0.025135049
Npr3	1.4215	4.9179	8.1092	0.00440423	0.025617948
Gm26558	1.1766	0.3964	8.0749	0.004488259	0.02600403
Cpsf4l	2.9187	-0.3379	8.0701	0.0045002	0.026065239
Cdhr2	3.7835	-2.0785	8.0548	0.004538285	0.026224273
Pah	3.7873	-2.0781	8.0473	0.004557254	0.026306033
St6galnac4	1.3005	4.4871	8.0418	0.004571108	0.026344206
Ccdc125	1.3131	1.2019	8.0295	0.00460215	0.026474186
Gm379	3.8019	-2.0764	8.0197	0.004627144	0.026601079
Gm4758	3.8019	-2.0764	8.0197	0.004627144	0.026601079
Gm14085	1.3065	1.9868	8.0098	0.004652405	0.026699963
Aunip	1.2433	-0.2411	8.0003	0.004676982	0.026780904
Hrasls5	3.2113	-1.6780	7.9961	0.004687784	0.026815621
Pcdhgb5	1.3679	1.1636	7.9865	0.004712726	0.026918766
Trf	1.8206	8.8543	7.9684	0.004760222	0.02714744
Ms4a2	1.4102	1.8180	7.9406	0.004833768	0.02746642
Ccrl2	1.2973	3.0298	7.9303	0.004861263	0.027572418
Nup210l	1.2640	-0.0258	7.9216	0.004884849	0.027684615
Pcdh12	2.7915	0.2921	7.9118	0.00491131	0.027811264
Shcbp1	1.0381	2.5224	7.8734	0.005016763	0.028278069
Pinlyp	1.3720	3.0819	7.8480	0.005087789	0.028611934
Pcdhb7	1.2900	0.3528	7.8263	0.005149082	0.028911939
Donson	1.1011	2.2655	7.8155	0.005179913	0.029055161
Raet1e	1.6281	-0.3061	7.8128	0.005187672	0.029080999
Nog	1.0690	1.9242	7.7886	0.005257578	0.029407685
Hnf4a	3.1023	-0.8152	7.7803	0.005281964	0.029483651
Kcnj10	2.4945	-1.6258	7.7591	0.005344274	0.029785767
Slc1a2	1.6806	-0.1171	7.7522	0.005364757	0.029877042
Gm867	3.1516	-1.4131	7.7491	0.00537391	0.029912756
Pram1	2.5006	2.0072	7.7443	0.005388137	0.029961391
Gsdmc4	1.9997	0.1011	7.7423	0.005394193	0.029979795
Antxrl	2.5498	-0.9214	7.6943	0.005539609	0.030678642
Ctla4	1.9057	-1.1053	7.6747	0.005600162	0.030959012
Grip1	1.6658	1.0391	7.6256	0.0057545	0.031671876
Sdk1	1.1465	3.4418	7.6108	0.0058019	0.031872492
Prss27	1.0637	3.8153	7.5918	0.005863288	0.032104698
Klk1b11	3.7709	-2.0794	7.5878	0.005876518	0.032156402
Ltb	1.4392	1.7356	7.5871	0.005878622	0.032156402
Htr2a	1.3621	-0.5762	7.5854	0.005884429	0.032172042
Itgb8	1.3506	2.6444	7.5720	0.005928114	0.032348901
Apoc3	3.9792	-2.0141	7.5587	0.005972234	0.032521786
Rtp3	1.1005	1.8631	7.5488	0.00600502	0.032675871
Slc4a3	1.0924	1.3306	7.5471	0.006010553	0.032697828
Krt84	2.1743	0.0649	7.5337	0.00605554	0.032868843
Gm45717	2.1565	-0.2657	7.5325	0.006059445	0.032873695
Agxt	4.1202	-1.9846	7.5223	0.006094134	0.033020861

Gm10130	3.8143	-2.0756	7.5182	0.006107815	0.033070365
Olfr466	1.9523	-1.2709	7.5101	0.006135234	0.033185899
Ccdc167	1.0892	2.0181	7.4642	0.006293756	0.033808805
Mrgprx2	1.5383	1.5989	7.4573	0.006318067	0.033897695
Pcdhb5	1.1228	-0.1532	7.4538	0.006330347	0.033938557
Slc13a3	1.3672	-0.5779	7.4468	0.006354797	0.03404456
Slc22a20	4.1239	-1.9546	7.4387	0.006383639	0.03415716
Asf1b	1.0018	3.3703	7.4372	0.006388907	0.034176973
Hcar1	1.8011	2.4722	7.4332	0.006403163	0.034214079
Glycam1	5.0636	-1.5765	7.4319	0.006407658	0.034218588
Prrt4	1.0864	0.1848	7.4247	0.006433457	0.034314389
Tusc5	1.1913	5.1801	7.3298	0.006781944	0.035823038
Cmpk2	1.1057	2.4638	7.3191	0.006822629	0.035959635
Il10	1.8678	-0.8613	7.3183	0.006825434	0.035965738
Cldn7	2.7304	-0.1986	7.3040	0.006879965	0.036174497
Ppm1e	1.1343	-0.2344	7.2914	0.006928355	0.036332671
Hdx	1.9582	-1.2033	7.2898	0.006934682	0.036348389
Vsig2	1.0415	1.5903	7.2890	0.006937829	0.036356157
Gpr173	1.3276	-0.4580	7.2713	0.007006525	0.036576776
Isg15	1.3818	3.7689	7.2614	0.007045042	0.036732819
Spata33	1.1751	0.0925	7.2219	0.007201762	0.037380328
Il23r	1.9709	-1.4031	7.2057	0.007267298	0.037622098
Dusp4	1.2426	2.8351	7.2050	0.00727025	0.037628454
Krt18	2.5864	2.9984	7.1987	0.0072955	0.037741246
Gphb5	4.8110	-1.7209	7.1759	0.00738875	0.038160353
Pxdc1	1.3783	2.1850	7.1624	0.007444873	0.038386637
Apol6	1.0806	4.9749	7.1444	0.007519843	0.038672718
1700012B07Rik	3.7711	-2.0789	7.1150	0.007644206	0.039229117
Tigit	2.0589	-1.0359	7.1139	0.007648693	0.039242916
Ifitm5	1.3560	0.4515	7.1119	0.007657516	0.039269729
Glis1	2.2905	-0.7576	7.0995	0.007710606	0.039467817
Folr1	2.5394	-0.1052	7.0900	0.007751479	0.03964765
Rasd1	1.0237	3.9106	7.0750	0.00781656	0.039833282
Pcdhb18	1.3875	0.0548	7.0734	0.007823883	0.039842126
Chil5	2.8746	-0.6996	7.0704	0.00783659	0.03988217
Reep2	1.0128	0.3658	7.0598	0.007883466	0.040042799
1700016H13Rik	2.4065	-1.4524	7.0397	0.007972017	0.040389209
4931406B18Rik	1.8265	-1.1296	7.0341	0.007997053	0.040492333
Scg5	2.9649	-1.7798	7.0205	0.008058116	0.040712041
Ces2c	2.8066	-1.8361	7.0161	0.008078185	0.040785122
B3galt5	1.6663	-0.8687	7.0135	0.008089515	0.040823439
Olfr1346	3.5500	-2.1444	6.9838	0.008225111	0.041316703
Pask	1.1369	1.3716	6.9654	0.008309868	0.041665764
Ppp1r3d	1.0621	2.7183	6.9643	0.008315347	0.041683661
Rnf207	1.3552	-0.4574	6.9626	0.008322943	0.041702588
Slc51b	3.5444	-2.1459	6.9578	0.008345339	0.041745892
S100a2	3.5444	-2.1459	6.9578	0.008345339	0.041745892
Ndc80	1.0514	2.0263	6.9575	0.008346884	0.041745892
6820408C15Rik	3.5574	-2.1442	6.9549	0.008358793	0.041786307
Oasl2	1.5761	2.7851	6.9071	0.008585504	0.042684349
Pdyn	3.7576	-2.0798	6.9029	0.008605503	0.042745558
Gm10306	2.3690	-1.6755	6.8923	0.008656982	0.042962169
Fam222a	1.5902	-0.5283	6.8578	0.008825572	0.043669781
Gm3448	1.2779	0.1092	6.8227	0.009000799	0.044365874
Pcdhb11	1.0905	0.5024	6.8070	0.009080112	0.0446719
Ms4a3	3.6646	-2.1097	6.8065	0.009082705	0.0446719
Tnfsf11	2.1135	-0.7291	6.8006	0.009112609	0.044765384
Foxi1	4.7133	0.7174	6.7863	0.0091858	0.045043845
Spata22	3.5351	-2.1468	6.7767	0.009235573	0.045250033

Olfr222	3.5386	-2.1464	6.7748	0.009245305	0.045274621
Gm6583	3.5506	-2.1450	6.7692	0.009274223	0.045385688
Fbp1	1.6895	-0.4977	6.7664	0.009288972	0.045437496
Hist1h2ah	3.8188	-2.0730	6.7434	0.009409445	0.045893117
Spdef	3.2701	-1.6414	6.7347	0.009455621	0.046056594
Ticam2	1.0372	1.9936	6.7286	0.009487949	0.046177829
Wee2	4.1119	-1.9550	6.6942	0.009672476	0.046872324
Fndc8	1.4477	-0.7775	6.6829	0.009734256	0.047119411
Kcnk1	1.1560	1.1949	6.6818	0.009740049	0.047137
Triqk	1.0343	0.3244	6.6758	0.009773016	0.047223266
Daam2	3.5394	-2.1508	6.6729	0.009788852	0.047278856
Erc2	3.6082	-2.1253	6.6144	0.010115703	0.048535364
Fgl1	3.5242	-2.1545	6.6066	0.010160427	0.048728531
4930555G01Rik	3.7391	-2.1013	6.6059	0.010163965	0.048734797
Gpr156	1.9426	-0.1615	6.5967	0.010216973	0.04893522
Cma1	1.2382	6.0156	6.5955	0.010223744	0.048948518
D13Ertd608e	3.5025	-2.1523	6.5753	0.010340312	0.049331152
Gm10406	3.5069	-2.1537	6.5737	0.010349793	0.049345839
Cited1	3.9313	1.2857	6.5734	0.010351665	0.049345839
Madcam1	2.1207	-1.5362	6.5557	0.010454586	0.049735031

Supplementary Table S4. The genes downregulated in the SSS samples compared with the NT controls

Gene	logFC	logCPM	LR	PValue	FDR
4933427D14Rik	-5.2391	0.2816	54.8263	1.3167E-13	1.1906E-11
Rorc	-1.3455	6.0386	52.9384	3.4418E-13	2.8899E-11
Wnt10b	-2.9178	2.5047	52.0807	5.3265E-13	4.3068E-11
Satb1	-1.2619	5.5994	48.2438	3.7638E-12	2.7298E-10
Tmem45a2	-3.3214	0.1891	46.9235	7.3813E-12	5.0515E-10
Krtap3-3	-3.1779	1.7101	46.7624	8.0134E-12	5.4499E-10
Ccer2	-2.9858	1.8896	46.6744	8.3815E-12	5.6825E-10
Slc25a27	-1.4346	3.8218	44.7700	2.216E-11	1.3861E-09
Csrnp2	-1.2997	4.2381	43.4383	4.3754E-11	2.6314E-09
Cyb5r2	-2.1565	2.2618	42.8518	5.9047E-11	3.522E-09
Scgb1a1	-3.5440	1.2279	42.2616	7.9843E-11	4.7237E-09
Bmp4	-1.6308	4.0981	42.1515	8.447E-11	4.9705E-09
Pick1	-1.0835	5.0150	41.0713	1.4678E-10	8.1951E-09
Tmem177	-1.8080	4.6565	40.3297	2.1453E-10	1.1708E-08
Calcoco1	-1.2109	7.0061	39.4388	3.385E-10	1.7936E-08
Dyrk1b	-1.4797	5.6700	38.7624	4.7867E-10	2.453E-08
Cbx7	-1.6378	5.5652	38.7556	4.8032E-10	2.4557E-08
Axin2	-1.5406	4.9208	38.4489	5.6205E-10	2.8078E-08
Arrdc3	-1.2162	7.2773	38.1097	6.6876E-10	3.3333E-08
Pik3ip1	-1.6332	5.3991	37.4791	9.2399E-10	4.4826E-08
Snx21	-1.3054	4.8278	37.1300	1.1051E-09	5.2562E-08
Tfap2b	-1.3047	4.8892	36.9594	1.2062E-09	5.6145E-08
Ppfibp2	-1.3830	5.8148	36.3979	1.6088E-09	7.3785E-08
Homer2	-1.3586	5.2486	35.9221	2.0536E-09	9.2059E-08
Sox6	-1.2009	5.5125	35.7331	2.2629E-09	1.0021E-07
Fam171b	-1.6564	2.5253	35.6843	2.3203E-09	1.0254E-07
Tfap2e	-2.0473	3.3202	35.6476	2.3644E-09	1.0407E-07
Retnla	-1.7570	7.6261	35.2876	2.8444E-09	1.2248E-07
Arrdc2	-1.3586	4.1782	35.1613	3.035E-09	1.2992E-07
Fam214a	-1.3222	5.9363	34.6034	4.0419E-09	1.657E-07
Eps8l3	-1.7165	2.9613	34.1002	5.2345E-09	2.1123E-07
Ulk1	-1.0727	6.8763	33.9261	5.7245E-09	2.2805E-07
Zranb1	-1.0446	6.6199	33.7560	6.2477E-09	2.4664E-07
Ephx1	-1.1368	5.9110	33.6093	6.7369E-09	2.6357E-07
Camk2n1	-1.2693	3.6410	33.3883	7.5478E-09	2.9112E-07
Syngr1	-1.2378	4.5280	32.9798	9.3121E-09	3.505E-07
Slc17a8	-2.4223	1.4480	32.9394	9.5078E-09	3.5603E-07
Trim7	-1.7527	5.7053	32.8092	1.0166E-08	3.782E-07
Fgfr2	-1.3438	6.0283	32.3182	1.3088E-08	4.7543E-07
Osbp2	-2.0479	1.7610	31.4095	2.0896E-08	7.2179E-07
Ypel3	-1.0495	6.9393	31.2205	2.3033E-08	7.8444E-07
Plcd3	-1.0971	4.4217	31.1445	2.3951E-08	8.1205E-07
Slc37a1	-1.6490	3.5040	31.0379	2.5304E-08	8.5381E-07
Gm10036	-5.1929	2.0616	30.8290	2.818E-08	9.4212E-07
Arl4d	-1.1657	5.0655	30.6822	3.0395E-08	1.0054E-06
Nuak1	-1.6373	5.6196	30.6088	3.1566E-08	1.041E-06
Krba1	-1.1333	5.4053	30.5212	3.3023E-08	1.0841E-06
Hdac11	-1.3423	4.1429	30.4982	3.3417E-08	1.0954E-06
Cyp27a1	-1.2144	4.7023	30.3744	3.562E-08	1.1589E-06
Il34	-1.7769	5.5178	30.3509	3.6053E-08	1.1678E-06
Igfbp3	-1.4554	7.4836	30.1904	3.9164E-08	1.2611E-06
Ptpn14	-1.6249	6.9151	29.9476	4.4389E-08	1.4209E-06
Syne2	-1.1858	6.6994	29.8984	4.553E-08	1.451E-06
Gm973	-2.2467	2.3787	29.8645	4.6333E-08	1.4745E-06
Col4a6	-2.0573	4.5500	29.6148	5.27E-08	1.6602E-06
Fn3k	-2.0160	2.1353	29.3901	5.918E-08	1.8449E-06
Klhl33	-3.1400	4.7723	29.2175	6.4691E-08	2.0004E-06
Ago4	-1.3348	4.9066	28.7493	8.2379E-08	2.4874E-06
Tnk2	-1.1134	6.9585	28.7382	8.2855E-08	2.4949E-06
D7Ertd443e	-2.0418	3.3024	28.6894	8.4966E-08	2.555E-06
Fbrsl1	-1.0478	5.3162	28.6454	8.6919E-08	2.6101E-06

Chmp4c	-1.1502	5.4312	28.5738	9.0196E-08	2.6974E-06
Cnst	-1.0961	5.9179	28.5429	9.1647E-08	2.7348E-06
Acot2	-1.2260	4.4511	28.1186	1.141E-07	3.3302E-06
Fry	-1.0932	6.0027	28.0450	1.1853E-07	3.4273E-06
Thap3	-1.1515	4.3545	27.8008	1.3447E-07	3.8474E-06
Nyap1	-1.8930	2.6784	27.7811	1.3585E-07	3.8818E-06
Sh2d4a	-1.3776	4.4879	27.6563	1.449E-07	4.1189E-06
Wdr45	-1.0579	5.5977	27.6261	1.4718E-07	4.1783E-06
Lysmd1	-1.5565	5.0767	27.4417	1.619E-07	4.5605E-06
Ascl2	-2.1046	2.9289	27.0061	2.0281E-07	5.5553E-06
Camkk1	-1.5133	4.8373	26.9115	2.1299E-07	5.7978E-06
Spag11b	-5.7431	-1.0900	26.8384	2.212E-07	5.9987E-06
Fhdc1	-1.6223	6.3031	26.7927	2.2649E-07	6.1271E-06
Gpha2	-2.8984	0.1540	26.5697	2.5419E-07	6.7923E-06
Ppp1r13b	-1.1130	5.4232	26.4146	2.7545E-07	7.2538E-06
Edaradd	-1.5823	3.8487	26.3434	2.8579E-07	7.49E-06
Bco1	-2.3410	1.2693	26.2993	2.9239E-07	7.6354E-06
Dlg3	-1.2029	3.8818	26.2055	3.0695E-07	7.9489E-06
Ufsp1	-1.4580	3.6620	26.2021	3.0749E-07	7.9537E-06
Kazald1	-2.5142	1.8165	26.1512	3.157E-07	8.1275E-06
Dst	-1.2188	9.3465	26.1260	3.1984E-07	8.2146E-06
Rab3a	-1.1550	3.9967	25.9746	3.4594E-07	8.8331E-06
Prodh	-1.2503	5.0261	25.7657	3.8548E-07	9.8083E-06
Ninl	-1.4389	2.5217	25.6764	4.0373E-07	1.0237E-05
Kctd15	-1.3414	6.5693	25.5267	4.363E-07	1.0973E-05
Paqr7	-1.5268	5.1961	25.4781	4.4744E-07	1.1211E-05
Slc25a42	-1.0503	5.3053	25.4147	4.6239E-07	1.155E-05
Ccna1	-4.3141	-0.9103	25.3875	4.6894E-07	1.1687E-05
Fam189a2	-1.1385	5.4382	25.3256	4.8424E-07	1.2027E-05
Ccl27a	-1.9070	5.5973	25.3015	4.9034E-07	1.2164E-05
Rnf43	-1.2881	3.8109	25.2632	5.0015E-07	1.2366E-05
Nipal3	-1.2978	4.6060	25.2535	5.0268E-07	1.2414E-05
Lgr6	-1.6015	5.1025	25.1795	5.2235E-07	1.2842E-05
Kcnu1	-1.9910	1.1985	25.1655	5.2616E-07	1.2921E-05
Col23a1	-1.1339	5.4588	25.1047	5.4299E-07	1.3276E-05
Mtss1l	-1.2873	5.6921	25.1045	5.4306E-07	1.3276E-05
Lnx1	-1.5115	4.3534	25.1023	5.4368E-07	1.3276E-05
Rgmb	-1.3472	5.8936	25.0561	5.5687E-07	1.3553E-05
Ccdc3	-1.4608	5.2566	25.0222	5.6674E-07	1.3763E-05
Mycl	-1.7954	5.2494	24.9929	5.7541E-07	1.3958E-05
Gmpr	-2.2331	6.1187	24.9407	5.912E-07	1.4293E-05
Ahnak2	-1.9687	8.0596	24.9334	5.9345E-07	1.4332E-05
Spns2	-1.3642	7.4218	24.9037	6.0266E-07	1.4513E-05
2210011C24Rik	-1.9206	0.7187	24.8504	6.1956E-07	1.488E-05
Thra	-1.0414	6.3972	24.7754	6.4414E-07	1.5398E-05
Hlf	-1.9005	5.3968	24.7486	6.5317E-07	1.555E-05
Pyy	-3.7276	1.1493	24.7427	6.5518E-07	1.5581E-05
Rab11fip4	-2.0616	6.4407	24.6071	7.0294E-07	1.6554E-05
Cbs	-2.5585	4.0228	24.5186	7.3595E-07	1.722E-05
Prob1	-1.1970	5.9763	24.4847	7.4904E-07	1.7482E-05
Ptpn21	-1.0224	4.8193	24.4834	7.4952E-07	1.7482E-05
Sh3rf2	-1.6345	5.9161	24.4600	7.587E-07	1.7654E-05
Nfix	-1.0899	7.9820	24.4295	7.7081E-07	1.7883E-05
Zfp949	-1.2460	6.6625	24.3755	7.927E-07	1.8371E-05
Ramp1	-1.1925	5.0113	24.3282	8.1243E-07	1.8788E-05
Zfp219	-1.0970	5.8950	24.2996	8.2454E-07	1.9048E-05
Gpr37	-1.7108	1.8435	24.2728	8.3612E-07	1.9243E-05
H2afv	-1.0297	5.2145	24.2417	8.4974E-07	1.9527E-05
Pdzd7	-2.1980	0.8360	24.2255	8.5688E-07	1.965E-05
Nhsl1	-1.3749	6.3363	24.1318	8.9962E-07	2.0522E-05
Bcl2l14	-2.0740	1.7385	24.1200	9.0517E-07	2.0627E-05
Slc16a9	-1.3642	3.5614	23.9356	9.9611E-07	2.2419E-05
Cdc42bpg	-1.3123	7.2797	23.9231	1.0026E-06	2.2542E-05

Hdac5	-1.1500	6.5968	23.9104	1.0092E-06	2.2667E-05
Adamts14	-1.6730	5.3089	23.6658	1.146E-06	2.5582E-05
Slc38a3	-1.6930	4.1772	23.5873	1.1937E-06	2.6414E-05
Kif21a	-1.5561	7.3282	23.4688	1.2695E-06	2.7939E-05
Micu1	-1.0764	6.8387	23.3049	1.3825E-06	3.0241E-05
Otop2	-3.0994	0.0264	23.2404	1.4297E-06	3.1211E-05
Necab3	-2.2456	0.1222	23.1994	1.4605E-06	3.1851E-05
BC024139	-2.1546	0.5332	23.1123	1.5281E-06	3.3063E-05
Cyp39a1	-1.2727	3.7018	23.0949	1.542E-06	3.3317E-05
Hpgds	-1.2088	6.2220	23.0918	1.5445E-06	3.3317E-05
Olfr1505	-2.5423	1.6106	23.0505	1.578E-06	3.3943E-05
Klhdc8b	-1.3114	3.4462	23.0503	1.5781E-06	3.3943E-05
Rtn4r	-1.8535	3.2149	22.9688	1.6465E-06	3.5275E-05
Frem2	-1.7465	2.7555	22.9576	1.6561E-06	3.5446E-05
Abtb1	-1.1804	4.6793	22.9235	1.6858E-06	3.6009E-05
Foxn3	-1.1915	6.9572	22.8860	1.719E-06	3.6647E-05
Efna3	-1.5720	6.6975	22.8040	1.7939E-06	3.8133E-05
Ldb1	-1.0171	7.2590	22.7892	1.8078E-06	3.839E-05
Cib2	-1.0760	5.8823	22.7680	1.8278E-06	3.8699E-05
Gata3	-1.8548	7.5001	22.7664	1.8294E-06	3.8699E-05
Vipr1	-2.0750	3.8335	22.7572	1.8382E-06	3.8847E-05
Epha5	-1.7086	1.5490	22.7309	1.8635E-06	3.9305E-05
Efs	-1.3395	5.9507	22.5998	1.995E-06	4.1638E-05
Trpv6	-1.5823	3.0832	22.3816	2.235E-06	4.5901E-05
Tbc1d16	-1.0672	5.0004	22.1053	2.581E-06	5.2269E-05
Fbp2	-1.3923	6.2618	22.0670	2.6329E-06	5.3222E-05
Slc37a4	-1.2306	4.7565	22.0645	2.6364E-06	5.3234E-05
Ache	-1.6623	4.8381	22.0630	2.6384E-06	5.3234E-05
Wnk2	-1.6062	6.8428	22.0228	2.6943E-06	5.4312E-05
Rspo3	-2.4426	1.4507	21.9534	2.7936E-06	5.5951E-05
Tnnt2	-1.6646	3.0767	21.9510	2.797E-06	5.5969E-05
Chst3	-1.3945	1.9736	21.9454	2.8051E-06	5.6028E-05
Itpkb	-1.1473	7.3234	21.8086	3.0125E-06	5.995E-05
Gpr155	-1.0313	4.1588	21.7471	3.1106E-06	6.1545E-05
Ccnd2	-1.1290	7.1503	21.7329	3.1338E-06	6.178E-05
Phyhip	-1.4711	6.3571	21.6513	3.2699E-06	6.4194E-05
Cachd1	-1.0475	4.8631	21.6345	3.2987E-06	6.4592E-05
Fam83f	-1.4044	5.5652	21.5743	3.4037E-06	6.6464E-05
Rdm1	-1.1310	3.6182	21.5562	3.4361E-06	6.6977E-05
Lmbr1l	-1.0446	5.3687	21.4586	3.6155E-06	7.016E-05
Cyp2j9	-1.3897	3.0471	21.4190	3.691E-06	7.1497E-05
Exph5	-1.5693	4.1705	21.3783	3.7702E-06	7.2903E-05
Pkib	-1.7278	4.6279	21.3453	3.8356E-06	7.397E-05
Rec8	-2.0710	0.8741	21.3425	3.8412E-06	7.4014E-05
Spry1	-1.3595	5.8479	21.3211	3.8844E-06	7.4779E-05
Pou4f3	-5.1940	-1.4193	21.3166	3.8935E-06	7.4889E-05
Car14	-1.1003	3.4280	21.3003	3.9267E-06	7.5394E-05
Vsig10l	-1.2810	5.7368	21.2849	3.9584E-06	7.5804E-05
Tmem35a	-1.7807	2.3656	21.2364	4.0599E-06	7.7475E-05
Crlf1	-1.5477	4.5085	21.1809	4.179E-06	7.9401E-05
Mcf2l	-1.0137	5.0746	21.1595	4.226E-06	8.0154E-05
Ccdc27	-2.2018	1.0290	21.1548	4.2364E-06	8.028E-05
Cttnbp2	-2.0470	4.6949	21.1193	4.3157E-06	8.1291E-05
Trat1	-1.7253	0.8580	21.0754	4.4155E-06	8.3028E-05
Rasgrp1	-1.4026	4.4989	21.0457	4.4846E-06	8.411E-05
Mblac2	-1.0435	3.4793	20.9571	4.6969E-06	8.779E-05
Plekhn1	-1.4350	7.3026	20.9404	4.738E-06	8.8405E-05
Ucn2	-1.7121	2.9618	20.9276	4.7697E-06	8.8921E-05
Plch2	-1.0556	6.3887	20.8436	4.9835E-06	9.274E-05
Il1f10	-2.4843	3.4261	20.8356	5.0044E-06	9.298E-05
Myh14	-1.2082	6.9290	20.7910	5.1224E-06	9.4608E-05
Cited4	-1.7478	5.4582	20.7889	5.1279E-06	9.463E-05
Tmc3	-1.4027	2.0466	20.7870	5.1331E-06	9.4647E-05

Nkg7	-1.8599	2.1150	20.7072	5.3515E-06	9.8175E-05
Rtn2	-1.5113	8.0498	20.7044	5.3594E-06	9.8237E-05
Cacna1e	-1.6133	3.2803	20.7024	5.365E-06	9.8257E-05
C2cd2	-1.0571	7.4418	20.5847	5.705E-06	0.00010405
Bpgm	-1.0817	4.9998	20.5339	5.8585E-06	0.00010649
Tfdp2	-1.1091	5.1528	20.5160	5.9136E-06	0.00010731
Pramef12	-2.2754	1.0913	20.5033	5.9528E-06	0.00010785
Cd82	-1.2752	7.1826	20.4551	6.1047E-06	0.00011042
Mlycd	-1.1270	5.9277	20.3861	6.3287E-06	0.0001139
Cdkn2b	-1.3812	3.5400	20.3427	6.4739E-06	0.00011632
Kcna2	-1.4581	2.4313	20.3354	6.4986E-06	0.00011667
Arhgef19	-1.3823	4.0026	20.2567	6.7715E-06	0.00012107
Tmem159	-1.0288	4.6142	20.2136	6.9258E-06	0.00012353
Foxo4	-1.0323	6.4046	20.1999	6.9758E-06	0.00012414
Jag2	-1.0356	7.4158	20.1967	6.9872E-06	0.00012418
Chat	-1.5595	0.8900	20.1957	6.991E-06	0.00012418
Chac1	-1.5233	5.4167	20.1317	7.2287E-06	0.00012778
Tiam1	-1.0852	7.4882	20.1223	7.2645E-06	0.00012831
Gpr165	-5.2500	-1.3855	20.0569	7.5171E-06	0.00013192
Zfp827	-1.3497	2.7814	20.0397	7.5851E-06	0.00013279
Sowaha	-2.4030	-0.3269	19.9778	7.8347E-06	0.00013661
Gm2115	-2.7792	0.8509	19.9396	7.9929E-06	0.00013926
Clca3a1	-1.4591	4.6773	19.9295	8.035E-06	0.00013977
Trp53bp2	-1.0549	5.7590	19.8892	8.2061E-06	0.00014263
Pamr1	-1.2138	4.2458	19.8673	8.3009E-06	0.00014417
Foxo6	-1.7743	1.7205	19.8639	8.3157E-06	0.00014431
Krt36	-2.5229	1.6684	19.8195	8.5109E-06	0.00014737
Trim66	-2.2041	1.2518	19.6547	9.2775E-06	0.00015937
Nectin3	-1.4869	5.4677	19.6464	9.3178E-06	0.00015992
Pard6b	-1.1002	3.7617	19.6449	9.3253E-06	0.00015992
Itgb1bp2	-1.3562	5.6909	19.6421	9.3387E-06	0.00016003
Syne1	-1.0413	6.0139	19.5570	9.7644E-06	0.0001663
Nipsnap1	-1.0324	2.8392	19.5567	9.766E-06	0.0001663
Dzip1	-1.3603	4.1938	19.4784	1.0174E-05	0.00017245
Dnaaf1	-1.3871	2.3610	19.4525	1.0313E-05	0.00017426
Col7a1	-1.5011	6.7365	19.4488	1.0333E-05	0.00017447
Wdr47	-1.3335	5.4739	19.4427	1.0366E-05	0.00017489
2510002D24Rik	-1.0676	3.8465	19.3890	1.0662E-05	0.00017932
Ccdc28a	-1.1185	3.5181	19.3256	1.1022E-05	0.0001848
Baiap2l2	-1.8185	1.5312	19.2583	1.1417E-05	0.0001902
Tmem38a	-1.3719	8.4925	19.2220	1.1637E-05	0.00019333
Ephb6	-1.5671	7.1925	19.1858	1.1859E-05	0.00019644
Foxq1	-1.6043	3.0209	19.1786	1.1904E-05	0.00019688
Arvcf	-1.1595	4.9329	19.1675	1.1973E-05	0.00019775
Mpz	-1.1918	5.5145	19.1631	1.2001E-05	0.00019803
Abca4	-1.6839	0.9064	19.0836	1.2512E-05	0.00020614
Dsg2	-1.3174	5.0080	18.9792	1.3215E-05	0.00021627
Mreg	-1.1002	6.1666	18.9655	1.3311E-05	0.00021702
Ccl24	-1.5742	2.8654	18.9356	1.352E-05	0.00021978
Abcc5	-1.2179	4.8733	18.7954	1.4552E-05	0.0002348
Atp2b4	-1.2719	7.5535	18.7853	1.4629E-05	0.0002357
6430573F11Rik	-1.5594	1.2224	18.7685	1.4758E-05	0.00023708
Asb2	-1.2403	6.4143	18.7371	1.5003E-05	0.00024013
Nt5c3	-1.1049	6.7816	18.6555	1.5659E-05	0.0002488
St8sia1	-1.6347	2.0252	18.6494	1.571E-05	0.00024943
3425401B19Rik	-1.8153	7.6688	18.6421	1.577E-05	0.0002502
Vamp5	-1.2503	5.1239	18.5909	1.6199E-05	0.0002559
Lhx2	-1.0001	3.1020	18.5734	1.6349E-05	0.00025788
Sult5a1	-1.6202	4.3445	18.5651	1.642E-05	0.00025882
Tcap	-1.9024	10.1511	18.5443	1.66E-05	0.00026109
Arl4a	-1.0682	6.4139	18.5414	1.6625E-05	0.0002613
Mcc	-1.2646	4.9343	18.5256	1.6763E-05	0.00026301
Gm10638	-1.7598	0.6562	18.5023	1.697E-05	0.00026538

Stk10	-1.0942	5.5598	18.5009	1.6982E-05	0.00026538
Hoxa5	-1.3063	4.0972	18.4944	1.704E-05	0.00026591
Egfl6	-1.1772	2.0878	18.4689	1.727E-05	0.00026911
Gstm1	-1.0786	7.1242	18.3406	1.8473E-05	0.00028622
Ccdc24	-1.4226	4.6731	18.3201	1.8673E-05	0.0002887
P2rx2	-2.4992	3.7927	18.2699	1.9171E-05	0.00029577
Col20a1	-1.0207	3.5096	18.2473	1.94E-05	0.00029867
Krt12	-2.2697	0.4516	18.2322	1.9554E-05	0.00030063
Gan	-1.6551	7.7422	18.2236	1.9643E-05	0.00030156
Capn13	-5.1251	-1.4593	18.2218	1.9661E-05	0.00030163
Ano7	-1.8695	5.2501	18.2185	1.9696E-05	0.0003018
Rab3b	-2.4334	0.3534	18.2127	1.9756E-05	0.00030223
1700028P14Rik	-3.0849	-0.3473	18.1659	2.0248E-05	0.00030825
Cbx4	-1.0114	5.3366	18.0969	2.0994E-05	0.00031828
Rbl2	-1.0679	5.9211	18.0847	2.1129E-05	0.0003201
Lrig3	-1.0193	4.8916	18.0234	2.1821E-05	0.00032944
Dbp	-1.1636	5.7202	17.9436	2.2755E-05	0.00034189
2010107G23Rik	-1.2122	4.1327	17.9324	2.2889E-05	0.00034295
Arhgef28	-1.1674	4.5844	17.9036	2.3238E-05	0.0003477
Hoxa3	-1.0577	2.7432	17.8961	2.333E-05	0.00034884
Tgm7	-2.4056	2.3501	17.8467	2.3943E-05	0.0003568
Map7	-1.0120	5.6985	17.8157	2.4336E-05	0.00036142
Lin7b	-2.4998	0.9481	17.7686	2.4946E-05	0.00036947
Magix	-1.8074	2.3478	17.6488	2.6568E-05	0.00039124
Npy1r	-1.3816	3.5132	17.6389	2.6707E-05	0.00039236
Sh3d21	-1.6092	5.2555	17.5815	2.7526E-05	0.00040357
Rnf39	-1.3884	6.1411	17.5627	2.7799E-05	0.00040703
Plb1	-1.8032	3.3518	17.5345	2.8214E-05	0.000412
Marveld3	-1.2005	3.1395	17.4601	2.934E-05	0.00042649
Coro6	-1.3156	6.6588	17.3777	3.0641E-05	0.00044182
Camsap3	-1.2644	6.0679	17.3553	3.1003E-05	0.00044675
Dgcr6	-1.2646	5.1057	17.3004	3.1912E-05	0.00045803
Slc2a12	-1.6015	4.8649	17.2978	3.1955E-05	0.00045835
Ppp1r1a	-1.2585	4.6879	17.2690	3.2443E-05	0.00046413
Bicdl2	-1.7098	5.4915	17.1940	3.375E-05	0.00048219
Wipf3	-1.0654	4.6912	17.1658	3.4255E-05	0.00048877
Cyp4f37	-2.3544	2.2564	17.1580	3.4397E-05	0.00048978
Phldb3	-1.5931	5.5137	17.1379	3.4761E-05	0.00049374
Rhbg	-1.5522	4.8423	17.1001	3.5461E-05	0.00050237
Adgra3	-1.5479	6.7585	17.0866	3.5714E-05	0.00050563
Il6ra	-1.0153	7.1573	17.0176	3.7035E-05	0.00052296
H2-M5	-1.8752	1.0214	17.0019	3.7343E-05	0.00052629
Itpr2	-1.0520	6.0667	16.9791	3.7794E-05	0.00053231
Ccdc36	-1.9310	0.0985	16.9681	3.8013E-05	0.00053505
Trim2	-1.5537	6.3338	16.9516	3.8346E-05	0.00053938
Mn1	-1.2642	5.4300	16.9451	3.8476E-05	0.00054087
Itpr3	-1.3627	8.4278	16.8930	3.9548E-05	0.00055415
Pik3r2	-1.0545	6.4206	16.8359	4.0755E-05	0.00056815
Mss51	-2.0610	3.2917	16.8021	4.1488E-05	0.0005769
Cgn	-1.6889	6.6991	16.8004	4.1524E-05	0.00057702
Tnfaip8l3	-1.7336	3.2943	16.7736	4.2115E-05	0.00058486
Gal3st1	-2.2313	4.4362	16.7390	4.289E-05	0.0005945
Cfap100	-2.0970	0.1084	16.7282	4.3135E-05	0.00059709
Rassf7	-1.3213	5.6755	16.6951	4.3894E-05	0.00060649
Plekha5	-1.0650	5.3108	16.6609	4.4694E-05	0.00061597
Rarg	-1.0918	7.4831	16.6456	4.5055E-05	0.00061861
Klhdc1	-1.2236	4.0472	16.5979	4.6202E-05	0.00063317
Fam131a	-1.1323	3.2748	16.5901	4.6392E-05	0.00063497
Slc8a3	-1.4892	5.1019	16.5878	4.6449E-05	0.00063535
Gm3045	-2.2910	0.1535	16.5325	4.7823E-05	0.00065334
Trdn	-1.5476	8.0081	16.5062	4.849E-05	0.00066162
Col8a2	-1.1438	2.4701	16.5012	4.8619E-05	0.00066296
Peli3	-1.0842	2.7361	16.4989	4.868E-05	0.00066337

Ube2d1	-1.1542	6.0287	16.4820	4.9115E-05	0.00066764
Cobl	-1.5229	7.2655	16.4557	4.98E-05	0.00067569
Scn8a	-1.6054	1.1686	16.4377	5.0275E-05	0.00068045
Wdyhv1	-1.0423	4.1380	16.4206	5.0731E-05	0.00068577
Lrrc46	-3.5594	-0.8795	16.3988	5.1317E-05	0.00069283
Rapgefl1	-1.4281	7.0399	16.3856	5.1677E-05	0.00069683
Ddr1	-1.0766	7.3805	16.3690	5.213E-05	0.0007025
Pdk2	-1.2113	7.8713	16.3578	5.2439E-05	0.00070584
Comp	-1.5905	0.7900	16.3455	5.2781E-05	0.00070909
Kcng4	-2.1244	0.8342	16.3151	5.3635E-05	0.00071923
Themis	-2.9156	0.2431	16.3058	5.3899E-05	0.00072189
Arhgef26	-1.2851	3.8892	16.2859	5.4467E-05	0.00072816
Lrp2bp	-2.3990	2.0057	16.2836	5.4535E-05	0.00072861
Sgca	-1.2099	6.3690	16.2583	5.5268E-05	0.0007366
2310007B03Rik	-1.5973	4.2391	16.2452	5.5651E-05	0.00074045
Myoz1	-1.4556	9.1401	16.2247	5.6256E-05	0.00074795
Tnfrsf25	-1.3307	2.5585	16.2210	5.6367E-05	0.00074896
Gm5127	-1.6134	1.4655	16.1563	5.8324E-05	0.00077234
Gjc2	-1.9303	1.3942	16.1558	5.8339E-05	0.00077234
Prf1	-1.7947	2.7266	16.1512	5.8483E-05	0.00077378
Krt27	-5.1182	-1.4609	16.1446	5.8686E-05	0.000776
Srgap2	-1.0456	6.7060	16.1270	5.9232E-05	0.00078228
Ciart	-1.1453	3.5948	16.0548	6.1535E-05	0.00080892
Slc23a1	-1.6291	1.1535	16.0405	6.2002E-05	0.00081246
A930018M24Rik	-2.7518	3.4870	16.0123	6.2932E-05	0.00082316
Lpar3	-1.4111	2.7279	15.9992	6.3371E-05	0.00082841
Zfp775	-1.1057	3.2858	15.9969	6.3447E-05	0.00082891
Ralgps2	-1.1255	5.2082	15.9723	6.4278E-05	0.00083926
Cd247	-1.0142	3.2767	15.9609	6.4665E-05	0.00084332
Fzd10	-1.0869	5.1364	15.9448	6.5217E-05	0.00084949
Igsf9	-1.2488	4.9146	15.9427	6.5288E-05	0.00084991
Zc3h6	-1.0228	3.1271	15.9374	6.5471E-05	0.00085179
Ifnlr1	-1.1573	4.6120	15.9227	6.5984E-05	0.00085693
Vangl2	-1.1849	5.0969	15.8832	6.7376E-05	0.00087241
Slc6a4	-1.0589	5.0221	15.8668	6.7962E-05	0.00087792
Ell3	-2.0861	3.3989	15.8441	6.878E-05	0.00088691
8430408G22Rik	-1.4548	4.3199	15.8405	6.8912E-05	0.00088809
Ly6g6e	-1.6230	5.4580	15.8342	6.9141E-05	0.00088999
Frs3	-1.2173	1.9255	15.8290	6.9331E-05	0.00089191
Hs3st6	-2.0925	3.8885	15.8244	6.9501E-05	0.00089304
Ikzf2	-1.4338	4.7695	15.7752	7.133E-05	0.00091385
Dhrs7c	-1.2798	5.5103	15.7625	7.1812E-05	0.00091835
Art4	-1.3701	3.7686	15.7300	7.3055E-05	0.00093157
Psors1c2	-1.8512	5.2614	15.7035	7.4087E-05	0.00094253
Cd3e	-1.7493	2.9271	15.6990	7.4263E-05	0.00094423
Gm14137	-1.6358	5.1326	15.6521	7.6129E-05	0.0009657
Pik3c2g	-1.7324	1.7612	15.5880	7.8755E-05	0.00099554
Lrp4	-1.6615	7.0320	15.5662	7.9665E-05	0.00100646
Kcnh2	-1.6219	1.7243	15.5615	7.9865E-05	0.0010084
Celsr1	-1.2030	6.6119	15.5333	8.1064E-05	0.0010209
Ano8	-1.1763	5.7232	15.5105	8.205E-05	0.00103182
Slitrk6	-1.2264	4.7515	15.4601	8.4268E-05	0.00105364
Pygo1	-1.1832	3.7000	15.4571	8.4398E-05	0.00105467
Anxa9	-1.6019	6.6750	15.4287	8.5677E-05	0.00106759
Wnt2	-2.0664	2.1657	15.3948	8.7228E-05	0.00108259
Tuft1	-1.4592	6.7991	15.3771	8.8049E-05	0.00109216
BC107364	-2.1654	0.1280	15.3747	8.8161E-05	0.00109293
Mylk2	-1.4861	9.4903	15.3693	8.8414E-05	0.00109544
Upk3b	-3.3071	1.6235	15.3530	8.9179E-05	0.00109886
Bbc3	-1.3484	2.3334	15.3516	8.9244E-05	0.00109886
Gipc2	-1.6305	0.9426	15.3431	8.9647E-05	0.00110258
Neu3	-1.3605	4.1129	15.3060	9.1426E-05	0.00111867
Gpsm1	-1.2736	5.7518	15.2690	9.3235E-05	0.00113774

Tjp3	-1.6258	5.9454	15.2649	9.3439E-05	0.00113959
Rassf10	-1.4254	3.2322	15.2607	9.3647E-05	0.00114149
Agr3	-3.2332	-1.0899	15.2587	9.3745E-05	0.00114204
Micalcl	-1.7400	3.1906	15.2515	9.4101E-05	0.00114467
Fut1	-1.4329	4.1842	15.2023	9.6588E-05	0.0011721
Tanc1	-1.2040	6.8416	15.1959	9.6911E-05	0.00117472
Dhcr7	-1.0824	6.8129	15.1332	0.00010019	0.00120773
Smco1	-1.5321	3.5423	15.0835	0.00010286	0.00123598
Dnajc28	-1.4080	1.6517	15.0700	0.0001036	0.001244
Kcnj16	-2.6331	2.9649	15.0527	0.00010455	0.0012541
Pmp2	-2.2704	-0.0552	15.0441	0.00010503	0.00125913
Krt15	-2.0667	9.5482	14.9648	0.00010954	0.00130385
Fam117a	-1.1645	4.4983	14.9281	0.00011169	0.001328
Slc25a34	-1.4564	4.3665	14.8233	0.00011807	0.00139223
Cbx2	-1.0683	4.7838	14.8205	0.00011824	0.00139304
Ptpn13	-1.0280	6.9989	14.8177	0.00011842	0.00139442
Inmt	-2.9022	4.7346	14.7606	0.00012206	0.00143266
Omp	-2.6912	-0.4306	14.7034	0.00012582	0.00147039
Ypel2	-1.2390	5.2529	14.6777	0.00012755	0.00148587
Traf5	-1.0978	2.5879	14.6744	0.00012777	0.00148764
Kcng1	-2.2154	0.0942	14.6724	0.00012791	0.00148776
C1qtnf4	-1.0355	1.9146	14.6722	0.00012792	0.00148776
Atg9b	-1.5790	6.3295	14.6613	0.00012866	0.00149561
Kel	-5.0950	-1.4743	14.6137	0.00013195	0.00152899
Ak1	-1.2275	8.0139	14.6067	0.00013244	0.00153388
Unc79	-2.0096	0.2682	14.5222	0.00013852	0.0015966
Blnk	-1.0430	4.8295	14.5147	0.00013907	0.00160126
Prss8	-1.2155	5.5216	14.4812	0.00014157	0.00162663
Rhbdl3	-1.0619	3.9537	14.4638	0.00014288	0.00163999
Jph1	-1.3654	6.0734	14.4607	0.00014312	0.00164181
Spink1	-3.0521	-0.1726	14.4548	0.00014356	0.00164523
Smarcd3	-1.2155	5.1534	14.4141	0.0001467	0.00167323
Wee1	-1.0549	4.9900	14.4118	0.00014688	0.00167363
Hsbp1l1	-1.3377	2.7091	14.4090	0.0001471	0.00167431
Lsr	-1.2777	6.8247	14.3890	0.00014867	0.00169132
Mob3b	-1.0732	3.8807	14.3638	0.00015067	0.00170962
Ctnnd2	-1.1482	2.9166	14.3445	0.00015223	0.00172546
Casq1	-1.2492	8.8169	14.3330	0.00015316	0.00173332
Snai3	-1.3636	5.4548	14.2649	0.0001588	0.00178788
Klhl31	-1.4465	6.6334	14.2626	0.00015899	0.00178915
Odf3b	-2.0356	-0.4431	14.2512	0.00015996	0.00179911
N4bp3	-1.0996	4.7496	14.2394	0.00016097	0.00180766
Slc7a2	-1.4620	5.8363	14.1988	0.00016448	0.00184234
Rhbdl1	-1.2589	2.7644	14.1947	0.00016483	0.00184539
Cuedc1	-1.0545	5.5049	14.1797	0.00016615	0.00185728
Arhgef5	-1.1547	6.9183	14.1569	0.00016818	0.00187296
Tcea3	-1.4117	5.8057	14.1562	0.00016824	0.00187296
Pdzk1ip1	-1.4586	6.7892	14.1478	0.00016899	0.00188039
Ntf3	-1.2226	2.0006	14.0964	0.00017368	0.00192658
Ccdc120	-1.0874	5.9101	14.0943	0.00017387	0.00192779
Best2	-2.0928	1.8886	14.0645	0.00017665	0.00195476
Cr2	-2.5557	-0.5343	14.0589	0.00017717	0.00195843
Myom1	-1.2348	9.3337	14.0497	0.00017804	0.00196499
Krt80	-1.4569	8.6468	14.0445	0.00017853	0.00196947
Pfkm	-1.2095	10.4524	14.0394	0.00017902	0.00197388
Gm20219	-1.1620	5.5080	14.0191	0.00018096	0.00199224
Amdhd1	-1.7513	0.8701	14.0072	0.00018211	0.00200283
Col17a1	-1.1064	10.0499	13.9773	0.00018503	0.00202881
Liph	-1.2530	4.4701	13.9738	0.00018538	0.00203159
Cmya5	-1.3062	9.8448	13.9420	0.00018854	0.00206105
Dpp6	-2.1410	-0.5047	13.9016	0.00019264	0.00210166
Atp1b2	-1.5640	6.4270	13.8950	0.00019332	0.00210592
Pdlim2	-1.2399	6.6597	13.8824	0.00019461	0.00211896

Actbl2	-4.7570	-1.6415	13.8624	0.0001967	0.00213745
Krt32	-2.7648	-0.3445	13.8487	0.00019813	0.00214984
Slc6a2	-1.6438	4.3830	13.8470	0.00019831	0.0021507
Sept5	-1.0966	7.3630	13.7840	0.00020507	0.00221302
Sema6c	-1.6843	4.6472	13.7404	0.00020989	0.00226055
Fut2	-1.1530	5.5493	13.7345	0.00021056	0.00226324
9430007A20Rik	-2.5776	-0.9037	13.7030	0.00021411	0.00229133
Tmie	-1.3504	1.8205	13.6946	0.00021507	0.00230045
Rpl3l	-1.1376	7.2902	13.6725	0.00021762	0.00232314
Omd	-1.7931	1.9789	13.6377	0.00022169	0.00235619
Foxn1	-1.0134	3.8521	13.6219	0.00022357	0.00237155
Olfr851	-2.0549	-0.5196	13.6048	0.00022561	0.00238974
Capn3	-1.1577	5.8302	13.5969	0.00022656	0.00239702
Dlg2	-1.4746	4.5853	13.5718	0.00022961	0.00242041
G6b	-1.5833	2.0370	13.5401	0.00023352	0.0024545
Nectin4	-1.1208	7.3980	13.5331	0.00023439	0.00246013
Fndc5	-1.0413	3.9762	13.5232	0.00023564	0.00247198
Frzb	-1.6767	2.8927	13.5163	0.0002365	0.0024798
Marveld2	-1.0826	3.9260	13.4809	0.000241	0.00251734
Il20rb	-1.4638	6.6638	13.4531	0.0002446	0.00254763
Lmod3	-1.3208	6.2669	13.4510	0.00024488	0.00254813
Sh3d19	-1.0796	7.1907	13.4450	0.00024566	0.00255377
Baiap2l1	-1.1662	7.9232	13.4295	0.0002477	0.0025701
Bpifc	-1.3457	7.2659	13.4142	0.00024973	0.00258624
9330159F19Rik	-1.1740	4.1763	13.3937	0.00025247	0.00260848
Slc4a10	-3.7525	-0.7498	13.3649	0.00025638	0.0026451
Synm	-1.2503	8.3323	13.3224	0.00026225	0.00269874
Ampd1	-1.8578	6.7826	13.2915	0.00026661	0.00273389
Pou2f3	-1.6542	6.6828	13.2797	0.00026829	0.00274719
Gem	-1.0797	4.2571	13.2480	0.00027288	0.00278894
Slc9a3	-1.6718	2.7303	13.1866	0.00028196	0.00287635
Asb16	-1.2583	5.4848	13.1450	0.00028829	0.00293683
Ceacam20	-2.8889	-1.2303	13.1241	0.00029152	0.00296148
Rnf208	-1.2359	5.1253	13.1063	0.00029431	0.00298565
Capns2	-1.4929	6.3592	13.1041	0.00029464	0.00298625
Wnt6	-1.7831	2.1222	13.1023	0.00029493	0.0029878
Ust	-1.0021	4.3281	13.1010	0.00029514	0.00298846
Rasal1	-1.1476	3.2809	13.0981	0.00029559	0.0029917
Slc6a9	-1.0844	4.5015	13.0880	0.00029719	0.00300366
Gdpd3	-1.1591	4.5108	13.0776	0.00029884	0.0030162
Sgcg	-1.3597	5.5589	13.0478	0.00030363	0.00305608
Dsg1c	-1.9934	2.6568	13.0249	0.00030738	0.00308807
Fbxo32	-1.4825	8.4386	13.0089	0.00031001	0.0031088
Rnf222	-1.0577	3.8581	12.9599	0.00031824	0.00318397
Myl4	-1.6763	2.2590	12.9076	0.00032726	0.00326673
Skint8	-1.8993	0.9909	12.9012	0.00032838	0.0032764
Klf15	-1.1416	5.1934	12.8902	0.00033031	0.00328974
Pak3	-1.3694	4.5663	12.8794	0.00033222	0.00330272
Cdk15	-1.7524	-0.1155	12.8595	0.00033577	0.00333193
Fitm1	-1.1108	5.5989	12.8495	0.00033756	0.00334516
Hsd17b14	-2.1119	2.6405	12.8467	0.00033808	0.00334721
5730596B20Rik	-1.5306	0.2504	12.8412	0.00033907	0.00335551
Fam89a	-1.4679	5.1861	12.8132	0.00034418	0.00339529
Lmod1	-2.0897	6.1699	12.8075	0.00034523	0.00340415
Chd3os	-1.7696	6.5248	12.7989	0.00034682	0.00341824
Fam189b	-1.0675	3.4253	12.7972	0.00034713	0.00341978
Gna15	-1.0213	4.5239	12.7888	0.0003487	0.00343218
Flywch2	-1.5317	0.0725	12.7852	0.00034938	0.00343649
Dcaf6	-1.0319	6.3537	12.7847	0.00034946	0.00343649
Opcml	-1.4573	3.0502	12.7737	0.00035153	0.00345216
Abca5	-1.2984	6.2307	12.7575	0.00035458	0.00347593
Lpin3	-1.1021	4.4211	12.7470	0.00035657	0.00348762
Nrtn	-1.6492	4.9259	12.7412	0.00035768	0.0034969

F2rl1	-1.1093	5.1824	12.7389	0.00035812	0.00349964
Acsbg1	-1.4639	7.8408	12.7240	0.00036099	0.0035261
Rorb	-2.6646	-0.6258	12.7179	0.00036217	0.00353603
Ebf4	-5.2014	-1.4304	12.6993	0.00036579	0.00356499
Bmp3	-1.0562	4.0623	12.6957	0.0003665	0.00356754
Bmp8b	-4.5058	-1.7569	12.6842	0.00036876	0.00358436
Lypd2	-2.8041	-0.0361	12.6666	0.00037224	0.00360593
Sox21	-1.3243	4.7732	12.6604	0.00037349	0.00361261
Wfdc5	-1.0314	5.6014	12.6332	0.00037896	0.00366231
Ano9	-1.5692	7.3968	12.6133	0.00038301	0.00369649
Ppm1l	-1.0481	5.4183	12.5680	0.0003924	0.00377048
Mesp2	-1.6315	1.7228	12.5644	0.00039316	0.00377446
Tnfrsf19	-1.6390	6.1038	12.5496	0.00039629	0.00379943
Ccdc62	-1.2096	1.3969	12.5445	0.00039736	0.0038081
Tcp11	-2.0828	-0.2828	12.4896	0.00040923	0.00390642
Pramel7	-3.7281	-1.2668	12.4342	0.00042155	0.00400649
Lrrc36	-3.1886	-1.1164	12.4266	0.00042325	0.00402092
Gabrp	-2.0324	3.2368	12.4244	0.00042377	0.00402406
Papln	-1.0545	3.4451	12.4174	0.00042535	0.00403163
Ppfia3	-1.1634	5.1424	12.4151	0.00042587	0.00403344
A530084C06Rik	-1.2262	1.8735	12.4003	0.00042926	0.00405327
Gm2244	-5.1734	-1.4478	12.3887	0.00043194	0.00407332
Mdga1	-1.1398	1.8955	12.3844	0.00043293	0.00408091
Osr2	-1.2523	3.4161	12.3806	0.00043382	0.00408656
Col4a5	-1.1320	4.2049	12.3802	0.00043391	0.00408656
Pygm	-1.2701	11.2935	12.3778	0.00043448	0.00409017
9230102O04Rik	-1.5093	0.7712	12.3757	0.00043496	0.00409292
Xkrx	-1.4800	5.7906	12.3682	0.00043671	0.00410763
Fam110a	-1.3720	6.0745	12.3200	0.00044814	0.00420246
Cyp3a13	-1.2810	2.9701	12.3121	0.00045004	0.00421485
Sp6	-1.4469	6.0007	12.2720	0.00045981	0.00428089
Cyp2j6	-1.1710	5.3107	12.2706	0.00046015	0.00428089
Smpd3	-1.2012	6.4668	12.2516	0.00046486	0.00431848
Epha1	-1.1107	6.5108	12.2291	0.00047049	0.00436334
Gm3488	-3.2027	-1.3720	12.2066	0.00047621	0.00440645
Bsn	-1.2467	2.4444	12.1776	0.00048367	0.0044666
Efhd1	-1.2619	3.2901	12.1619	0.00048776	0.00449672
Npas2	-1.1617	1.5354	12.1269	0.00049701	0.00456657
Eps8l1	-1.5765	7.8085	12.0984	0.00050466	0.00462329
Lsmem1	-1.6679	2.6882	12.0733	0.00051149	0.00467995
Islr2	-1.6432	3.4214	12.0513	0.00051756	0.00471963
Ankrd35	-1.5407	7.1805	12.0487	0.00051829	0.00472433
Fxyd1	-1.0879	6.7792	12.0373	0.00052148	0.00474941
Gng4	-1.8316	4.0481	12.0216	0.00052587	0.00477745
Dnajc6	-1.2606	1.9380	12.0125	0.00052845	0.00479292
Fcgbp	-1.5770	7.9291	12.0050	0.00053059	0.00481036
Tnfrsf11b	-1.6136	3.2816	11.9784	0.00053819	0.00486917
Dlx5	-1.3920	2.3591	11.9711	0.00054032	0.00488637
D6Ertd527e	-1.4113	0.9402	11.9369	0.00055034	0.00496669
Trpm3	-1.3953	0.2653	11.9315	0.00055191	0.00497884
Trim71	-2.1557	-0.4868	11.9084	0.00055883	0.00503498
Nkx1-2	-2.8540	-1.3057	11.9020	0.00056074	0.00504719
Dact2	-2.0877	2.7581	11.8795	0.00056756	0.00509895
Nrg2	-1.3936	1.1324	11.8661	0.00057166	0.00513154
Rimbp2	-1.3122	1.2731	11.8635	0.00057243	0.00513489
Mef2b	-1.4629	-0.0509	11.8633	0.0005725	0.00513489
Sbsn	-1.1968	11.3167	11.8612	0.00057316	0.0051368
Susd2	-1.5209	8.3183	11.8114	0.00058868	0.00526051
Krt24	-1.9020	0.3325	11.8066	0.00059022	0.00527214
Crct1	-1.4472	8.1184	11.7726	0.00060109	0.00535823
F830045P16Rik	-2.5646	2.9356	11.7469	0.00060943	0.00541714
Tmem233	-2.0655	4.8330	11.7381	0.00061232	0.00543178
Paqr8	-1.3720	4.8565	11.7190	0.00061864	0.00547666

Robo1	-1.6292	4.6922	11.7181	0.00061894	0.00547666
Ctsw	-1.1662	2.3557	11.7065	0.00062283	0.00550712
Pdzd2	-1.0468	7.4806	11.6984	0.00062554	0.00552658
Ppl	-1.2267	9.1926	11.6841	0.00063038	0.0055603
Scd3	-1.7844	4.9348	11.6832	0.00063067	0.00556065
Heph	-1.0570	4.6461	11.6699	0.0006352	0.00559379
Pla2g3	-2.0957	3.0460	11.6629	0.00063759	0.00561256
Ccdc187	-1.1833	2.0995	11.6591	0.00063888	0.00561943
Fhl3	-1.2242	7.5793	11.6571	0.00063958	0.00562022
Ackr2	-1.0814	4.8397	11.6482	0.00064263	0.0056388
Gm5941	-3.1769	-1.1202	11.6267	0.00065011	0.00568844
Zfp773	-1.1116	2.3060	11.5360	0.00068263	0.00590198
Ppp1r36	-2.1436	-0.6349	11.5188	0.00068897	0.00595102
Nos1	-1.4903	5.5203	11.5184	0.00068912	0.00595102
Sytl1	-1.2718	6.4481	11.5106	0.00069199	0.00597111
Gapdh	-1.1682	8.9140	11.5019	0.00069523	0.00599323
Serpinb8	-1.1319	6.5358	11.4945	0.00069802	0.00601305
Slc2a5	-1.2364	3.3615	11.4793	0.00070377	0.00604881
Cyp2b19	-1.7030	6.4665	11.4776	0.00070441	0.00605197
Cpxm2	-1.3524	5.5014	11.4691	0.00070763	0.00607247
Rnf225	-1.3625	6.4743	11.4038	0.00073296	0.00625393
Mmaa	-1.0812	1.9740	11.3706	0.00074614	0.00634912
Msx2	-1.3842	1.0640	11.3506	0.00075422	0.00639931
Shisa4	-1.4473	5.8356	11.2908	0.00077892	0.00657564
Defb1	-1.0287	4.5681	11.2475	0.00079729	0.00670475
Ccdc42	-2.5007	-1.1903	11.2384	0.00080122	0.0067327
Il12rb2	-1.6515	3.7328	11.2162	0.00081087	0.0067928
Rnf165	-1.3296	1.3989	11.2075	0.00081465	0.00682074
Oas1c	-1.1380	2.5382	11.2020	0.00081708	0.00683174
Ssxb5	-3.0902	-1.5036	11.1694	0.00083157	0.00692933
Nrk	-1.4776	0.0112	11.1485	0.00084099	0.00698471
S1pr5	-1.2335	4.7454	11.1482	0.00084113	0.00698471
Skint7	-2.4671	3.7496	11.1180	0.00085492	0.007083
Sebox	-2.3886	2.1526	11.0894	0.00086822	0.00716329
AA986860	-1.0811	5.5263	11.0751	0.00087495	0.00720793
Dkkl1	-1.5093	5.7269	11.0541	0.00088493	0.00727641
Slc13a4	-1.1688	0.6201	11.0362	0.00089352	0.00733873
Anxa8	-1.0488	9.9991	11.0266	0.00089815	0.00737127
Rcsd1	-1.0580	6.2892	11.0204	0.00090116	0.0073821
Tacstd2	-1.1856	8.6022	11.0184	0.00090214	0.00738733
Grm4	-1.9288	0.6875	11.0065	0.00090793	0.00742082
Grhl1	-1.0080	7.4366	10.9993	0.00091148	0.00744426
Skint2	-2.0342	3.8749	10.9834	0.0009193	0.00749969
Tesc	-2.0875	3.8870	10.9824	0.00091982	0.00750118
Adad2	-3.3660	-0.9997	10.9724	0.00092479	0.00753372
Car8	-1.6914	1.6694	10.9540	0.000934	0.00759691
Sfrp2	-1.3501	4.8059	10.9482	0.00093693	0.00761795
Aif1l	-1.6554	6.5188	10.9470	0.00093757	0.00762028
Gm45826	-4.2886	-1.8505	10.9360	0.00094311	0.00765962
Adssl1	-1.1854	8.6102	10.9021	0.00096054	0.007778
Esyt3	-1.0167	5.7302	10.8990	0.00096214	0.00778071
Neurl1a	-1.1469	5.9712	10.8805	0.00097184	0.00784046
Spag1	-1.0727	4.7837	10.8743	0.0009751	0.00785513
Cacnb1	-1.0156	6.4607	10.8644	0.00098029	0.00788825
Pxmp2	-1.1074	3.6259	10.8475	0.00098927	0.00795391
Cpne5	-2.9947	-1.2195	10.8470	0.00098955	0.00795391
H2-M2	-1.3427	4.0664	10.8363	0.00099531	0.00799141
Ly6g6c	-1.0948	8.1725	10.8284	0.00099955	0.00801951
Akr1c14	-1.6013	3.6071	10.8072	0.00101106	0.00810293
Epm2a	-1.0137	4.9890	10.8053	0.00101213	0.00810852
Mrgprg	-2.8810	1.3441	10.7951	0.00101769	0.00814715
Hebp2	-1.1199	6.5068	10.7656	0.00103405	0.00825989
Atp1a2	-1.0992	9.5940	10.7315	0.00105329	0.00838906

Clec3b	-1.0159	7.3102	10.7263	0.00105622	0.00840934
Emp2	-1.1765	8.3002	10.7180	0.00106098	0.00843181
Lce1l	-2.0567	7.0263	10.7029	0.00106969	0.00847635
Pik3cb	-1.2432	4.0561	10.6797	0.0010832	0.00857407
Fam81a	-1.0640	4.7444	10.6519	0.00109955	0.00867209
Rasl10a	-1.6281	2.7306	10.6469	0.00110254	0.00868623
Smim24	-1.0724	1.8624	10.6247	0.00111588	0.0087618
Ripor3	-1.1279	4.2924	10.6213	0.0011179	0.00876962
Gatsl2	-1.2353	5.8700	10.6022	0.00112952	0.00884771
Kctd4	-1.2594	4.5650	10.6009	0.00113032	0.00884941
Icam5	-1.2375	0.5609	10.5948	0.00113408	0.00887389
Wnt7b	-1.5809	6.0387	10.5861	0.00113941	0.00889329
Fcho1	-1.6661	4.8020	10.5846	0.00114031	0.00889708
Hyal4	-1.4018	1.4886	10.5804	0.00114292	0.00891432
Nlrp10	-1.6365	6.2023	10.5564	0.00115787	0.00901479
Fndc9	-1.4822	6.9555	10.5457	0.00116458	0.00905414
Aff3	-1.4567	4.9871	10.5450	0.00116501	0.00905422
Stag3	-2.9726	-0.8864	10.5316	0.0011735	0.00910403
Sh3gl2	-1.3883	3.6675	10.5026	0.00119205	0.00923148
Prima1	-1.6561	0.4984	10.5014	0.00119287	0.00923394
Col4a4	-1.1553	2.6873	10.4921	0.00119887	0.00927443
Engase	-1.2014	2.7125	10.4829	0.00120485	0.00930098
Clic3	-1.4504	6.3650	10.4712	0.00121249	0.00935331
Cd207	-1.5640	4.6706	10.4411	0.00123241	0.00948355
Hepacam2	-1.6561	1.4017	10.4316	0.00123874	0.00952555
Cldn23	-1.9298	4.8218	10.4295	0.00124016	0.00952947
Txlnb	-1.1820	8.0069	10.4241	0.00124379	0.00954417
Tnnc2	-1.1742	11.1432	10.4227	0.00124473	0.00954468
Pls1	-2.0030	0.6365	10.4082	0.0012546	0.00960689
Mstn	-2.2697	6.5275	10.4058	0.00125621	0.00961581
Aldoa	-1.0205	12.9086	10.3913	0.00126612	0.00968149
Ocln	-1.0547	4.4967	10.3474	0.0012966	0.00988
Scin	-1.2893	5.5699	10.3474	0.00129661	0.00988
2310050C09Rik	-1.8629	8.5380	10.3297	0.00130904	0.00996777
Prkcq	-1.2142	5.7074	10.3198	0.00131611	0.01001116
Acer1	-1.3321	6.8450	10.3003	0.00133007	0.01009625
Ptp4a3	-1.0189	8.7632	10.2969	0.00133256	0.01010811
Serpina12	-1.9566	6.7075	10.2714	0.00135112	0.0102381
Otop3	-1.7162	3.6110	10.2359	0.00137732	0.01038268
Pou3f1	-1.1508	5.9997	10.2147	0.00139329	0.01047774
Tmem52	-1.1451	4.1719	10.2048	0.00140073	0.01052646
Gal3st2c	-1.6657	-0.2938	10.1763	0.00142258	0.01066126
Hoxa2	-1.1389	1.1201	10.1752	0.00142341	0.01066388
Pgm2	-1.0243	7.7047	10.1699	0.00142749	0.01068346
Ccr4	-2.0779	2.3787	10.1524	0.00144111	0.01077056
Smim18	-3.5607	-1.3747	10.1394	0.0014513	0.0108346
Lhfpl1	-1.3938	1.4649	10.1384	0.00145216	0.0108346
Abcc6	-2.4359	-1.2260	10.1235	0.00146391	0.01088881
Hrc	-1.2791	8.0086	10.1208	0.00146608	0.01089915
Fsd2	-1.0992	5.7864	10.0938	0.00148772	0.01103586
Kcnh6	-4.2163	-1.8756	10.0829	0.00149654	0.01109336
Mill1	-1.1677	3.0656	10.0730	0.00150454	0.01114542
Mylk4	-2.4097	8.1561	10.0708	0.00150635	0.01115007
Glcci1	-1.7234	2.3991	10.0421	0.00153004	0.01127454
Slc12a5	-1.3551	1.3151	10.0390	0.00153261	0.01128442
A630010A05Rik	-1.3485	0.6724	10.0260	0.00154345	0.01134897
Lce1c	-2.0147	7.9096	10.0169	0.00155114	0.01138081
Cbarp	-1.1517	4.7464	10.0044	0.00156169	0.01144836
Dapl1	-2.1860	6.7811	10.0028	0.00156301	0.01145038
Olfr1293-ps	-2.7586	-1.3515	10.0022	0.00156357	0.01145065
Cd55b	-1.2542	1.1511	9.9971	0.00156789	0.01147456
Dpf3	-1.2907	2.2760	9.9913	0.00157282	0.01150678
Sall4	-1.7268	-0.3182	9.9524	0.0016064	0.01172493

Apol7a	-2.1974	2.9959	9.9447	0.00161317	0.01175864
Cplx2	-1.0686	1.9960	9.9418	0.00161568	0.01177297
Gna14	-1.2297	3.3253	9.9394	0.0016178	0.01178024
Pvalb	-1.2273	10.3614	9.9059	0.00164752	0.0119492
Rhebl1	-1.1306	1.8440	9.8916	0.00166037	0.01203005
Lor	-2.1884	12.0003	9.8672	0.00168247	0.01215022
Rilpl1	-1.1653	6.5628	9.8510	0.00169736	0.01223744
Sostdc1	-1.1374	3.4061	9.8467	0.00170138	0.01225835
Amz1	-1.4736	2.1934	9.8446	0.00170328	0.01225886
Tuba3a	-4.1583	-1.9006	9.8443	0.0017036	0.01225886
Zc2hc1c	-1.0404	1.2611	9.8360	0.00171127	0.01229718
Teddm2	-1.8911	-0.9338	9.8346	0.00171256	0.01230236
Fmo2	-1.1409	5.0510	9.8214	0.00172496	0.01237112
Tgfbr3l	-1.2063	1.0012	9.8149	0.00173106	0.01240261
Fabp6	-1.3326	1.0652	9.8096	0.00173601	0.0124299
Fam166b	-1.8288	-0.8218	9.8083	0.00173722	0.01243046
Atp10b	-1.1161	4.3316	9.7830	0.00176134	0.01258651
Chit1	-1.4231	6.8613	9.7741	0.0017699	0.01263527
Krt2	-3.0567	-0.1848	9.7467	0.00179641	0.01278688
Frmd7	-6.1655	-0.7838	9.7447	0.00179844	0.01279719
Ppil6	-2.0202	0.3392	9.7288	0.00181406	0.01288982
Acad10	-1.0366	2.3304	9.7284	0.00181441	0.01288982
Dlx3	-1.2725	4.8432	9.7264	0.00181643	0.01289947
Atp2a1	-1.2985	13.3335	9.7077	0.00183497	0.01300036
Vldlr	-1.0989	6.9532	9.6905	0.00185225	0.0130947
Tmtc1	-1.0343	4.5280	9.6865	0.00185628	0.0131147
Vsig10	-1.1327	3.1393	9.6829	0.00185989	0.01313596
Kremen2	-1.7702	2.1081	9.6595	0.0018838	0.01326619
Gdnf	-1.0403	2.7708	9.6268	0.00191756	0.01346053
Guca2a	-2.0107	1.9391	9.6256	0.00191889	0.01346119
Serpina5	-2.2717	0.0391	9.6060	0.00193946	0.0135975
Tmem132b	-2.6947	-0.8325	9.5858	0.00196084	0.0137114
Lce1b	-1.9597	8.0711	9.5770	0.00197028	0.01375099
Lce1a1	-1.8617	8.5723	9.5598	0.0019888	0.01385811
Krt23	-1.8520	7.8093	9.5572	0.00199164	0.01386464
Scel	-1.2364	7.4711	9.5521	0.00199726	0.01389048
Zfp831	-1.3373	0.3642	9.5415	0.00200881	0.01395299
Egr1	-1.0998	8.1364	9.5279	0.00202368	0.01402507
Ryr1	-1.1671	10.5682	9.5250	0.00202697	0.01404251
Fosb	-1.8890	6.4337	9.5235	0.00202856	0.01404554
Cd96	-1.7054	1.3890	9.5192	0.00203333	0.01407407
Mb	-1.1825	8.7115	9.5065	0.00204741	0.01414744
Spice1	-1.1047	2.7926	9.5037	0.00205054	0.01416178
Fam25c	-1.5670	8.8112	9.4941	0.00206128	0.01421796
9930012K11Rik	-1.2578	3.6613	9.4911	0.0020647	0.01423704
P2rx6	-1.1807	3.0854	9.4410	0.00212187	0.01457597
Adamts16	-4.0000	-1.1046	9.4359	0.00212779	0.01461206
Nme5	-1.5503	-0.3095	9.4273	0.00213781	0.01465784
Myh4	-1.3580	13.7736	9.4198	0.0021466	0.01469966
Gm3776	-1.3818	1.6884	9.3998	0.00217008	0.01482784
Col11a1	-1.6097	0.0706	9.3940	0.00217692	0.0148653
Ntrk3	-1.0997	2.3941	9.3566	0.0022218	0.01511034
P4htm	-1.0211	2.7498	9.3524	0.00222689	0.01513552
Neb	-1.1359	10.4741	9.3518	0.00222771	0.01513638
Pifo	-3.1210	-1.5900	9.3438	0.00223738	0.01519734
Lrrn1	-1.0809	2.8401	9.3141	0.00227392	0.01541413
Zfp707	-1.1957	3.1869	9.2935	0.00229967	0.0155577
Aloxe3	-1.4289	6.4912	9.2753	0.00232265	0.01566928
Hoxa6	-1.4085	1.2182	9.2634	0.00233784	0.015742
Fsbp	-1.4798	0.3509	9.2629	0.00233848	0.015742
Nudt17	-1.0904	4.0258	9.2436	0.00236319	0.01586434
Tppp	-1.1224	6.1384	9.2389	0.00236926	0.01590021
Osbpl6	-1.5197	2.3304	9.2325	0.00237759	0.01595117

Hcfc1r1	-1.1791	6.3687	9.2211	0.00239237	0.01603559
Gm2916	-3.9993	-1.9588	9.2030	0.00241621	0.0161606
Olfr173	-1.3786	-0.2637	9.1752	0.0024532	0.0163584
Stab2	-1.4844	0.3355	9.1705	0.00245951	0.01638497
Ckm	-1.0806	12.2318	9.1660	0.00246554	0.01641013
Rgs9	-1.2715	0.9098	9.1197	0.00252869	0.01675893
Cytl1	-1.0289	2.5229	9.1123	0.00253897	0.01681578
Nova1	-1.1111	4.7743	9.1105	0.00254141	0.01682281
Map2k6	-1.1245	3.3466	9.0941	0.00256435	0.01693353
Tnnt3	-1.1620	12.2208	9.0690	0.00259985	0.01714664
Ccdc155	-4.0032	-1.9581	9.0240	0.00266455	0.01746842
Calcb	-1.2708	3.9589	9.0202	0.00267009	0.01748998
Kcnk7	-1.1928	3.7068	9.0201	0.0026703	0.01748998
Cox6a2	-1.0538	7.6919	8.9743	0.00273809	0.01786468
Alox12e	-2.0894	7.4486	8.9542	0.00276831	0.01801341
Lce1a2	-1.9418	8.1849	8.9488	0.00277644	0.01806095
Tmod4	-1.1155	6.8519	8.9452	0.00278205	0.01808122
Xlr3a	-3.3202	-1.4932	8.9287	0.00280732	0.01821365
Olfr1129	-1.6204	-0.5047	8.9226	0.00281663	0.01825706
Ccdc63	-2.8287	-0.5638	8.8999	0.00285184	0.01844697
Lax1	-1.1929	2.0637	8.8953	0.00285911	0.01847758
Slc16a11	-1.4280	1.8646	8.8790	0.0028847	0.01856601
Acss1	-1.3269	6.7616	8.8769	0.00288806	0.01858218
Frmpd1	-1.3560	4.7188	8.8745	0.00289189	0.01860133
Gabrq	-1.9937	-1.0428	8.8616	0.00291237	0.01869996
Stx19	-1.0203	3.2940	8.8477	0.00293465	0.01879333
Wnt3	-1.6664	6.0414	8.8389	0.00294878	0.0188561
Hoxb6	-1.0318	2.1978	8.8314	0.0029609	0.01890914
Nccrp1	-1.3613	7.8705	8.8291	0.00296461	0.01891305
Gm94	-1.3899	7.3247	8.8058	0.00300276	0.0191173
Csnk2a2	-1.0353	7.5558	8.7738	0.00305586	0.01938751
Lipn	-1.2876	4.0265	8.7646	0.00307135	0.01946882
Scml2	-1.0684	1.9341	8.7432	0.00310761	0.01965871
Pbx4	-2.1326	-0.6727	8.7202	0.00314701	0.01985615
Lce1d	-1.8266	7.2550	8.7166	0.00315326	0.01988548
Cysrt1	-1.1748	6.2097	8.7070	0.00316995	0.0199663
Ldlrad1	-4.3299	-1.8419	8.7040	0.00317507	0.01998703
Bdh2	-1.4021	0.6804	8.6907	0.0031983	0.02009844
Tnfsf15	-1.9776	-0.3610	8.6875	0.00320407	0.02011735
Cks1brt	-1.9637	-1.0462	8.6537	0.00326396	0.02038337
Ptgs1	-1.0825	7.4177	8.6473	0.00327538	0.02044163
Slc4a9	-1.9972	-0.0510	8.6397	0.00328915	0.02052168
Mrln	-1.5620	2.7721	8.6225	0.00332032	0.02067483
Slc27a2	-1.8418	1.4340	8.5965	0.00336816	0.02093093
Tnni2	-1.0340	11.1836	8.5899	0.00338033	0.02099461
Alox12	-1.2853	4.2423	8.5558	0.00344427	0.02127635
Olfm2	-1.2271	3.7109	8.5548	0.00344616	0.02127635
Gm14440	-4.8419	0.8948	8.5492	0.00345668	0.02131657
Lzts1	-1.1519	1.7811	8.5490	0.00345705	0.02131657
Krt222	-1.0099	1.0371	8.5455	0.00346384	0.02133723
Ccdc113	-1.4305	1.9874	8.5246	0.00350377	0.02152245
Ovol2	-1.1020	2.2588	8.5190	0.00351464	0.02158313
Gm4775	-4.1610	-1.9004	8.5180	0.00351645	0.02158821
Cyp2w1	-2.0877	2.0084	8.5149	0.00352252	0.02161937
Tpm2	-1.0649	11.8180	8.5131	0.00352595	0.02163437
Itga11	-1.3049	4.1319	8.4975	0.00355637	0.02176616
Asb10	-1.1330	4.8631	8.4793	0.00359218	0.02194817
Gfra4	-5.3471	-1.3585	8.4750	0.00360054	0.02197468
Crybg2	-1.1143	6.3762	8.4669	0.0036167	0.02205478
Mfsd4b5	-1.4473	-0.1007	8.4376	0.00367539	0.02232537
Olfr1416	-1.9015	-0.7361	8.4188	0.00371355	0.02252581
Gramd1c	-1.2614	5.1554	8.4058	0.00374023	0.02264365
Aox3	-1.0596	0.6693	8.3767	0.00380058	0.02293459

BC016579	-1.0707	2.9367	8.3715	0.00381154	0.02296706
Krtap3-2	-2.2824	5.2036	8.3566	0.00384291	0.02311339
Lypd6b	-1.1188	4.6518	8.3561	0.00384391	0.02311339
Clca3a2	-1.6103	9.1205	8.3559	0.00384429	0.02311339
Cd3g	-1.1388	3.4868	8.3517	0.00385326	0.0231482
Olfr1342	-3.1826	-1.5481	8.3471	0.00386314	0.02319478
Phf21b	-1.0573	1.3260	8.3449	0.00386771	0.02320304
Cacna1s	-1.1329	8.4339	8.3326	0.00389404	0.02330341
Il20ra	-2.4504	5.7565	8.3183	0.00392471	0.02346122
Gdpd2	-1.3678	5.6465	8.3176	0.00392624	0.02346392
Mroh6	-1.0669	3.9063	8.2992	0.00396635	0.02359383
Lce1m	-2.4791	9.6182	8.2957	0.00397388	0.02362377
Rnase2a	-1.7420	1.3535	8.2838	0.00399996	0.0237162
Trp53inp1	-1.2951	5.0079	8.2709	0.00402856	0.02384118
Ttn	-1.0719	12.0856	8.2666	0.00403803	0.02386418
Srcin1	-1.7930	6.0243	8.2409	0.00409574	0.02415108
C130060K24Rik	-4.8195	-1.5973	8.2184	0.00414667	0.02441338
Abca6	-1.3240	3.0375	8.2113	0.00416313	0.02448419
Ddit4l	-2.2606	5.5264	8.2067	0.00417349	0.02453187
Zfp952	-1.0004	2.5799	8.2006	0.00418771	0.02460222
Zmat4	-2.8969	-0.6841	8.1906	0.00421069	0.02470399
Etv2	-3.9882	-1.9602	8.1718	0.00425477	0.02492241
Lrrc17	-1.0521	3.6192	8.1611	0.00427985	0.02504248
Tmem229a	-1.5688	2.6379	8.1596	0.00428328	0.02505582
Chrne	-1.7509	0.2632	8.1169	0.0043855	0.02554425
Gm996	-1.9896	-0.1538	8.1140	0.00439254	0.02557348
Asb14	-1.3324	4.1155	8.1088	0.00440519	0.02561795
Cacnb2	-1.3653	0.0367	8.1027	0.00442003	0.02568368
Srl	-1.0093	8.9572	8.0953	0.004438	0.0257744
Hrasls	-1.1984	3.3590	8.0925	0.00444495	0.02580791
Ucp1	-3.0140	-1.6368	8.0697	0.00450121	0.02606524
Wnt3a	-1.1067	2.8125	8.0661	0.0045102	0.02610344
Olfr720	-2.8990	-1.6862	8.0532	0.00454222	0.02624006
Edar	-1.2898	-0.1381	8.0459	0.00456058	0.02631826
Acpp	-1.0802	7.8227	8.0450	0.00456297	0.02632512
Rgag1	-2.3541	-1.2646	8.0408	0.00457364	0.02635182
Pde4dip	-1.1346	10.4546	8.0395	0.00457679	0.02635605
Hus1b	-3.8153	-2.0206	8.0383	0.00457974	0.02636011
Btc	-1.7785	5.7110	8.0335	0.00459205	0.02642304
Sh2d7	-1.2763	1.3064	8.0179	0.00463183	0.02661689
Actn3	-1.2685	10.6955	8.0134	0.0046433	0.02666174
Serpinb9b	-1.3475	1.4357	8.0088	0.00465518	0.02670184
Lgi3	-1.0652	0.5328	8.0047	0.00466556	0.02674735
Bco2	-1.1166	1.7376	7.9958	0.00468853	0.02681562
Oas1e	-2.0634	1.6617	7.9939	0.00469342	0.0268366
Mapk13	-1.0612	6.8495	7.9895	0.00470498	0.02688155
Fthl17a	-2.2193	-1.3363	7.9583	0.00478676	0.02727105
Mboat2	-1.4053	6.9212	7.9555	0.00479402	0.02730162
Skint1	-1.3571	3.7028	7.9553	0.00479476	0.02730162
Alox8	-1.4544	0.8345	7.9226	0.00488218	0.02767668
2010109I03Rik	-1.0993	4.5813	7.9035	0.00493385	0.0279044
Igsf1	-1.7467	0.2199	7.8997	0.00494427	0.02794881
Vps37d	-1.4180	-0.1963	7.8929	0.00496302	0.02803303
Il17rb	-1.2768	1.4746	7.8775	0.00500533	0.02822864
Paqr5	-1.3880	4.8254	7.8636	0.00504405	0.02841658
Kprp	-1.5697	9.0040	7.8612	0.00505055	0.02843914
Cnih2	-1.5879	0.8028	7.8549	0.00506836	0.02853205
Dpp4	-1.0318	5.9257	7.8450	0.00509625	0.02865216
Ctnnbip1	-1.0658	6.3456	7.8268	0.00514773	0.02891178
Clca2	-1.3693	6.7788	7.8094	0.00519762	0.02911705
Olfr111	-1.8748	-0.7948	7.8077	0.0052025	0.02912944
Fgf22	-1.2477	3.0610	7.7852	0.00526759	0.02944861
Lrtm1	-1.4565	-0.0466	7.7770	0.00529157	0.02952933

Lbx1	-1.0001	3.6144	7.7761	0.0052942	0.02952933
Gria2	-3.1601	-1.4056	7.7642	0.00532916	0.02971672
Car6	-2.1352	3.7917	7.7608	0.00533926	0.02976541
Tcp10a	-3.8294	-2.0185	7.7501	0.00537081	0.02990313
Snph	-1.7001	-0.4251	7.7399	0.00540137	0.03001206
Gjb5	-1.5489	6.0271	7.7150	0.00547634	0.03038993
Kif19a	-1.0722	0.5039	7.7101	0.00549131	0.03044977
Gm9821	-1.2101	3.0757	7.7016	0.00551733	0.03057852
Olfr1410	-2.2027	-1.3339	7.6971	0.00553106	0.03064686
Zfp459	-1.1058	0.6395	7.6632	0.00563581	0.03110882
Ccdc142	-2.9643	-1.1055	7.6315	0.00573588	0.03161323
Bhlha9	-2.6316	-0.8708	7.6269	0.00575052	0.03166193
Plcxd2	-1.2142	4.2271	7.6215	0.00576757	0.03172383
Islr	-1.0398	5.4488	7.6172	0.00578143	0.03178402
Defb15	-2.8983	-1.6871	7.6150	0.00578853	0.03181503
Pla2g2f	-1.0983	8.3656	7.5994	0.00583891	0.03202743
Krtdap	-1.0692	10.2315	7.5947	0.00585412	0.03207057
4932411N23Rik	-2.0030	-0.8776	7.5927	0.0058605	0.0320975
Agtr2	-1.5118	-0.2635	7.5719	0.00592863	0.0323489
Krt78	-1.3511	8.1591	7.5650	0.00595122	0.03243164
Rgs6	-1.3299	0.9287	7.5490	0.00600432	0.03267587
Mt4	-2.3069	5.2600	7.5376	0.00604228	0.0328132
Rhbdl2	-1.2415	3.8975	7.5365	0.006046	0.0328252
Asb18	-1.3289	3.1246	7.5332	0.00605715	0.03286943
Tfr2	-1.3630	0.5626	7.5309	0.00606482	0.03289055
Map2	-1.0101	6.0742	7.5307	0.00606557	0.03289055
Gal3st4	-1.4348	3.8244	7.5196	0.0061032	0.03305359
Fmo6	-3.8208	-2.0197	7.5159	0.00611583	0.03310556
Tmem54	-1.0156	6.0465	7.5140	0.00612199	0.03312248
Gp1bb	-1.1685	3.0352	7.5094	0.00613785	0.03319184
Rpl9-ps6	-2.8953	-0.8260	7.5004	0.00616859	0.0333168
Il18	-1.1290	5.7399	7.4773	0.00624823	0.03364706
Hmgcs2	-1.0268	5.5201	7.4717	0.0062678	0.03374408
Lce1i	-1.4762	6.2563	7.4634	0.00629673	0.03381525
Lrrc74b	-1.7143	1.9896	7.4630	0.00629805	0.03381525
Gprasp2	-1.6309	-0.0478	7.4510	0.00634024	0.03397492
Mlana	-2.4601	-1.5028	7.4400	0.00637913	0.0341498
Celf4	-1.0462	2.7424	7.4390	0.00638252	0.03415716
Olfr133	-2.3580	-1.5468	7.4311	0.00641055	0.03422568
Clca1	-1.5843	2.6254	7.4292	0.0064175	0.03423764
Mybpc2	-1.0894	10.7671	7.3951	0.00654035	0.03480798
Rgs11	-3.5304	-1.1873	7.3730	0.00662116	0.0351866
Ky	-1.1880	5.5032	7.3706	0.00662977	0.0352152
Dmkn	-1.0482	11.5693	7.3527	0.00669621	0.03549042
Atp2b3	-1.3338	1.0063	7.3457	0.00672224	0.03560244
4921507P07Rik	-1.5518	-0.0505	7.3440	0.00672852	0.03562703
Col2a1	-2.8175	-1.7195	7.3419	0.00673649	0.03565194
Hoxa7	-1.2719	5.4566	7.3410	0.0067399	0.03565273
Slc27a5	-2.7794	-1.6302	7.3367	0.00675592	0.0357013
Olfr348	-3.8207	-2.0198	7.3279	0.00678935	0.03583616
Napb	-7.2016	-0.0274	7.3153	0.00683684	0.03600281
Slc35f1	-1.1477	0.3721	7.3028	0.00688476	0.036191
Dscaml1	-3.6662	-2.0680	7.3008	0.00689249	0.03621418
Ttll10	-1.1928	0.7963	7.2979	0.00690347	0.03625442
Hfe2	-1.1106	7.0316	7.2949	0.00691509	0.03629801
Vit	-1.0047	3.2146	7.2921	0.00692566	0.03633267
Aqp4	-1.5588	3.4375	7.2919	0.0069267	0.03633267
Hcn2	-1.0383	1.1249	7.2777	0.00698175	0.03655996
Agrp	-1.5044	0.5442	7.2733	0.00699879	0.03657678
BC048671	-2.7801	-1.7390	7.2707	0.00700883	0.03657678
Rsrp1	-1.0637	4.9255	7.2581	0.00705834	0.03678457
Pitx3	-1.3665	1.4675	7.2475	0.00709981	0.03695657
Serpinb7	-1.4662	6.6573	7.2230	0.00719769	0.03736806

Mapk15	-1.8344	0.1546	7.2149	0.00723026	0.0374748
2310046K23Rik	-1.0163	8.2818	7.2122	0.00724095	0.03751787
Bbox1	-1.6051	6.2016	7.2073	0.00726087	0.03759844
Plcxd1	-1.1025	1.7968	7.1660	0.00742991	0.03832758
Gm15737	-1.4517	1.3896	7.1541	0.0074791	0.03853944
Cap2	-1.0095	6.4430	7.1490	0.00750057	0.0386191
Smim5	-1.0927	3.5635	7.1460	0.00751319	0.0386476
Efnb3	-1.0578	1.1549	7.1286	0.00758655	0.03897902
Gpr50	-3.7077	-2.0539	7.1114	0.00765973	0.03927187
Phkb	-1.0545	7.1238	7.0986	0.00771431	0.0394775
Elmod1	-1.2938	5.5973	7.0808	0.00779155	0.03977953
D630003M21Rik	-1.0723	0.3818	7.0665	0.007854	0.03993959
Ugt3a2	-1.9571	0.7621	7.0567	0.00789711	0.04010277
Ndufa4l2	-1.5631	6.8323	7.0504	0.00792472	0.04021491
Sdcbp2	-1.0553	5.6471	7.0480	0.00793525	0.04025508
Nlrp1a	-1.6281	1.2758	7.0466	0.00794164	0.04027269
Mrgprb3	-1.9132	3.1279	7.0408	0.00796749	0.04037564
Ankfn1	-3.7443	-2.0468	7.0385	0.00797741	0.04040717
Slc25a21	-3.0239	-1.6390	7.0225	0.00804931	0.04067695
Asmt	-3.6097	-2.0843	7.0200	0.00806053	0.04071483
Ogn	-1.1210	6.0950	7.0046	0.00813024	0.04097213
Cux2	-1.0866	0.1928	6.9930	0.00818282	0.04113268
Tpm1	-1.0366	11.2931	6.9560	0.00835366	0.04177021
Kir3dl2	-1.1853	1.2392	6.9420	0.00841942	0.04204123
Shisa3	-1.2916	0.4788	6.9176	0.00853513	0.04252177
Il22ra2	-1.4231	4.2091	6.9136	0.00855432	0.04258823
Itpka	-1.0425	0.9738	6.9092	0.00857546	0.04266431
Wfdc9	-3.6160	-2.0832	6.9014	0.00861289	0.04277251
1700007K09Rik	-2.0135	-1.2227	6.8936	0.00865025	0.04293852
Gabrr1	-2.7715	-1.3399	6.8668	0.0087813	0.04349017
Arhgap20	-1.3308	2.9129	6.8608	0.00881092	0.04360719
Rgs9bp	-1.0860	1.5006	6.8475	0.00887639	0.04387152
Riiad1	-1.5632	0.3903	6.8434	0.00889686	0.04396274
Dusp26	-1.1045	2.4097	6.8305	0.00896155	0.04423235
Hs3st5	-1.3438	0.8357	6.8244	0.00899229	0.04434396
Apol9a	-1.5449	1.9212	6.8107	0.00906108	0.04462273
Tssk1	-1.6762	-1.0557	6.7954	0.00913921	0.04486576
Capn9	-1.1592	1.5858	6.7930	0.00915168	0.04489671
5430419D17Rik	-1.2201	1.1822	6.7909	0.00916253	0.04493986
Pou1f1	-2.5763	-1.4304	6.7782	0.00922755	0.04522827
Sap25	-2.0665	2.3486	6.7694	0.00927345	0.04538569
Gm27021	-2.3236	-1.2146	6.7598	0.00932353	0.04558609
Olfr1245	-3.5536	-2.1007	6.7535	0.0093566	0.04571135
S100g	-2.2105	-1.3365	6.7382	0.00943711	0.04598693
Lce6a	-1.5187	5.5218	6.7269	0.00949701	0.04619633
Ace2	-1.0136	5.0854	6.7215	0.00952585	0.04631261
Gm28308	-2.0587	-1.2668	6.7199	0.00953449	0.04633737
Gsta2	-1.2677	2.9957	6.7181	0.00954377	0.04637213
Adgre4	-1.0082	2.0504	6.6964	0.00966078	0.04682603
Fgf20	-2.2381	-0.9094	6.6842	0.00972685	0.04710443
2410137M14Rik	-2.0117	-0.3393	6.6770	0.0097663	0.04720127
Fam131b	-1.0456	1.6595	6.6272	0.01004338	0.04828386
Lmntd2	-1.1805	1.4293	6.6216	0.0100752	0.04839424
Adgrv1	-1.2381	0.5251	6.6190	0.01008953	0.04844175
Hal	-1.1825	8.3873	6.6177	0.0100971	0.04846742
4930412O13Rik	-1.2630	0.7268	6.5852	0.01028274	0.04914239

Supplementary Table S5. The KEGG pathways upregulated in the CHS samples compared with the VT controls

Pathway ID	Pathway description	Nominal P-value
mmu00100	Steroid biosynthesis	9.81E-003
mmu00565	Ether lipid metabolism	2.67E-003
mmu00590	Arachidonic acid metabolism	1.43E-003
mmu00591	Linoleic acid metabolism	3.30E-004
mmu00592	alpha-Linolenic acid metabolism	1.24E-003
mmu00240	Pyrimidine metabolism	1.61E-002
mmu00563	Glycosylphosphatidylinositol (GPI)-anchor biosynthesis	1.16E-002
mmu00670	One carbon pool by folate	1.06E-002
mmu00900	Terpenoid backbone biosynthesis	1.37E-002
mmu00232	Caffeine metabolism	2.94E-003
mmu00524	Neomycin, kanamycin and gentamicin biosynthesis	1.55E-002
mmu00983	Drug metabolism - other enzymes	1.76E-002
mmu03040	Spliceosome	6.14E-003
mmu03013	RNA transport	1.47E-002
mmu03015	mRNA surveillance pathway	1.07E-002
mmu03018	RNA degradation	6.29E-003
mmu03030	DNA replication	7.26E-004
mmu03410	Base excision repair	8.60E-003
mmu03420	Nucleotide excision repair	7.87E-003
mmu03430	Mismatch repair	1.71E-003
mmu03440	Homologous recombination	4.22E-003
mmu03450	Non-homologous end-joining	1.23E-003
mmu03460	Fanconi anemia pathway	3.10E-003
mmu04370	VEGF signaling pathway	8.95E-005
mmu04064	NF-kappa B signaling pathway	7.10E-003
mmu04668	TNF signaling pathway	3.41E-003
mmu04110	Cell cycle	2.02E-003
mmu04210	Apoptosis	3.85E-003
mmu04215	Apoptosis - multiple species	8.39E-004
mmu04217	Necroptosis	6.79E-004
mmu04622	RIG-I-like receptor signaling pathway	2.29E-003
mmu04657	IL-17 signaling pathway	7.28E-004
mmu04664	Fc epsilon RI signaling pathway	5.94E-003
mmu04913	Ovarian steroidogenesis	2.43E-003
mmu04914	Progesterone-mediated oocyte maturation	3.05E-003
mmu04978	Mineral absorption	6.15E-004
mmu04966	Collecting duct acid secretion	1.33E-002
mmu04726	Serotonergic synapse	1.35E-003
mmu04730	Long-term depression	2.86E-003

Supplementary Table S6. The KEGG pathways downregulated in the CHS samples compared with the VT controls

Pathway ID	Pathway description	Nominal P-value
mmu01210	2-Oxocarboxylic acid metabolism	1.09E-002
mmu00010	Glycolysis / Gluconeogenesis	6.50E-003
mmu00020	Citrate cycle (TCA cycle)	9.64E-003
mmu00500	Starch and sucrose metabolism	2.30E-003
mmu00620	Pyruvate metabolism	2.61E-003
mmu00640	Propanoate metabolism	4.26E-003
mmu00280	Valine, leucine and isoleucine degradation	1.45E-002
mmu00440	Phosphonate and phosphinate metabolism	1.65E-002
mmu00471	D-Glutamine and D-glutamate metabolism	8.91E-005
mmu00603	Glycosphingolipid biosynthesis - globo and isoglobo series	3.91E-004
mmu00604	Glycosphingolipid biosynthesis - ganglio series	1.15E-002
mmu00760	Nicotinate and nicotinamide metabolism	4.56E-003
mmu00785	Lipoic acid metabolism	1.05E-002
mmu04015	Rap1 signaling pathway	7.99E-003
mmu04310	Wnt signaling pathway	2.69E-003
mmu04340	Hedgehog signaling pathway	1.69E-002
mmu04371	Apelin signaling pathway	8.66E-004
mmu04068	FoxO signaling pathway	3.32E-003
mmu04020	Calcium signaling pathway	4.97E-003
mmu04024	cAMP signaling pathway	4.26E-003
mmu04022	cGMP-PKG signaling pathway	1.20E-003
mmu04152	AMPK signaling pathway	1.06E-002
mmu04080	Neuroactive ligand-receptor interaction	9.16E-004
mmu04146	Peroxisome	9.47E-003
mmu04510	Focal adhesion	1.12E-002
mmu04911	Insulin secretion	1.52E-002
mmu04910	Insulin signaling pathway	4.10E-003
mmu04922	Glucagon signaling pathway	3.18E-003
mmu04923	Regulation of lipolysis in adipocytes	3.00E-003
mmu04915	Estrogen signaling pathway	1.03E-002
mmu04921	Oxytocin signaling pathway	1.36E-002
mmu04916	Melanogenesis	8.16E-004
mmu04924	Renin secretion	6.58E-003
mmu04925	Aldosterone synthesis and secretion	6.93E-004
mmu04261	Adrenergic signaling in cardiomyocytes	1.19E-002
mmu04270	Vascular smooth muscle contraction	8.84E-003
mmu04970	Salivary secretion	3.64E-003
mmu04971	Gastric acid secretion	3.18E-003
mmu04972	Pancreatic secretion	1.81E-002
mmu04962	Vasopressin-regulated water reabsorption	1.06E-002
mmu04961	Endocrine and other factor-regulated calcium reabsorption	9.23E-003
mmu04727	GABAergic synapse	1.19E-003
mmu04725	Cholinergic synapse	1.07E-003
mmu04728	Dopaminergic synapse	3.64E-003
mmu04720	Long-term potentiation	5.56E-003
mmu04723	Retrograde endocannabinoid signaling	2.87E-003
mmu04213	Longevity regulating pathway - multiple species	1.27E-003
mmu04713	Circadian entrainment	5.48E-004
mmu04714	Thermogenesis	4.03E-003

Supplementary Table S7. The KEGG pathways upregulated in the SSS samples compared with the NT controls

Pathway ID	Pathway description	Nominal P-value
mmu01100	Metabolic pathways	1.36E-002
mmu00520	Amino sugar and nucleotide sugar metabolism	3.52E-004
mmu00230	Purine metabolism	7.65E-003
mmu00240	Pyrimidine metabolism	1.19E-002
mmu00330	Arginine and proline metabolism	6.26E-004
mmu00430	Taurine and hypotaurine metabolism	1.20E-002
mmu00510	N-Glycan biosynthesis	1.09E-004
mmu00512	Mucin type O-glycan biosynthesis	7.41E-003
mmu00532	Glycosaminoglycan biosynthesis - chondroitin sulfate / dermatan sulfate	2.58E-003
mmu00790	Folate biosynthesis	5.88E-003
mmu00860	Porphyrin and chlorophyll metabolism	9.77E-004
mmu00232	Caffeine metabolism	8.62E-004
mmu03060	Protein export	9.73E-003
mmu04141	Protein processing in endoplasmic reticulum	7.02E-004
mmu04015	Rap1 signaling pathway	1.16E-002
mmu04010	MAPK signaling pathway	5.67E-003
mmu04370	VEGF signaling pathway	5.80E-003
mmu04630	Jak-STAT signaling pathway	5.60E-004
mmu04064	NF-kappa B signaling pathway	1.42E-004
mmu04668	TNF signaling pathway	1.10E-005
mmu04066	HIF-1 signaling pathway	1.18E-002
mmu04151	PI3K-Akt signaling pathway	2.62E-003
mmu04060	Cytokine-cytokine receptor interaction	4.32E-005
mmu04512	ECM-receptor interaction	1.11E-002
mmu04514	Cell adhesion molecules (CAMs)	2.39E-003
mmu04145	Phagosome	2.54E-003
mmu04210	Apoptosis	2.05E-003
mmu04215	Apoptosis - multiple species	1.38E-002
mmu04216	Ferroptosis	5.14E-003
mmu04217	Necroptosis	3.79E-004
mmu04810	Regulation of actin cytoskeleton	2.13E-003
mmu04640	Hematopoietic cell lineage	1.03E-003
mmu04610	Complement and coagulation cascades	1.95E-003
mmu04620	Toll-like receptor signaling pathway	1.19E-005
mmu04621	NOD-like receptor signaling pathway	5.89E-005
mmu04622	RIG-I-like receptor signaling pathway	4.74E-003
mmu04623	Cytosolic DNA-sensing pathway	1.42E-005
mmu04625	C-type lectin receptor signaling pathway	2.57E-004
mmu04650	Natural killer cell mediated cytotoxicity	1.38E-002
mmu04657	IL-17 signaling pathway	2.57E-005
mmu04664	Fc epsilon RI signaling pathway	8.54E-004
mmu04666	Fc gamma R-mediated phagocytosis	1.42E-003
mmu04670	Leukocyte transendothelial migration	1.30E-003
mmu04672	Intestinal immune network for IgA production	5.39E-003
mmu04062	Chemokine signaling pathway	5.64E-005
mmu04380	Osteoclast differentiation	9.15E-004

Supplementary Table S8. The KEGG pathways downregulated in the SSS samples compared with the NT controls

Pathway ID	Pathway description	Nominal P-value
mmu00562	Inositol phosphate metabolism	2.97E-003
mmu00920	Sulfur metabolism	1.70E-003
mmu00310	Lysine degradation	1.10E-004
mmu00750	Vitamin B6 metabolism	1.07E-002
mmu00785	Lipoic acid metabolism	1.93E-003
mmu00982	Drug metabolism - cytochrome P450	3.95E-003
mmu04120	Ubiquitin mediated proteolysis	1.23E-003
mmu02010	ABC transporters	6.08E-003
mmu04012	ErbB signaling pathway	5.54E-003
mmu04310	Wnt signaling pathway	9.85E-004
mmu04330	Notch signaling pathway	1.08E-002
mmu04390	Hippo signaling pathway	1.37E-002
mmu04070	Phosphatidylinositol signaling system	4.28E-003
mmu04150	mTOR signaling pathway	2.00E-003
mmu04136	Autophagy - other	1.19E-003
mmu04137	Mitophagy - animal	1.03E-002
mmu04550	Signaling pathways regulating pluripotency of stem cells	2.35E-003
mmu04658	Th1 and Th2 cell differentiation	9.37E-003
mmu04919	Thyroid hormone signaling pathway	1.05E-002
mmu04740	Olfactory transduction	1.18E-003
mmu04742	Taste transduction	1.32E-002
mmu04360	Axon guidance	3.16E-003
mmu04710	Circadian rhythm	1.25E-003

Supplementary Table S9. The published 10-gene and 14-gene signatures

Category	Gene
10-gene lesional signature	*PI3*
	IL17A
	S100A9
	S100A7
	S100A12
	KRT16
	IL22
	FLG
	PPL
	MX1
14-gene non-lesional signature	*TREM1*
	KRT16
	ITGAX
	CCL2
	MMP12
	S100A7
	IL22
	IL13
	CXCL1
	S100A9
	FLG
	IL31
	LOR
	PPL
